# Catalytic Atroposelective C7 Functionalisation of Indolines and Indoles

**DOI:** 10.1002/chem.202103365

**Published:** 2021-11-05

**Authors:** Saad Shaaban, Christian Merten, Herbert Waldmann

**Affiliations:** ^1^ Max Planck Institute of Molecular Physiology Department of Chemical Biology Otto-Hahn-Straße 11 44227 Dortmund Germany; ^2^ Technical University Dortmund Faculty of Chemical Biology Otto-Hahn-Straße 4a 44227 Dortmund Germany; ^3^ Ruhr University Bochum Department of Organic Chemistry Universität Straße 150 44801 Bochum Germany

**Keywords:** atropisomers, C−H functionalisation, chiral Cp^x^ complexes, indoles, indolines, rhodium

## Abstract

Axially chiral atropisomeric compounds are widely applied in asymmetric catalysis and medicinal chemistry. In particular, axially chiral indole‐ and indoline‐based frameworks have been recognised as important heterobiaryl classes because they are the core units of bioactive natural alkaloids, chiral ligands and bioactive compounds. Among them, the synthesis of C7‐substituted indole biaryls and the analogous indoline derivatives is particularly challenging, and methods for their efficient synthesis are in high demand. Transition‐metal catalysis is considered one of the most efficient methods to construct atropisomers. Here, we report the enantioselective synthesis of C7‐indolino‐ and C7‐indolo biaryl atropisomers by means of C−H functionalisation catalysed by chiral Rh*Jas*Cp complexes.

## Graphical abstract

**The first enantioselective synthesis** of C7‐indolino‐ and C7‐indolo‐biaryl atropisomers by means of C−H functionalisation catalysed by our chiral rhodium complex (Rh*Jas*Cp) is reported. The reaction works for a wide range of substrates and gives excellent yields (up to 92 %) and very good level of enantioselectivity (up to 90 % *ee*). Furthermore, this transformation tolerates a variety of functional groups in both indoles and indolines.

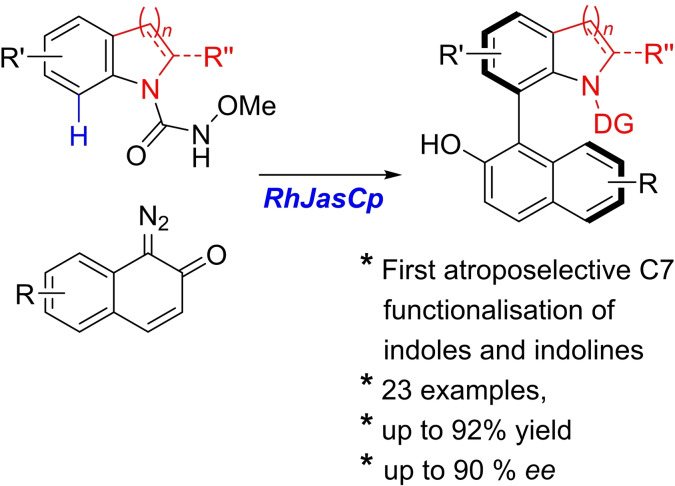

Axially chiral biaryls, have emerged as highly valuable chiral skeletons, with broad applications in drug discovery and chiral catalysis.^[1]^ This has led to the development of novel catalytic methods for the asymmetric synthesis of axially chiral biaryl scaffolds.^[2]^ In particular, axially chiral indole‐ and indoline‐based frameworks have been recognised as important classes of heterobiaryls because they are the core units of natural alkaloids, chiral ligands and bioactive compounds.[^3^, ^4^, ^5^] Direct transition‐metal mediated functionalisation of the C7‐position of indolines and indoles through directing group‐assisted C−H activation has become an efficient strategy for the synthesis of unique indoline derivatives (Scheme 1a).[^6^, ^7^, ^8^] Direct enantioselective C−H functionalisation of the indole at the C7‐position has been described in a few cases,^[9]^ but the enantioselective syntheses of C7‐substituted indole and indoline atropisomers through C−H activation has not yet been reported. Chiral transition‐metal Cp^x^ complexes have proven to be effective tools for enantioselective C−H functionalisation reactions,^[10]^ and different types of chiral Cp^x^ complexes have been successfully applied in asymmetric reactions.^[11]^ Enantioselective C−H functionalisation using chiral Cp^x^ ligands has been employed as an alternative strategy to access atropisomers. Since the early report by Heller et al. on the Co‐catalysed enantioselective synthesis of axial biaryls by means of [2+2+2] cycloaddition reactions,^[12]^ a variety of other MCp^x^ complexes have successfully been developed by You et al.,^[13]^ Cramer et al.,^[14]^ Li et al.^[15]^ and us[^11d^, ^16^] for the asymmetric synthesis of different atropisomers.

**Scheme 1:**
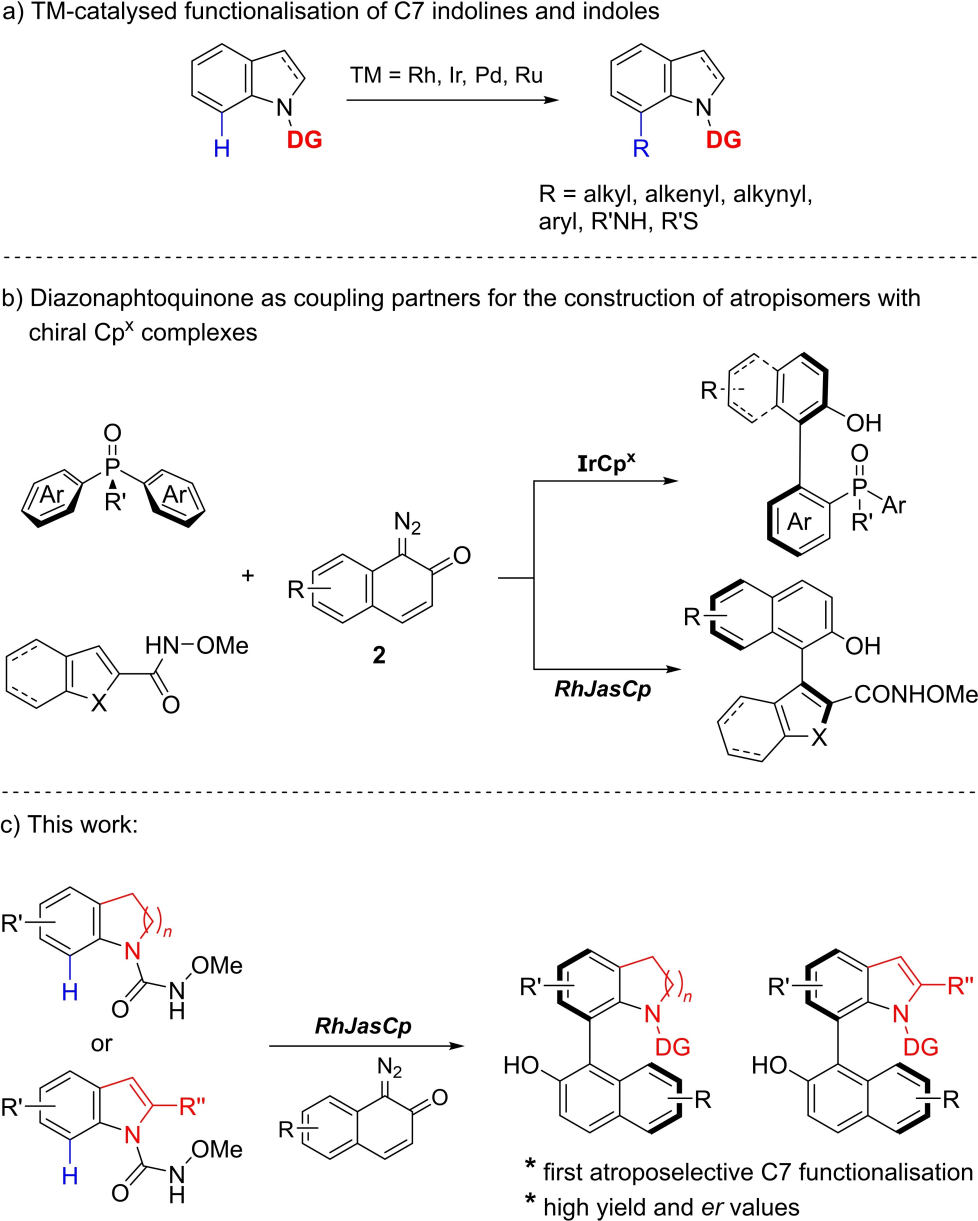
a) General C7 functionalisation reactions with transition metals (TM). b) Previous reports on the synthesis of atropisomers by means of chiral MCp^x^ complexes and diazonaphthoquinones. c) This work: first atroposelective C7‐indoline and ‐indole functionalisation.

Diazonaphthoquinones (**2**) have proven to be advantageous coupling partners in several C−H functionalisation reactions, including the synthesis of atropisomeric biaryls by means of MCp^x^ C−H functionalisation. Thus, Cramer et al reported their use in the Ir‐catalysed enantioselective synthesis of biaryl phosphine oxides (Scheme 1b upper reaction).^[14a]^ We demonstrated their use with our chiral Rh*Jas*Cp complexes and aryl hydroxamates^[11d]^ and more recently used them in the synthesis of more challenging five‐membered ring atropisomers (Scheme 1b lower reaction).^[17]^

Given the versatile reactivity of diazonaphthoquinones in C−H functionalisation reactions, we reasoned that they might serve as efficient coupling partners in transformations with indolines and indoles bearing a directing group on the nitrogen. Such transformations could yield heteroaryl atropisomers at the 7‐position of indolines and indoles. Herein, we report the development of a chiral Rh*Jas*Cp‐catalysed coupling of diazonaphthoquinones with indolines and indoles by C−H bond activation to construct atropisomers (Scheme 1c), at the C7‐position. To the best of our knowledge this is the first enantioselective C7 indoline functionalisation.

In initial experiments the directing group effect of different nitrogen substituents was investigated (Table 1). In contrast to previous reports, 2‐pyrimidine (entry 1), amide (entry 2) and urea (entry 3) directing groups did not lead to the formation of the desired coupling products. However, with a hydroxamate directing group (entry 4), the desired product **3 a** was obtained in excellent yield and with appreciable enantiomer ratio (*er*). This finding indicates the importance of increased electronegativity on the nitrogen atom of the directing group to facilitate the coordination with the Rh catalyst.^[18]^ Investigation of different *Jas*Cp catalysts which we had successfully employed in C−H activation reactions before (entries 5–7) did not lead to a significant increase in yield, and catalyst **Rh1** differentiated best between the possible atropisomers. To our surprise, cat **Rh2** and **Rh3** which gave the best results in similar transformations[^11d^, ^17^] led to low enantiomer ratios in this transformation. Solvent screening revealed that 1,4‐dioxane is most advantageous with respect to both yield and stereoselectivity (entries 8–10). Finally, varied substrate concentrations (entries 11–13) and external oxidants (entries 14–15) were tested and conditions were identified under which the desired product was obtained in excellent yield and with high *er* value (entry 11).

**Table 1:** Reaction optimisation for the enantioselective synthesis of six‐membered‐ring atropoisomeric indoline **3 a**.^[a]^

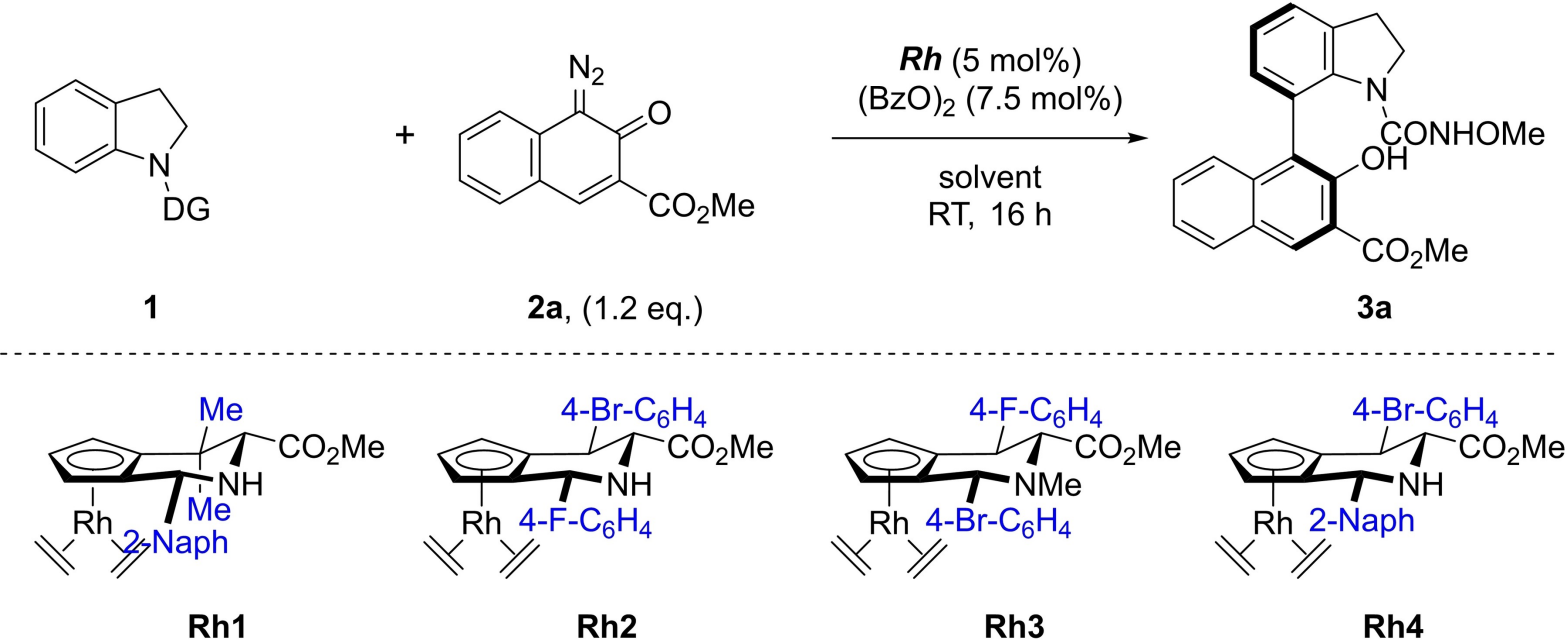						
	DG	Cat.	Solvent	Conc [M]	Yield [%]	er
1	Pym	**Rh1**	THF	[0.25]	0	n.d.
2	COMe	**Rh1**	THF	[0.25]	0	n.d.
3	CONHPr	**Rh1**	THF	[0.25]	0	n.d.
4	CONHOMe	**Rh1**	THF	[0.25]	90	84 : 16
5	CONHOMe	**Rh2**	THF	[0.25]	88	56 : 44
6	CONHOMe	**Rh3**	THF	[0.25]	87	60 : 40
7	CONHOMe	**Rh4**	THF	[0.25]	83	80 : 20
8	CONHOMe	**Rh1**	benzene	[0.25]	66	78 : 22
9	CONHOMe	**Rh1**	CH_2_Cl_2_	[0.25]	58	81 : 19
10	CONHOMe	**Rh1**	1,4‐dioxane	[0.25]	90	89 : 11
11	CONHOMe	**Rh1**	1,4‐dioxane	[0.4]	91	92 : 8
12	CONHOMe	**Rh1**	1,4‐dioxane	[0.5]	89	90 : 10
13	CONHOMe	**Rh1**	1,4‐dioxane	[1.0]	–	n.d.
14^[b]^	CONHOMe	**Rh1**	1,4‐dioxane	[0.4]	20	n.d.
15^[c]^	CONHOMe	**Rh1**	1,4‐dioxane	[0.4]	–	n.d.

[a] Reactions were run for 16 h at RT. Yields were determined for isolated products. DG: Directing group. *er*: enantiomer ratio, determined using chiral HPLC. Pym: 2‐pyrimidine. n.d.: not detected. [b] Cu(OAc)_2_ (10 mol %) was used as an oxidant. [c] Cu(OTf)_2_ (10 mol %) was used as an oxidant.

With these reaction conditions determined, we explored the substrate scope for this transformation. As shown in Scheme 2, a variety of diazonaphthoquinones (**2**) yielded the desired products in very good yields and with high *er* values (**3 a**–**d**), and different functional groups on the indoline are also tolerated under the reaction conditions (**3 e**–**j**). Particularly appealing is the finding that bromine is well tolerated (**3 c**, **3 e**, **3 f**) which allows for further modification using a variety of cross coupling reactions. We hypothesised that in the presence of a substituent in the 6‐position of the indoline, the enantiomer ratio might be improved, as the presence of such a substituent should increase the rotation barrier. 6‐Cl‐ and 6‐Me‐indolines did not give the desired product, which is in accordance with previous reports stressing steric hindrance as reason for this lack of reactivity.^[19]^ However, in the presence of a smaller atom (6‐F) the desired product was formed, albeit in slightly lower yield, and with moderate *er* value (**3 k**). Investigation of different nitrogen heterocycles revealed that a piperidine moiety was not tolerated under the reaction conditions (**3 l**). In contrast, in the presence of a morpholine derivative the transformation proceeded smoothly and products **3 m** and **3 n** were obtained in excellent yields and with appreciable enantioselectivity. In addition, C2‐ and C3‐substituted indolines afforded the desired products **3 o** and **3 p** with excellent yield and good *ee* values.

**Scheme 2:**
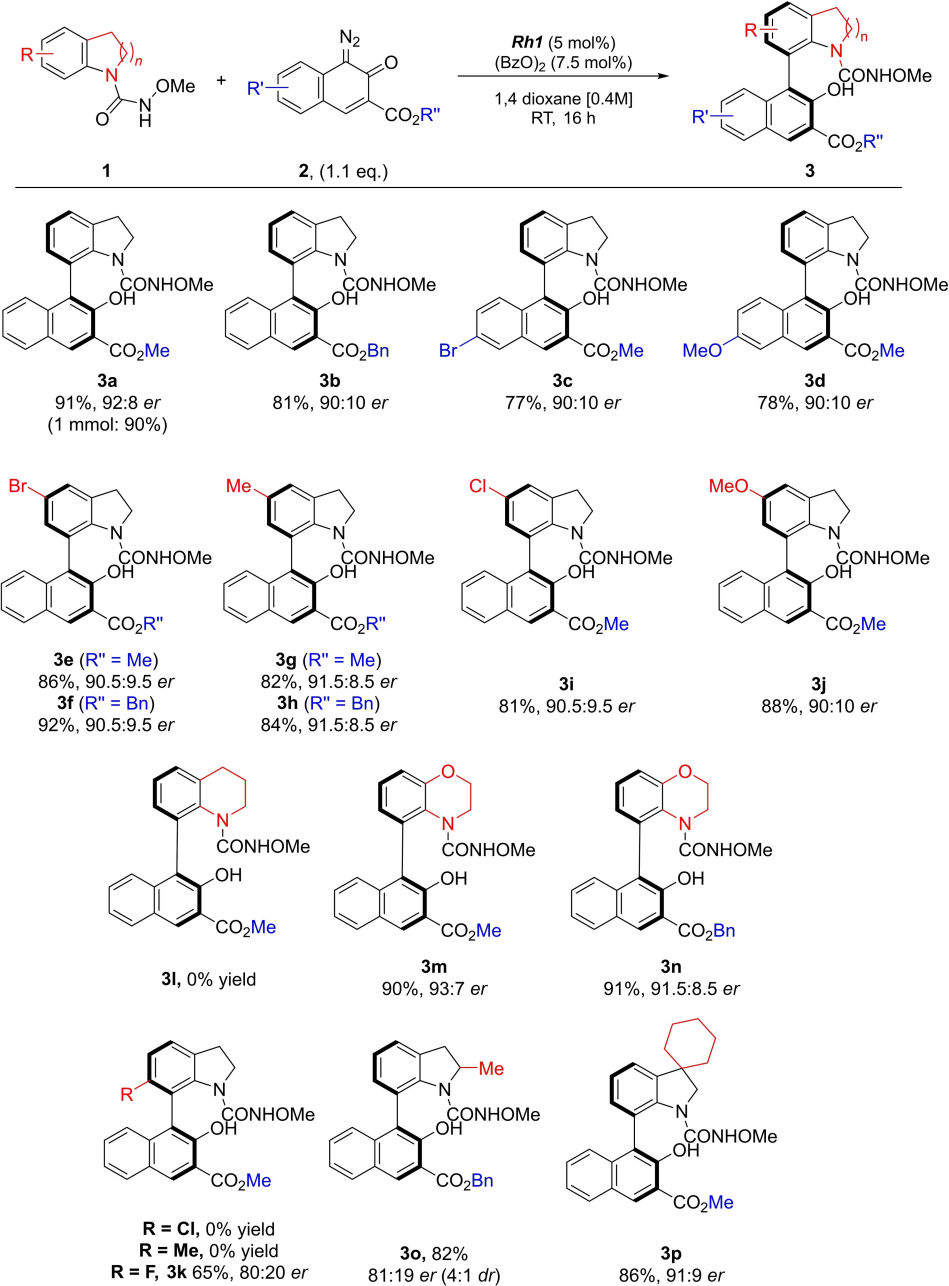
Exploration of the scope of the enantioselective synthesis of C7‐substituted atropoisomeric indolines and analogues.

C7 indoline functionalisation is a reliable method for the synthesis of C7‐substituted indoles through an additional oxidation step. Based on our previous findings, we expected that indole hydroxamates would react exclusively at the C‐2 position.^[17]^ In fact, for indole hydroxamate the exclusive functionalisation of the heterocycle at C‐2 (**4 a**) was observed (Scheme 3). This product however racemises quickly. When the C‐2 position of the indole was blocked by introduction of a substituent, the reaction yielded the C7‐functionalised indoles with good yield and *er* (**4 b**). This reaction is of high importance as efficient methodology for the regio‐ and enantioselective functionalisation of indoles at C‐7 is only available for isolated cases.^[9]^

**Scheme 3:**
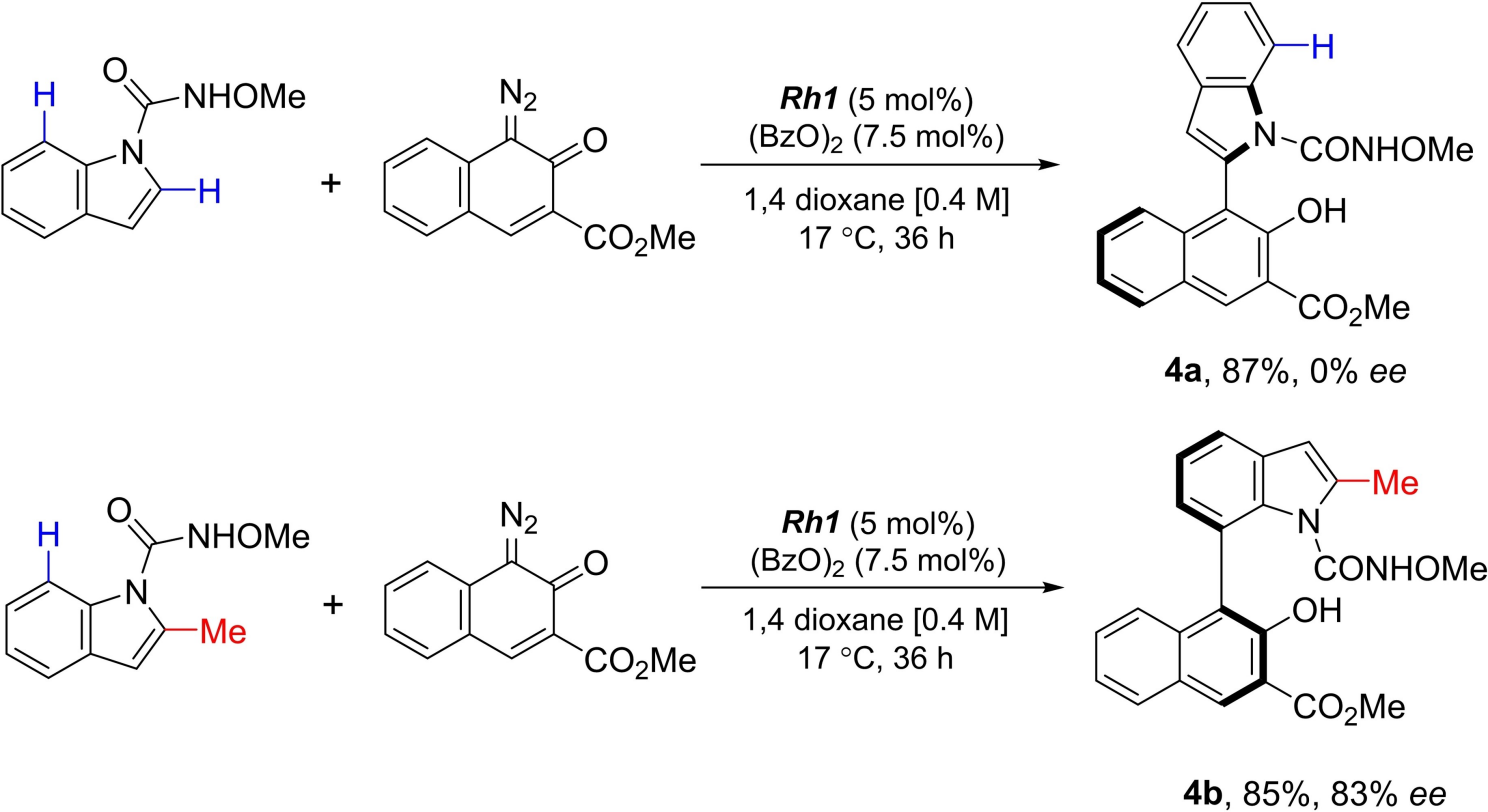
C2‐ vs. C7‐indole functionalisation.

Exploration of reaction scope revealed that, gratifyingly, different 2‐substituted indoles bearing electron‐donating and ‐withdrawing groups yielded the desired atropisomers in very good yields and with high *er* values (**4 b**–**j**; Scheme 4).

**Scheme 4:**
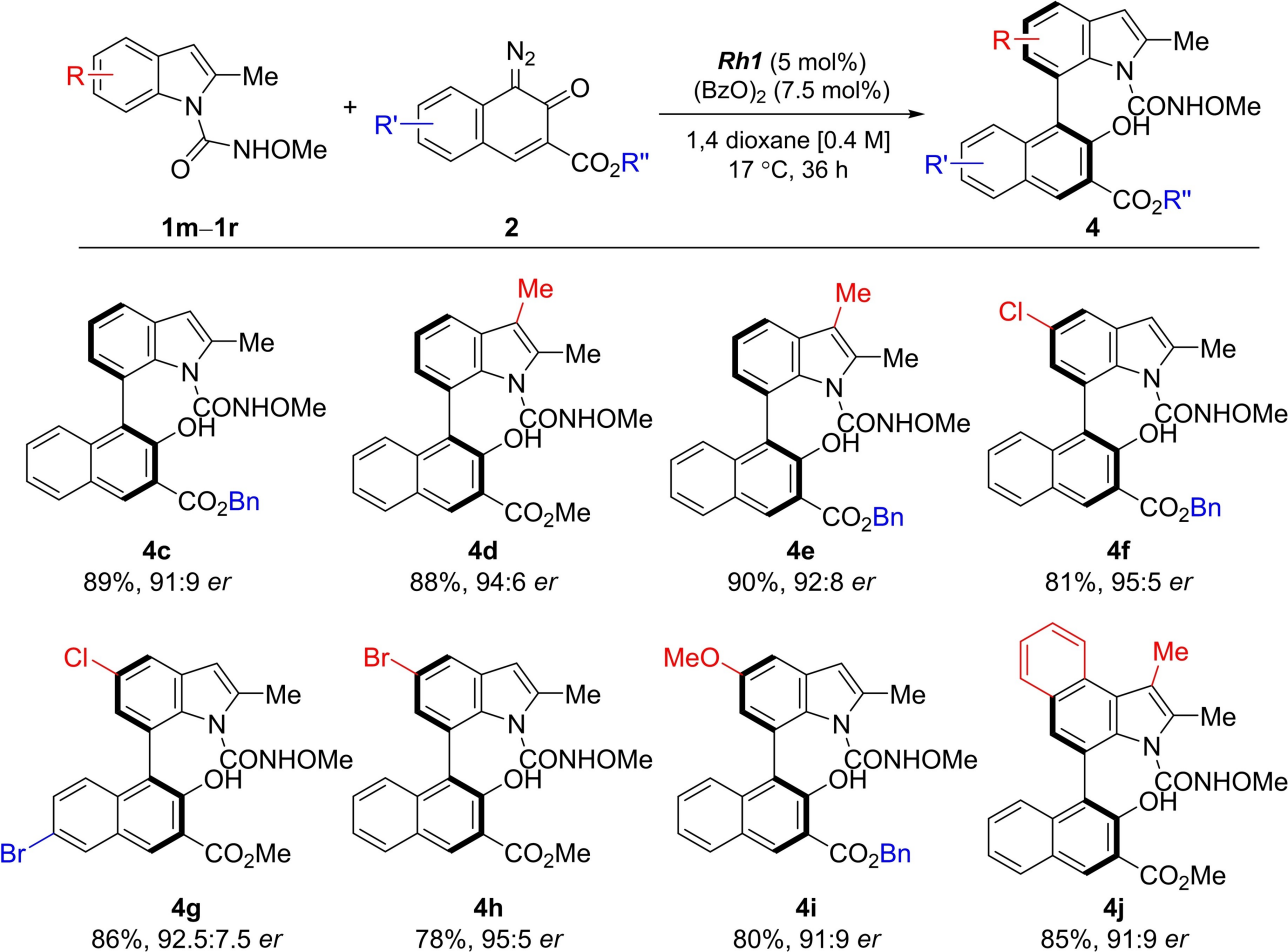
Exploration of the scope for the synthesis of atropisomeric C7‐substituted indoles.

The absolute configuration of compound **3 a** was determined to be (a*R*) by means of vibrational circular dichroism (VCD) spectroscopy.^[20]^ A comparison of the obtained final spectra with the experimental IR and VCD spectra is shown in Figure 1. The visual comparison of the predicted VCD pattern of *R* isomer reveals an exceptionally good agreement with the experimental spectrum of **3 a** as all characteristic bands are found well reproduced. Hence, based on the VCD spectra, the configuration of **3 a** is confirmed as a*R*. (see the Supporting Information for more details).

**Figure 1:**
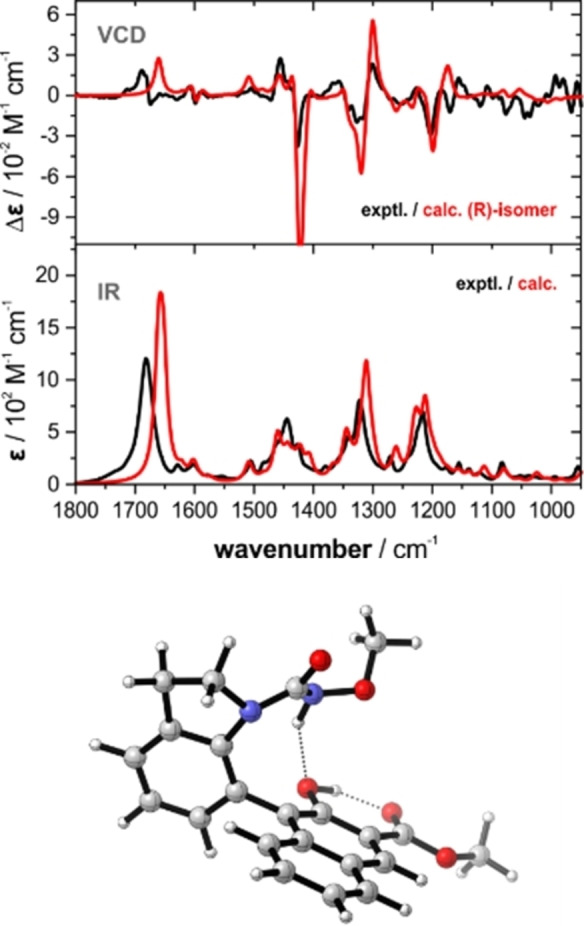
Top: Comparison of experimental and computed IR and VCD spectra of **3 a** and the *R* isomer (6 cm^−1^ HWHH, *σ*=0.98). Bottom: Structure of the lowest‐energy conformer, C1.

As a plausible mechanism for this transformation we propose that the reaction begins with an oxidative addition of the active Rh^III^ complex **I** to give the six‐membered‐ring rhodacyle **II**. Insertion of the diazonaphthoquinone **2 a** affords intermediate **III**, which upon loss of nitrogen (N_2_) yields the Rh‐carbene intermediate **IV**. This intermediate undergoes 1,2‐migration furnishing intermediate **V**. A subsequent reductive elimination followed by aromatisation (point to axis chirality transfer) yields the final biaryl atropisomer **3** (Scheme 5). This proposal is in agreement with earlier related findings.[^17^, ^21^]

**Scheme 5:**
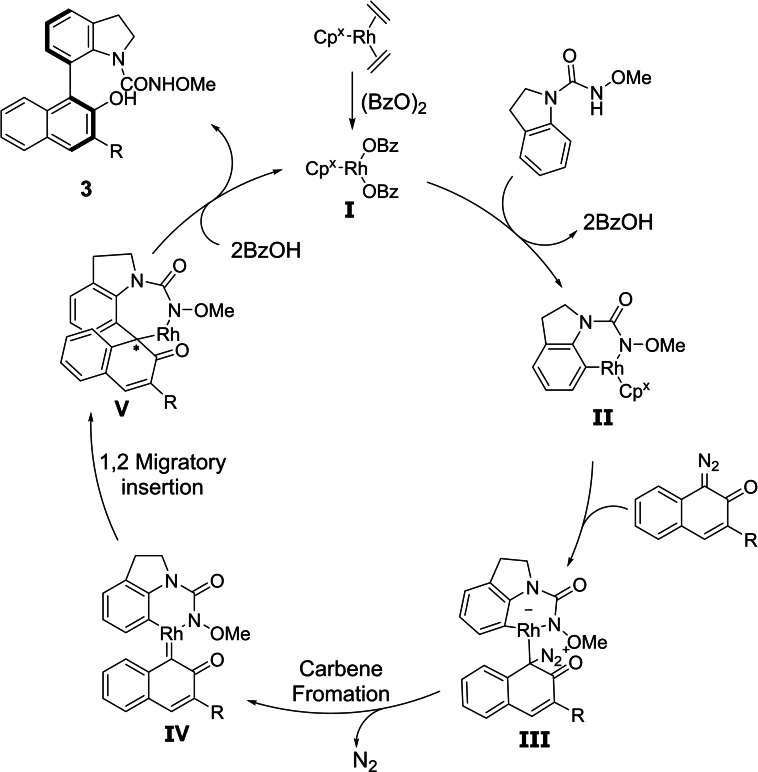
Proposed reaction mechanism.

In conclusion, we have developed an enantioselective C−H functionalisation method that gives access to C7‐indolino‐ and C7‐indolo‐biaryl atropisomers under mild conditions. The method enables the synthesis of these six‐membered‐ring atropisomers in high yield and with high enantioselectivity. In light of the challenging synthesis of C7‐indolino and ‐indolo atropisomers, we anticipate that this practical, enantioselective direct C−H functionalisation methodology could find widespread application.

## Conflict of interest

The authors declare no conflict of interest.

## Supporting information

As a service to our authors and readers, this journal provides supporting information supplied by the authors. Such materials are peer reviewed and may be re‐organized for online delivery, but are not copy‐edited or typeset. Technical support issues arising from supporting information (other than missing files) should be addressed to the authors.

Supporting Information
