# Supplementary material for: Catalytic Atroposelective C7 Functionalisation of Indolines and Indoles
Source: Chemistry. 2021 Nov 5;28(1):e202103365. doi: 10.1002/chem.202103365 (PMC9298066; doi:10.1002/chem.202103365)
Supplement: Supplementary file 1 — Supporting Information [file CHEM-28-0-s001.pdf]

Chemistry–A European Journal

Supporting Information

Catalytic Atroposelective C7 Functionalisation of Indolines and Indoles

Saad Shaaban, Christian Merten, and Herbert Waldmann*

Table of Contents

General Information: 3

General procedure for the synthesis of *N*-methoxyindoline-1-carboxamides 1 4

General procedure for the synthesis of *N*-indolo-benzamides 1........ 5

General procedure for the synthesis of 6-memeberd ring atropisomers 3 and 4........ 8

Determination of the absolute configuration of 3a by VCD spectroscopy........ 22

NMR and HPLC Spectra:........ 26

References:........ 87

General Information:

Unless otherwise noted, all commercially available compounds were used as provided without further purification. Solvents for chromatography were technical grade.

Analytical thin-layer chromatography (TLC) was performed on Merck silica gel aluminium plates with F-254 indicator. Compounds were visualized by irradiation with UV light or potassium permanganate staining. Column chromatography was performed using silica gel Merck 60 (particle size 0.040-0.063 mm).

^{1}H-NMR, ^{13}C-NMR and ^{19}F-NMR were recorded on a Bruker DRX400 (400 MHz), Bruker DRX500 (500 MHz), INOVA500 (500 MHz) and Bruker DRX700 using $CDCl_3$ as solvent. Data are reported in the following order: chemical shift (δ) values are reported in ppm with the solvent resonance as internal standard ($CDCl_3$: δ = 7.26 ppm for ^{1}H, δ = 77.16 ppm for ^{13}C). Multiplicities are indicated br s (broad singlet), s (singlet), d (doublet), t (triplet), q (quartet), m (multiplet); coupling constants (J) are given in Hertz (Hz).

High resolution mass spectra (HR-MS) were recorded on an LTQ Orbitrap mass spectrometer coupled to an Accela HPLC-System (HPLC column: Hypersyl GOLD, 50 mm x 1 mm, particle size 1.9 µm, ionization method: electron spray ionization).

The enantiomeric excesses were determined by HPLC analysis using a chiral stationary phase column (CHIRALCEL IC, CHIRALCEL IA; eluent: (CH_2Cl_2/EtOH = 100/2) / *iso*-hexane, *i*-Propanol / iso-hexane; 4.6 mm x 250 mm, particle size 5 µm). The chiral HPLC methods were calibrated with the corresponding racemic mixtures.

Diazonaphthoquinones and Rh-catalysts were prepared according to the previously reported procedure and their characterization match the one reported. [1]

General procedure for the synthesis of *N*-methoxyindoline-1-carboxamides 1

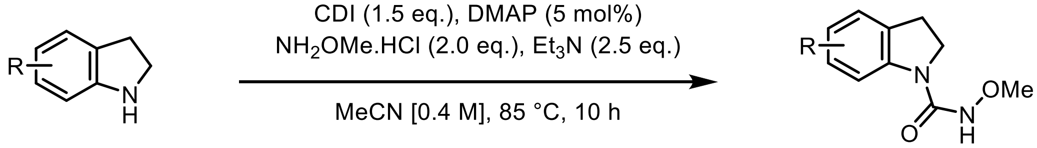

According to the modified procedure of Li *et al.* [2] A 100 mL round bottle charged with stirring bar, was added indoline (1.0 equiv.), 1,1'-carbonyldiimidazole (CDI, 1.5 equiv.) and 4-dimethylaminepyridine (DMAP, 5 mol%). Then anhydrous acetonitrile [0.4 M] was added. The mixture was stirred for 5 mins till it became homogenous. $MeONH_2$.HCl (2.0 equiv.) and Et_3N (2.5 eq.) were added and the mixture was refluxed at 85 °C for 10 h. After cooling down to room temperature, the solvents were removed under reduced pressure. To the residue, Water was added (20 ml) and the mixture was extracted 3 times with EtOAc (20 ml). The organic layers were combined, washed with brine (10 ml), dried over Na_2SO_4 and then concentrated under reduced pressure. The residue was purified by silica chromatography suing Pent : EtOAc (4:1 to 2:1) as eluent to afford the corresponding *N*-methoxyindoline-1-carboxamides **1** and their characterization match the one reported. [2]

6-fluoro-*N*-methoxyindoline-1-carboxamide (1f)

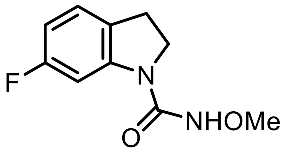

Off-white solid, 68% yield. **^{1}H-NMR (500 MHz - $CDCl_3$)**: δ 7.61 (dd, J = 10.5, 2.5 Hz, 1H), 7.37 (s, 1H), 6.99 (ddt, J = 8.1, 5.5, 1.2 Hz, 1H), 6.57 (ddd, J = 8.9, 8.2, 2.5 Hz, 1H), 3.84 (dd, J = 9.1, 8.1 Hz, 2H), 3.74 (s, 3H), 3.12 – 3.03 (m, 2H); **^{13}C-NMR (126 MHz - $CDCl_3$)**: 163.5, 155.9, 144.1, 125.3, 124.9, 109.1, 103.7, 64.7, 47.2, 27.4; **^{19}F-NMR (470 MHz - $CDCl_3$)**: δ -114.22 (td, J = 10.6, 10.0, 5.8 Hz); **HR-MS**: calc. for $[M+H]^+$ $C_{10}H_{12}O_2N_2F$ = 211.0883 found: 211.0880.

***N*-methoxy-2,3-dihydro-4H-benzo[b][1,4]oxazine-4-carboxamide (1j)**

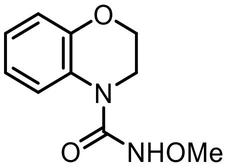

Brown solid, 61% yield. **^{1}H-NMR (500 MHz - $CDCl_3$)**: δ 7.87 (s, 1H), 7.27 (dd, J = 8.0, 1.6 Hz, 1H), 7.00 (ddd, J = 8.2, 7.2, 1.6 Hz, 1H), 6.89 – 6.79 (m, 2H), 4.23 – 4.18 (m, 2H), 3.85 – 3.73 (m, 2H), 3.73 (s, 3H); **^{13}C-NMR (126 MHz - $CDCl_3$)**: 156.5, 146.9, 126.3, 124.9, 122.9,

120.6, 118.0, 66.3, 64.7, 40.7; **HR-MS**: calc. for $[M+H]^+$ $C_{10}H_{13}O_3N_2$ = 209.0926 found: 209.0924. **FT-IR:** ṽ = 3227, 2956, 1650, 1585, 1455, 1322, 1231, 1120, 1054, 937, 835 cm^{-1}.

***N*-methoxyspiro[cyclohexane-1,3'-indoline]-1'-carboxamide (1k)**

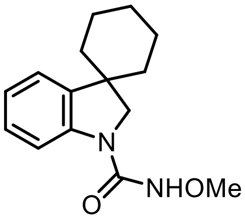

Orange solid, 61% yield. **^{1}H-NMR (400 MHz - $CDCl_3$)**: δ 7.77 (t, d = 8.5 Hz, 1H), 7.60 (s, 1H), 7.16 – 7.06 (m, 1H), 7.05 (dd, J = 7.6, 1.3 Hz, 1H), 7.02 – 6.89 (m, 1H), 3.76 (s, 3H), 3.63 (s, 2H), 1.63 (s, 2H), 1.57 – 1.42 (m, 6H), 1.34 – 1.18 (m, 2H); **^{13}C-NMR (101 MHz - $CDCl_3$)**: 141.5, 139.8, 127.9, 122.9, 122.5, 115.1, 64.6, 56.9, 44.8, 37.3, 25.3, 23.0; **HR-MS**: calc. for $[M+H]^+$ $C_{15}H_{21}O_2N_2$ = 261.1603 found: 261.1602; **FT-IR:** ṽ = 3228, 2928, 1651, 1601, 1496, 1349, 1264, 1099, 1058, 937, 893 cm^{-1}.

General procedure for the synthesis of *N*-indolo-benzamides 1

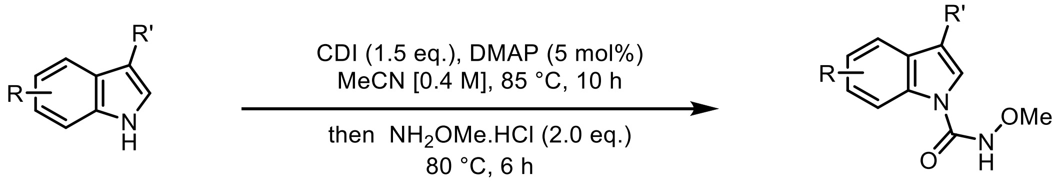

According to the reported procedure, [2b] A 100 mL round bottle charged with stirring bar, was added indole (1g, 1.0 equiv.), 1,1'-carbonyldiimidazole (CDI, 1.5 equiv.) and 4-dimethylaminepyridine (DMAP, 5 mol%). Then anhydrous acetonitrile [0.4 M] was added. The mixture was refluxed at 85 °C for 10 h. After cooling down to room temperature, $MeONH_2$.HCl (2.0 equiv.) and Et_3N (2.2 equiv.) were added and the mixture was stirred at 80 °C for another 6 h. The mixture was cooled down to room temperature and then the solvents were removed under reduced pressure. The crude was dissolved in H_2O (20 ml), extracted (3x15 ml EA), the organic layers were dried over Na_2SO_4 and concentrated under reduced pressure. The residue was purified by silica chromatography to afford the corresponding *N*-methoxy-*1H*-indole-1-carboxamides **1**.

***N*-methoxy-2-methyl-*1H*-indole-1-carboxamide (1m)**

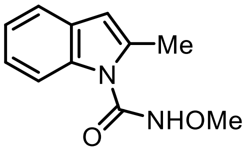

Off-white solid, 69% yield. **^{1}H-NMR (500 MHz - CDCl$_3$)**: δ 8.31 (s, 1H), 7.71 (dq, J = 8.3, 1.0 Hz, 1H), 7.48 (dt, J = 7.5, 0.9 Hz, 1H), 7.27 – 7.15 (m, 2H), 6.35 (s, 1H), 3.95 (s, 3H), 2.58 (s, 3H); **^{13}C-NMR (126 MHz - CDCl$_3$)**: 152.8, 136.9, 135.2, 129.4, 122.9, 122.3, 120.2, 112.4, 106.9, 64.9, 15.1; **HR-MS**: calc. for $[M+H]^+$ $C_{11}H_{13}O_2N_2$ = 205.0977 found: 205.0972; **FT-IR:** ṽ = 3223, 2929, 1652, 1601, 1445, 1321, 1264, 1212, 1149, 1060, 957, 893 cm^{-1}.

***N*-methoxy-2,3-dimethyl-1H-indole-1-carboxamide (1n)**

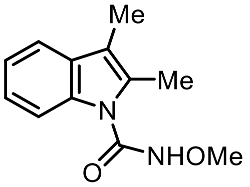

Yellow solid, 34% yield. **^{1}H-NMR (500 MHz - CDCl$_3$)**: δ 8.18 (s, 1H), 7.70 (d, J = 8.3 Hz, 1H), 7.44 (d, J = 8.5 Hz, 1H), 7.26 – 7.17 (m, 2H), 3.95 (s, 4H), 2.50 (s, 3H), 2.19 (s, 3H); **^{13}C-NMR (126 MHz - CDCl$_3$)**: 153.3, 134.7, 132.3, 130.8, 123.2, 122.2, 118.7, 113.5, 112.5, 65.1, 12.5, 8.7; **HR-MS**: calc. for $[M+H]^+$ $C_{12}H_{15}O_2N_2$ = 219.1134 found: 219.1128; **FT-IR:** ṽ = 3225, 2928, 1651, 1585, 1381, 1204, 1160, 1011, 957, 873 cm^{-1}.

5-chloro-*N*-methoxy-2-methyl-1H-indole-1-carboxamide (1o)

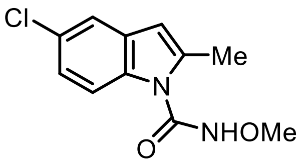

Brown solid, 61% yield. **^{1}H-NMR (500 MHz - CDCl$_3$)**: δ 8.25 (s, 1H), 7.54 (d, J = 8.8 Hz, 1H), 7.34 (d, J = 2.1 Hz, 1H), 7.07 (dd, J = 8.7, 2.1 Hz, 1H), 6.20 (s, 1H), 3.85 (s, 3H), 2.47 (s, 3H); **^{13}C-NMR (126 MHz - CDCl$_3$)**: 152.5, 138.1, 133.8, 130.5, 127.9, 122.9, 119.6, 113.6, 106.4, 64.9, 15.1; **HR-MS**: calc. for $[M+H]^+$ $C_{11}H_{12}O_2N_2Cl$ = 239.0587 found: 239.0585; **FT-IR:** ṽ = 3223, 2929, 1652, 1558, 1445, 1301, 1211, 1049, 1011, 987, 873 cm^{-1}.

5-bromo-*N*-methoxy-2-methyl-1H-indole-1-carboxamide (1p)

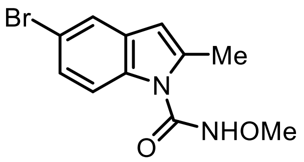

Pink solid, 53% yield. **^{1}H-NMR (500 MHz - CDCl$_3$)**: δ 8.29 (s, 1H), 7.49 (d, J = 8.8 Hz, 2H), 7.21 – 7.18 (m, 1H), 6.19 (s, 1H), 3.84 (s, 3H), 2.46 (s, 3H); **^{13}C-NMR (126 MHz - CDCl$_3$)**: 152.5, 138.0, 134.1, 132.0, 131.0, 125.6, 122.9, 122.7, 122.1, 121.2, 115.5, 114.0, 106.2, 64.9, 15.1; **HR-MS**: calc. for $[M+H]^+$ $C_{11}H_{12}O_2N_2Br$ = 283.0082 and 285.0062 found: 283.0076 and 285.0055; **FT-IR:** ṽ = 2934, 1679, 1441, 1340, 1316, 1215, 1154, 1072, 953, 859 cm^{-1}.

5-methoxy-*N*-methoxy-2-methyl-1H-indole-1-carboxamide (1q)

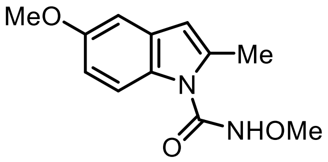

Yellow solid, 66% yield. **^{1}H-NMR (600 MHz - CDCl$_3$)**: δ 8.44 (s, 1H), 7.55 (dt, J = 9.0, 0.6 Hz, 1H), 6.88 (d, J = 2.5 Hz, 1H), 6.76 (dd, J = 9.0, 2.6 Hz, 1H), 6.21 (s, 1H), 3.99 (s, 2H), 3.87 (s, 3H), 3.79 (s, 3H), 2.50 (s, 3H). **^{13}C-NMR (150 MHz - CDCl$_3$)**: 155.7, 153.2, 144.1, 137.8, 130.4, 130.2, 113.6, 111.6, 107.0, 102.8, 65.5, 64.8, 55.8, 15.4; **HR-MS**: calc. for $[M+H]^+$ $C_{12}H_{15}O_3N_2$ = 235.1083 found: 235.1079; **FT-IR:** ṽ = 3129, 1682, 1588, 1473, 1356, 1204, 1175, 1103, 1045, 929, 858 cm^{-1}.

***N*-methoxy-1,2-dimethyl-*3H*-benzo*[e]*indole-3-carboxamide (1r)**

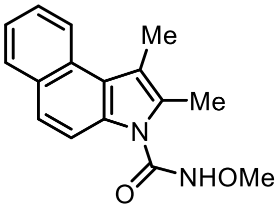

Off-white solid, 58% yield. **^{1}H-NMR (500 MHz - CDCl$_3$)**: δ 8.41 (d, J = 8.4 Hz, 1H), 8.35 (d, J = 4.2 Hz, 1H), 7.89 – 7.78 (m, 1H), 7.75 (dd, J = 9.0, 1.0 Hz, 1H), 7.52 (d, J = 4.2 Hz, 1H), 7.47 (ddd, J = 8.4, 6.9, 1.4 Hz, 1H), 7.37 (ddd, J = 8.1, 6.9, 1.2 Hz, 1H), 3.90 (s, 3H), 2.52 (s, 3H), 2.45 (s, 3H); **^{13}C-NMR (126 MHz - CDCl$_3$)**: 152.3, 131.3, 130.4, 130.2, 128.7, 128.5, 125.9, 123.8, 123.6, 123.5, 123.4, 114.8, 112.7, 64.9, 12.7, 11.6; **HR-MS**: calc. for $[M+H]^+$ $C_{16}H_{17}O_2N_2$ = 269.1290 found: 269.1287; **FT-IR:** ṽ = 3223, 2929, 1652, 1601, 1445, 1321, 1264, 1212, 1149, 1060, 957, 893 cm^{-1}.

General procedure for the synthesis of 6-memeberd ring atropisomers 3 and 4

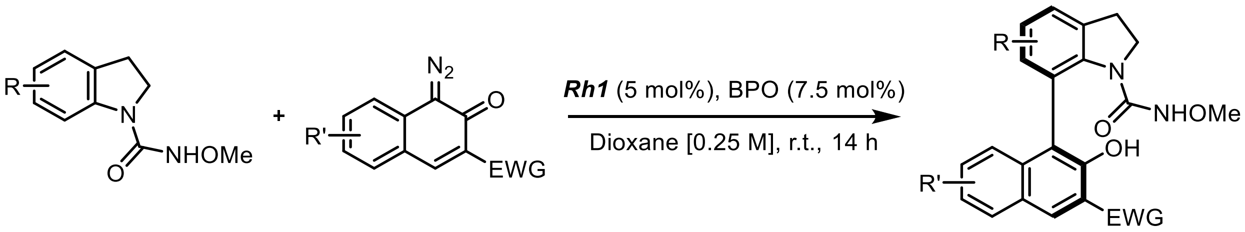

Without protection from oxygen or moisture, the corresponding arylamide **1** (1.0 equiv., 0.05 mmol), **Rh1** (5 mol %, 2.5 µmol, 1.5 mg), benzoyl peroxide (BPO) (7.5 mol%, 3.75 µmol, 1.2 mg) and diazonaphthoquinone **2** (1.2 equiv., 0.06 mmol) were dissolved in dry 1,4-dioxane [0.4 M]. The mixture was stirred at room temperature for 14 h. Then, the solvent was removed under reduced pressure and the crude was directly purified by silica gel chromatography using Pent: EtOAc (3:1 to 1:1) as an eluent to afford the desired products **3** and **4**.

Procedure for the synthesis of ''3a'' (1.0 mmol)

Without protection from oxygen or moisture, the arylamide **1a** (1.0 equiv., 1.0 mmol, 192 mg), **Rh1** (5 mol %, 0.05 mmol, 30 mg), benzoyl peroxide (BPO) (7.5 mol%, 0.07 mmol, 24 mg) and diazonaphthoquinone **2a** (1.2 equiv., 1.2 mmol, 273 mg) were dissolved in 4 ml dry 1,4-dioxane [0.4 M]. The mixture was stirred at room temperature for 14 h. Then, the solvent was removed under reduced pressure and the crude was directly purified by silica gel chromatography using Pent: EtOAc (3:1 to 1:1) as an eluent to afford **3a** in 90% yield (352 mg).

Methyl (*R*)-3-hydroxy-4-(1-(methoxycarbamoyl)indolin-7-yl)-2-naphthoate (3a)

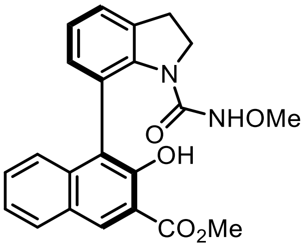

Yellow oil, 17.8 mg, 91%. **^{1}H-NMR (500 MHz - $CDCl_3$)**: 10.89 (s, 1H), 8.49 (s, 1H), 7.74 (d, J = 6.7 Hz, 1H), 7.66 (s, 1H), 7.36 (t, J = 8.3 Hz, 1H), 7.32 – 7.19 (m, 3H), 7.16 (t, J = 8.0 Hz, 1H), 7.11 (dd, J = 7.7, 1.5 Hz, 1H), 4.25 (ddd, J = 11.0, 8.7, 4.3 Hz, 1H), 3.98 (s, 3H), 3.97 – 3.86 (m, 1H), 3.18 – 3.07 (m, 1H), 3.08 (s, 3H), 2.95 (ddd, J = 15.4, 8.6, 4.3 Hz, 1H); **^{13}C-NMR (125 MHz - $CDCl_3$):** 170.5, 156.3, 152.4, 141.7, 135.8, 135.4, 132.8, 131.5, 129.8, 129.7, 127.2, 125.0, 124.9, 124.2, 124.1, 123.5, 121.4, 113.9, 63.7, 52.8, 51.2, 29.6, 26.9; **FT-IR:** ṽ = 2936, 1678, 1599, 1440, 1385, 1304, 1232, 1173, 1085, 1033, 990, 958, 829 cm^{-1}; **HR-MS**: calc. for $[M+H]^+$ $C_{22}H_{21}O_5N_2$ = 393.1450 found: 393.1445; **HPLC conditions:** CHIRAPAK IC column, DCM: EtOH (100:2) / *iso*-hexane = 40/60, flow rate = 0.5 mL min^{-1}, major enantiomer: t_R = 43.5 min; minor enantiomer: t_R = 48.6 min, 84% ee.

Benzyl (*R*)-3-hydroxy-4-(1-(methoxycarbamoyl)indolin-7-yl)-2-naphthoate (3b)

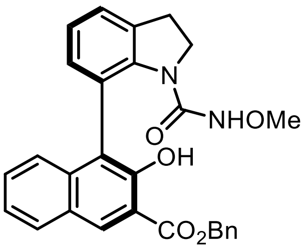

Dark yellow oil, 19 mg, 81%. **^{1}H-NMR (500 MHz - $CDCl_3$)**: 10.89 (s, 1H), 8.51 (s, 1H), 7.75 (d, J = 8.1 Hz, 1H), 7.65 (s, 1H), 7.49 – 7.40 (m, 2H), 7.40 – 7.30 (m, 4H), 7.33 – 7.23 (m, 2H), 7.22 (t, J = 8.8 Hz, 1H), 7.15 (t, J = 7.5 Hz, 1H), 7.10 (dd, J = 7.8, 1.5 Hz, 1H), 5.41 (s, 2H), 4.25 (ddd, J = 11.0, 8.7, 4.3 Hz, 1H), 3.94 (dt, J = 11.1, 8.8 Hz, 1H), 3.13 (dt, J = 16.5, 8.8 Hz, 1H), 3.05 (s, 3H), 2.95 (ddd, J = 15.0, 8.3, 4.2 Hz, 1H); **^{13}C-NMR (125 MHz - $CDCl_3$):** 175.8, 169.9, 164.6, 156.1, 152.5, 145.4, 141.7, 135.8, 132.8, 131.8, 130.3, 129.9, 128.7, 127.7, 126.1, 125.0, 124.9, 123.5, 122.7, 121.4, 119.3, 115.2, 113.9, 64.6, 51.2, 46.4, 29.6, 28.1; **HR-MS**: calc. for $[M+H]^+$ $C_{28}H_{25}O_5N_2$ = 469.1763 found: 469.1765; **FT-IR:** ṽ = 3223, 2929, 1652, 1601, 1445, 1321, 1264, 1212, 1149, 1060, 957, 893 cm^{-1}; **HPLC conditions:** CHIRAPAK IC column, DCM: EtOH (100:2) / *iso*-hexane = 40/60, flow rate = 0.5 mL min^{-1}, major enantiomer: t_R = 41.6 min; minor enantiomer: t_R = 39.0 min, 80% ee.

Methyl (*R*)-7-bromo-3-hydroxy-4-(1-(methoxycarbamoyl)indolin-7-yl)-2-naphthoate (3c)

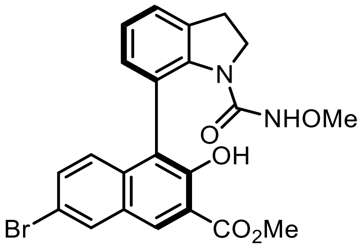

Red solid, 18 mg, 77%. **^{1}H-NMR (500 MHz - $CDCl_3$)**: 10.89 (s, 1H), 8.38 (s, 1H), 7.91 (d, J = 2.0 Hz, 1H), 7.45 (s, 1H), 7.39 (dd, J = 9.1, 2.0 Hz, 1H), 7.28 (dd, J = 7.3, 1.2 Hz, 1H), 7.19 – 7.05 (m, 4H), 4.22 (ddd, J = 10.9, 8.8, 4.7 Hz, 1H), 3.98 (s, 3H), 3.92 (dt, J = 10.9, 8.7 Hz, 1H), 3.15 (s, 3H), 3.18 – 3.08 (m, 1H), 3.03 – 2.93 (m, 1H); **^{13}C-NMR (125 MHz - $CDCl_3$):** 170.3, 156.4, 141.7, 135.5, 133.9, 132.6, 131.4, 131.4, 131.3, 128.2, 126.2, 125.0, 124.9, 123.0, 122.2, 117.8, 114.7, 63.8, 52.9, 52.7, 29.6; **FT-IR:** ṽ = 2936, 1678, 1599, 1493, 1386, 1305, 1232, 1173, 1085, 990, 829 cm^{-1}. **HR-MS**: calc. for $[M+H]^+$ $C_{22}H_{20}O_5N_2Br$ = 471.0556 and 473.0535 found: 471.0556 and 471.0535; **HPLC conditions:** CHIRAPAK IC column, *iso*-Propanol / *iso*-hexane = 40/60, flow rate = 0.5 mL min^{-1}, major enantiomer: t_R = 55.3 min; minor enantiomer: t_R = 66.7 min, 80% ee.

Methyl (*R*)-7-methoxy-3-hydroxy-4-(1-(methoxycarbamoyl)indolin-7-yl)-2-naphthoate (3d)

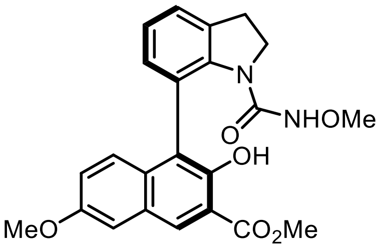

Red solid, 16.5 mg, 78%. **^{1}H-NMR (500 MHz - CDCl$_3$)**: 10.73 (s, 1H), 8.37 (s, 1H), 7.69 (s, 1H), 7.27 (dd, J = 9.1, 2.0 Hz, 1H), 7.15 – 7.06 (m, 4H), 7.05 – 7.01 (m, 2H), 4.28 – 4.23 (m, 1H), 3.96 (s, 3H), 3.95 – 3.89 (m, 1H), 3.81 (s, 3H), 3.12 (s, 3H), 3.15 – 3.10 (m, 1H), 2.96 – 2.90 (m, 1H); **^{13}C-NMR (125 MHz - CDCl$_3$):** 170.5, 156.3, 156.2, 151.0, 141.7, 135.8, 131.4, 131.0, 128.1, 125.7, 125.0, 124.9, 123.6, 122.9, 121.6, 114.1, 106.9, 63.7, 55.3, 52.7, 51.2, 29.5; **HR-MS**: calc. for $[M+H]^+$ $C_{23}H_{23}O_6N_2$ = 423.1556 found: 423.1555; **FT-IR:** ṽ = 3198, 1712, 1670, 1606, 1426, 1342, 1206, 1146, 1076, 949, 904 cm^{-1}; **HPLC conditions:** CHIRAPAK IC column, DCM: EtOH (100:2) / *iso*-hexane = 40/60, flow rate = 0.5 mL min^{-1}, major enantiomer: t_R = 51.1 min; minor enantiomer: t_R = 64.7 min, 80% ee.

Methyl (*R*)-4-(5-bromo-1-(methoxycarbamoyl)indolin-7-yl)-3-hydroxy-2-naphthoate (3e)

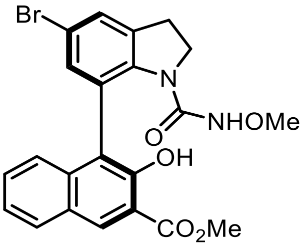

Red oil, 20 mg, 86%. **^{1}H-NMR (500 MHz - CDCl$_3$)**: 10.91 (s, 1H), 8.49 (s, 1H), 7.79 – 7.74 (m, 1H), 7.58 – 7.51 (m, 2H), 7.50 – 7.43 (m, 1H), 7.43 – 7.35 (m, 3H), 7.37 – 7.26 (m, 2H), 7.26 (d, J = 2.1 Hz, 1H), 7.26 – 7.19 (m, 1H), 4.26 – 4.12 (m, 1H), 3.98 (s, 3H), 3.96 (dt, J = 11.0, 8.7 Hz, 1H), 3.20 – 3.09 (m, 1H), 3.07 (s, 3H), 3.01 – 2.89 (m, 1H); **^{13}C-NMR (125 MHz - CDCl$_3$):** 156.0, 152.4, 141.2, 137.8, 137.3, 135.1, 133.9 (2C), 133.1, 130.7, 129.9 (2C), 129.4, 128.8, 127.8, 127.2, 125.1, 124.3, 123.9, 120.2, 117.3, 113.8, 63.7, 52.8, 29.4, 26.5; **HR-MS**: calc. for $[M+H]^+$ $C_{22}H_{20}O_5N_2Br$ = 471.0556 and 473.0535 found: 471.0557 and 471.0538; **FT-IR:** ṽ = 3223, 2929, 1652, 1601, 1445, 1321, 1264, 1212, 1149, 1060, 957, 893 cm^{-1}; **HPLC conditions:** CHIRAPAK IC column, DCM: EtOH (100:2) / *iso*-hexane = 40/60, flow rate = 0.5 mL min^{-1}, major enantiomer: t_R = 46.4 min; minor enantiomer: t_R = 59.5 min, 81% ee.

Benzyl (*R*)-4-(5-bromo-1-(methoxycarbamoyl)indolin-7-yl)-3-hydroxy-2-naphthoate (3f)

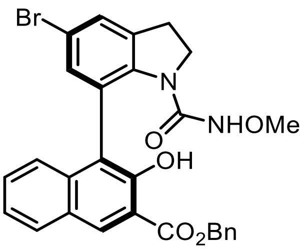

Dark red oil, 25 mg, 92%. **^{1}H-NMR (500 MHz - $CDCl_3$)**: 10.90 (s, 1H), 8.50 (s, 1H), 7.78 – 7.67 (m, 2H), 7.48 (s, 1H), 7.47 – 7.40 (m, 2H), 7.43 – 7.32 (m, 4H), 7.36 – 7.28 (m, 1H), 7.31 – 7.22 (m, 2H), 7.24 – 7.18 (m, 1H), 5.40 (s, 2H), 4.24 – 4.13 (m, 1H), 3.94 (dt, J = 10.9, 8.7 Hz, 1H), 3.18 – 3.07 (m, 1H), 3.05 (s, 3H), 2.96 (ddd, J = 15.6, 8.7, 4.8 Hz, 1H); **^{13}C-NMR (125 MHz - $CDCl_3$):** 169.87, 156.12, 152.55, 141.28, 137.79, 135.19, 134.99, 133.98, 133.19, 130.53, 130.00, 129.98, 128.83, 128.81, 128.56, 127.86, 127.62, 127.19, 125.18, 124.29, 123.92, 120.33, 117.34, 116.70, 113.88, 67.69, 63.74, 51.24, 29.44.; **HR-MS**: calc. for $[M+H]^+$ $C_{28}H_{24}O_5N_2Br$ = 547.0869 and 549.0848 found: 547.0863 and 549.0842; **FT-IR:** $\tilde{v}$ = 2939, 1678, 1599, 1493, 1385, 1232, 1173, 1084, 990, 909 cm^{-1}; **HPLC conditions:** CHIRAPAK IC column, *iso*-Propanol / *iso*-hexane = 40/60, flow rate = 0.5 mL min^{-1}, major enantiomer: t_R = 36.5 min; minor enantiomer: t_R = 44.4 min, 81% ee.

Methyl (*R*)-4-(6-fluoro-1-(methoxycarbamoyl)indolin-7-yl)-3-hydroxy-2-naphthoate (3k)

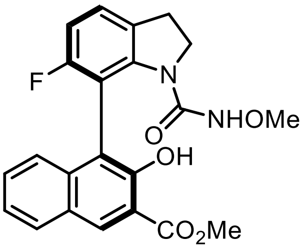

Yellow oil, 9 mg, 45%. **^{1}H-NMR (500 MHz - $CDCl_3$)**: 10.99 (s, 1H), 8.52 (s, 1H), 7.80 – 7.74 (m, 1H), 7.64 (s, 1H), 7.37 (ddd, J = 8.4, 6.8, 1.3 Hz, 1H), 7.32 – 7.20 (m, 2H), 7.16 (dt, J = 8.5, 1.0 Hz, 1H), 6.88 (dd, J = 9.4, 8.1 Hz, 1H), 4.26 (ddd, J = 11.0, 8.8, 4.0 Hz, 1H), 3.98 (s, 3H), 3.98 – 3.91 (m, 1H), 3.10 (s, 3H), 3.18 – 3.00 (m, 1H), 2.95 – 2.86 (m, 1H); **^{13}C-NMR (125 MHz - $CDCl_3$):** 170.4, 161.3, 156.0, 153.4, 135.4, 133.5, 130.7, 129.9, 127.2, 125.5, 125.5, 124.2, 123.7, 114.7, 113.8, 112.6, 112.5, 111.5, 63.7, 52.8, 29.0, 26.9; **^{19}F-NMR (470 MHz - $CDCl_3$)**: δ -114.20; **HR-MS**: calc. for $[M+H]^+$ $C_{22}H_{20}O_5N_2F$ = 411.1356 found: 411.1344; HPLC conditions: CHIRAPAK IC column, DCM: EtOH (100:2) / *iso*-hexane = 40/60, flow rate = 0.5 mL min^{-1}, major enantiomer: t_R = 38.1 min; minor enantiomer: t_R = 42.6 min, 60% ee.

Methyl (*R*)-3-hydroxy-4-(5-methyl-1-(methoxycarbamoyl)indolin-7-yl)-2-naphthoate (3g)

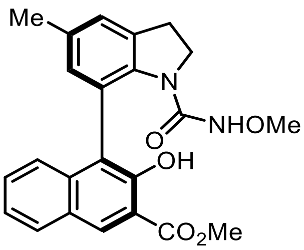

Brown oil, 16.5 mg, 82%. **^{1}H-NMR (500 MHz - $CDCl_3$)**: 10.90 (s, 1H), 8.48 (s, 1H), 7.79 – 7.73 (m, 1H), 7.70 (s, 1H), 7.36 (ddd, J = 8.4, 6.8, 1.3 Hz, 1H), 7.26 (ddd, J = 8.1, 6.8, 1.2 Hz, 1H), 7.22 (dq, J = 8.5, 1.0 Hz, 1H), 7.10 (dd, J = 1.8, 0.9 Hz, 1H), 6.91 (dd, J = 1.8, 1.0 Hz, 1H), 4.25 (ddd, J = 11.0, 8.7, 4.3 Hz, 1H), 3.98 (s, 3H), 3.92 (dt, J = 11.0, 8.7 Hz, 1H), 3.13 – 3.03 (m, 1H), 3.07 (s, 3H), 2.88 (ddd, J = 15.4, 8.4, 4.2 Hz, 1H), 2.30 (s, 3H); **^{13}C-NMR (125 MHz - $CDCl_3$):** 170.5, 156.4, 152.4, 139.4, 136.1, 135.5, 134.8, 132.7, 131.5, 129.8, 129.7, 127.2, 125.9, 124.2, 124.2, 123.2, 121.4, 113.9, 63.6, 52.8, 51.3, 29.5, 21.0; **HR-MS**: calc. for $[M+H]^+$ $C_{23}H_{23}O_5N_2$ = 407.1607 found:407.1601; **FT-IR:** ṽ = 2935, 1678, 1599, 1493, 1386, 1304, 1232, 1172, 1085, 990, 829 cm^{-1}; **HPLC conditions:** CHIRAPAK IC column, DCM: EtOH (100:2) / *iso*-hexane = 40/60, flow rate = 0.5 mL min^{-1}, major enantiomer: t_R = 42.4 min; minor enantiomer: t_R = 55.1 min, 83% ee.

Benzyl (*R*)-3-hydroxy-4-(5-methyl-1-(methoxycarbamoyl)indolin-7-yl)-2-naphthoate (3h)

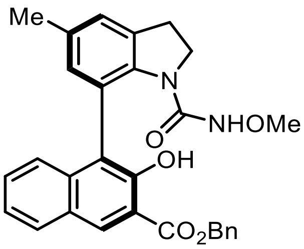

Dark red oil, 20 mg, 84%. **^{1}H-NMR (400 MHz - $CDCl_3$)**: 10.88 (s, 1H), 8.50 (s, 1H), 7.77 – 7.71 (m, 1H), 7.69 (s, 1H), 7.46 – 7.39 (m, 2H), 7.42 – 7.28 (m, 4H), 7.30 – 7.20 (m, 1H), 7.24 – 7.17 (m, 1H), 7.09 (dd, J = 1.9, 0.9 Hz, 1H), 6.95 – 6.87 (m, 1H), 5.40 (s, 2H), 4.24 (ddd, J = 11.0, 8.6, 4.2 Hz, 1H), 3.90 (dt, J = 11.0, 8.7 Hz, 1H), 3.08 (dd, J = 14.9, 8.6 Hz, 1H), 3.04 (s, 3H), 2.87 (ddd, J = 15.3, 8.4, 4.4 Hz, 1H), 2.29 (s, 3H); **^{13}C-NMR (101 MHz - $CDCl_3$):** 156.57, 152.50, 136.12, 132.81, 131.57, 129.87, 129.78, 128.82, 128.77, 128.50, 127.27, 125.91, 124.20, 123.24, 67.61, 63.65, 51.36, 29.59, 21.02; **HR-MS**: calc. for $[M+H]^+$ $C_{29}H_{27}O_5N_2$ = 483.1920 found: 483.1914; **FT-IR:** ṽ = 3213, 2928, 1672, 1586, 1447, 1301, 1254, 1179, 1060, 937, 893 cm^{-1}; **HPLC conditions:** CHIRAPAK IC column, DCM: EtOH (100:2) / *iso*-hexane = 40/60, flow rate = 0.5 mL min^{-1}, major enantiomer: t_R = 44.6 min; minor enantiomer: t_R = 39.9 min, 83% ee.

Methyl (*R*)-3-hydroxy-4-(5-chloro-1-(methoxycarbamoyl)indolin-7-yl)-2-naphthoate (3i)

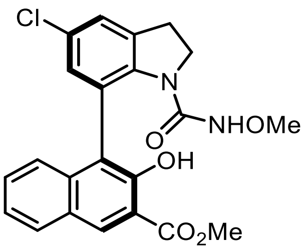

Dark yellow oil, 17 mg, 81%. **^{1}H-NMR (400 MHz - CDCl$_3$)**: 10.90 (s, 1H), 8.49 (s, 1H), 7.80 – 7.73 (m, 1H), 7.46 (s, 1H), 7.38 (ddd, J = 8.3, 6.8, 1.3 Hz, 1H), 7.32 – 7.23 (m, 1H), 7.28 – 7.21 (m, 2H), 7.19 (s, 1H), 7.11 (t, J = 1.4 Hz, 1H), 4.22 (ddd, J = 11.0, 8.8, 4.8 Hz, 1H), 4.10 – 3.90 (m, 1H), 3.98 (s, 3H), 3.17 – 3.04 (m, 1H), 3.08 (s, 3H), 3.01 – 2.90 (m, 1H); **^{13}C-NMR (101 MHz - CDCl$_3$):** 170.4, 156.1, 152.4, 137.5, 135.1, 133.1, 131.1, 129.9, 129.9, 129.8, 127.2, 125.0, 124.7, 124.2, 123.9, 120.3, 113.9, 63.7, 52.8, 51.3, 26.9; **HR-MS**: calc. for $[M+H]^+$ $C_{22}H_{20}O_5N_2Cl$ = 427.1061 found: 427.1055; **FT-IR:** ṽ = 3223, 2929, 1652, 1601, 1445, 1321, 1264, 1212, 1149, 1060, 957, 893 cm^{-1}; **HPLC conditions:** CHIRAPAK IC column, DCM: EtOH (100:2) / *iso*-hexane = 40/60, flow rate = 0.5 mL min^{-1}, major enantiomer: t_R = 43.4 min; minor enantiomer: t_R = 52.3 min, 81% ee.

Methyl (*R*)-3-hydroxy-4-(5-methoxy-1-(methoxycarbamoyl)indolin-7-yl)-2-naphthoate (3j)

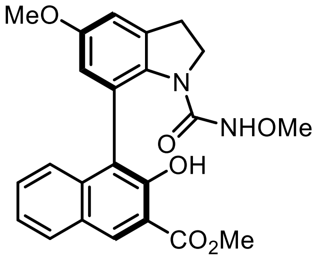

Violet oil, 18.5 mg, 88%. **^{1}H-NMR (600 MHz - CDCl$_3$)**: 10.90 (s, 1H), 8.49 (s, 1H), 7.79 – 7.74 (m, 1H), 7.68 (s, 1H), 7.36 (ddd, J = 8.3, 6.8, 1.3 Hz, 1H), 7.30 – 7.21 (m, 2H), 6.87 (dd, J = 2.5, 1.2 Hz, 1H), 6.62 (d, J = 2.6 Hz, 1H), 4.27 (ddd, J = 11.0, 8.5, 4.0 Hz, 1H), 3.98 (s, 3H), 3.95 – 3.86 (m, 1H), 3.72 (s, 3H), 3.14 – 3.05 (m, 1H), 3.08 (s, 3H), 2.88 (ddd, J = 15.4, 8.2, 4.0 Hz, 1H); **^{13}C-NMR (151 MHz - CDCl$_3$):** 170.5, 157.4, 156.7, 152.4, 137.7, 135.5, 135.2, 132.9, 129.8, 127.2, 124.4, 124.2, 124.1, 121.2, 115.0, 113.9, 111.9, 63.6, 55.7, 52.8, 51.5, 30.0; **HR-MS**: calc. for $[M+H]^+$ $C_{23}H_{23}O_6N_2$ = 423.1556 found: 423.1551; **FT-IR:** ṽ = 2939, 1678, 1599, 1493, 1440, 1385, 1305, 1232, 1173, 1084, 990, 909 cm^{-1}; **HPLC conditions:** CHIRAPAK IC column, DCM: EtOH (100:2) / *iso*-hexane = 50/50, flow rate = 0.5 mL min^{-1}, major enantiomer: t_R = 34.8 min; minor enantiomer: t_R = 44.0 min, 80% ee.

Methyl (*R*)-3-hydroxy-4-(4-(methoxycarbamoyl)-3,4-dihydro-2H-benzo[*b*][1,4]oxazin-5-yl)-2-naphthoate (3m)

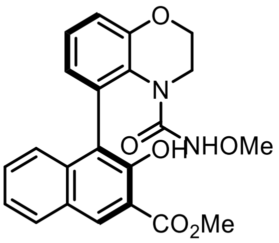

Dark red oil, 18 mg, 90%. **^{1}H-NMR (500 MHz - $CDCl_3$)**: 11.02 (s, 1H), 8.49 (s, 1H), 8.41 (s, 1H), 7.87 (s, 1H), 7.77 (dt, J = 8.2, 0.9 Hz, 1H), 7.36 (ddd, J = 8.4, 6.7, 1.4 Hz, 1H), 7.31 – 7.20 (m, 2H), 7.07 (d, J = 8.5 Hz, 1H), 7.00 (td, J = 7.9, 1.6 Hz, 1H), 6.89 – 6.80 (m, 2H), 4.31 (dt, J = 11.2, 3.1 Hz, 1H), 4.23 – 4.12 (m, 2H), 3.98 (s, 3H), 3.83 – 3.77 (m, 1H), 3.27 (s, 3H); **^{13}C-NMR (125 MHz - $CDCl_3$):** 170.5, 156.7, 152.6, 149.1, 133.2, 131.2, 130.0, 129.7, 127.6, 126.2, 125.1, 124.5, 124.2, 123.6, 122.9, 120.6, 119.8, 118.0, 117.5, 113.9, 66.3, 64.7, 63.9, 52.8, 41.8, 40.7, 26.9; **HR-MS**: calc. for $[M+H]^+$ $C_{22}H_{21}O_6N_2$ = 409.1400 found: 409.1394; **FT-IR:** ṽ = 3195, 1712, 1672, 1426, 1342, 1204, 1146, 1076, 949, 904 cm^{-1}; **HPLC conditions:** CHIRAPAK IC column, DCM: EtOH (100:2) / *iso*-hexane = 40/60, flow rate = 0.5 mL min^{-1}, major enantiomer: t_R = 22.7 min; minor enantiomer: t_R = 24.6 min, 86% ee.

Benzyl (*R*)-3-hydroxy-4-(4-(methoxycarbamoyl)-3,4-dihydro-2H-benzo[*b*][1,4]oxazin-5-yl)-2-naphthoate (3n)

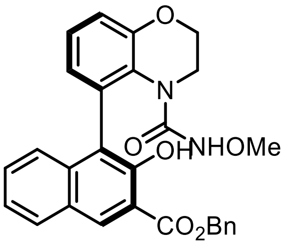

Red oil, 22 mg, 91%. **^{1}H-NMR (500 MHz - $CDCl_3$)**: 11.11 (s, 1H), 8.61 (s, 1H), 8.50 (s, 1H), 7.85 (dd, J = 8.3, 1.3 Hz, 1H), 7.56 – 7.51 (m, 2H), 7.50 – 7.39 (m, 4H), 7.39 – 7.29 (m, 2H), 7.19 – 7.15 (m, 1H), 7.09 (dd, J = 8.3, 1.4 Hz, 1H), 6.96 (dd, J = 7.5, 1.4 Hz, 1H), 5.54 – 5.44 (m, 2H), 4.41 (dt, J = 11.2, 3.1 Hz, 1H), 4.26 (dt, J = 12.2, 4.5 Hz, 2H), 3.34 (s, 3H); **^{13}C-NMR (125 MHz - $CDCl_3$):** 169.9, 156.7, 152.7, 149.2, 136.0, 134.9, 133.2, 131.2, 130.0, 129.8, 128.8(3C), 128.5(2C), 127.6, 127.1, 125.2, 124.5, 124.2, 123.6, 119.9, 117.5, 114.0, 67.7, 66.3, 63.9, 41.8; **HR-MS**: calc. for $[M+H]^+$ $C_{28}H_{25}O_6N_2$ = 485.1713 found: 485.1713; **FT-IR:** ṽ = 2929, 1748, 1652, 1441, 1301, 1264, 1211, 1172, 1020, 957, 893 cm^{-1}; **HPLC conditions:** CHIRAPAK IC column, DCM: EtOH (100:2) / *iso*-hexane = 40/60, flow rate = 0.5 mL min^{-1}, major enantiomer: t_R = 21.6 min; minor enantiomer: t_R = 23.1 min, 83% ee.

Benzyl 3-hydroxy-4-((7*R*)-1-(methoxycarbamoyl)-2-methylindolin-7-yl)-2-naphthoate (3o)

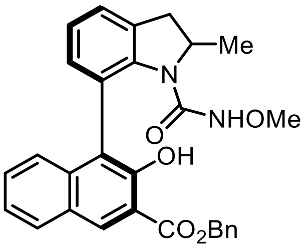

Yellow oil, 19.7 mg, 82%. **^{1}H-NMR (500 MHz - CDCl$_3$)**: 10.51 (s, 3H), 8.41 (s, 1H), 7.73 (ddt, J = 14.2, 8.2, 0.9 Hz, 2H), 7.35 – 7.25 (m, 8H), 7.20 – 7.12 (m, 2H), 6.86 (s, 1H), 5.29 (s, 2H), 4.71 – 4.57 (m, 1H), 3.29 (dddt, J = 15.6, 9.5, 8.3, 1.3 Hz, 1H), 2.62 (s, 3H), 2.41 (dd, J = 15.6, 4.8 Hz, 1H), 1.22 (d, J = 6.6 Hz, 3H); **^{13}C-NMR (125 MHz - CDCl$_3$):** 169.7, 155.9, 153.1, 141.0, 136.0, 135.0, 134.9, 132.7, 131.2, 130.2, 130.1, 129.7, 128.8, 128.7, 128.6, 128.4, 127.5, 125.7, 125.3, 124.4, 124.2, 124.1, 124.0, 123.5, 121.8, 120.9, 113.9, 102.4, 67.6, 63.2, 58.5, 36.5, 20.8; **HR-MS**: calc. for $[M+H]^+$ $C_{29}H_{27}O_5N_2$ = 483.1920 found: 483.1914; **FT-IR:** ṽ = 2935, 1679, 1441, 1340, 1316, 1270, 1215, 1154, 1072, 953, 859 cm^{-1}; **HPLC conditions:** CHIRAPAK IC column, *iso*-propanol / *iso*-hexane = 40/60, flow rate = 0.5 mL min^{-1}, major enantiomer: t_R = 25.0 min; minor enantiomer: t_R = 22.3 min, 62% ee.

Methyl (*R*)-3-hydroxy-4-(1'-(methoxycarbamoyl)spiro[cyclohexane-1,3'-indolin]-7'-yl)-2-naphthoate (3p)

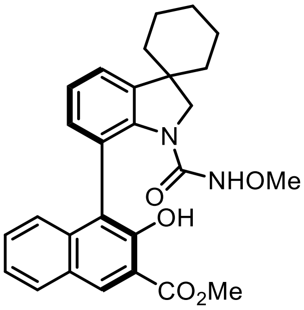

Dark-yellow oil, 23 mg, 86%. **^{1}H-NMR (600 MHz - CDCl$_3$)**: 10.92 (s, 1H), 8.49 (s, 1H), 7.84 (s, 1H), 7.75 (dd, J = 18.1, 8.1 Hz, 1H), 7.37 – 7.32 (m, 1H), 7.26 (t, J = 7.5 Hz, 1H), 7.21 (dd, J = 7.4, 1.6 Hz, 1H), 7.21 – 7.11 (m, 2H), 7.11 (dd, J = 7.2, 1.7 Hz, 1H), 4.21 (d, J = 10.9 Hz, 1H), 3.98 (s, 3H), 3.65 – 3.59 (m, 1H), 3.08 (s, 3H), 1.75 – 1.60 (m, 8H), 1.52 – 1.45 (m, 2H); **^{13}C-NMR (175 MHz - CDCl$_3$):** 170.5, 156.7, 152.4, 144.9, 140.9, 135.4, 132.8, 131.5, 129.8, 129.7, 128.0, 127.3, 125.3, 124.2, 124.1, 123.6, 122.9, 122.69, 122.5, 115.1, 113.9, 63.6, 52.8, 45.6, 37.3, 35.7, 35.5, 25.6, 23.0, 23.0; **HR-MS**: calc. for $[M+H]^+$ $C_{27}H_{28}O_5N_2$ = 461.2076 found: 461.2066; **FT-IR:** ṽ = 2936, 1678, 1599, 1493, 1385, 1305, 1232, 1173, 1085, 990, 909 cm^{-1}; **HPLC conditions:** CHIRAPAK IC column, DCM: EtOH (100:2) / *iso*-hexane = 40/60,

flow rate = 0.5 mL min^{-1}, major enantiomer: t_R = 28.1 min; minor enantiomer: t_R = 23.0 min, 82% ee.

Methyl 3-hydroxy-4-(1-(methoxycarbamoyl)-1H-indol-2-yl)-2-naphthoate (4a)

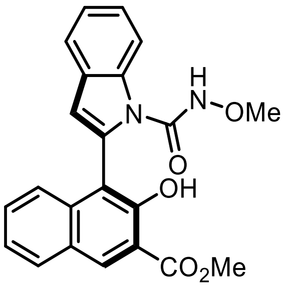

Red solid, 17 mg, 87%. **^{1}H-NMR (400 MHz - $CDCl_3$)**: 11.17 (s, 1H), 8.67 (d, J = 0.7 Hz, 1H), 8.29 (s, 1H), 8.23 – 8.14 (m, 1H), 7.93 – 7.86 (m, 1H), 7.72 – 7.54 (m, 2H), 7.56 – 7.33 (m, 4H), 7.28 (ddd, J = 8.1, 7.2, 1.0 Hz, 1H), 6.68 (d, J = 0.8 Hz, 1H), 4.08 (s, 3H), 3.43 (s, 3H); **^{13}C-NMR (101 MHz - $CDCl_3$):** 170.1, 152.2, 150.1, 138.1, 137.5, 135.0, 133.2, 133.0, 131.5, 130.1, 129.8, 129.7, 128.1, 127.2, 125.9, 124.5, 124.0, 122.0, 120.8, 119.1, 118.5, 113.4, 106.4, 63.1, 53.4; **HR-MS**: calc. for $[M+H]^+$ $C_{22}H_{19}O_5N_2$ = 391.1294 found: 391.1288; **FT-IR:** ṽ = 3223, 2929, 1652, 1601, 1445, 1321, 1264, 1212, 1149, 1060, 957, 893 cm^{-1}.

Methyl (*R*)-3-hydroxy-4-(1-(methoxycarbamoyl)-2-methyl-1H-indol-7-yl)-2-naphthoate (4b)

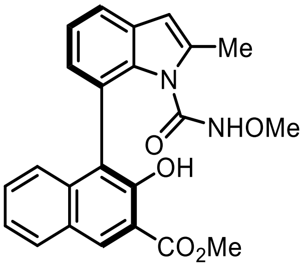

Red solid, 17 mg, 85%. **^{1}H-NMR (600 MHz - $CDCl_3$)**: 10.93 (s, 1H), 8.53 (s, 1H), 8.40 (s, 1H), 8.06 – 8.01 (m, 1H), 7.80 – 7.75 (m, 1H), 7.57 – 7.49 (m, 2H), 7.44 – 7.37 (m, 1H), 7.32 – 7.19 (m, 3H), 7.01 (dd, J = 8.4, 1.3 Hz, 1H), 6.98 (dd, J = 7.4, 1.2 Hz, 1H), 6.33 (q, J = 1.1 Hz, 1H), 3.98 (s, 3H), 3.23 (s, 3H), 2.30 (s, 3H); **^{13}C-NMR (151 MHz - $CDCl_3$):** 170.6, 152.8, 151.5, 137.1, 136.9, 135.0, 133.7, 133.0, 130.2, 130.1, 129.8, 129.7, 128.5, 127.2, 125.9, 124.5, 124.2, 122.0, 120.4, 119.9, 118.9, 113.9, 104.4, 63.8, 52.7, 13.1; **HR-MS**: calc. for $[M+H]^+$ $C_{23}H_{21}O_5N_2$ = 405.1450 found: 405.1444; **FT-IR:** ṽ = 2935, 1681, 1439, 1340, 1316, 1270, 1215, 1072, 953, 910 cm^{-1}; **HPLC conditions:** CHIRAPAK IC column, DCM: EtOH (100:2) / *iso*-hexane = 40/60, flow rate = 0.5 mL min^{-1}, major enantiomer: t_R = 25.7 min; minor enantiomer: t_R = 17.3 min, 78% ee.

Benzyl (*R*)-3-hydroxy-4-(1-(methoxycarbamoyl)-2-methyl-1H-indol-7-yl)-2-naphthoate (4c)

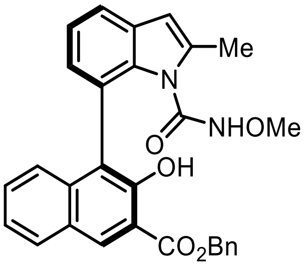

Orange oil, 21 mg, 89%. **^{1}H-NMR (600 MHz - $CDCl_3$)**: 11.02 (s, 1H), 8.64 (s, 1H), 8.51 (s, 1H), 7.88 – 7.82 (m, 1H), 7.55 – 7.38 (m, 5H), 7.40 – 7.27 (m, 3H), 7.06 (td, J = 7.9, 7.2, 1.2 Hz, 1H), 6.41 (s, 1H), 5.52 (d, J = 12.2 Hz, 2H), 3.26 (s, 3H), 2.37 (s, 3H); **^{13}C-NMR (151 MHz - $CDCl_3$):** 170.2, 153.0, 151.7, 137.3, 137.1, 135.3, 135.1, 133.3, 130.3, 130.2, 130.0, 129.9, 128.9, 128.6, 128.6, 127.3, 126.1, 124.7, 124.4, 122.2, 120.6, 120.1, 119.1, 114.2, 104.5, 67.6, 63.9, 13.3; **HR-MS**: calc. for $[M+H]^+$ $C_{29}H_{25}O_5N_2$ = 481.1763 found: 481.1754; ; **FT-IR:** ṽ = 2935, 1678, 1445, 1367, 1301, 1266, 1215, 1071, 953, 909 cm^{-1}; **HPLC conditions:** CHIRAPAK IC column, *iso*-Propanol / *iso*-hexane = 30/70, flow rate = 0.5 mL min^{-1}, major enantiomer: t_R = 19.6 min; minor enantiomer: t_R = 60.5 min, 82% ee.

Methyl (*R*)-3-hydroxy-4-(1-(methoxycarbamoyl)-2,3-dimethyl-1H-indol-7-yl)-2-naphthoate (4d)

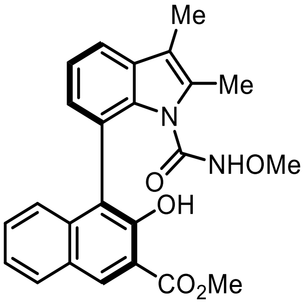

Yellow solid, 18 mg, 88%. **^{1}H-NMR (600 MHz - $CDCl_3$)**: 10.98 (s, 1H), 8.60 (s, 1H), 8.42 (s, 1H), 8.06 – 8.01 (m, 1H), 7.86 (d, J = 8.4 Hz, 1H), 7.57 (d, J = 8.1 Hz, 1H), 7.38 – 7.31 (m, 3H), 7.09 – 7.05 (m, 2H), 4.05 (s, 3H), 3.29 (s, 3H), 2.31 (s, 3H), 2.27 (s, 3H); **^{13}C-NMR (151 MHz - $CDCl_3$):** 170.8, 153.0, 152.1, 137.0, 134.4, 133.1, 132.9, 131.3, 130.3, 129.9, 129.9, 129.9, 128.6, 127.4, 126.2, 124.7, 124.4, 121.8, 120.8, 119.0, 118.4, 114.1, 111.1, 63.9, 52.9, 10.8, 8.9; **HR-MS**: calc. for $[M+H]^+$ $C_{24}H_{23}O_5N_2$ = 419.1607 found: 481.1599; ; **FT-IR:** ṽ = 2935, 1679, 1441, 1340, 1316, 1270, 1215, 1155, 1072, 953, 910 cm^{-1}; **HPLC conditions:** CHIRAPAK IC column, *iso*-Propanol / *iso*-hexane = 30/70, flow rate = 0.5 mL min^{-1}, major enantiomer: t_R = 36.7 min; minor enantiomer: t_R = 41.6 min, 88% ee.

Benzyl (*R*)-3-hydroxy-4-(1-(methoxycarbamoyl)-2,3-dimethyl-1H-indol-7-yl)-2-naphthoate (4e)

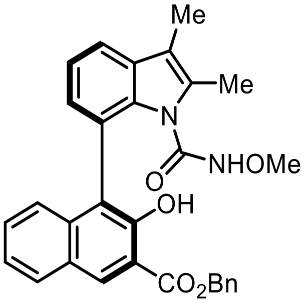

Orange oil, 22 mg, 90%. **^{1}H-NMR (500 MHz - $CDCl_3$)**: 10.94 (s, 1H), 8.56 (s, 1H), 8.41 (s, 1H), 8.06 – 8.01 (m, 1H), 7.82 – 7.74 (m, 1H), 7.49 (dd, J = 7.8, 1.2 Hz, 2H), 7.45 (d, J = 1.8 Hz, 1H), 7.46 – 7.35 (m, 5H), 7.38 – 7.29 (m, 2H), 7.32 – 7.20 (m, 4H), 7.02 – 6.95 (m, 2H), 5.41 (d, J = 17.1 Hz, 2H), 3.18 (s, 3H), 2.21 (s, 3H), 2.19 (s, 3H); **^{13}C-NMR (126 MHz - $CDCl_3$):** 170.0, 152.8, 135.1, 133.7, 133.0, 132.7, 131.0, 130.2, 129.8, 129.7, 128.8, 128.7, 128.5, 128.4, 127.1, 126.0, 124.5, 124.2, 121.6, 118.7, 118.2, 113.9, 110.9, 67.4, 63.7, 10.6, 8.7; **HR-MS**: calc. for $[M+H]^+$ $C_{30}H_{27}O_5N_2$ = 495.1920 found: 495.1914; ; **FT-IR:** ṽ = 2936, 1679, 1441, 1340, 1316, 1271, 1216, 1072, 983, 909 cm^{-1}; **HPLC conditions:** CHIRAPAK IA column, DCM: EtOH (100:2) / *iso*-hexane = 15/85, flow rate = 0.5 mL min^{-1}, major enantiomer: t_R = 48.5 min; minor enantiomer: t_R = 34.1min, 84% ee.

Benzyl (*R*)-4-(5-chloro-1-(methoxycarbamoyl)-2-methyl-1H-indol-7-yl)-3-hydroxy-2-naphthoate (4f)

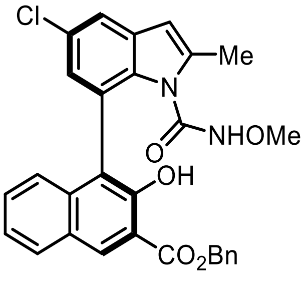

Yellow solid, 21 mg, 81%. **^{1}H-NMR (600 MHz - $CDCl_3$)**: 11.06 (s, 1H), 8.65 (s, 1H), 8.45 (s, 1H), 7.85 (d, J = 7.9 Hz, 1H), 7.56 (d, J = 2.0 Hz, 1H), 7.53 – 7.37 (m, 6H), 7.31 (ddd, J = 8.0, 6.8, 1.2 Hz, 1H), 7.11 – 7.04 (m, 2H), 6.35 (s, 1H), 5.52 (d, J = 12.2 Hz, 2H), 3.25 (s, 3H), 2.36 (s, 3H); **^{13}C-NMR (151 MHz - $CDCl_3$):** 170.1, 153.1, 151.2, 138.7, 136.9, 135.3, 133.7, 131.3, 130.3, 130.2, 130.0, 129.0, 128.9, 128.6, 127.6, 127.3, 125.8, 124.6, 124.4, 120.4, 119.5, 114.1, 104.0, 67.7, 64.0, 13.2; **HR-MS**: calc. for $[M+H]^+$ $C_{29}H_{24}O_5N_2Cl$ = 515.1374 found: 515.1368; ; **FT-IR:** ṽ = 3127, 2349, 1685, 1497, 1422, 1383, 1285, 1219, 1161, 1118, 1042, 951, 925 cm^{-1}; **HPLC conditions:** CHIRAPAK IC column, DCM: EtOH (100:2) / *iso*-hexane

= 30/70, flow rate = 0.5 mL min^{-1}, major enantiomer: t_R = 24.0 min; minor enantiomer: t_R = 27.0 min, 90% ee.

Methyl (*R*)-7-bromo-4-(5-chloro-1-(methoxycarbamoyl)-2-methyl-1H-indol-7-yl)-3-hydroxy-2-naphthoate (4g)

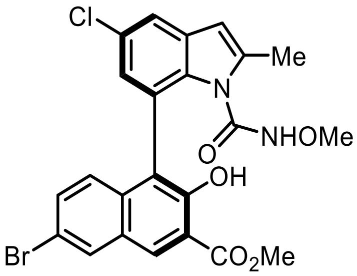

Dark orange solid, 22 mg, 86%. **^{1}H-NMR (500 MHz - $CDCl_3$)**: δ 11.11 (s, 1H), 8.54 (s, 1H), 8.45 (s, 1H), 8.03 (d, J = 2.0 Hz, 1H), 7.59 (d, J = 2.1 Hz, 1H), 7.53 – 7.43 (m, 1H), 1 7.06 – 6.98 (m, 2H), 6.37 (s, 1H), 4.09 (s, 3H), 3.39 (s, 3H), 2.39 (s, 3H); **^{13}C-NMR (125 MHz - $CDCl_3$):** 170.2, 153.2, 150.9, 138.6, 135.0, 133.4, 133.1, 132.3, 131.4, 131.1, 128.0, 127.5, 126.2, 125.5, 119.6, 119.5, 119.4, 118.2, 114.8, 103.9, 64.0, 53.0, 13.1; **HR-MS**: calc. for $[M+H]^+$ $C_{23}H_{19}O_5N_2ClBr$ = 517.0166 and 519.0145 found: 517.0160 and 519.0141; ; **FT-IR:** ṽ = 2934, 1679, 1441, 1340, 1315, 1270, 1218, 1070, 953, 909, 859 cm^{-1}; **HPLC conditions:** CHIRAPAK IC column, DCM: EtOH (100:2) / *iso*-hexane = 30/70, flow rate = 0.5 mL min^{-1}, major enantiomer: t_R = 38.7 min; minor enantiomer: t_R = 34.2 min, 85% ee.

Methyl (*R*)-4-(5-bromo-1-(methoxycarbamoyl)-2-methyl-1H-indol-7-yl)-3-hydroxy-2-naphthoate (4h)

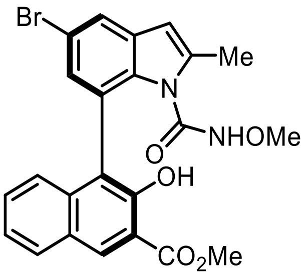

Yellow solid, 18.7 mg, 78%. **^{1}H-NMR (600 MHz - $CDCl_3$)**: 11.05 (s, 1H), 8.61 (s, 1H), 8.41 (s, 1H), 7.89 – 7.84 (m, 1H), 7.72 (d, J = 1.9 Hz, 1H), 7.40 (ddd, J = 8.4, 6.7, 1.4 Hz, 1H), 7.35 (ddd, J = 8.0, 6.7, 1.2 Hz, 1H), 7.18 (d, J = 1.9 Hz, 1H), 7.10 (dd, J = 8.4, 1.1 Hz, 1H), 6.35 (s, 1H), 4.06 (s, 3H), 3.30 (s, 3H), 2.37 (s, 3H); **^{13}C-NMR (151 MHz - $CDCl_3$):** 170.7, 153.1, 151.1, 138.6, 136.8, 134.1, 133.6, 131.8, 130.3, 130.0, 128.4, 127.3, 124.6, 124.4, 122.6, 120.8, 119.1, 115.2, 114.1, 103.9, 64.0, 53.0, 13.2; **HR-MS**: calc. for $[M+H]^+$ $C_{23}H_{20}O_5N_2Br$ = 483.0556 and 485.0535 found: 483.0550 and 485.0529; ; **FT-IR:** ṽ = 3129, 1682, 1588, 1473, 1450, 1386, 1286, 1221, 1172, 1146, 970, 929, 858 cm^{-1}; **HPLC conditions:** CHIRAPAK IC

column, *iso*-Propanol / *iso*-hexane = 30/70, flow rate = 0.5 mL min^{-1}, major enantiomer: t_R = 17.0 min; minor enantiomer: t_R = 59.6 min, 90% ee.

Benzyl (*R*)-4-(5-methoxy-1-(methoxycarbamoyl)-2-methyl-1H-indol-7-yl)-3-hydroxy-2-naphthoate (4i)

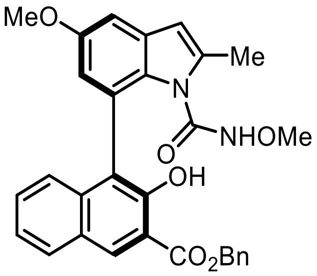

Pink oil, 20.4 mg, 80%. **^{1}H-NMR (400 MHz - CDCl$_3$)**: 10.95 (s, 1H), 8.56 (s, 1H), 8.40 (s, 1H), 7.80 – 7.73 (m, 1H), 7.49 – 7.38 (m, 2H), 7.40 – 7.34 (m, 2H), 7.37 – 7.30 (m, 2H), 7.34 – 7.18 (m, 3H), 7.09 – 6.98 (m, 2H), 6.63 (d, J = 2.4 Hz, 1H), 6.25 (s, 1H), 5.44 (q, J = 12.2 Hz, 2H), 3.78 (s, 3H), 3.17 (s, 3H), 2.28 (s, 3H); **^{13}C-NMR (101 MHz - CDCl$_3$):** 170.0, 155.5, 152.8, 151.6, 138.1, 136.7, 135.1, 133.2, 131.1, 130.0, 129.9, 129.7, 128.8, 128.7, 128.5, 128.4, 127.1, 124.5, 124.3, 120.1, 119.9, 114.5, 114.0, 104.5, 102.7, 67.5, 63.8, 55.7, 13.4; **HR-MS**: calc. for $[M+H]^+$ $C_{30}H_{27}O_6N_2$ = 511.1869 found: 511.1863; ; **FT-IR:** ṽ = 2935, 1681, 1439, 1340, 1316, 1270, 1215, 1072, 953, 910 cm^{-1}; **HPLC conditions:** CHIRAPAK IC column, *iso*-Propanol / *iso*-hexane = 30/70, flow rate = 0.5 mL min^{-1}, major enantiomer: t_R = 27.3 min; minor enantiomer: t_R = 67.6 min, 82% ee.

Methyl (*R*)-3-hydroxy-4-(3-(methoxycarbamoyl)-1,2-dimethyl-3H-benzo[e]indol-4-yl)-2-naphthoate (4j)

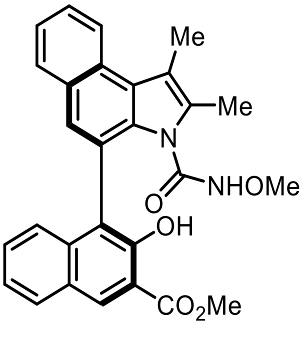

Off-white solid, 20 mg, 85%. **^{1}H-NMR (600 MHz - CDCl$_3$)**: δ 11.03 (s, 1H), 8.64 (s, 1H), 8.61 (d, J = 8.5 Hz, 1H), 8.44 (s, 1H), 7.94 – 7.88 (m, 1H), 7.90 – 7.84 (m, 1H), 7.60 (ddd, J = 8.4, 6.8, 1.4 Hz, 1H), 7.50 (s, 1H), 7.46 (ddd, J = 8.1, 6.7, 1.2 Hz, 1H), 7.33 (dt, J = 6.5, 3.4 Hz, 2H), 7.12 (dt, J = 6.3, 3.6 Hz, 1H), 4.06 (d, J = 8.4 Hz, 4H), 3.36 (s, 3H), 2.69 (s, 3H); **^{13}C-NMR (151 MHz - CDCl$_3$):** 170.8, 153.3, 137.4, 133.4, 131.3, 130.7, 130.0, 129.9, 129.0, 128.8, 127.3, 126.4, 126.1, 124.8, 124.4, 123.9, 123.6, 120.3, 120.2, 114.2, 112.9, 64.0, 52.9, 13.0, 10.3; **HR-**

MS: calc. for $[M+H]^+$ $C_{28}H_{25}O_5N_2$ = 469.1763 found: 469.1758; ; **FT-IR:** ṽ = 2935, 1679, 1442, 1341, 1315, 1270, 1215, 1154, 1072, 953, 909 cm^{-1}; **HPLC conditions:** CHIRAPAK IC column, DCM: EtOH (100:2) / *iso*-hexane = 40/60, flow rate = 0.5 mL min^{-1}, major enantiomer: t_R = 16.7 min; minor enantiomer: t_R = 29.8 min, 82% ee.

Determination of the absolute configuration of 3a by VCD spectroscopy

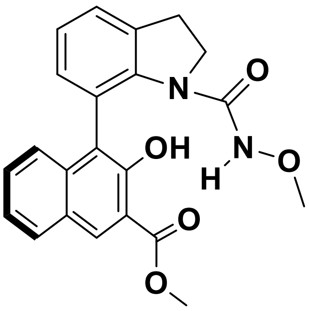

The configuration of the **3a** was determined by means of VCD spectroscopy. Experimental IR and VCD spectra of the enantiopure sample were obtained for solution in $CDCl_3$ (38 mM = 15 mg/ml) at 200 µm pathlength. The spectra were recorded on a Bruker Vertex 70 equipped with a PMA 50 module for VCD measurements. About 64000 scans were accumulated for the VCD spectra (approximately 10 hrs accumulation time). The baseline was corrected by subtraction of the spectrum of the racemic sample recorded under identical conditions. [3]

In order to analyse the experimental IR and VCD spectra, a systematic conformational analysis was carried out of the (*R*)-stereoisomer by manually generating starting structures through rotation about rotable bonds. The so-obtained structures were subjected to geometry optimizations at DFT level using Gaussian 09 Rev E.01[1] at B3LYP/6-311+g(2d,p)/IEFPCM($CHCl_3$) level of theory. The final conformer energies after geometry optimization are summarized in Table S1. The lowest energy conformation is shown in **Figure S1**.

Table S1. Conformer energies of (*R*)-isomer of **3a**. Cartesian coordinates are provided below.

conformer	E / hartree	G / hartree	ΔE_{ZPC}	ΔG_{298K}	Pop(ΔE)	Pop(ΔG)
C1	-1336.016949	-1336.072878	0.00	0.00	91.0	86.1
C2	-1336.015286	-1336.071239	1.04	1.03	6.0	5.9
C3	-1336.014862	-1336.071422	1.31	0.91	3.0	8.0
C4	-1336.011688	-1336.068372	3.30	2.83	0.0	0.1
C5	-1335.9997	-1336.057262	10.82	9.80	0.0	0.0

The IR and VCD spectra of the (*R*)-isomer were simulated using the Boltzmann weights determined based on the relative zero-point corrected energies ΔE_{ZPC}. A comparison of the obtained final spectra with the experimental IR and VCD spectra is also in **Figure S1.** The visual comparison of the predicted VCD pattern of (*R*)-isomer reveals an exceptionally good agreement with the experimental spectrum of **3a** as all characteristic bands are found well reproduced. Hence, based on the VCD spectra, the configuration of **3a** is confirmed as (*R*).

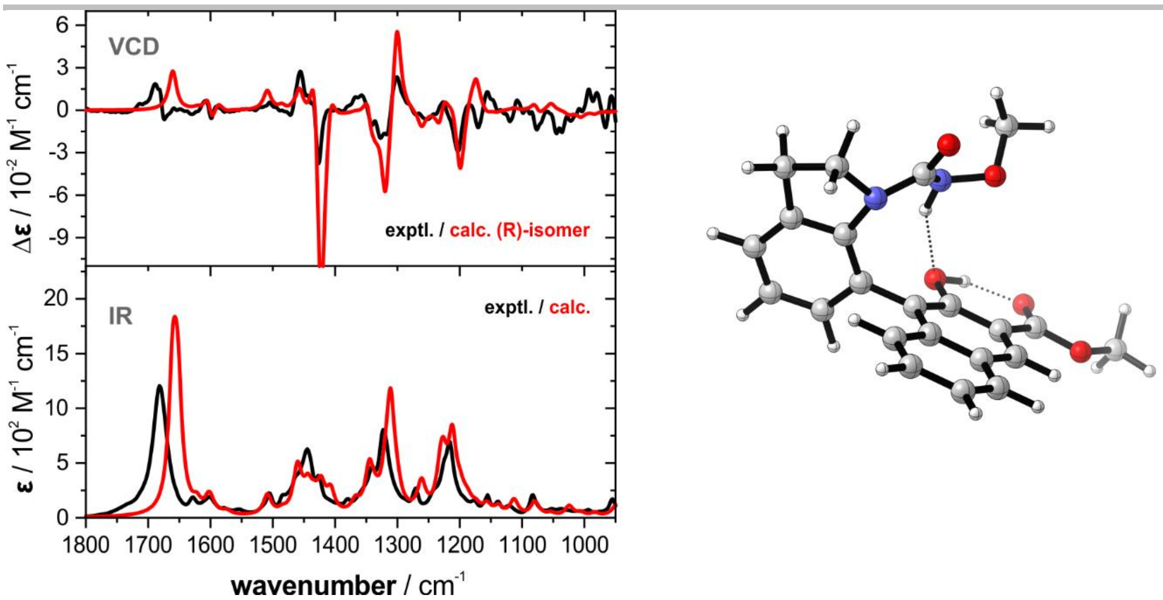

Figure S1. Left: Comparison of experimental and computed IR and VCD spectra of **3a** and the (*R*)-isomer (6 cm^{-1} HWHH, σ=0.98). Right: Structure of the lowest energy conformer C1.

Cartesian coordinates

C1

C	0.61698200	4.21876700	-1.68435900
C	1.68068200	3.40402500	-1.40303300
C	1.51074100	2.22884200	-0.62502900
C	0.21090300	1.88462100	-0.13345400
C	-0.86510000	2.75896400	-0.43845800
C	-0.66566600	3.88890200	-1.19104500
H	3.58391200	1.65604200	-0.68938500
H	0.75280300	5.11343400	-2.27927200
H	2.67235400	3.64400800	-1.76960900
C	2.60126400	1.39605200	-0.31921000
C	0.04361600	0.70139700	0.64390900
H	-1.85474900	2.52794300	-0.06810800
H	-1.50391700	4.53956500	-1.41083100
C	1.14360200	-0.08913400	0.92217900
C	2.44549700	0.25562000	0.43954400
C	-1.29013600	0.34648200	1.21927300
C	-2.34993400	-0.18402300	0.47777200
C	-1.54821300	0.63116600	2.56945400
C	-3.63608700	-0.30484600	1.02199600
C	-2.80450100	0.45390100	3.13263600
H	-0.74282600	1.03095600	3.17369300
C	-3.86876000	0.00187300	2.34892300
C	-4.59739600	-0.74900100	-0.05588800
C	-3.79759700	-0.46447100	-1.34274300
H	-2.96438700	0.69978800	4.17528600
H	-4.86187800	-0.09462500	2.77238600
H	-5.54083700	-0.20347300	-0.03171600
H	-4.01222400	0.53766300	-1.72130400
H	-3.96352700	-1.17943800	-2.14308100
H	-4.82623400	-1.81556800	0.03967000
N	-2.38003100	-0.52858000	-0.90368000
C	-1.53509400	-1.39433300	-1.58563700
O	-1.62389800	-1.55917400	-2.79341500
N	-0.67304200	-2.13717300	-0.80107500
H	-0.45390100	-1.84416700	0.14406400
C	0.39221900	-3.97449500	-1.75337400
H	0.24416300	-4.56639000	-0.84707300
H	-0.42155600	-4.14978700	-2.45840900
H	1.34572100	-4.23150100	-2.21359800
O	0.49391600	-2.58014400	-1.43718300
O	0.94750400	-1.22405500	1.63849100
H	1.82483700	-1.66986400	1.72188500
C	3.58750000	-0.62393900	0.75224600
O	3.48872300	-1.65619000	1.41035400
O	4.75146600	-0.20478300	0.25882400
C	5.90376200	-1.03276000	0.53078800
H	5.75773500	-2.02720900	0.11282000
H	6.73520200	-0.53011400	0.04593500
H	6.06943300	-1.10388100	1.60442100

C2

C	0.60658100	4.14258600	-1.63486700
C	1.67638900	3.33507300	-1.35790600
C	1.51618500	2.15368600	-0.58622600
C	0.21901000	1.79447800	-0.09648900
C	-0.86339900	2.66428500	-0.39580200
C	-0.67330500	3.79972600	-1.14173600
H	3.59606000	1.60772300	-0.64163200
H	0.73497300	5.04215500	-2.22402200
H	2.66654700	3.58590900	-1.72148800
C	2.61439900	1.33301900	-0.27950100
C	0.06212300	0.60703600	0.67408300
H	-1.85090400	2.42609600	-0.02483600
H	-1.51653600	4.44597300	-1.35552400
C	1.16907800	-0.17729300	0.94988300
C	2.46855200	0.18729300	0.47520500
C	-1.26272400	0.23864300	1.25733700
C	-2.36759800	-0.21137600	0.52676500
C	-1.45791900	0.43568900	2.63534100
C	-3.63295500	-0.33088400	1.12359300
C	-2.69166200	0.25577200	3.24157600
H	-0.61552600	0.77003000	3.22841200
C	-3.80086800	-0.11107400	2.47630400
C	-4.65897500	-0.67296200	0.07070100
C	-3.91181000	-0.33023100	-1.23033600
H	-2.79891600	0.43379300	4.30458600
H	-4.77840000	-0.21022500	2.93392100
H	-5.58058500	-0.10058100	0.17772900
H	-4.09423500	0.70677900	-1.52193600
H	-4.14689100	-0.97653100	-2.06897700
H	-4.91961600	-1.73595000	0.10816600
N	-2.47771300	-0.48335900	-0.87300300
C	-1.74189500	-1.39755800	-1.61815700

O	-2.06250600	-1.71218400	-2.75463300
N	-0.58335000	-1.86969500	-1.01839300
H	-0.56620100	-1.97123000	-0.00949100
C	1.04012500	-2.69617600	-2.46733000
H	0.70198900	-2.06555500	-3.29085000
H	1.83566600	-2.19713000	-1.91005000
H	1.39636700	-3.65062700	-2.85350400
O	-0.05404300	-3.02880500	-1.60447900
O	0.98354400	-1.32087500	1.65051600
H	1.86593000	-1.75130700	1.74608400
C	3.62257400	-0.67449600	0.79245900
O	3.53695400	-1.71373400	1.44126300
O	4.78422300	-0.23187900	0.31178500
C	5.94895100	-1.03909000	0.59328600
H	5.82683100	-2.03481800	0.17058400
H	6.77603400	-0.51893200	0.11952800
H	6.10432400	-1.11125200	1.66836100

C3

C	-1.95765800	4.17490400	-0.24422500
C	-2.70517600	3.02921800	-0.18344000
C	-2.10913300	1.75810300	-0.38877600
C	-0.70860300	1.66409000	-0.67431700
C	0.03473900	2.87560600	-0.71761800
C	-0.57302700	4.08880800	-0.50860000
H	-3.92706900	0.64979400	-0.08639400
H	-2.41879100	5.14164800	-0.08494300
H	-3.76752700	3.07399600	0.02777600
C	-2.87052600	0.58006600	-0.30760900
C	-0.11185000	0.38153200	-0.87448700
H	1.09701500	2.84036400	-0.91788900
H	0.02012000	4.99496600	-0.54554600
C	-0.90355000	-0.75832900	-0.79762900
C	-2.30229600	-0.66028900	-0.50219000
C	1.32610600	0.23665100	-1.24824900
C	2.28005300	-0.46524400	-0.49855900
C	1.75216400	0.75292500	-2.48460200
C	3.55789300	-0.72348600	-1.01865300
C	3.03369000	0.54995400	-2.97326800
H	1.03368900	1.29453400	-3.08778300
C	3.94546400	-0.21254400	-2.24155300
C	4.31840300	-1.62521200	-0.07729100
C	3.19718600	-2.18086400	0.81653500
H	3.31066200	0.95281100	-3.93972200
H	4.93527700	-0.41579100	-2.63386700
H	5.04488800	-1.05422400	0.51044500
H	3.48488000	-2.34201700	1.85061600
H	2.80092600	-3.11590400	0.41329700
H	4.85884100	-2.41636000	-0.59695400
N	2.14452800	-1.13749300	0.75137400
C	1.58760100	-0.71621600	1.94190500
O	1.55536700	-1.43358800	2.93087700
N	1.17446500	0.61424600	1.98696100
H	0.87354100	1.03009300	1.11591200
C	0.76769000	1.57692500	4.06578200
H	1.52031500	0.96012200	4.55927800
H	1.20647700	2.52398300	3.74280900
H	-0.06268700	1.76455200	4.74580600
O	0.19085200	0.87998100	2.95610900
O	-0.33507100	-1.95609700	-1.03088100
H	-1.03766400	-2.64007700	-0.92590100
C	-3.11245400	-1.89207000	-0.42288800
O	-2.65888300	-3.01723100	-0.60912200
O	-4.39516100	-1.68284400	-0.12951400
C	-5.23407200	-2.85523000	-0.03288800
H	-4.86640800	-3.51535100	0.75069900
H	-6.22174700	-2.47772000	0.21421400
H	-5.25038200	-3.38423700	-0.98421600

C4

C	0.21076000	4.44470700	-1.42707100
C	1.35502600	3.71202700	-1.25802600
C	1.32711400	2.47188500	-0.56811200
C	0.09053200	1.97552100	-0.04900300
C	-1.07263500	2.76736000	-0.23545600
C	-1.01123800	3.96365900	-0.90512500
H	3.43984600	2.08611200	-0.76021800
H	0.23707200	5.38985200	-1.95512400
H	2.30203800	4.06766200	-1.64758100
C	2.49725700	1.71947000	-0.37423100
C	0.07255200	0.72698900	0.63874000
H	-2.01815300	2.42082700	0.15912300
H	-1.91322200	4.54989600	-1.03584100
C	1.24936600	0.01867200	0.80594500
C	2.49060200	0.51503000	0.29688200
C	-1.19595000	0.21580500	1.24510900
C	-2.24585000	-0.34690900	0.51340500
C	-1.40895400	0.38282200	2.62259000
C	-3.49013800	-0.61101700	1.10243100
C	-2.61760000	0.06237500	3.22537100
H	-0.60968200	0.80613400	3.21892900
C	-3.68031900	-0.41951600	2.45750700
C	-4.46623000	-1.05251700	0.03676600
C	-3.75492100	-0.61226600	-1.25836100
H	-2.74488900	0.22081300	4.28926100
H	-4.64052200	-0.62701700	2.91555100
H	-5.44656600	-0.58774600	0.14305200
H	-4.06361600	0.39448900	-1.54914700
H	-3.90487900	-1.27805600	-2.10310300
H	-4.60742500	-2.13812600	0.06103100
N	-2.31625600	-0.59459200	-0.88777900
C	-1.44665000	-1.35431400	-1.65978300
O	-1.58058300	-1.45366600	-2.87017100
N	-0.49827600	-2.07830900	-0.96079400
H	-0.24967900	-1.81781800	-0.01355900
C	0.62945300	-3.77565600	-2.08508200
H	0.56243300	-4.43613400	-1.21716100
H	-0.20666900	-3.95432400	-2.76230100
H	1.57208400	-3.93958600	-2.60624400
O	0.66247800	-2.40237900	-1.67596700
O	1.15348900	-1.17967100	1.44693100
H	2.03761500	-1.58927800	1.47663600
C	3.80105400	-0.17456100	0.42331400
O	4.84890400	0.25240000	-0.00109100
O	3.72423400	-1.36336400	1.07730900
C	4.95920800	-2.10085100	1.23257200
H	5.67425600	-1.50978400	1.80093100
H	4.68825200	-3.00324400	1.77246900
H	5.37061000	-2.34321900	0.25491400

C5

C	-1.37632100	4.11402700	1.82032900
C	-2.22915200	3.19718700	1.26248500
C	-1.73144900	2.12297300	0.48316800
C	-0.32561600	1.99764300	0.26448100
C	0.52774200	2.95318000	0.87276200
C	0.01525100	3.98055300	1.62661000
H	-3.66200400	1.23605700	0.12496800
H	-1.76324400	4.93139200	2.41645500
H	-3.29982300	3.27597200	1.41417200
C	-2.59939200	1.15450900	-0.06335900
C	0.16515600	0.91903100	-0.53511000
H	1.59798000	2.86275200	0.74773800
H	0.68908800	4.69451000	2.08548800
C	-0.72956800	-0.00077400	-1.05753500
C	-2.12709500	0.09217500	-0.79616700
C	1.61260400	0.78630700	-0.87021600
C	2.39695200	-0.30587200	-0.47077600
C	2.24500300	1.75973300	-1.66437300
C	3.72992500	-0.43648800	-0.88563400
C	3.57246300	1.65011100	-2.05109200
H	1.66395800	2.61556900	-1.98644200
C	4.32280800	0.53454800	-1.66773800
C	4.27237500	-1.76150600	-0.41073200
C	3.23413700	-2.19663900	0.63607100
H	4.02045300	2.42318500	-2.66290800
H	5.35461700	0.43175400	-1.98402000
H	5.27455500	-1.69489400	0.01376500
H	3.55049600	-1.89687200	1.64004300
H	3.03627500	-3.26695400	0.63195600
H	4.31260500	-2.47284300	-1.24173000
N	1.99454000	-1.44493200	0.26498500
C	1.07543500	-1.29201600	1.36662900

O	1.22916300	-0.52216600	2.28832400
N	0.08226300	-2.22061500	1.26604300
H	-0.19139400	-2.53383200	0.34068100
C	-0.91298500	-3.16132100	3.14250500
H	-0.95876800	-4.14744400	2.67532900
H	-0.00122100	-3.06344800	3.73424900
H	-1.78533600	-3.00767800	3.77547900
O	-0.98606900	-2.12585600	2.14950200
O	-0.32105900	-1.01561500	-1.86840000
H	0.62425700	-0.90699300	-2.05147100
C	-3.06052300	-0.98467200	-1.22881000
O	-2.79345400	-2.16532400	-1.26065000
O	-4.27629100	-0.49684500	-1.54355900
C	-5.28076700	-1.47494700	-1.87824100
H	-5.44174200	-2.15424700	-1.04197300
H	-6.18132800	-0.90378600	-2.08596300
H	-4.97725500	-2.04337200	-2.756295

NMR and HPLC Spectra:

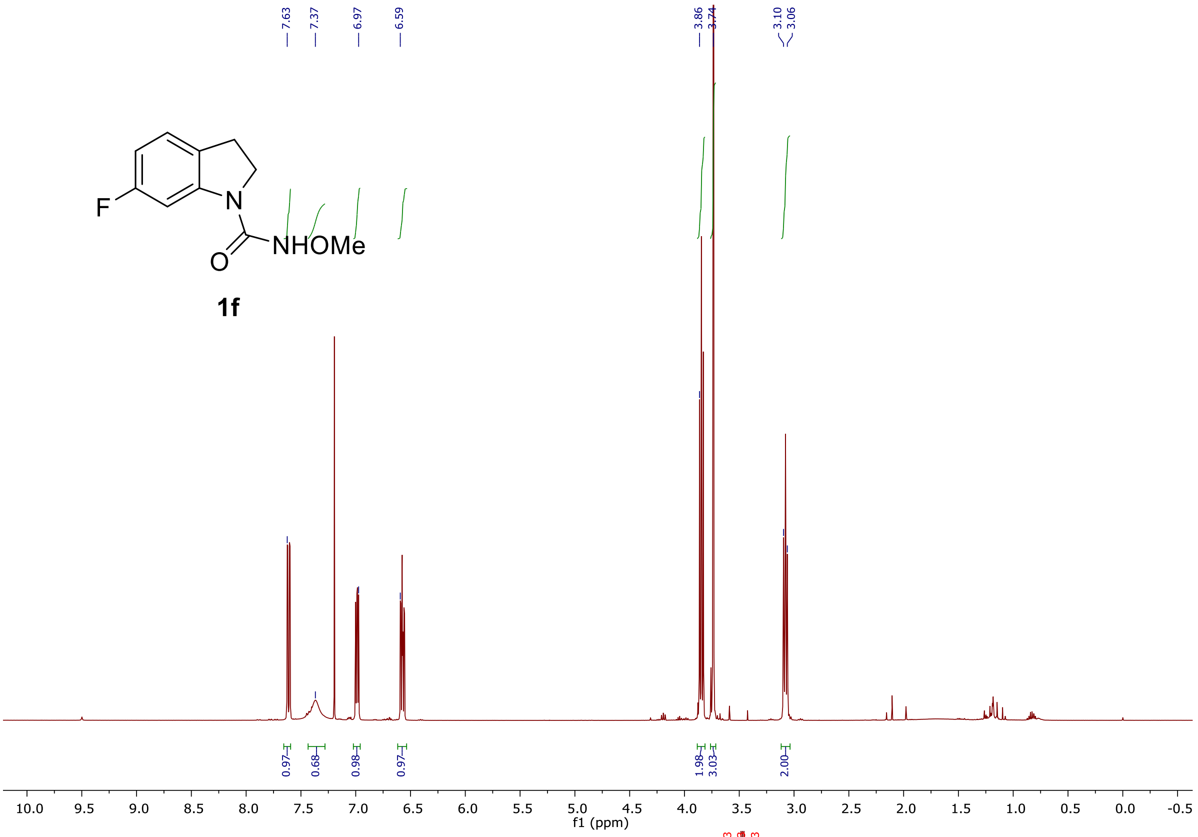

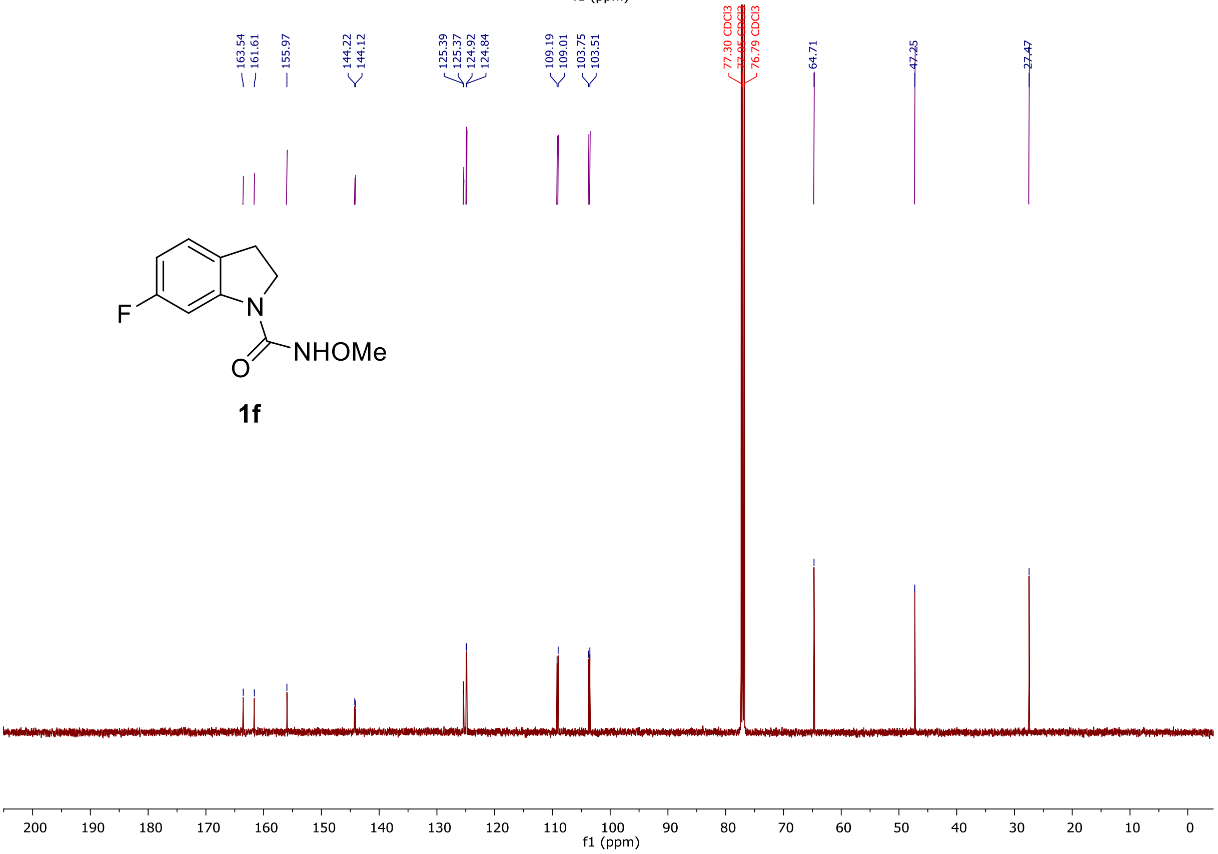

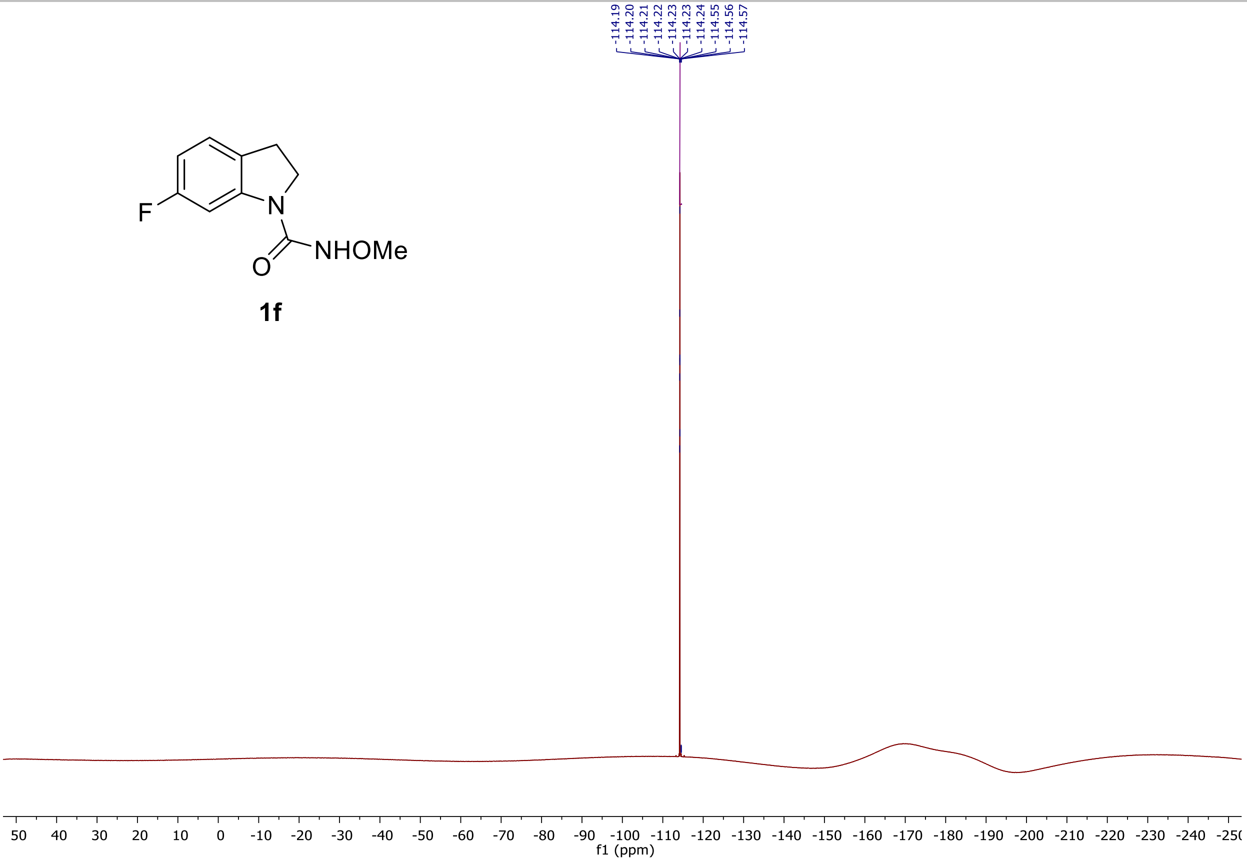

-114.19
-114.20
-114.21
-114.22
-114.23
-114.23
-114.24
-114.55
-114.56
-114.57
50 40 30 20 10 0 -10 -20 -30 -40 -50 -60 -70 -80 -90 -100 -110 -120 -130 -140 -150 -160 -170 -180 -190 -200 -210 -220 -230 -240
f1 (ppm)

1f

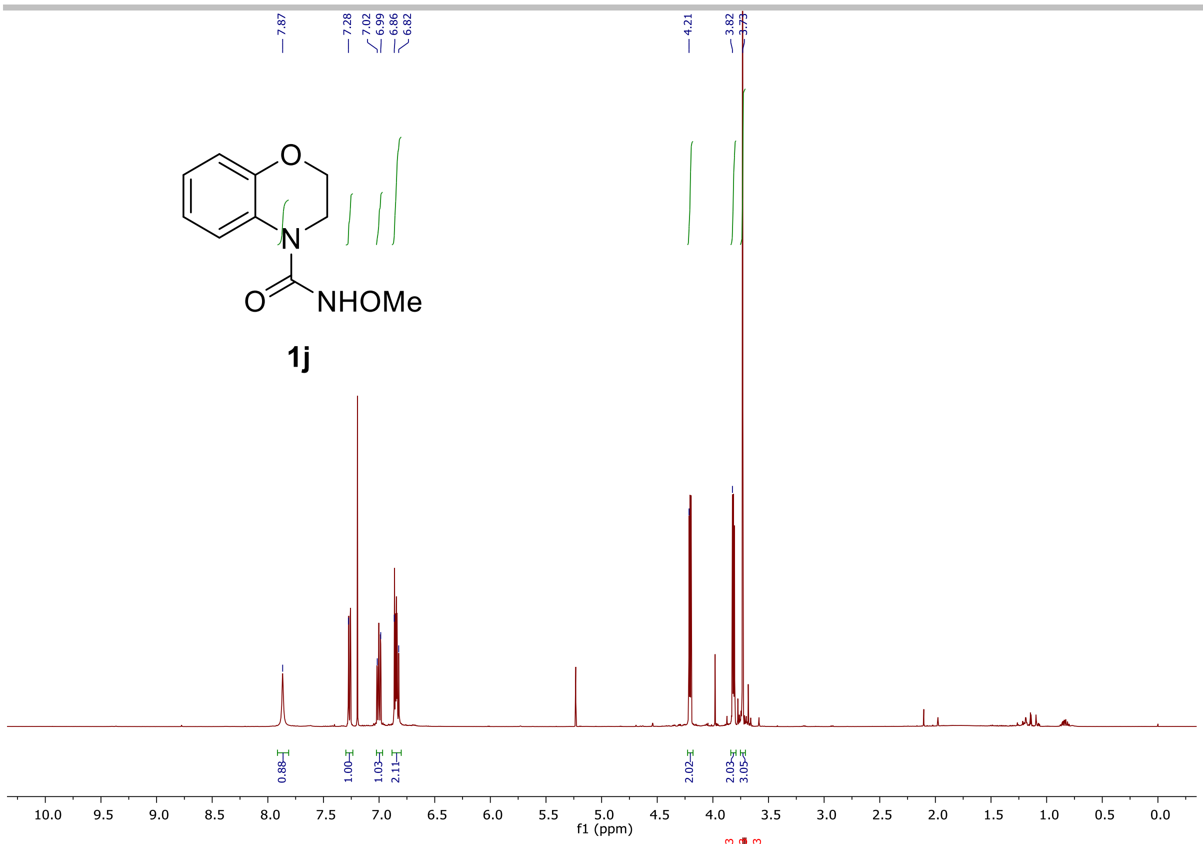
7.87
7.28
7.02
6.99
6.86
6.82
4.21
3.82
3.73
NHOMe
1j
0.88
1.00
1.03
2.11
2.02
2.03
3.05
f1 (ppm)

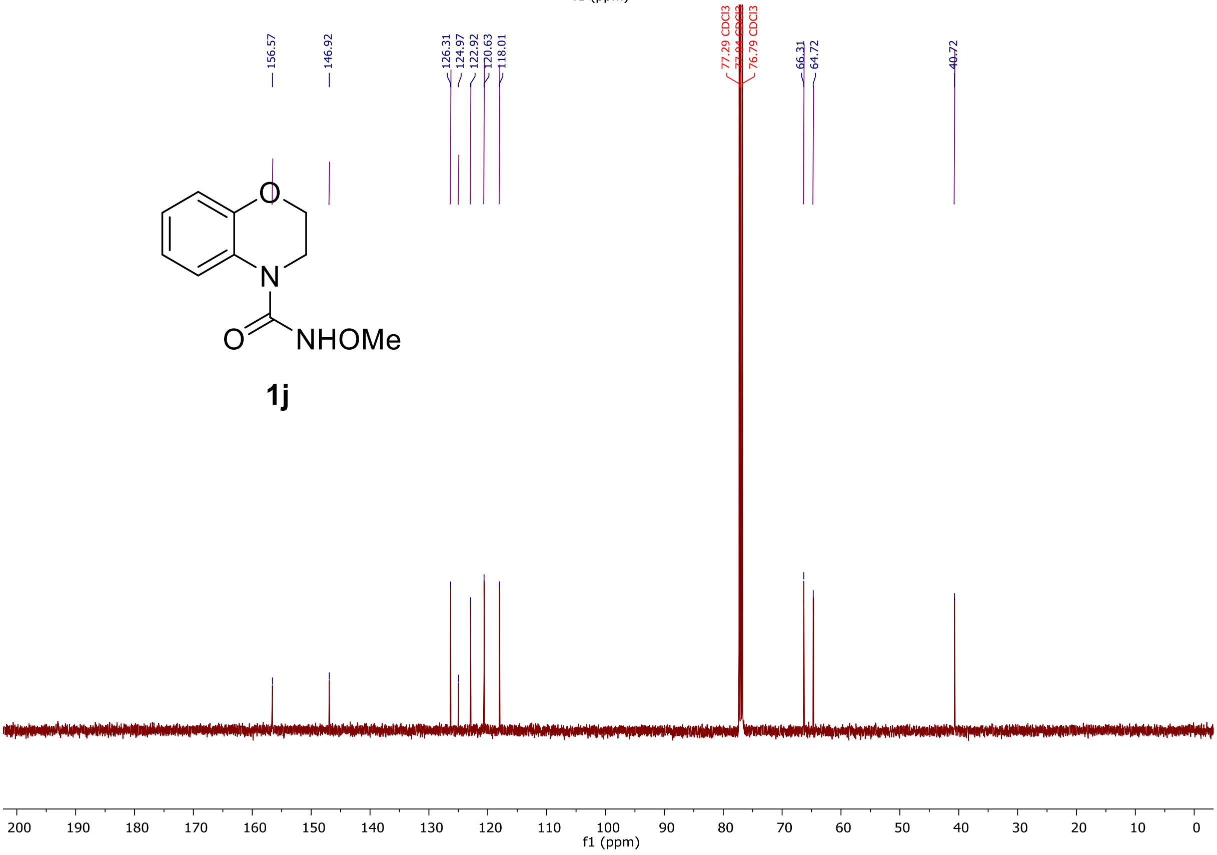
156.57
146.92
126.31
124.97
122.92
120.63
118.01
77.29 CDCl3
76.79 CDCl3
66.31
64.72
40.72
NHOMe
1j
f1 (ppm)

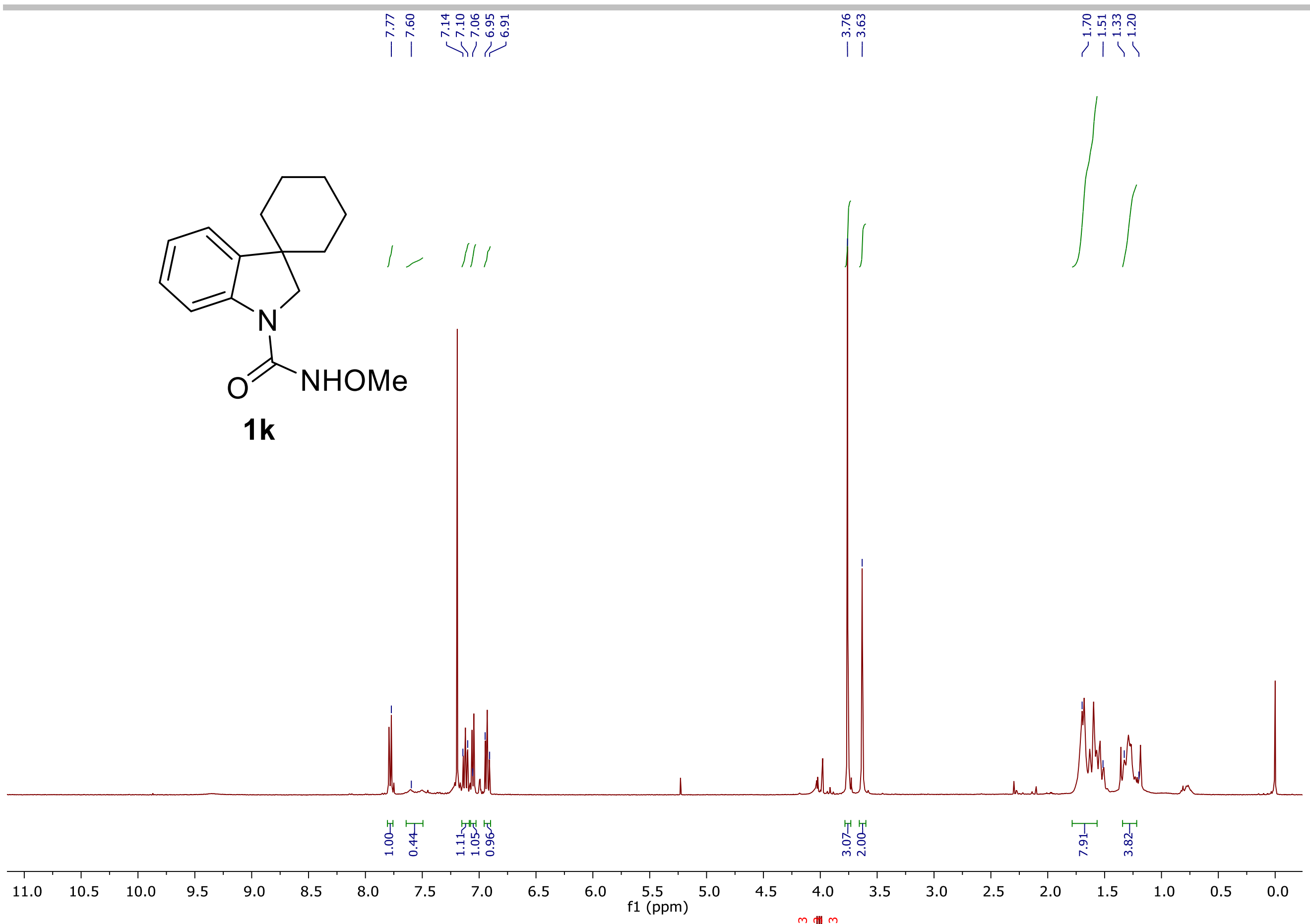
7.77
7.60
7.14
7.10
7.06
6.95
6.91
3.76
3.63
1.70
1.51
1.33
1.20
NHOMe
O
N
1k
1.00
0.44
1.11
1.05
0.96
3.07
2.00
7.91
3.82
11.0 10.5 10.0 9.5 9.0 8.5 8.0 7.5 7.0 6.5 6.0 5.5 5.0 4.5 4.0 3.5 3.0 2.5 2.0 1.5 1.0 0.5 0.0
f1 (ppm)

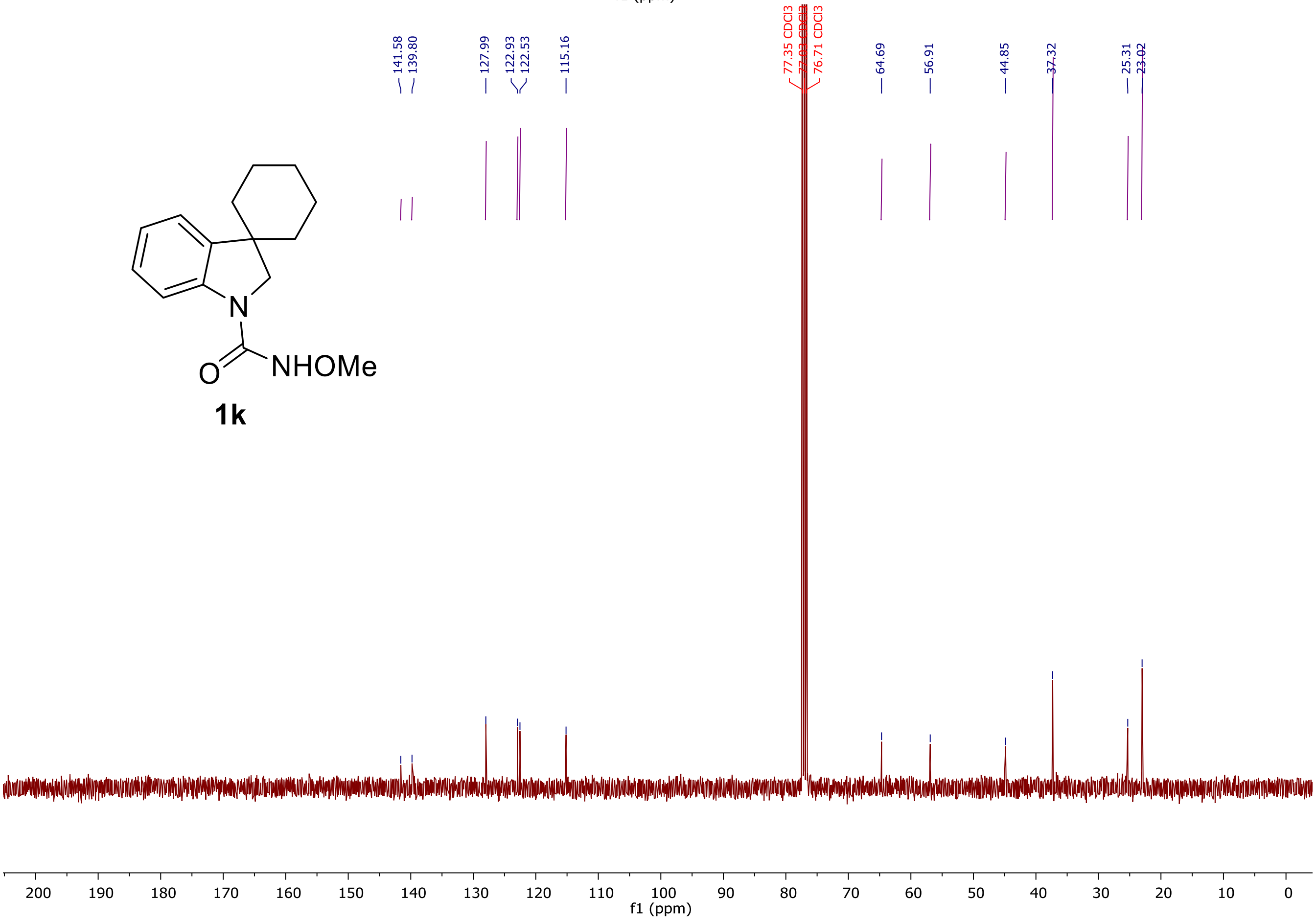
141.58
139.80
127.99
122.93
122.53
115.16
77.35 CDCl3
76.71 CDCl3
64.69
56.91
44.85
37.32
25.31
23.02
NHOMe
O
N
1k
200 190 180 170 160 150 140 130 120 110 100 90 80 70 60 50 40 30 20 10 0
f1 (ppm)

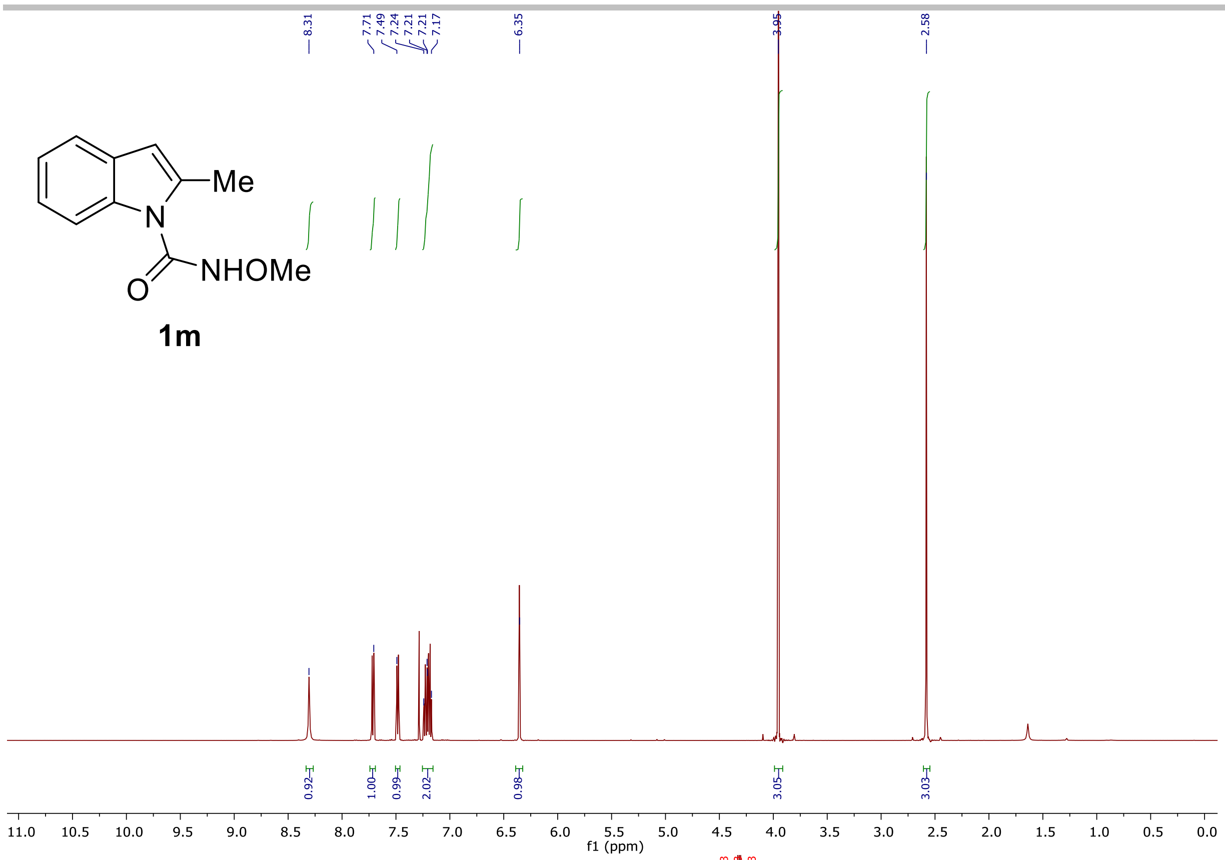

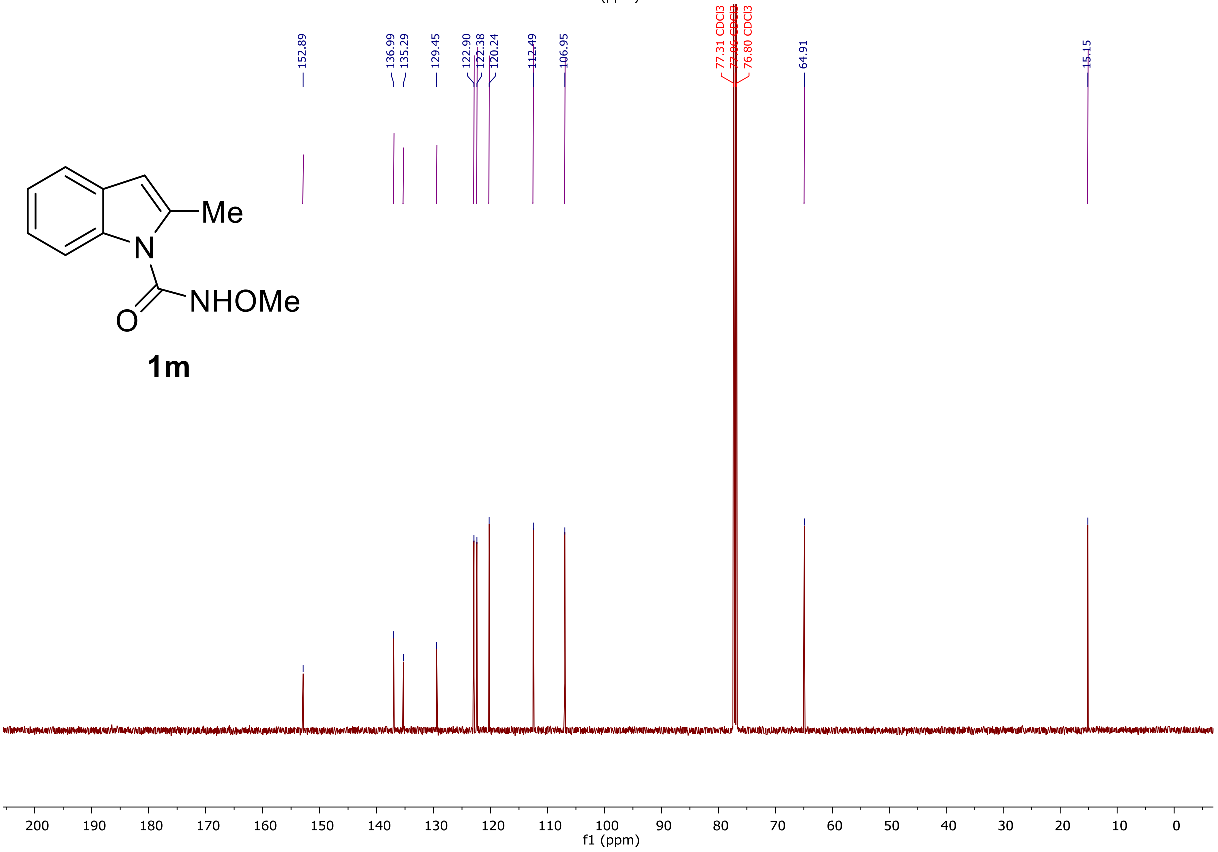

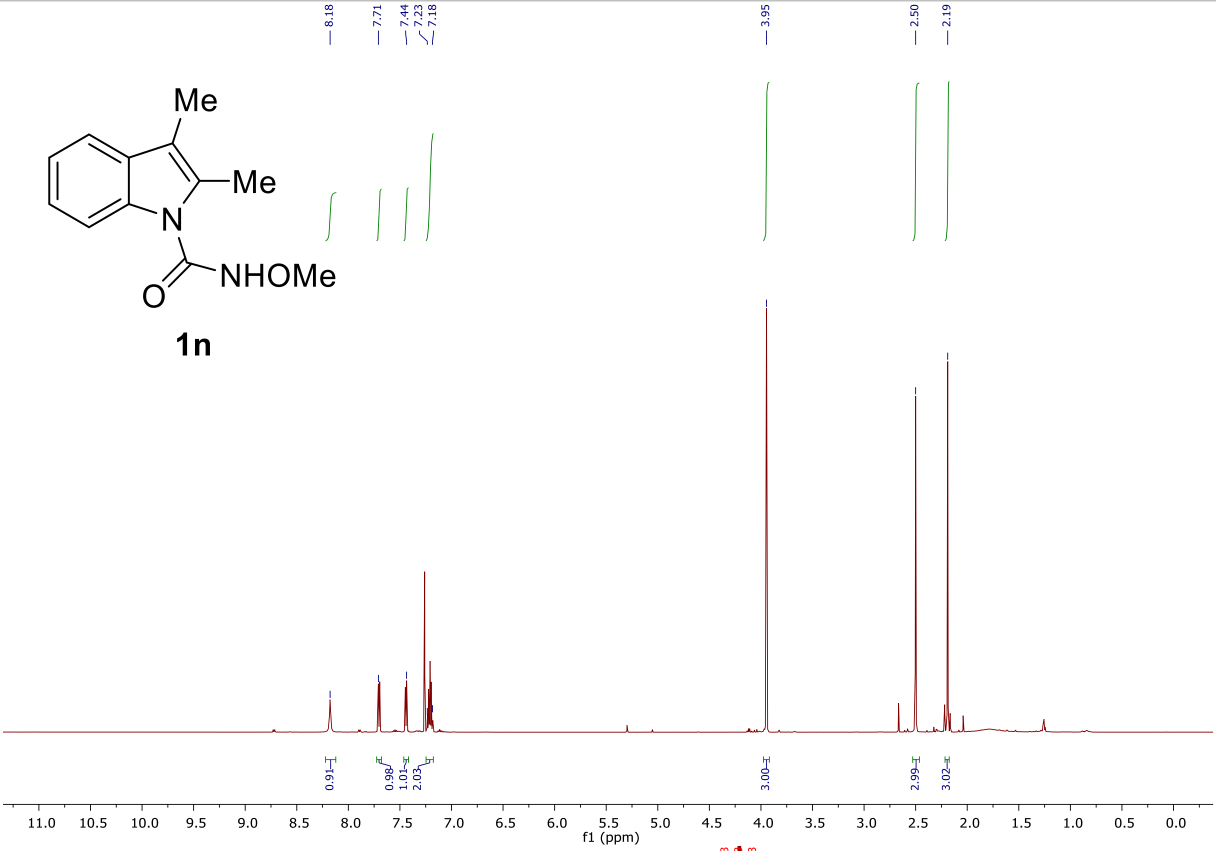
8.18
7.71
7.44
7.23
7.18
3.95
2.50
2.19
Me
Me
N
O
NHOMe
1n
0.91
0.98
1.01
2.03
3.00
2.99
3.02
11.0
10.5
10.0
9.5
9.0
8.5
8.0
7.5
7.0
6.5
6.0
5.5
5.0
4.5
4.0
3.5
3.0
2.5
2.0
1.5
1.0
0.5
0.0
f1 (ppm)

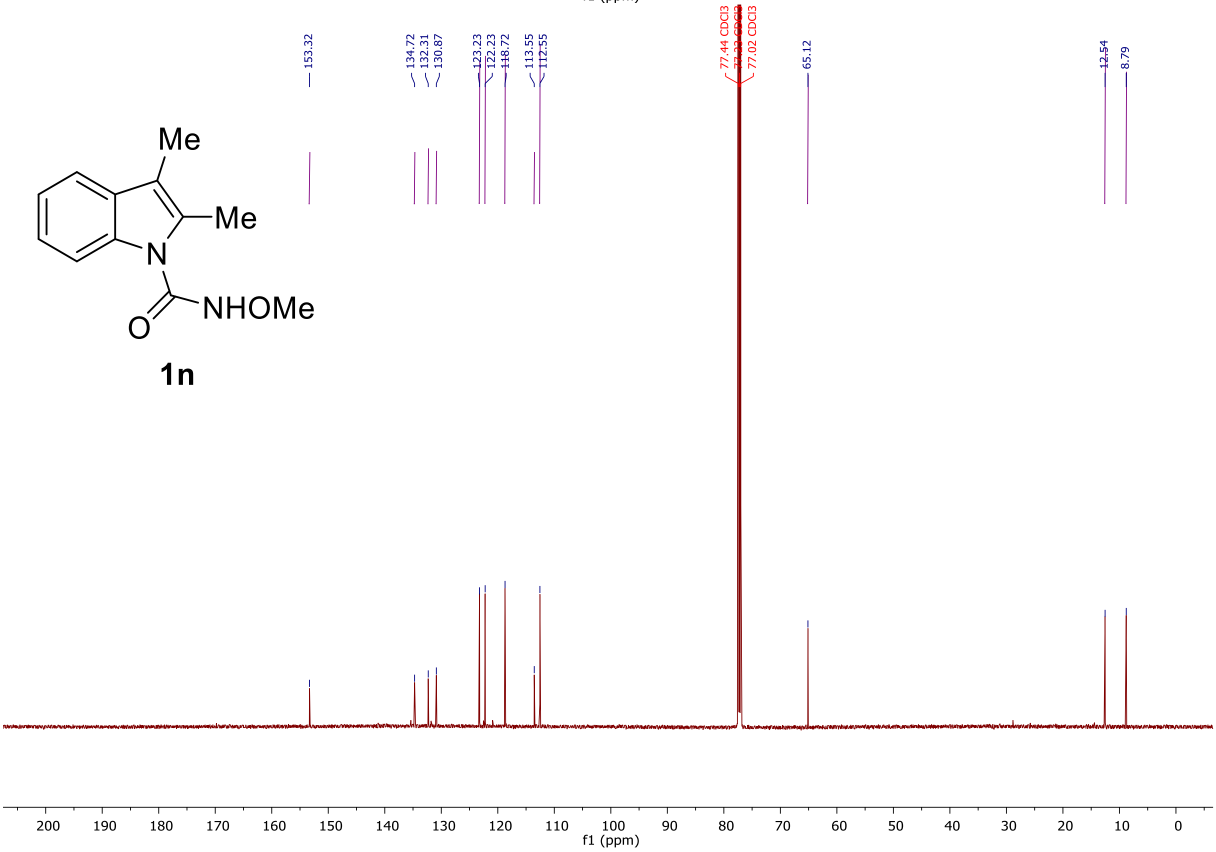
153.32
134.72
132.31
130.87
123.23
122.23
118.72
113.55
112.55
77.44 CDCl3
77.02 CDCl3
65.12
12.54
8.79
Me
Me
N
O
NHOMe
1n
200
190
180
170
160
150
140
130
120
110
100
90
80
70
60
50
40
30
20
10
0
f1 (ppm)

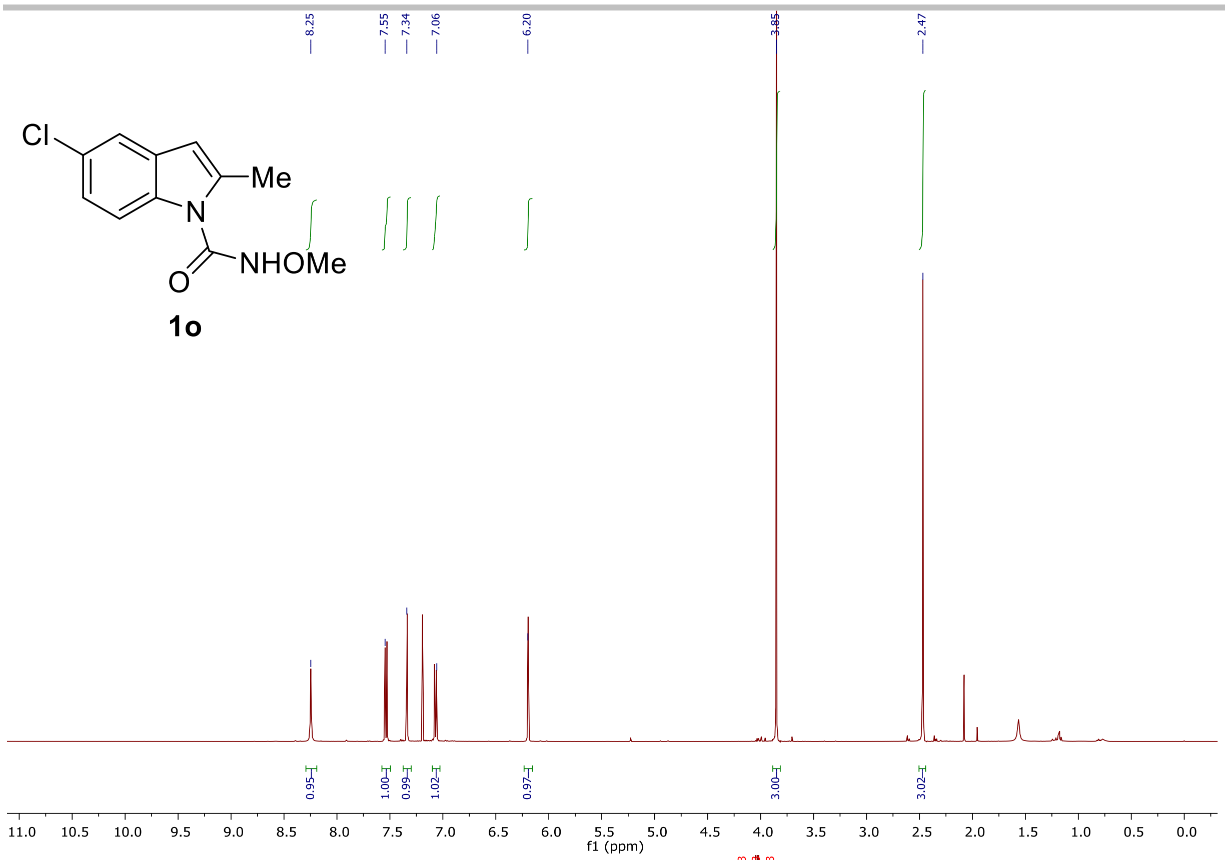
8.25
7.55
7.34
7.06
6.20
3.85
2.47
Cl
Me
N
O
NHOMe
1o
0.95
1.00
0.99
1.02
0.97
3.00
3.02
11.0
10.5
10.0
9.5
9.0
8.5
8.0
7.5
7.0
6.5
6.0
5.5
5.0
4.5
4.0
3.5
3.0
2.5
2.0
1.5
1.0
0.5
0.0
f1 (ppm)

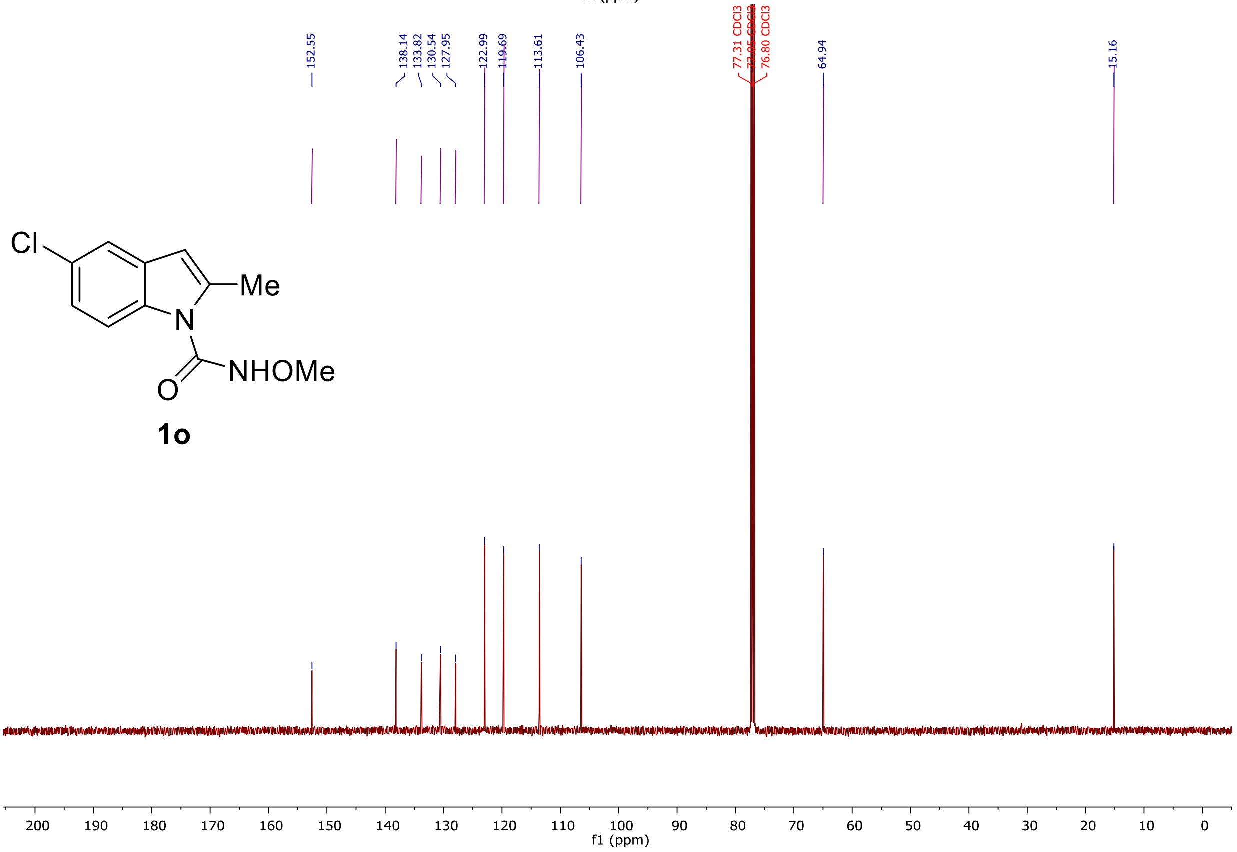
152.55
138.14
133.82
130.54
127.95
122.99
119.69
113.61
106.43
77.31 CDCl3
76.80 CDCl3
64.94
15.16
Cl
Me
N
O
NHOMe
1o
200
190
180
170
160
150
140
130
120
110
100
90
80
70
60
50
40
30
20
10
0
f1 (ppm)

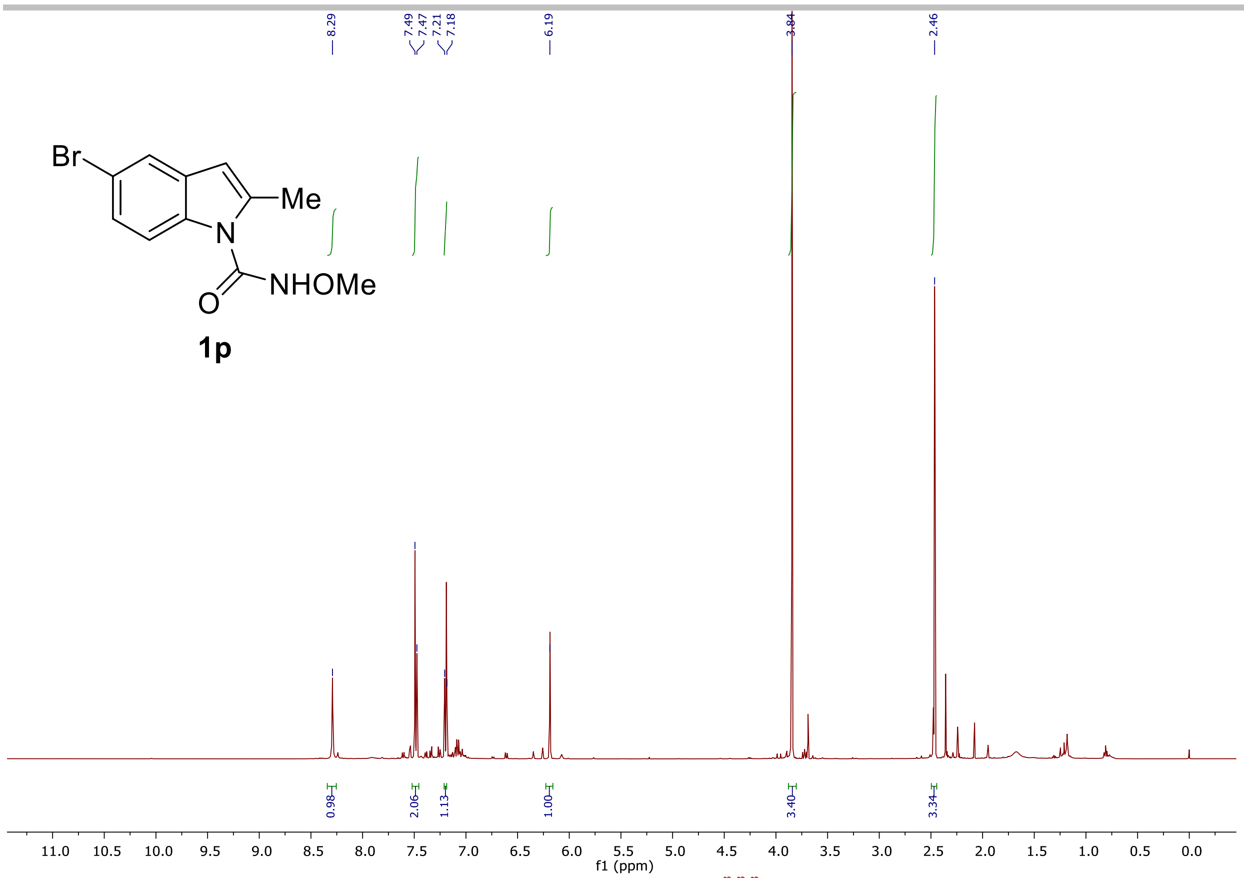
8.29
7.49
7.47
7.21
7.18
6.19
3.84
2.46
Br
Me
N
O
NHOMe
1p
0.98
2.06
1.13
1.00
3.40
3.34
f1 (ppm)

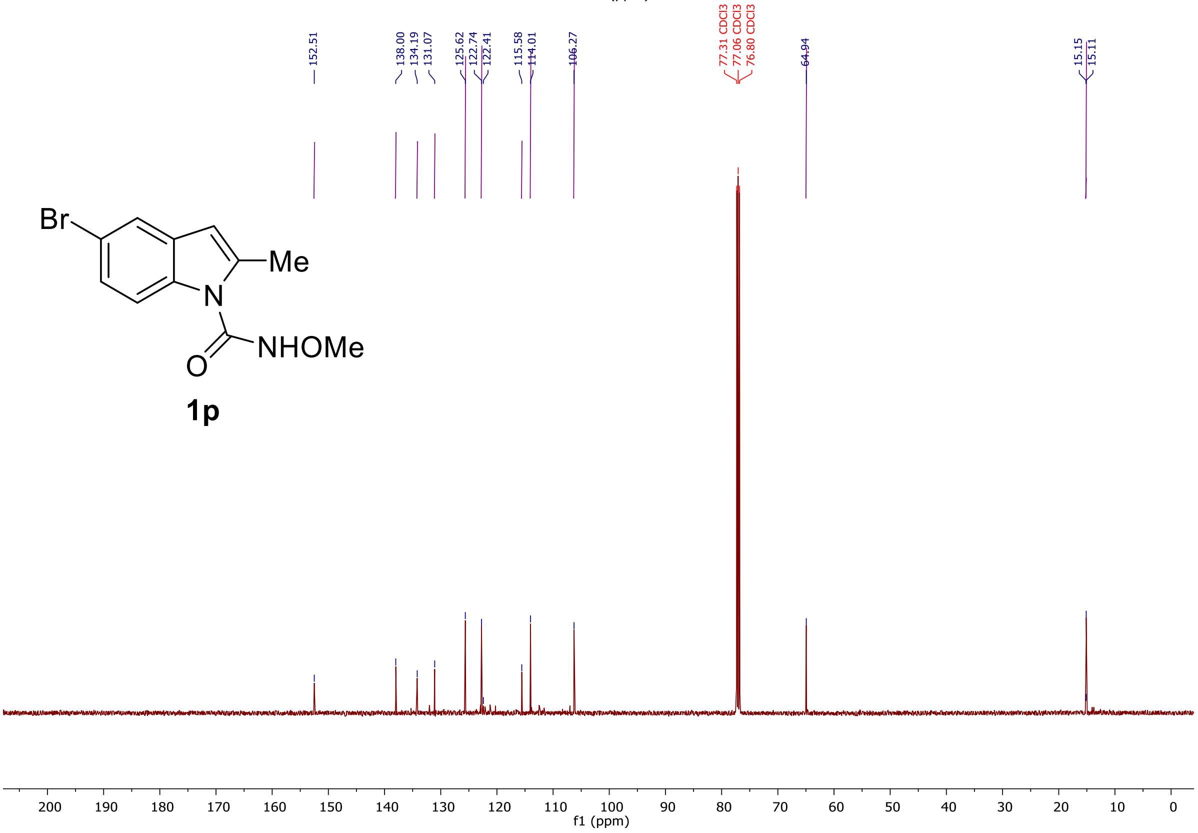
152.51
138.00
134.19
131.07
125.62
122.74
122.41
115.58
114.01
106.27
77.31 CDCl3
77.06 CDCl3
76.80 CDCl3
64.94
15.15
15.11
Br
Me
N
O
NHOMe
1p
f1 (ppm)

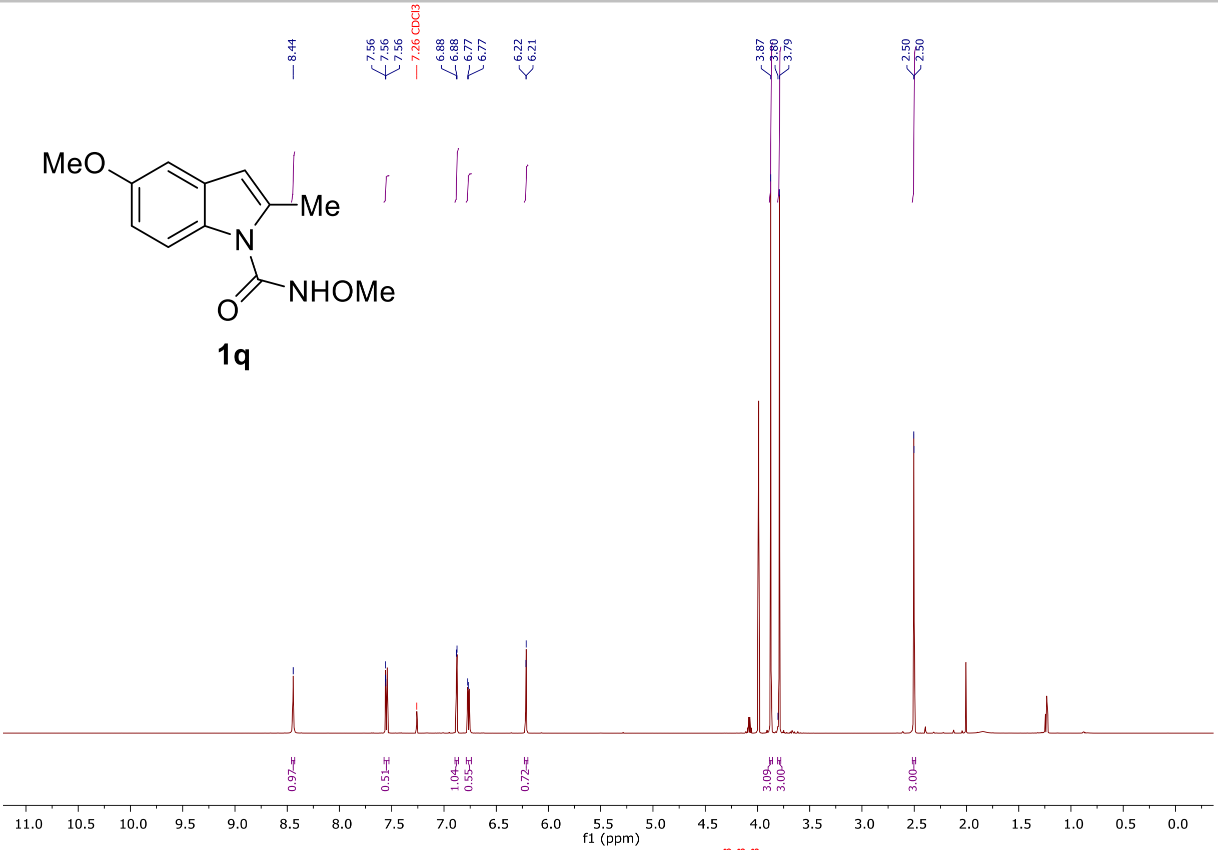
8.44
7.56
7.56
7.56
7.26 CDCl3
6.88
6.88
6.77
6.77
6.22
6.21
3.87
3.80
3.79
2.50
2.50
MeO
Me
N
O
NHOMe
1q
0.97
0.51
1.04
0.55
0.72
3.09
3.00
3.00
11.0
10.5
10.0
9.5
9.0
8.5
8.0
7.5
7.0
6.5
6.0
5.5
5.0
4.5
4.0
3.5
3.0
2.5
2.0
1.5
1.0
0.5
0.0
f1 (ppm)

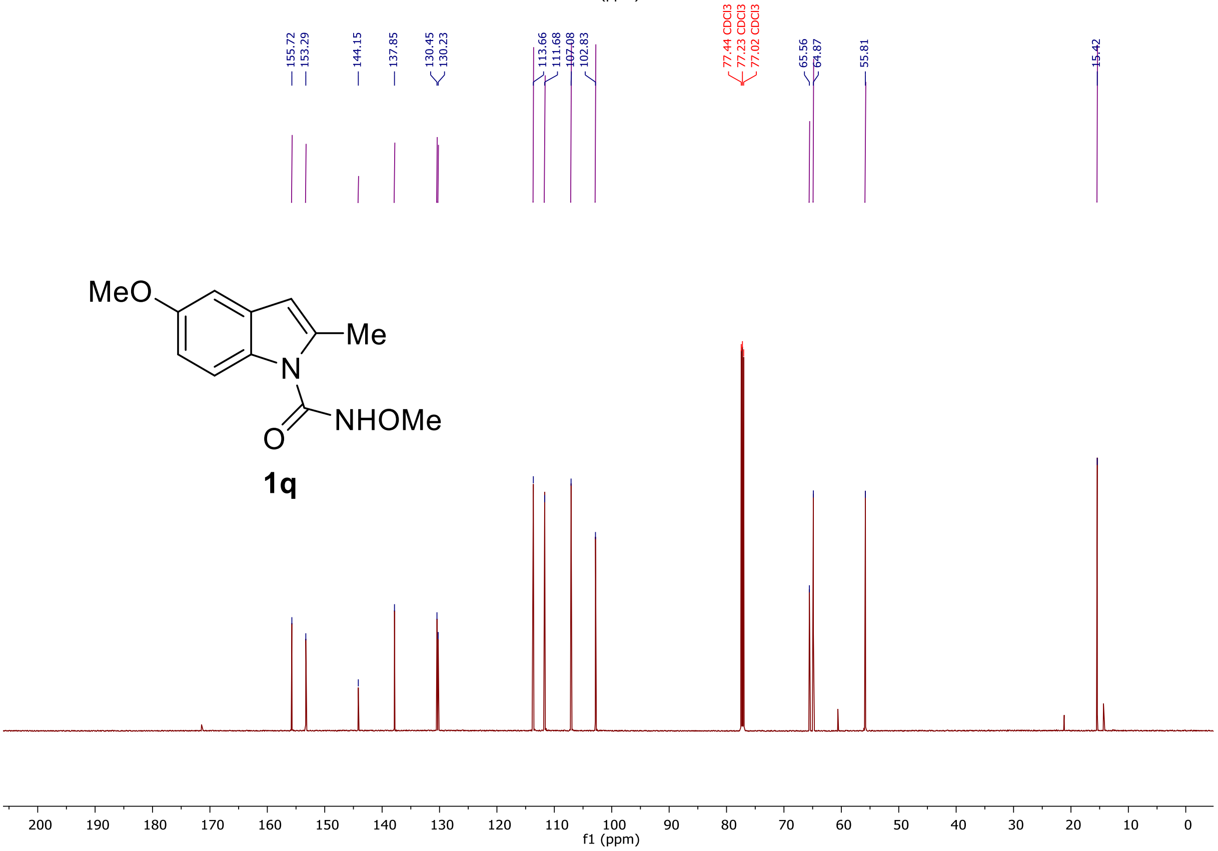
155.72
153.29
144.15
137.85
130.45
130.23
113.66
111.68
107.08
102.83
77.44 CDCl3
77.23 CDCl3
77.02 CDCl3
65.56
64.87
55.81
15.42
MeO
Me
N
O
NHOMe
1q
200
190
180
170
160
150
140
130
120
110
100
90
80
70
60
50
40
30
20
10
0
f1 (ppm)

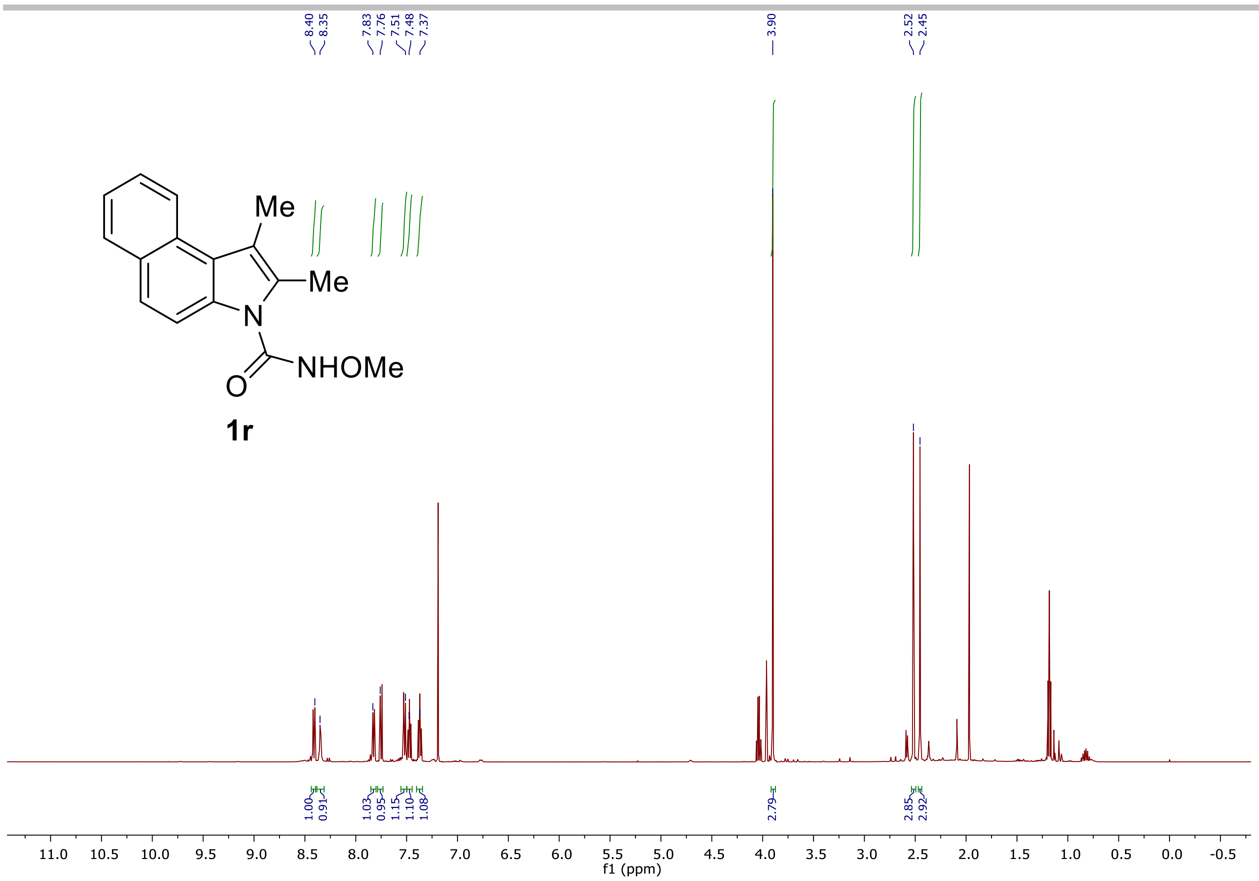
8.40
8.35
7.83
7.76
7.51
7.48
7.37
3.90
2.52
2.45
Me
Me
N
NHOMe
O
1r
1.00
0.91
1.03
0.95
1.15
1.10
1.08
2.79
2.85
2.92
f1 (ppm)

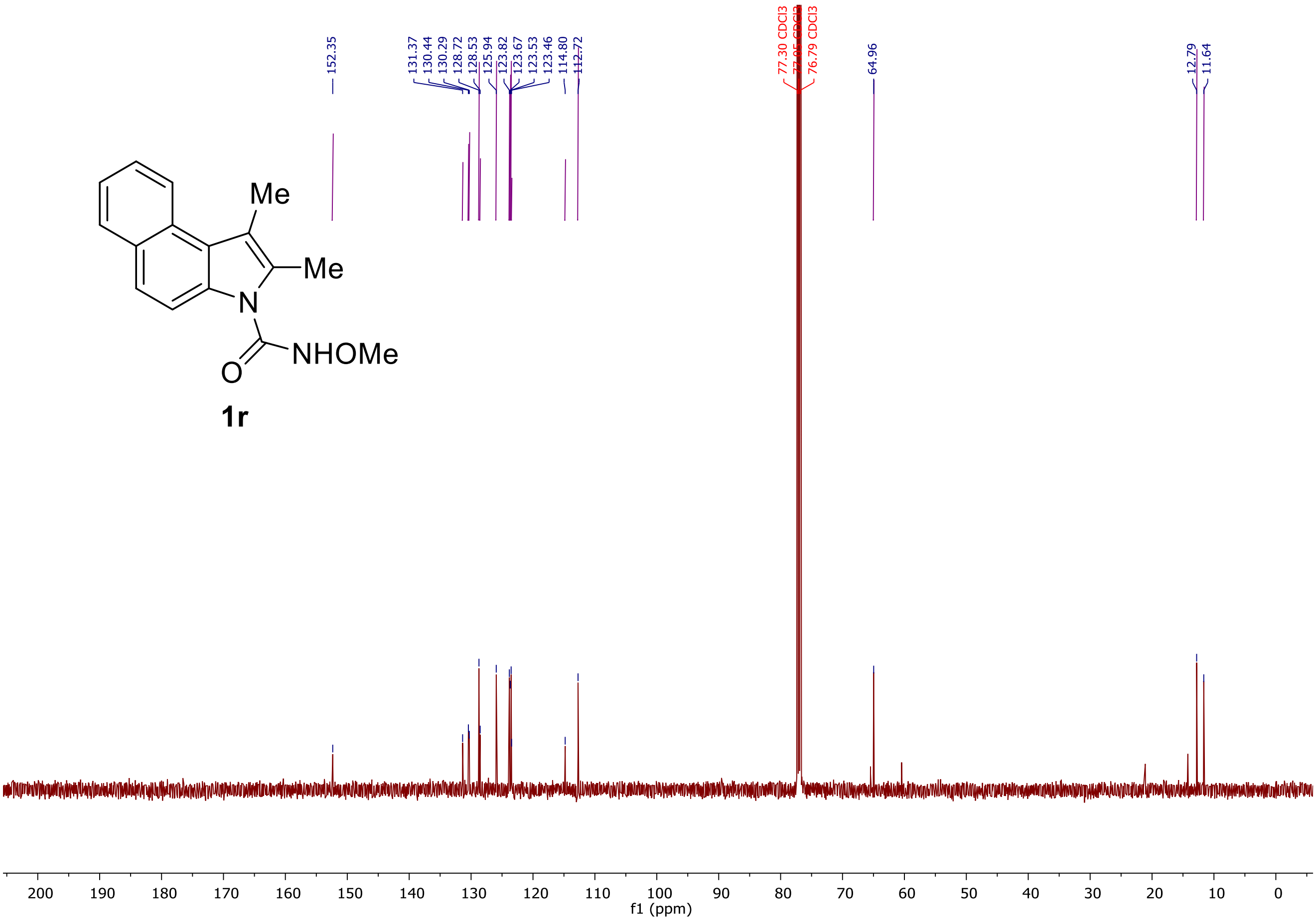
152.35
131.37
130.44
130.29
128.72
128.53
125.94
123.82
123.67
123.53
123.46
114.80
112.72
77.30 CDCl3
76.79 CDCl3
64.96
12.79
11.64
Me
Me
N
NHOMe
O
1r
f1 (ppm)

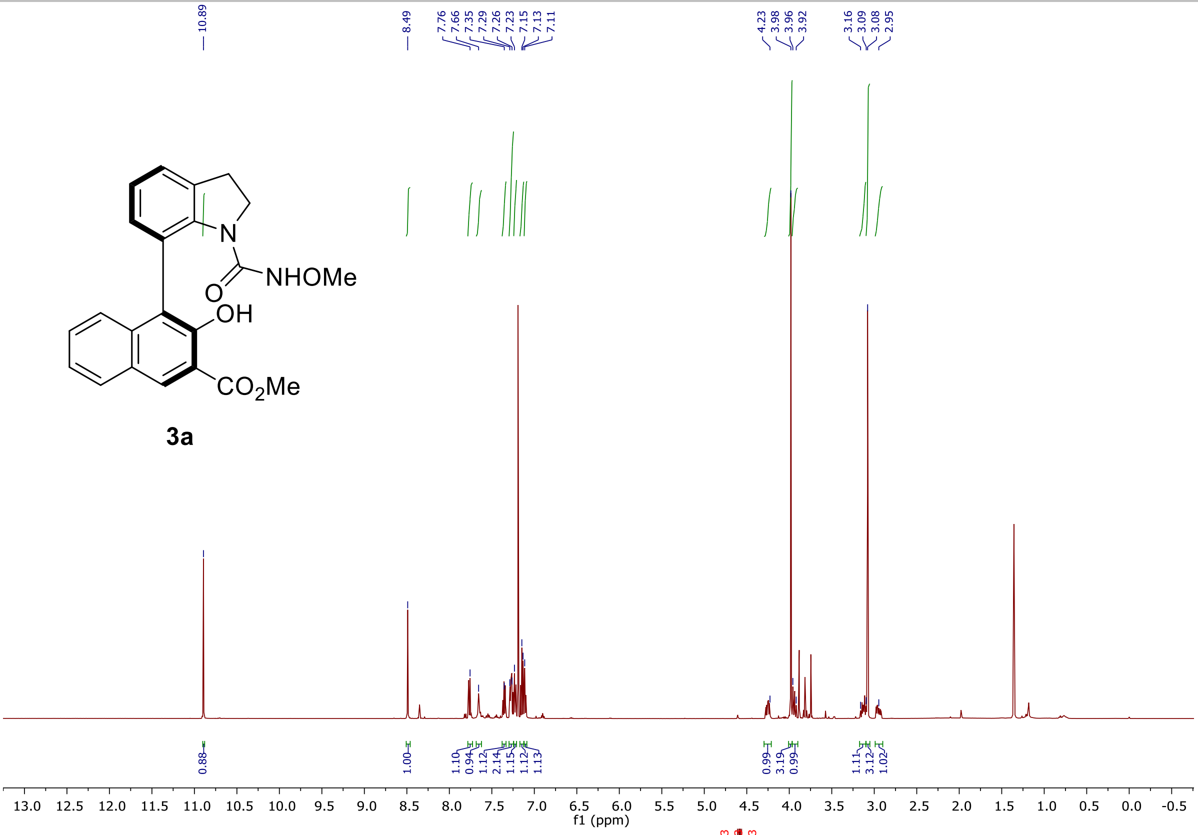

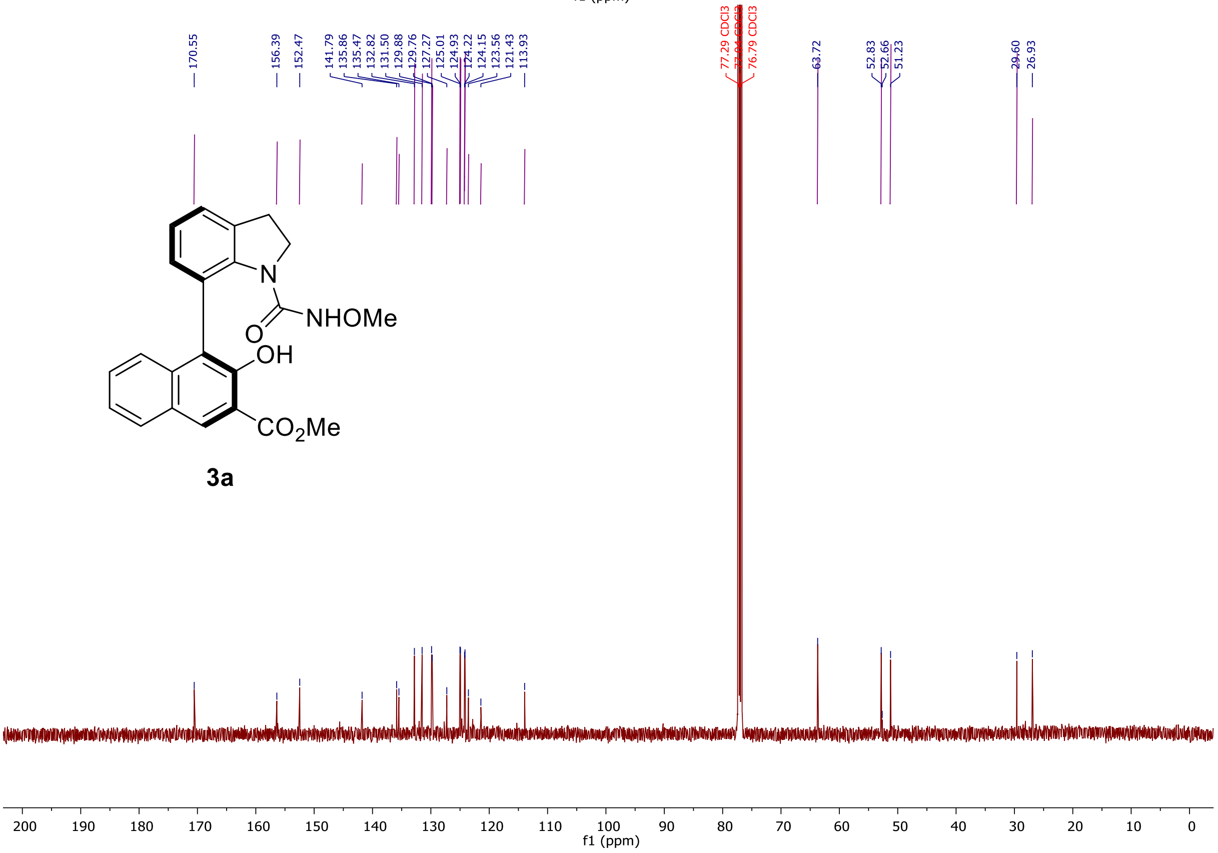

HPLC traces (**3a**): racemate top, enantiomer bottom:

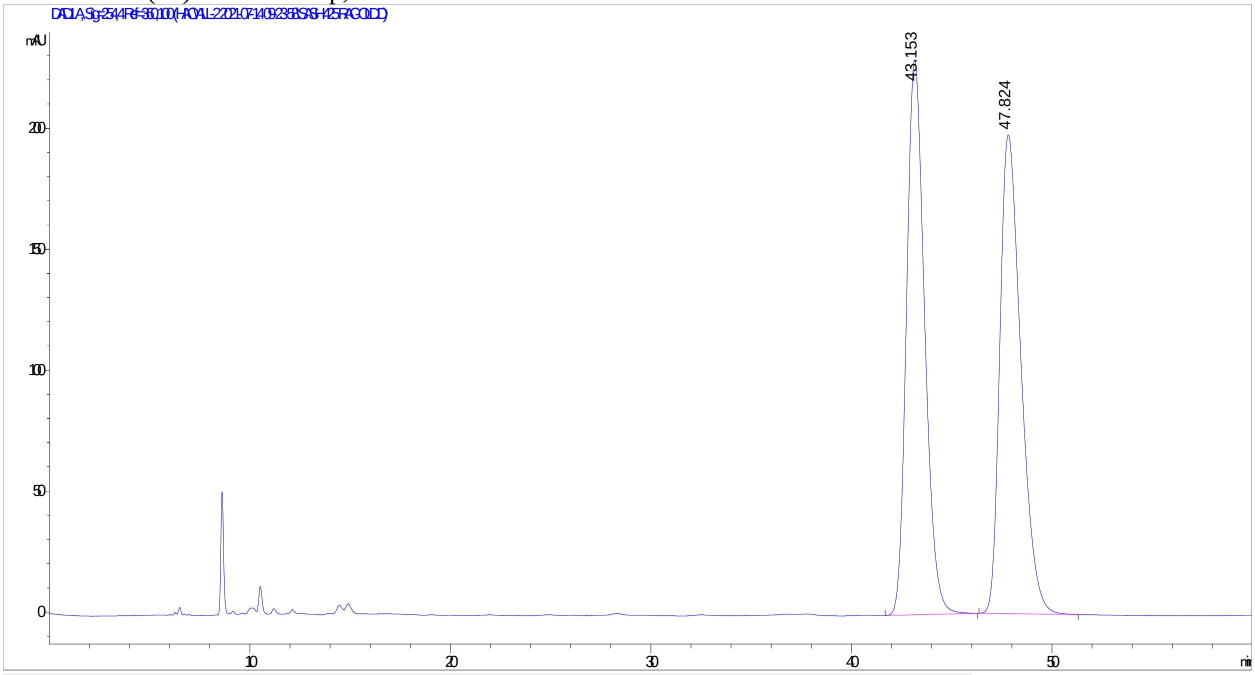

#	Time	Area	Height	Width	Area%	Symmetry
1	43.153	14406.4	229.4	0.9808	49.984	0.767
2	47.824	14415.8	197.9	1.1157	50.016	0.627

(*S*) enantiomer

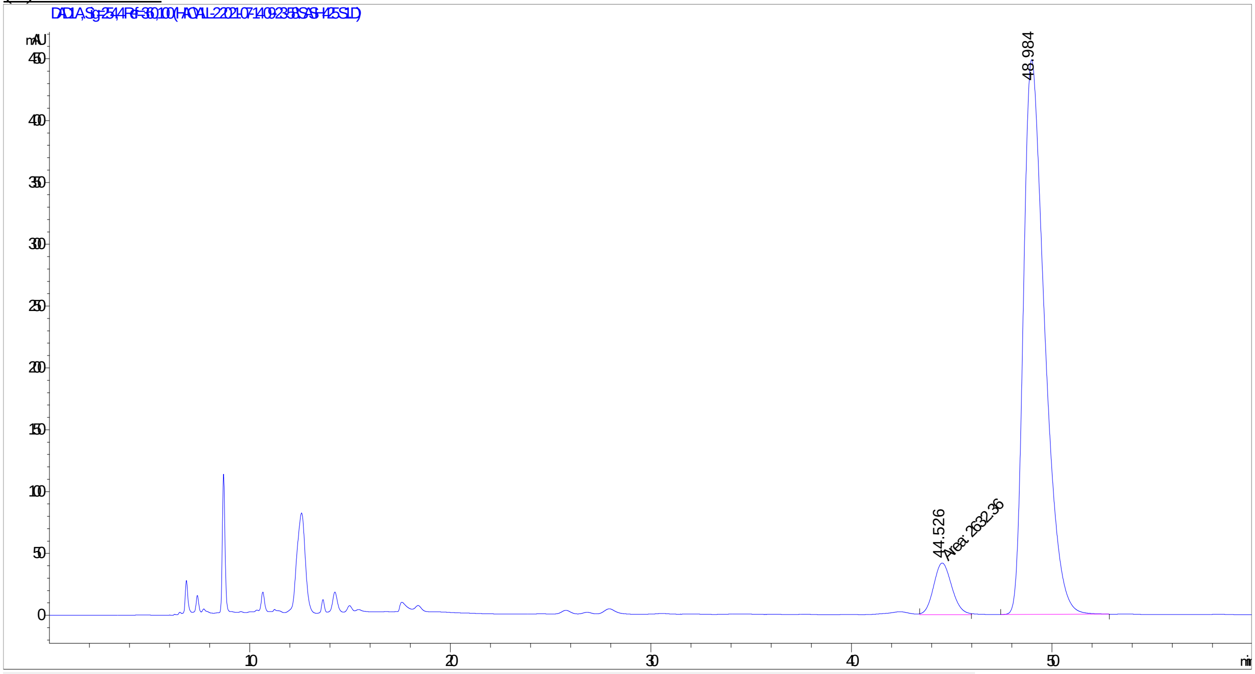

#	Time	Area	Height	Width	Area%	Symmetry
1	44.526	2684.2	42.2	1.06	7.538	0.868
2	48.984	32923.6	449.2	1.2215	92.462	0.603

(*R*) enantiomer

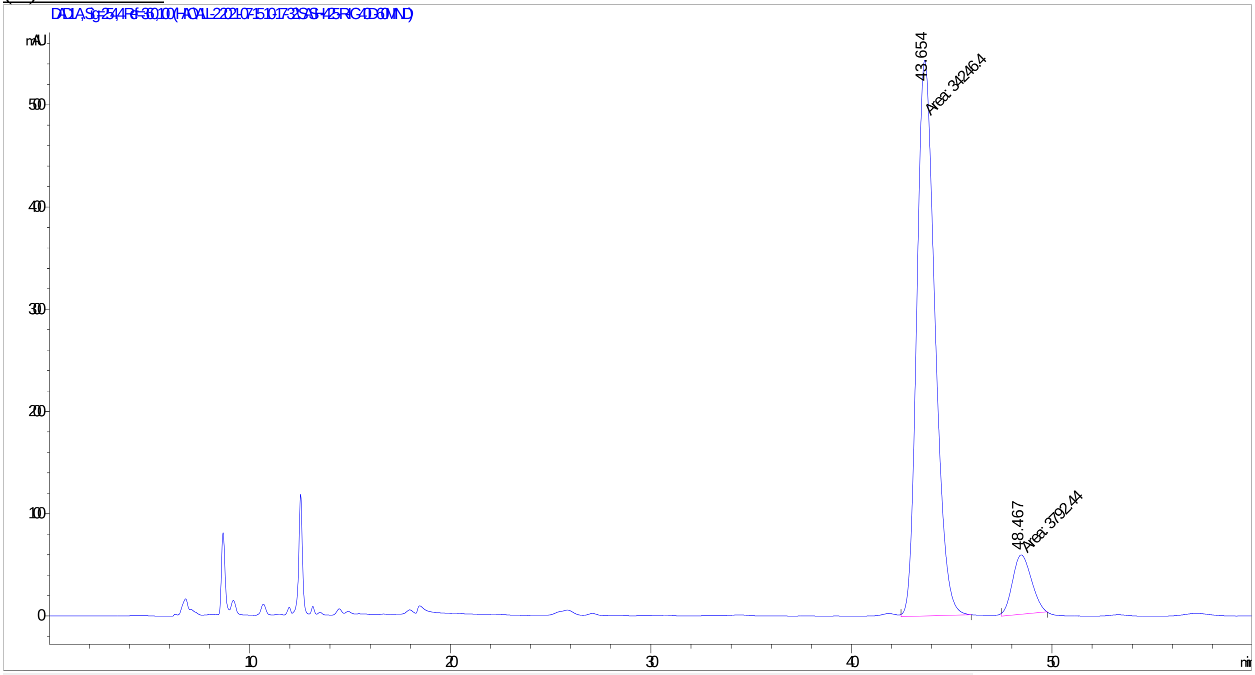

#	Time	Area	Height	Width	Area%	Symmetry
1	43.654	34263.7	543.7	1.0503	90.344	0.746
2	48.467	3662.3	57	1.0704	9.656	0.846

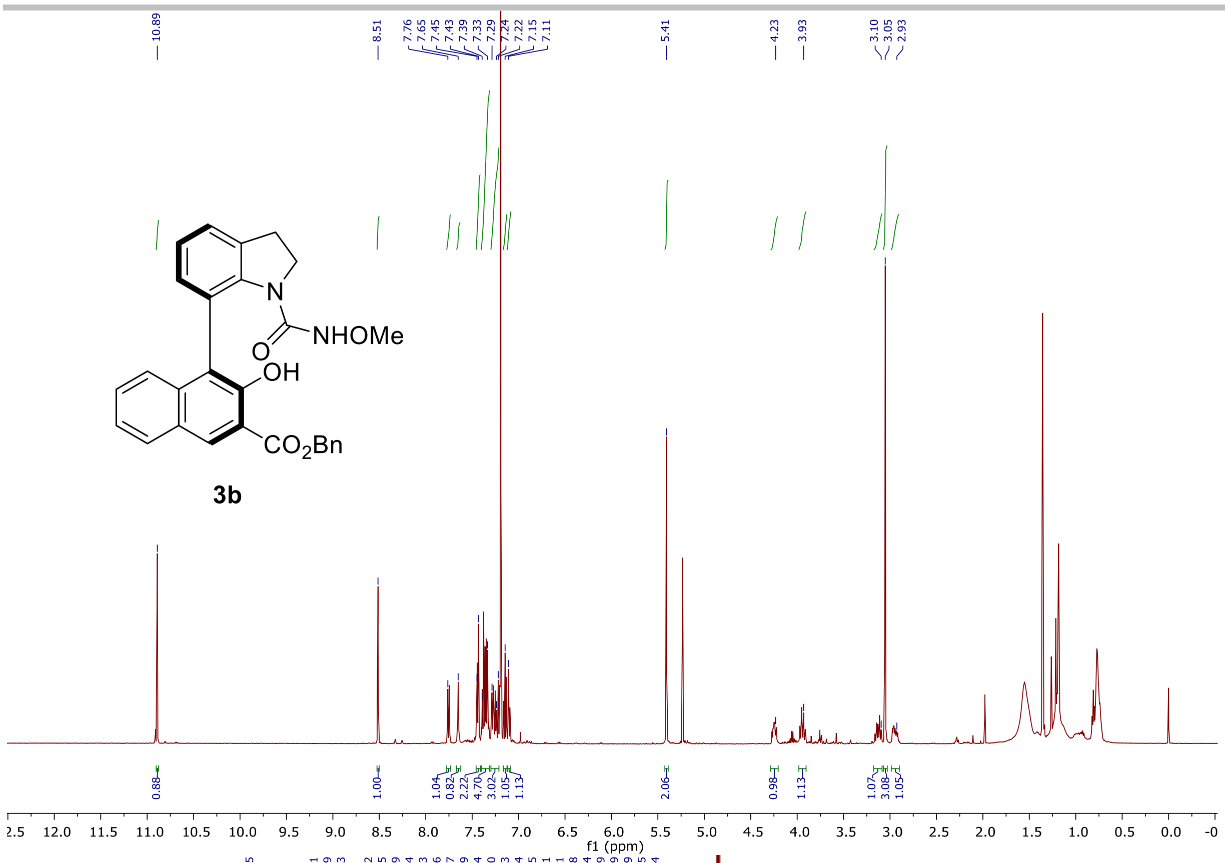
10.89
8.51
7.76
7.65
7.45
7.43
7.39
7.33
7.29
7.24
7.22
7.15
7.11
5.41
4.23
3.93
3.10
3.05
2.93
NHOMe
N
O
OH
CO2Bn
3b
0.88
1.00
1.04
0.82
2.22
4.70
3.02
1.05
1.13
2.06
0.98
1.13
1.07
3.08
1.05
f1 (ppm)

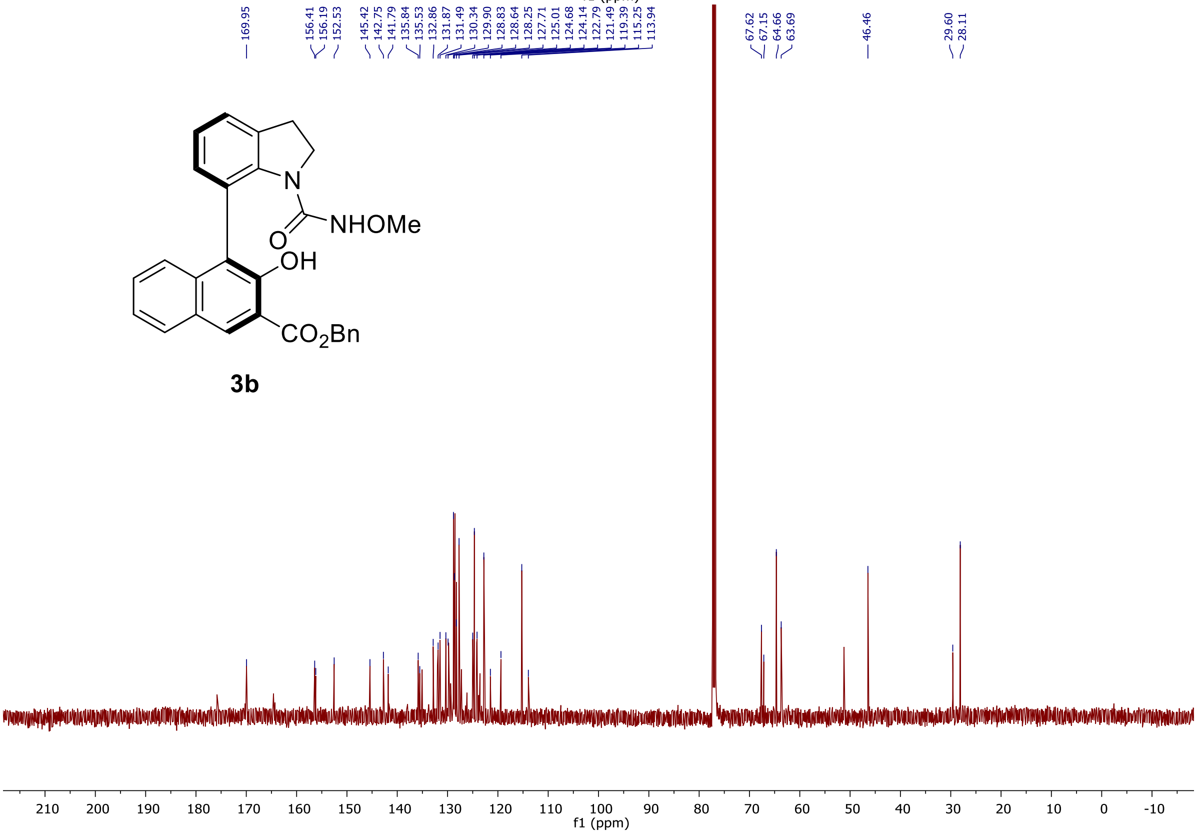
169.95
156.41
156.19
152.53
145.42
142.75
141.79
135.84
135.53
132.86
131.87
131.49
130.34
129.90
128.83
128.64
128.25
127.71
125.01
124.68
124.14
122.79
121.49
119.39
115.25
113.94
67.62
67.15
64.66
63.69
46.46
29.60
28.11
NHOMe
N
O
OH
CO2Bn
3b
f1 (ppm)

HPLC traces (**3b**): racemate top, enantiomer bottom:

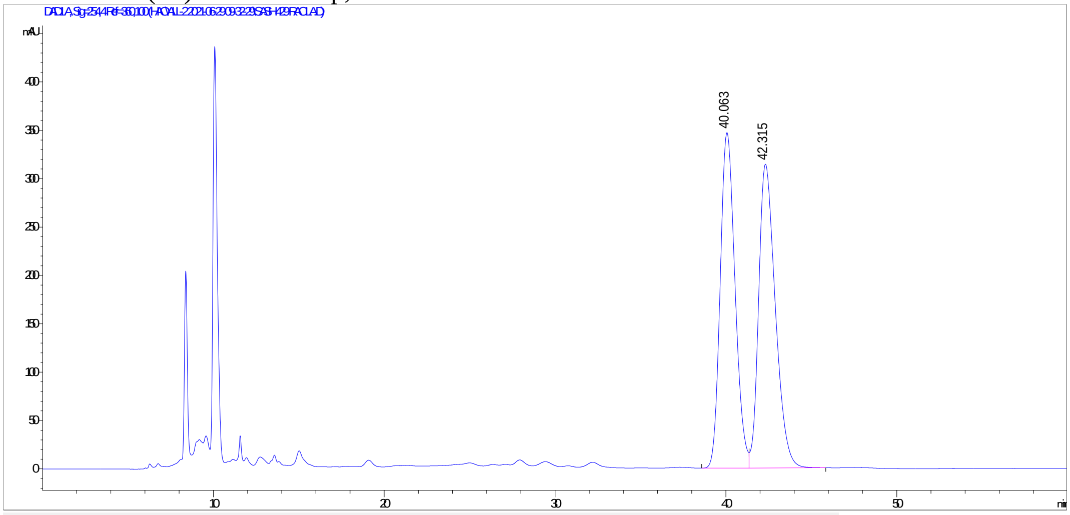

#	Time	Area	Height	Width	Area%	Symmetry
1	40.063	20926.4	346.7	0.9362	49.561	0.797
2	42.315	21297	314	1.0372	50.439	0.651

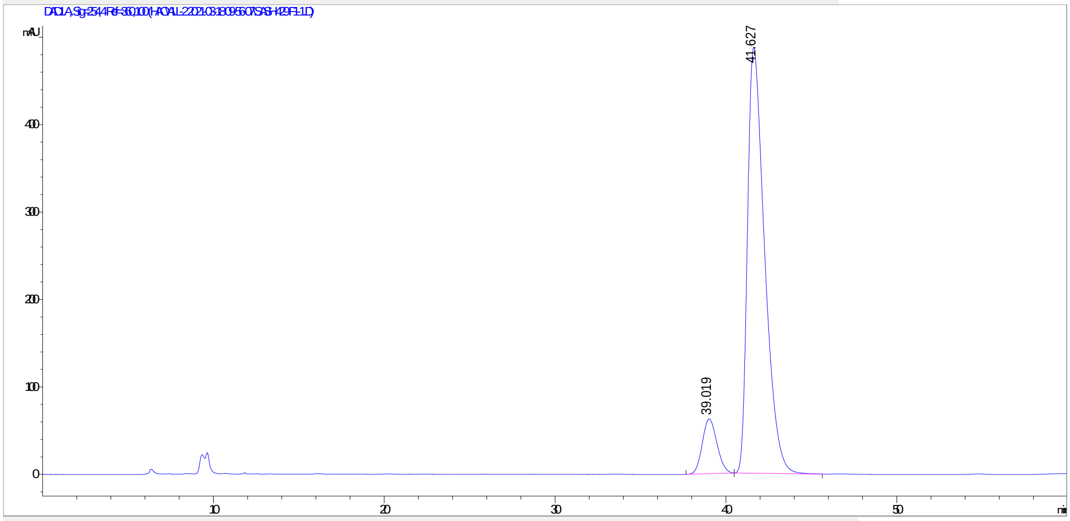

#	Time	Area	Height	Width	Area%	Symmetry
1	39.019	3742.2	62.8	0.9274	9.942	0.858
2	41.627	33900.3	487.4	1.0535	90.058	0.535

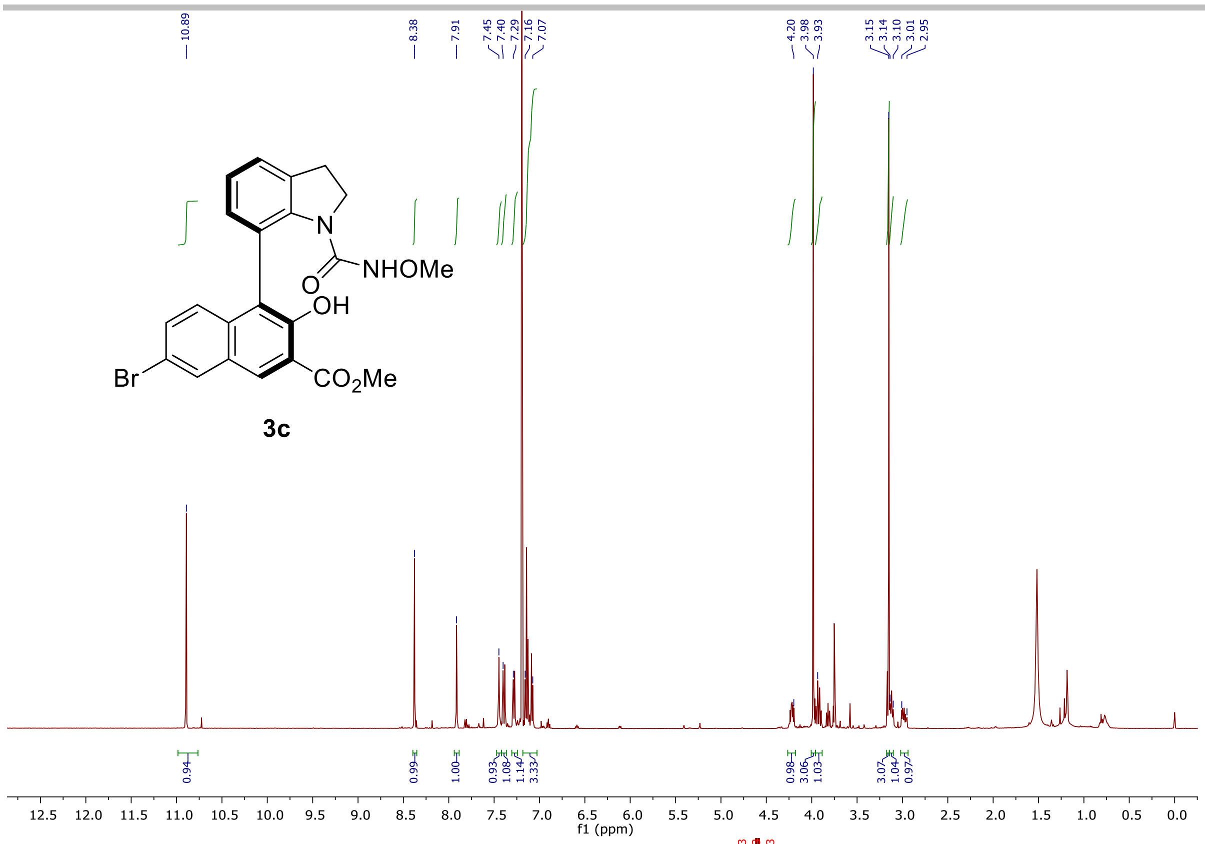
10.89
8.38
7.91
7.45
7.40
7.29
7.16
7.07
4.20
3.98
3.93
3.15
3.14
3.10
3.01
2.95
NHOMe
OH
Br
CO2Me
3c
0.94
0.99
1.00
0.93
1.08
1.14
3.33
0.98
3.06
1.03
3.07
1.04
0.97
12.5 12.0 11.5 11.0 10.5 10.0 9.5 9.0 8.5 8.0 7.5 7.0 6.5 6.0 5.5 5.0 4.5 4.0 3.5 3.0 2.5 2.0 1.5 1.0 0.5 0.0
f1 (ppm)

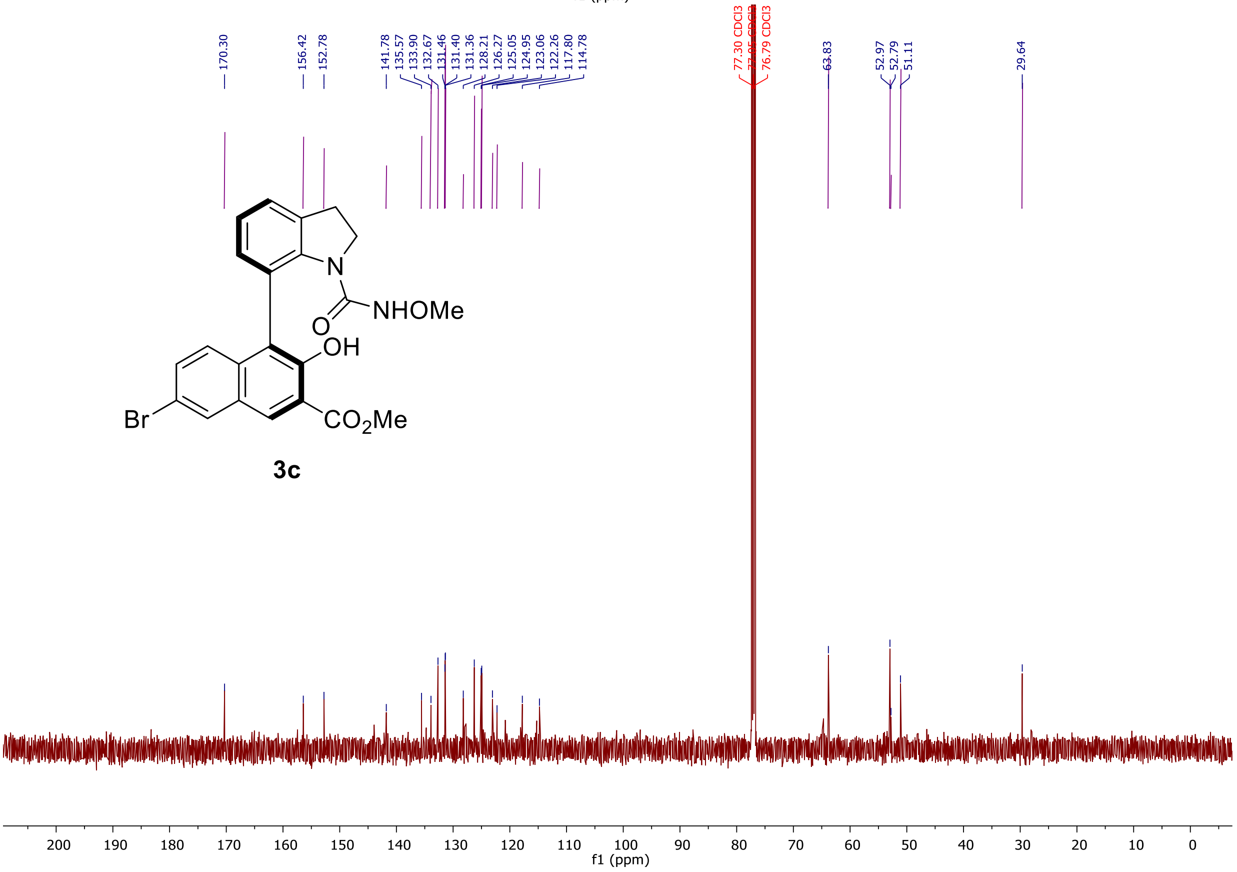
170.30
156.42
152.78
141.78
135.57
133.90
132.67
131.46
131.40
131.36
128.21
126.27
125.05
124.95
123.06
122.26
117.80
114.78
77.30 CDCl3
76.79 CDCl3
63.83
52.97
52.79
51.11
29.64
NHOMe
OH
Br
CO2Me
3c
200 190 180 170 160 150 140 130 120 110 100 90 80 70 60 50 40 30 20 10 0
f1 (ppm)

HPLC traces (**3c**): racemate top, enantiomer bottom:

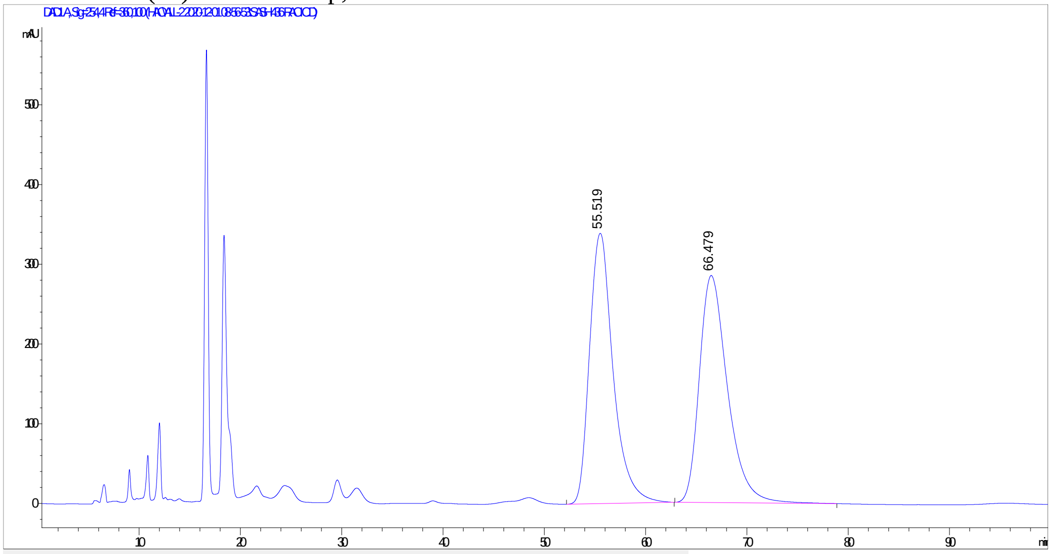

#	Time	Area	Height	Width	Area%	Symmetry
1	55.519	54805.4	339	2.2902	49.955	0.757
2	66.479	54904.6	284.9	2.7865	50.045	0.677

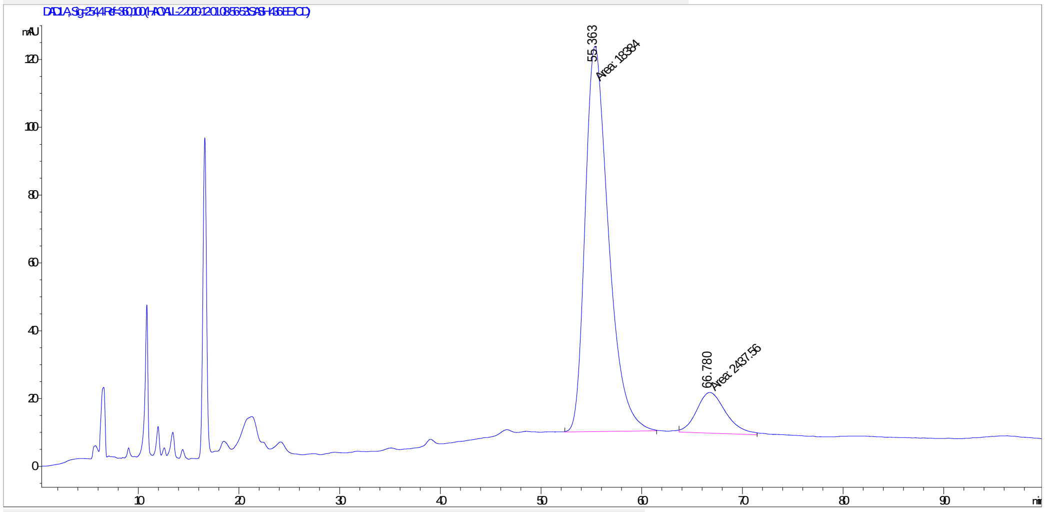

#	Time	Area	Height	Width	Area%	Symmetry
1	55.363	18361.7	113.5	2.697	89.503	0.685
2	66.78	2153.6	11.4	3.1489	10.497	0.785

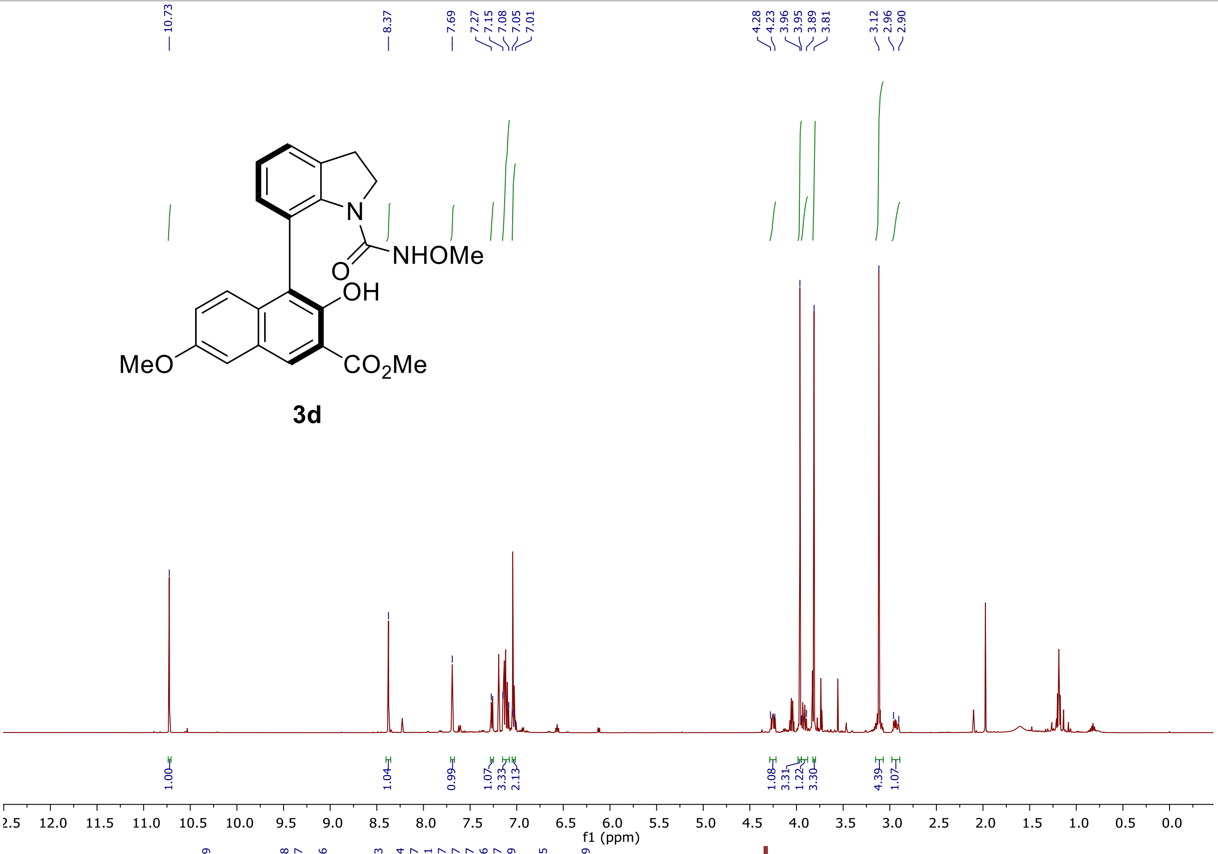
10.73
8.37
7.69
7.27
7.15
7.08
7.05
7.01
4.28
4.23
3.96
3.95
3.89
3.81
3.12
2.96
2.90
NHOMe
OH
MeO
CO2Me
3d
1.00
1.04
0.99
1.07
3.33
2.13
1.08
3.31
1.22
3.30
4.39
1.07
f1 (ppm)

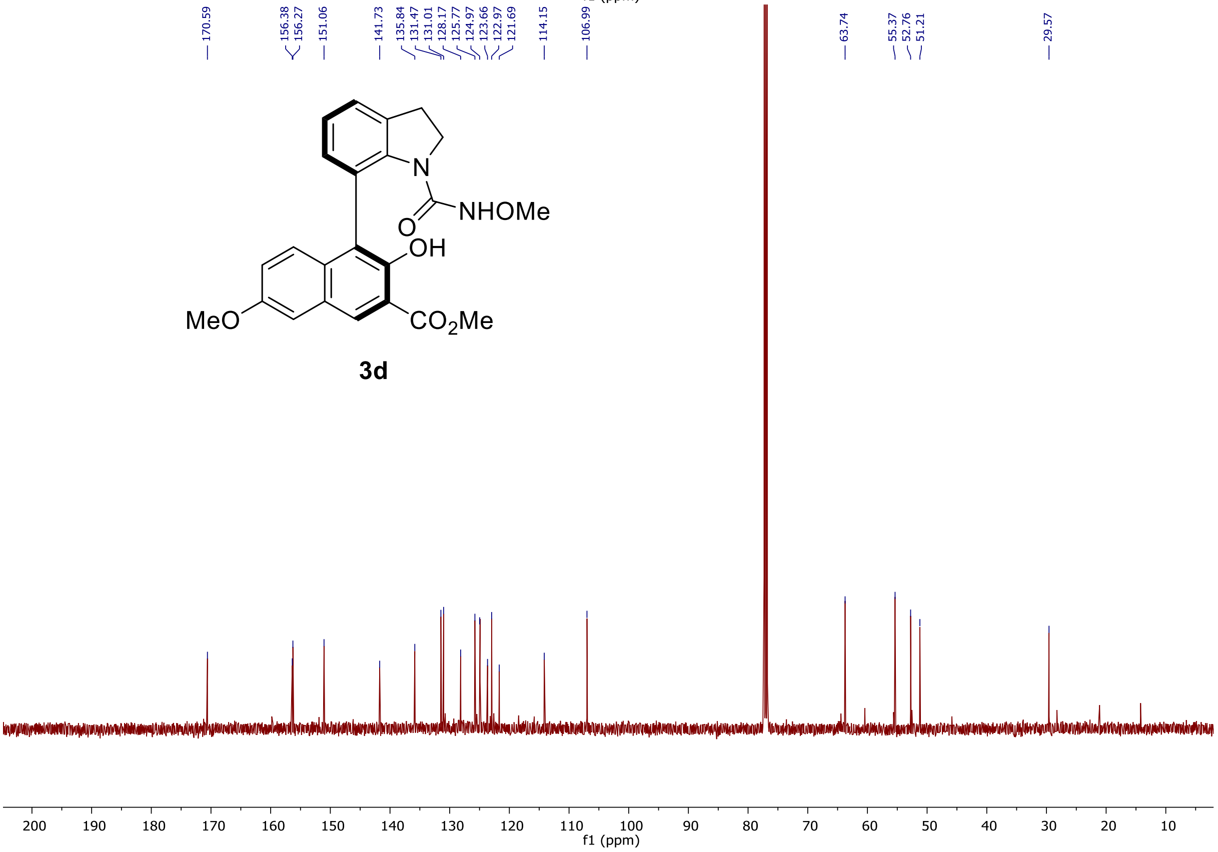
170.59
156.38
156.27
151.06
141.73
135.84
131.47
131.01
128.17
125.77
124.97
123.66
122.97
121.69
114.15
106.99
63.74
55.37
52.76
51.21
29.57
NHOMe
OH
MeO
CO2Me
3d
f1 (ppm)

HPLC traces (**3d**): racemate top, enantiomer bottom:

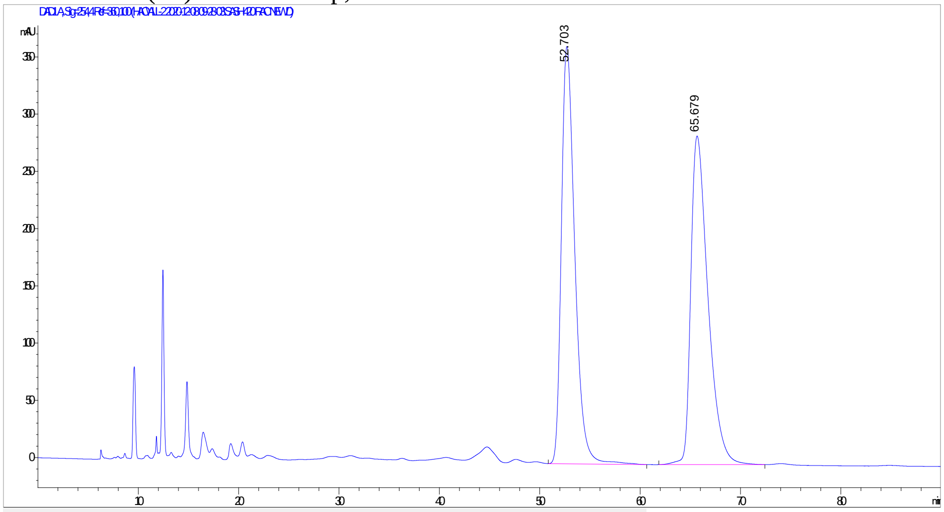

#	Time	Area	Height	Width	Area%	Symmetry
1	52.703	33874.9	364.6	1.4078	49.830	0.64
2	65.679	34105.9	287.1	1.7798	50.170	0.544

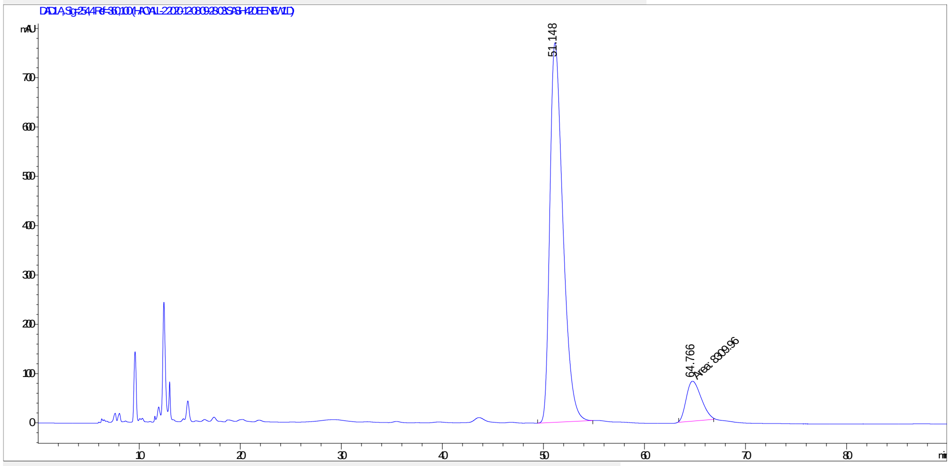

#	Time	Area	Height	Width	Area%	Symmetry
1	51.148	69111.9	769.9	1.3812	88.993	0.605
2	64.766	8547.7	81.6	1.7461	11.007	0.682

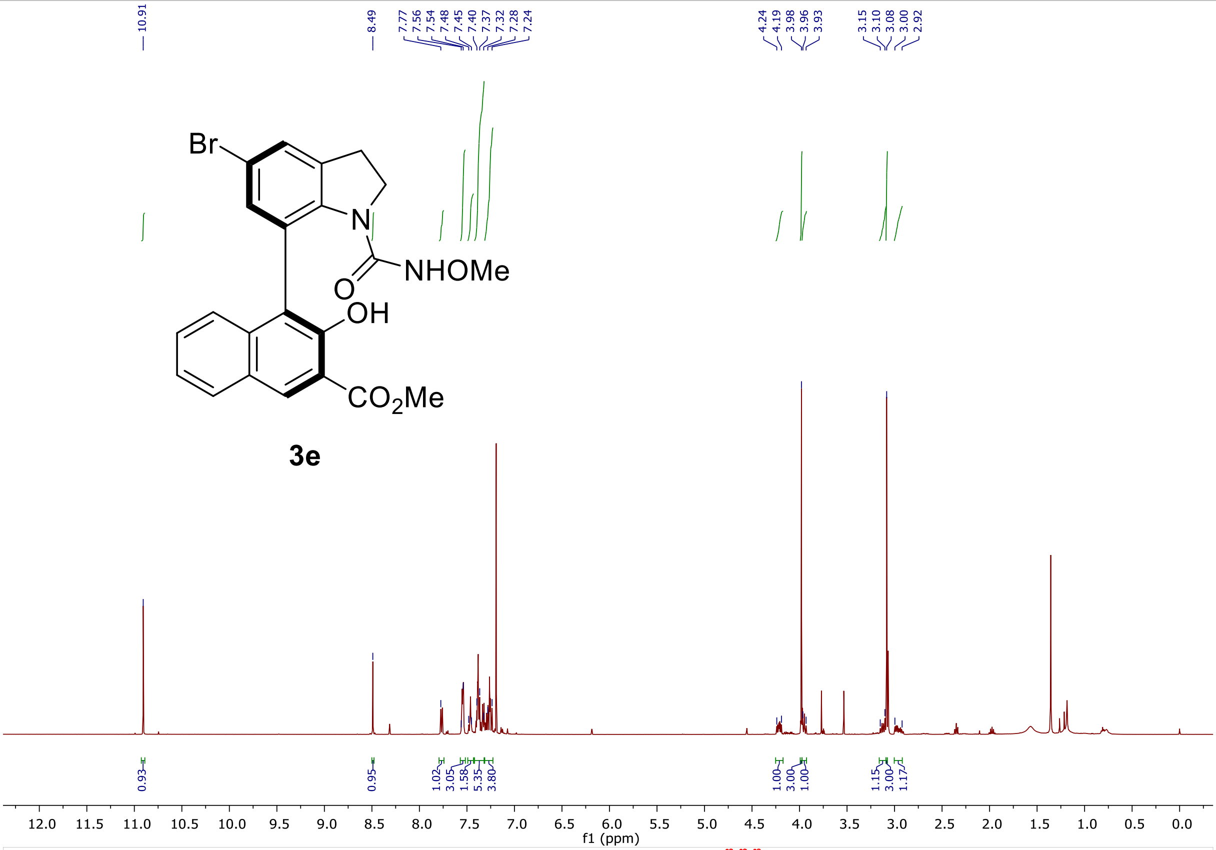
10.91
8.49
7.77
7.56
7.54
7.48
7.45
7.40
7.37
7.32
7.28
7.24
4.24
4.19
3.98
3.96
3.93
3.15
3.10
3.08
3.00
2.92
Br
N
O
NHOMe
OH
CO2Me
3e
0.93
0.95
1.02
3.05
1.58
5.35
3.80
1.00
3.00
1.00
1.15
3.00
1.17
f1 (ppm)

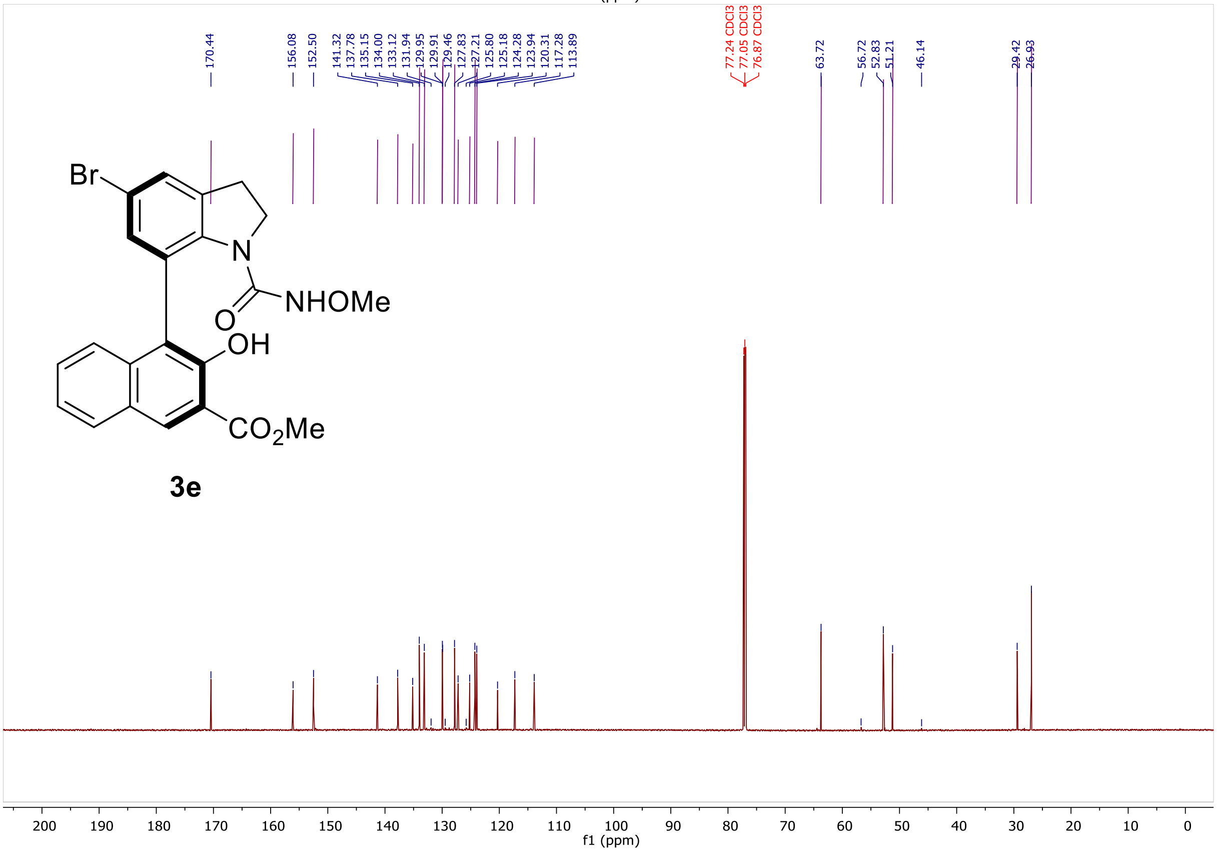
170.44
156.08
152.50
141.32
137.78
135.15
134.00
133.12
131.94
129.95
129.91
129.46
127.83
127.21
125.80
125.18
124.28
123.94
120.31
117.28
113.89
77.24 CDCl3
77.05 CDCl3
76.87 CDCl3
63.72
56.72
52.83
51.21
46.14
29.42
26.93
Br
N
O
NHOMe
OH
CO2Me
3e
f1 (ppm)

HPLC traces (**3e**): racemate top, enantiomer bottom:

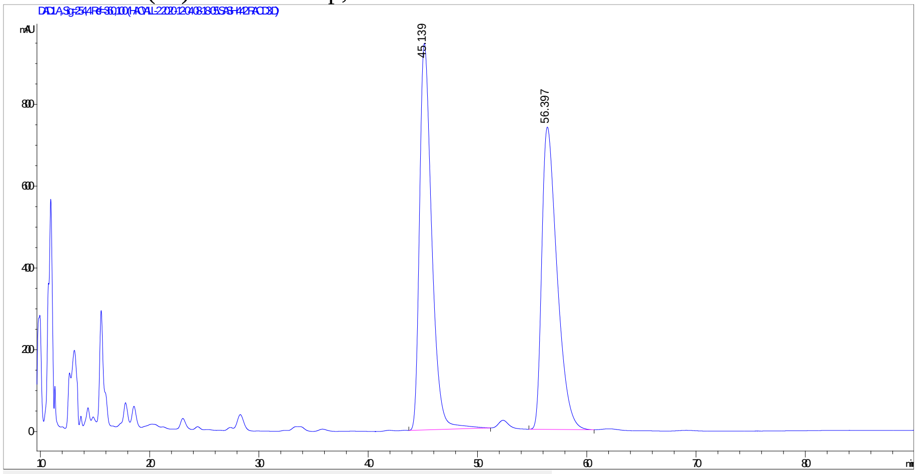

#	Time	Area	Height	Width	Area%	Symmetry
1	45.139	73303.7	945.7	1.1803	50.470	0.603
2	56.397	71939.4	739.4	1.441	49.530	0.543

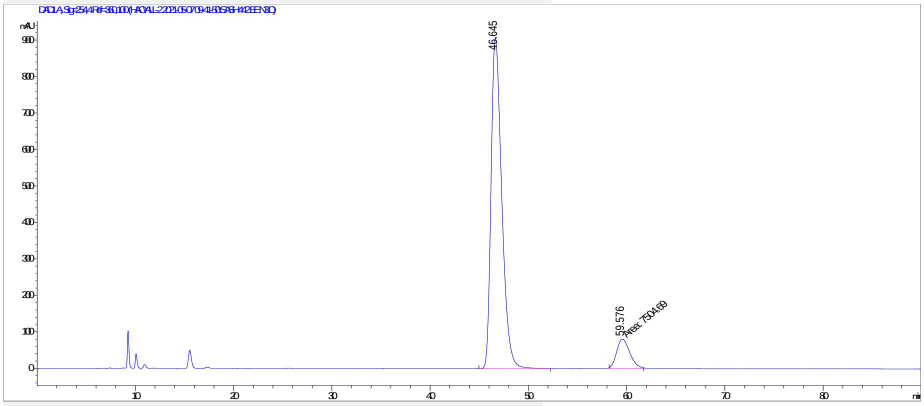

#	Time	Area	Height	Width	Area%	Symmetry
1	46.645	68531.9	909.3	1.2561	90.691	0.661
2	59.576	7034.6	78.8	1.4875	9.309	0.774

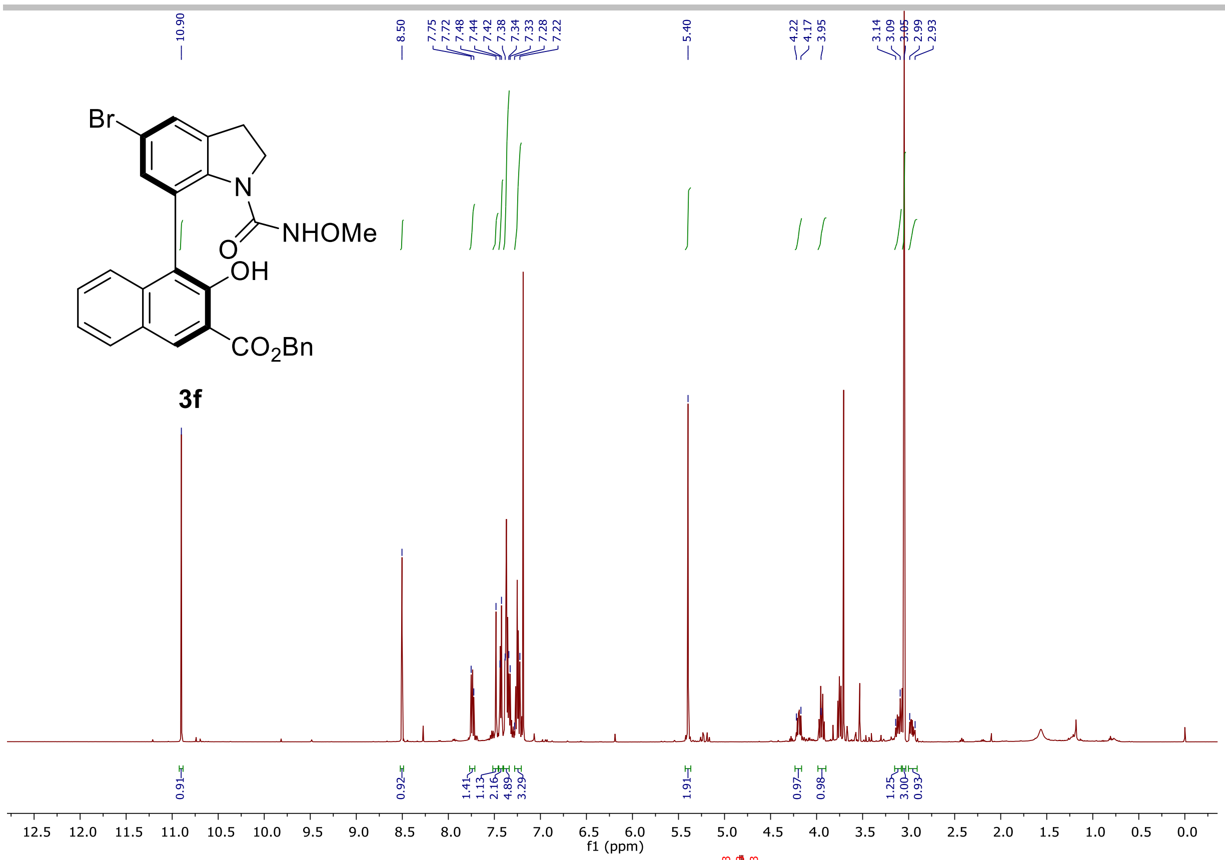
10.90
8.50
7.75
7.72
7.48
7.44
7.42
7.38
7.34
7.33
7.28
7.22
5.40
4.22
4.17
3.95
3.14
3.09
3.05
2.99
2.93
Br
N
NHOMe
O
OH
CO2Bn
3f
0.91
0.92
1.41
1.13
2.16
4.89
3.29
1.91
0.97
0.98
1.25
3.00
0.93
12.5 12.0 11.5 11.0 10.5 10.0 9.5 9.0 8.5 8.0 7.5 7.0 6.5 6.0 5.5 5.0 4.5 4.0 3.5 3.0 2.5 2.0 1.5 1.0 0.5 0.0
f1 (ppm)

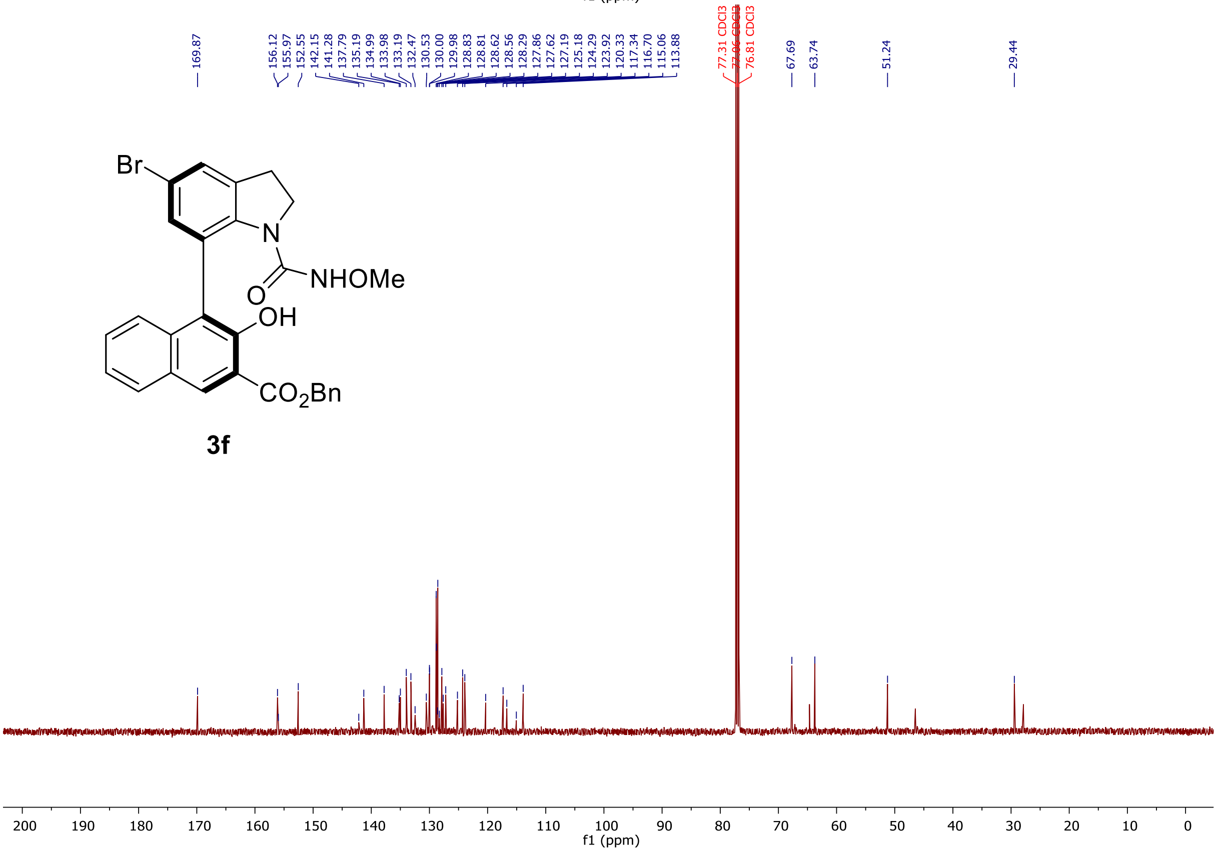
169.87
156.12
155.97
152.55
142.15
141.28
137.79
135.19
134.99
133.98
133.19
132.47
130.53
130.00
129.98
128.83
128.81
128.62
128.56
128.29
127.86
127.62
127.19
125.18
124.29
123.92
120.33
117.34
116.70
115.06
113.88
77.31 CDCl3
77.06 CDCl3
76.81 CDCl3
67.69
63.74
51.24
29.44
Br
N
NHOMe
O
OH
CO2Bn
3f
200 190 180 170 160 150 140 130 120 110 100 90 80 70 60 50 40 30 20 10 0
f1 (ppm)

HPLC traces (**3f**): racemate top, enantiomer bottom:

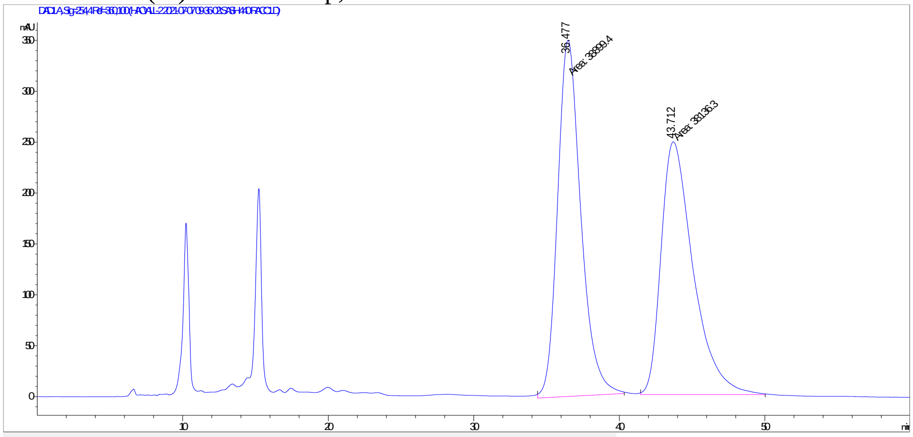

#	Time	Area	Height	Width	Area%	Symmetry
1	36.477	38526.9	349.2	1.8386	51.644	0.792
2	43.712	36074.4	244.9	2.455	48.356	0.625

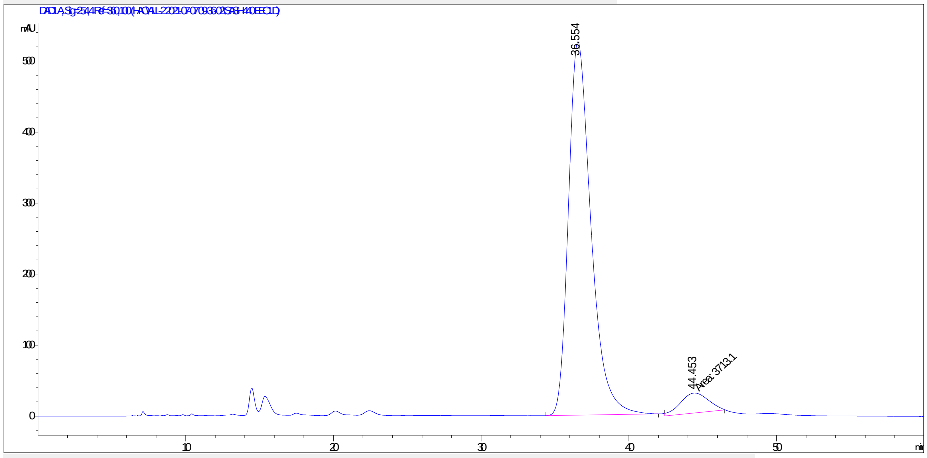

#	Time	Area	Height	Width	Area%	Symmetry
1	36.554	55774.1	525.9	1.6014	93.646	0.68
2	44.453	3784.5	28.1	2.243	6.354	0.823

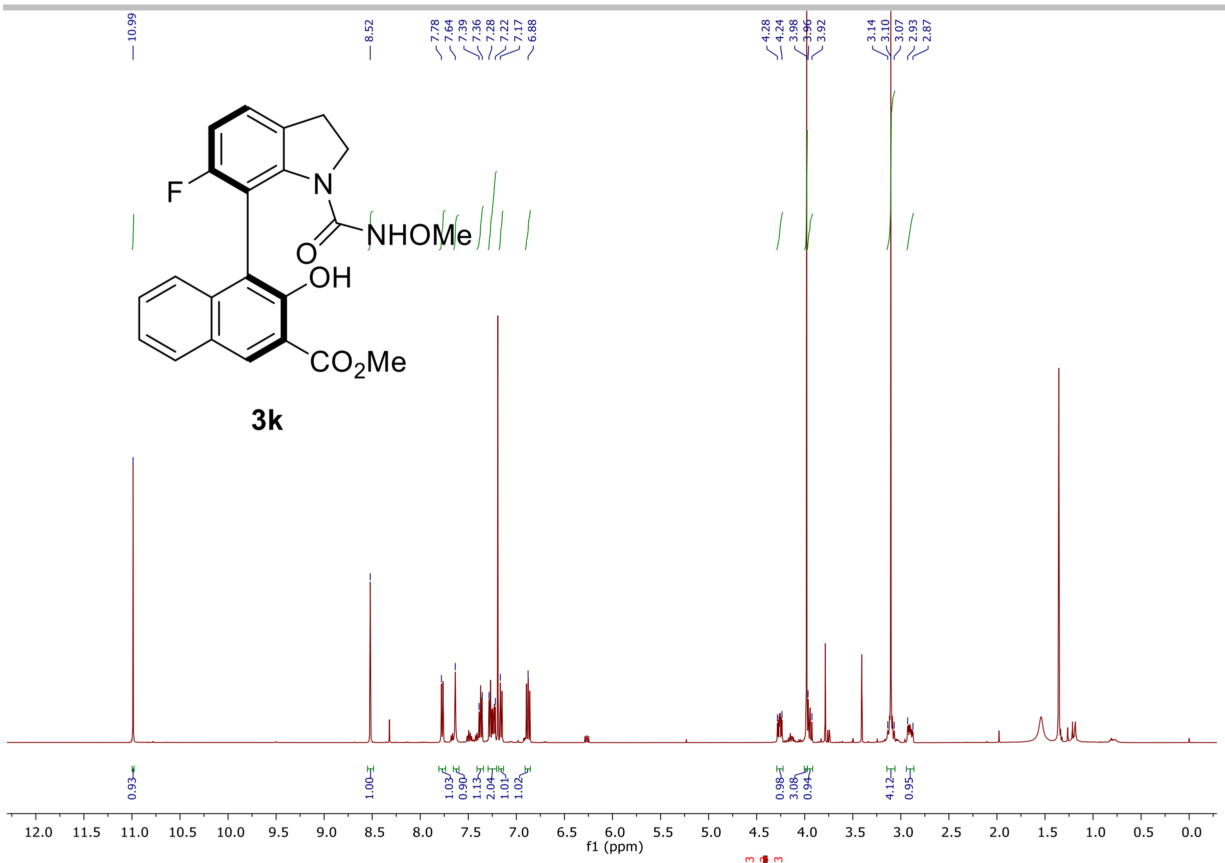
10.99
8.52
7.78
7.64
7.39
7.36
7.28
7.22
7.17
6.88
4.28
4.24
3.98
3.96
3.92
3.14
3.10
3.07
2.93
2.87
F
N
O
NHOMe
OH
CO2Me
3k
0.93
1.00
1.03
0.90
1.13
2.04
1.01
1.02
0.98
3.08
0.94
4.12
0.95
f1 (ppm)

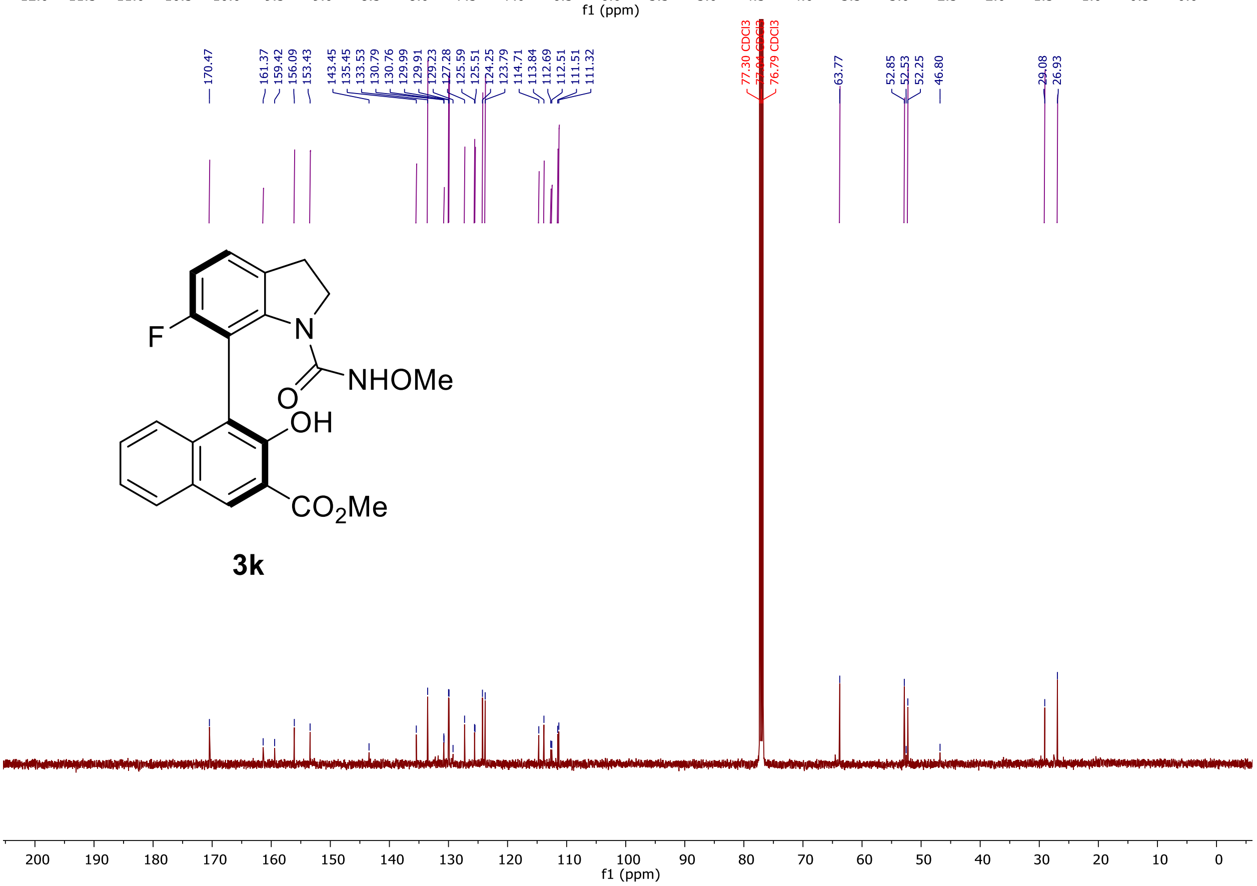
170.47
161.37
159.42
156.09
153.43
143.45
135.45
133.53
130.79
130.76
129.99
129.91
129.23
127.28
125.59
125.51
124.25
123.79
114.71
113.84
112.69
112.51
111.51
111.32
77.30 CDCl3
76.79 CDCl3
63.77
52.85
52.53
52.25
46.80
29.08
26.93
F
N
O
NHOMe
OH
CO2Me
3k
f1 (ppm)

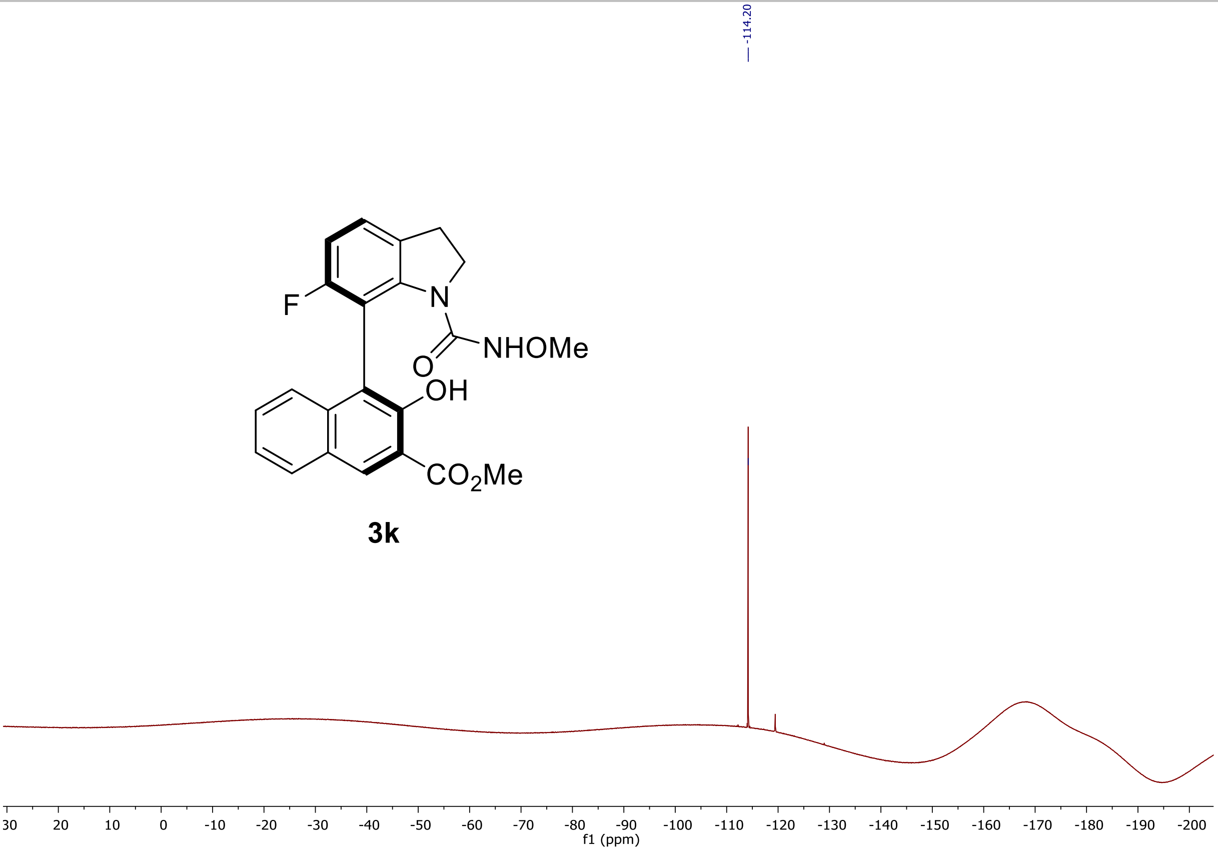
F
N
O
NHOMe
OH
CO2Me
3k
-114.20
30
20
10
0
-10
-20
-30
-40
-50
-60
-70
-80
-90
-100
-110
-120
-130
-140
-150
-160
-170
-180
-190
-200
f1 (ppm)

HPLC traces (**3k**): racemate top, enantiomer bottom:

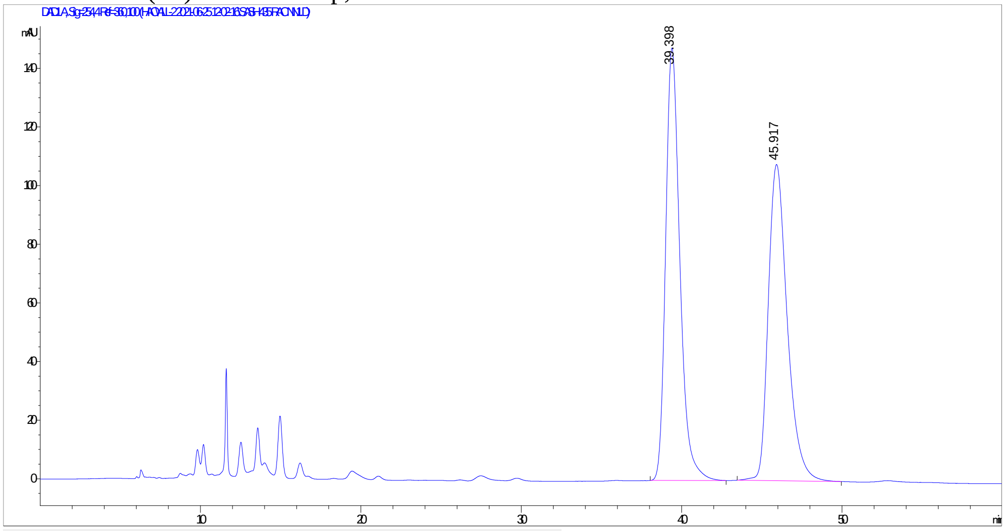

#	Time	Area	Height	Width	Area%	Symmetry
1	39.398	9086.7	147.5	0.9485	50.656	0.737
2	45.917	8851.2	107.9	1.2554	49.344	0.72

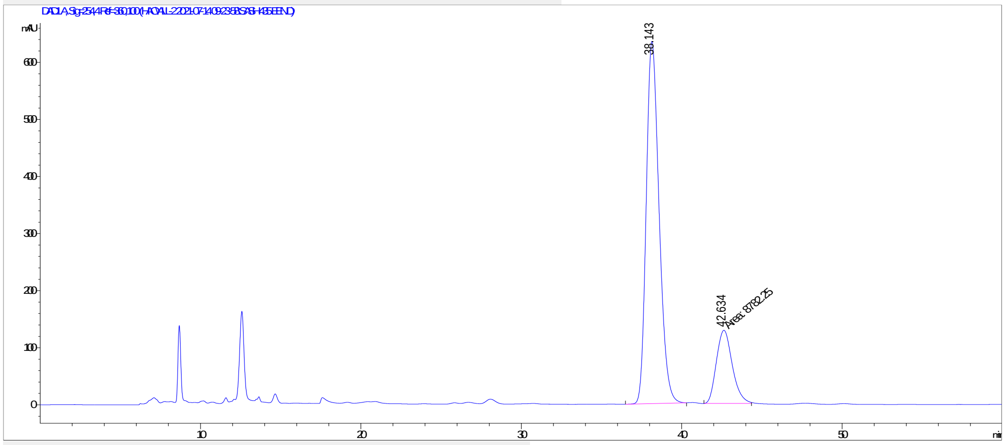

#	Time	Area	Height	Width	Area%	Symmetry
1	38.143	35680.3	637.1	0.9334	80.545	0.753
2	42.634	8618.5	127.4	1.1276	19.455	0.887

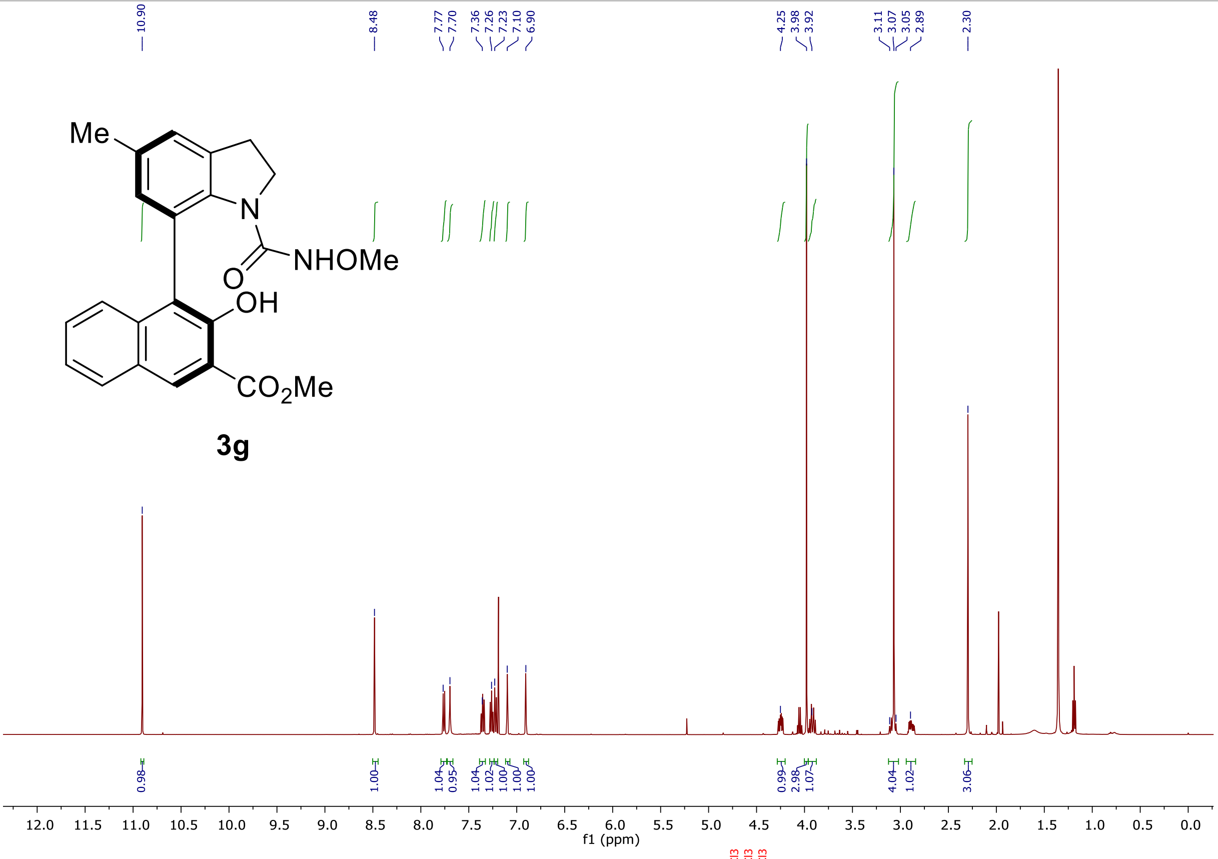
10.90
8.48
7.77
7.70
7.36
7.26
7.23
7.10
6.90
4.25
3.98
3.92
3.11
3.07
3.05
2.89
2.30
Me
N
NHOMe
O
OH
CO2Me
3g
0.98
1.00
1.04
0.95
1.04
1.02
1.00
1.00
1.00
0.99
2.98
1.07
4.04
1.02
3.06
f1 (ppm)

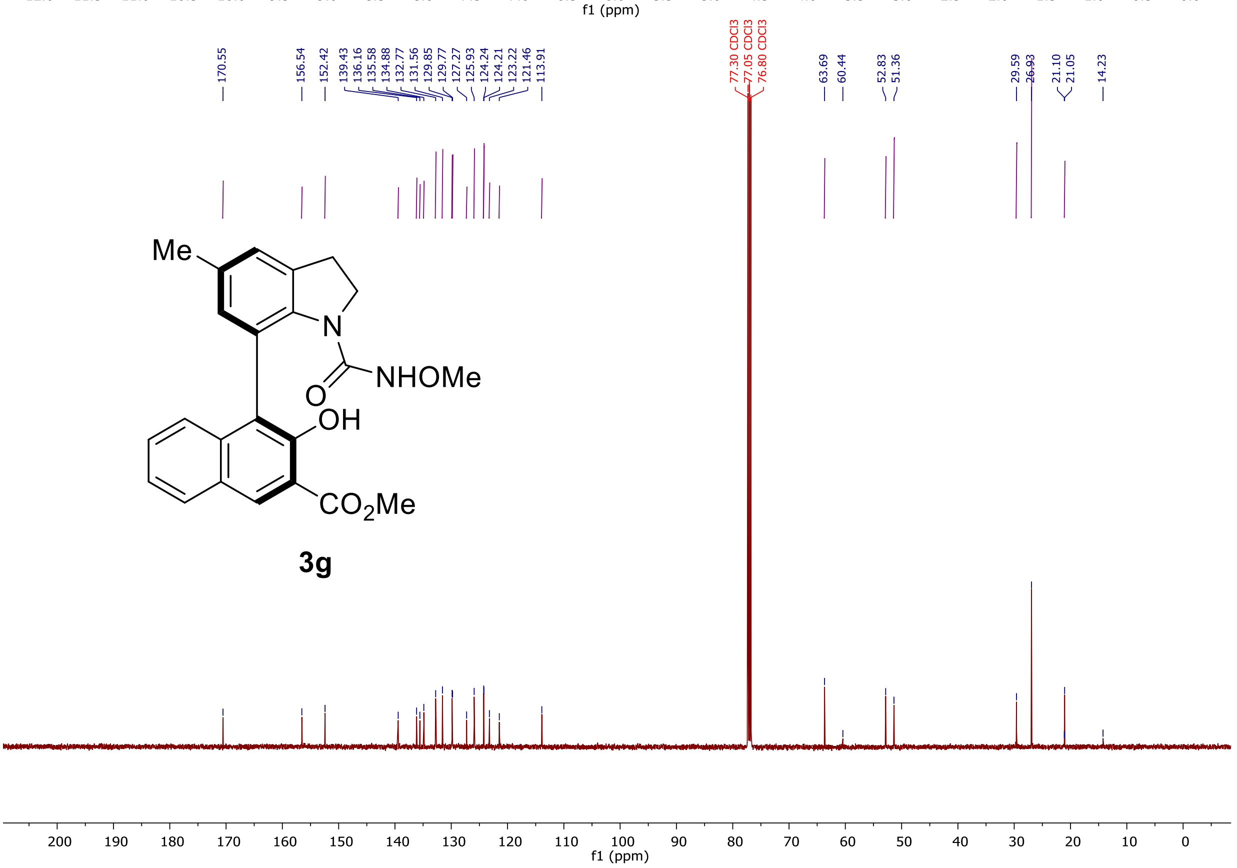
170.55
156.54
152.42
139.43
136.16
135.58
134.88
132.77
131.56
129.85
129.77
127.27
125.93
124.24
124.21
123.22
121.46
113.91
77.30 CDCl3
77.05 CDCl3
76.80 CDCl3
63.69
60.44
52.83
51.36
29.59
26.93
21.10
21.05
14.23
Me
N
NHOMe
O
OH
CO2Me
3g
f1 (ppm)

HPLC traces (**3g**): racemate top, enantiomer bottom:

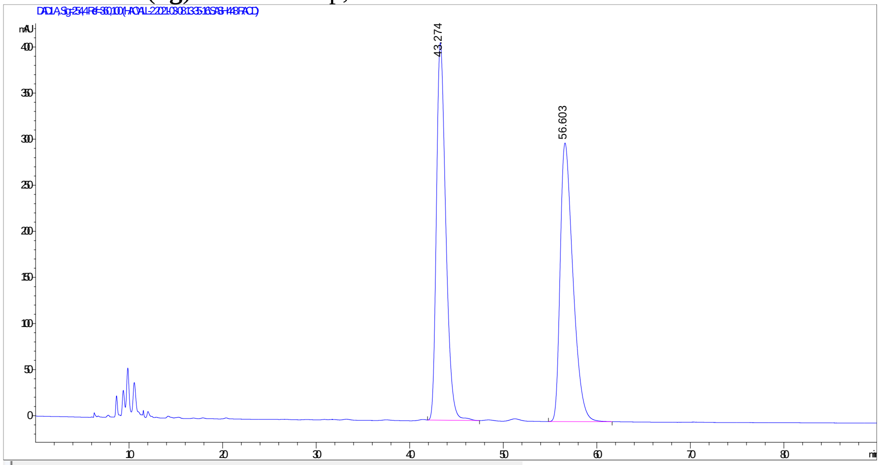

#	Time	Area	Height	Width	Area%	Symmetry
1	43.274	28485.1	409.8	1.0629	49.914	0.672
2	56.603	28583.6	302.3	1.4209	50.086	0.571

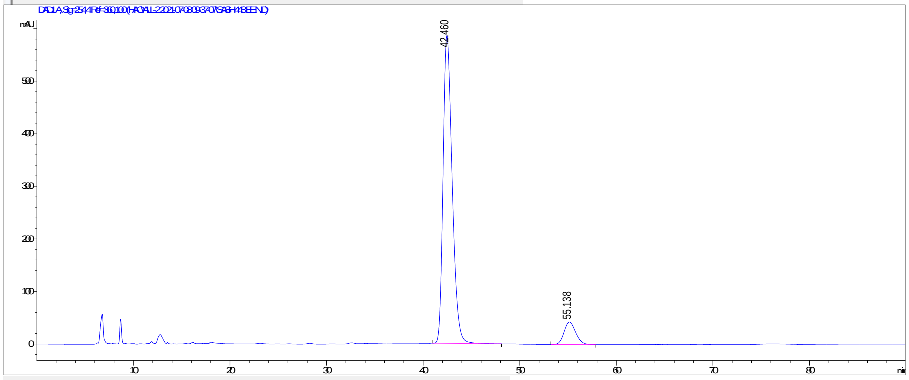

#	Time	Area	Height	Width	Area%	Symmetry
1	42.46	38297.2	585.6	1.0009	91.468	0.686
2	55.138	3572.3	42.8	1.2591	8.532	0.816

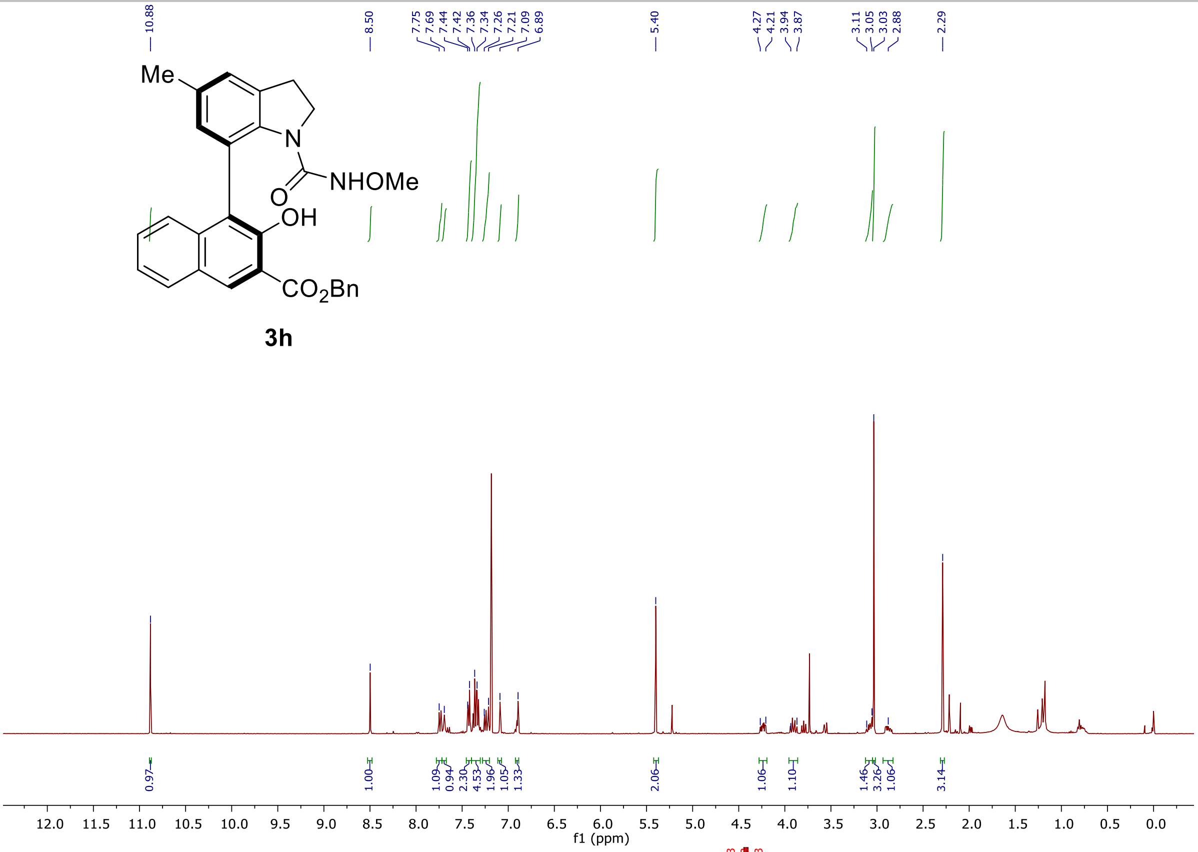

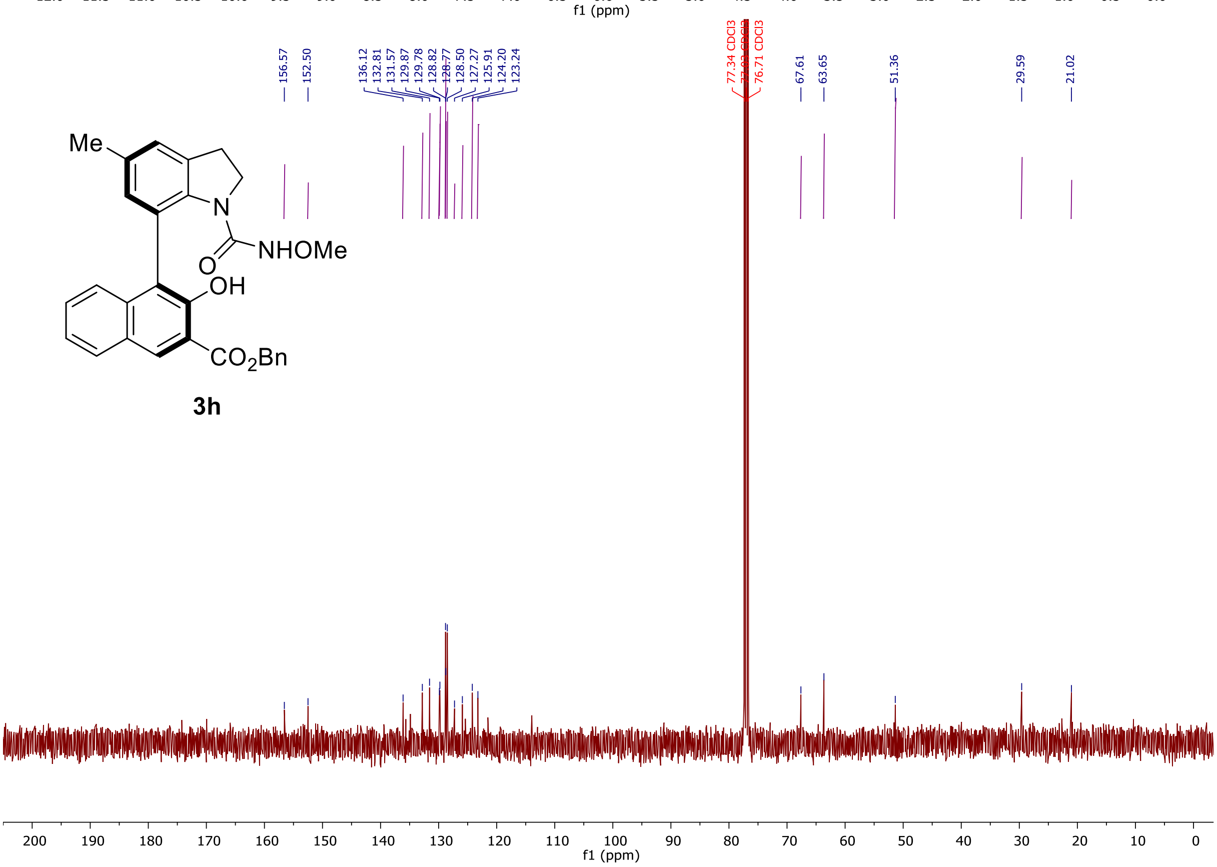

HPLC traces (**3h**): racemate top, enantiomer bottom:

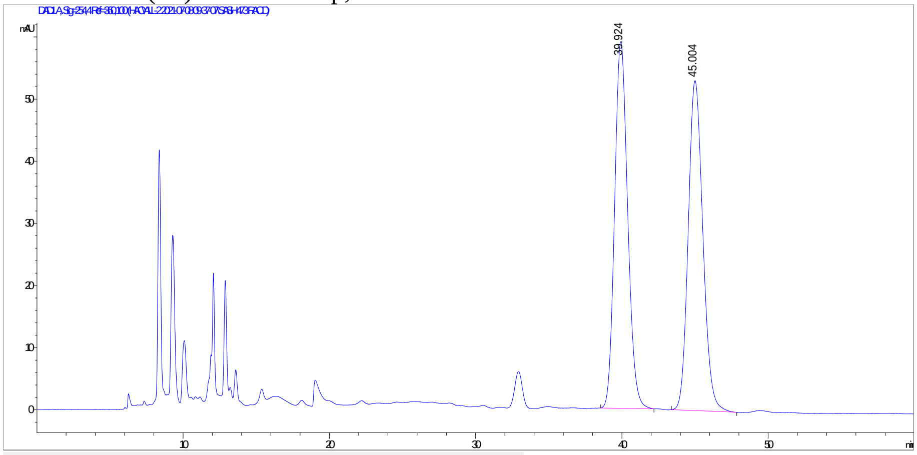

#	Time	Area	Height	Width	Area%	Symmetry
1	39.891	9037.5	146.8	0.9421	49.480	0.818
2	44.982	9227.6	131.2	1.0885	50.520	0.774

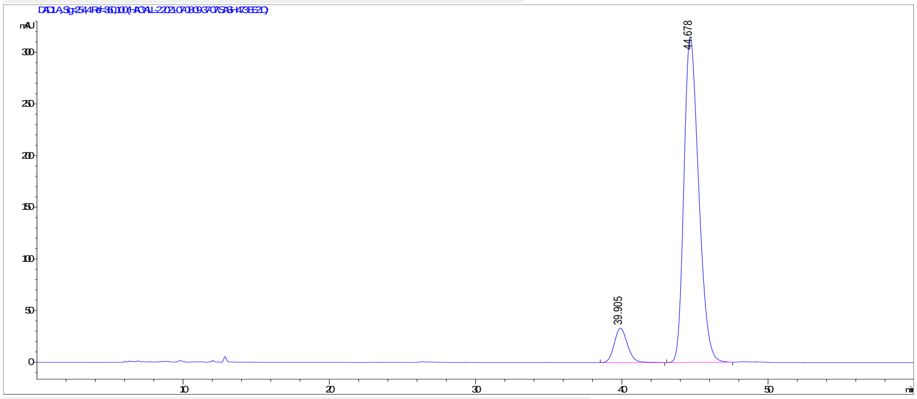

#	Time	Area	Height	Width	Area%	Symmetry
1	39.905	2081.5	33.3	0.9541	8.609	0.832
2	44.678	22098.2	314.9	1.0827	91.391	0.703

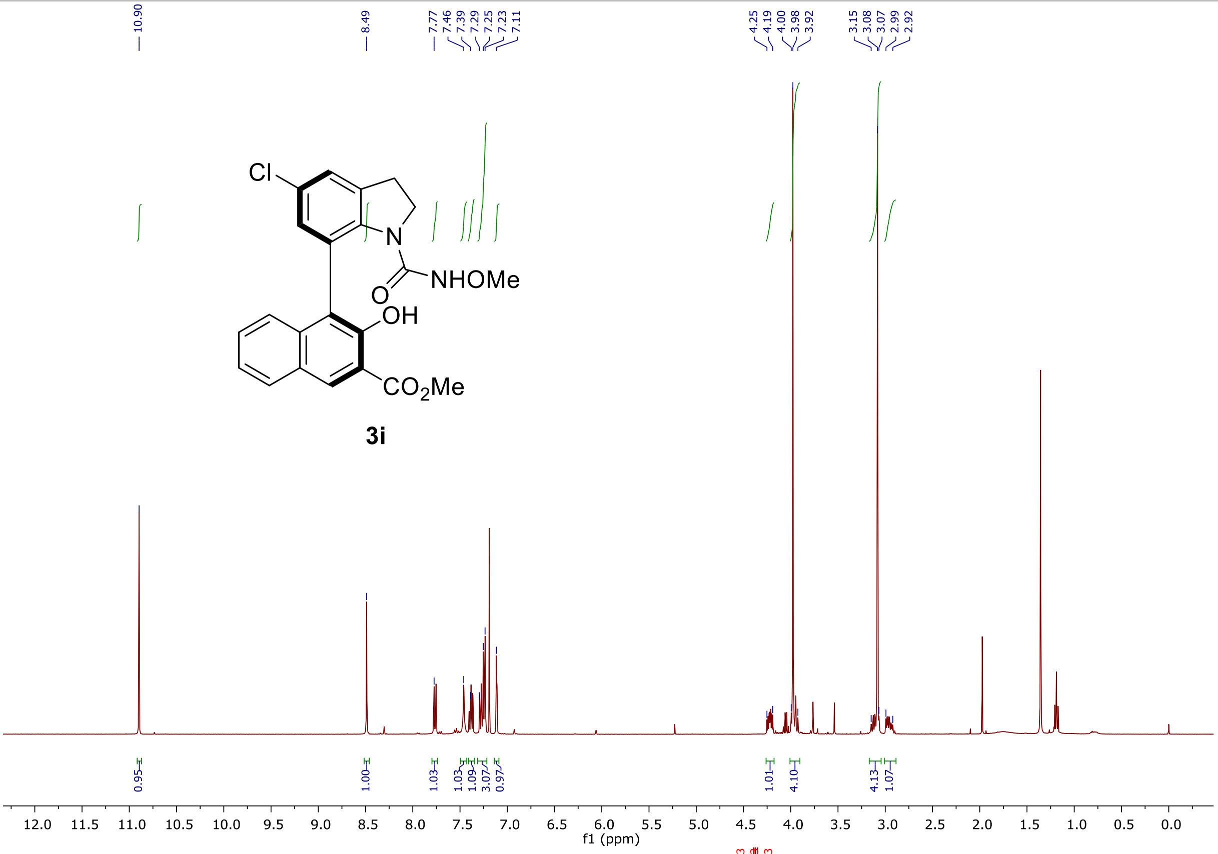

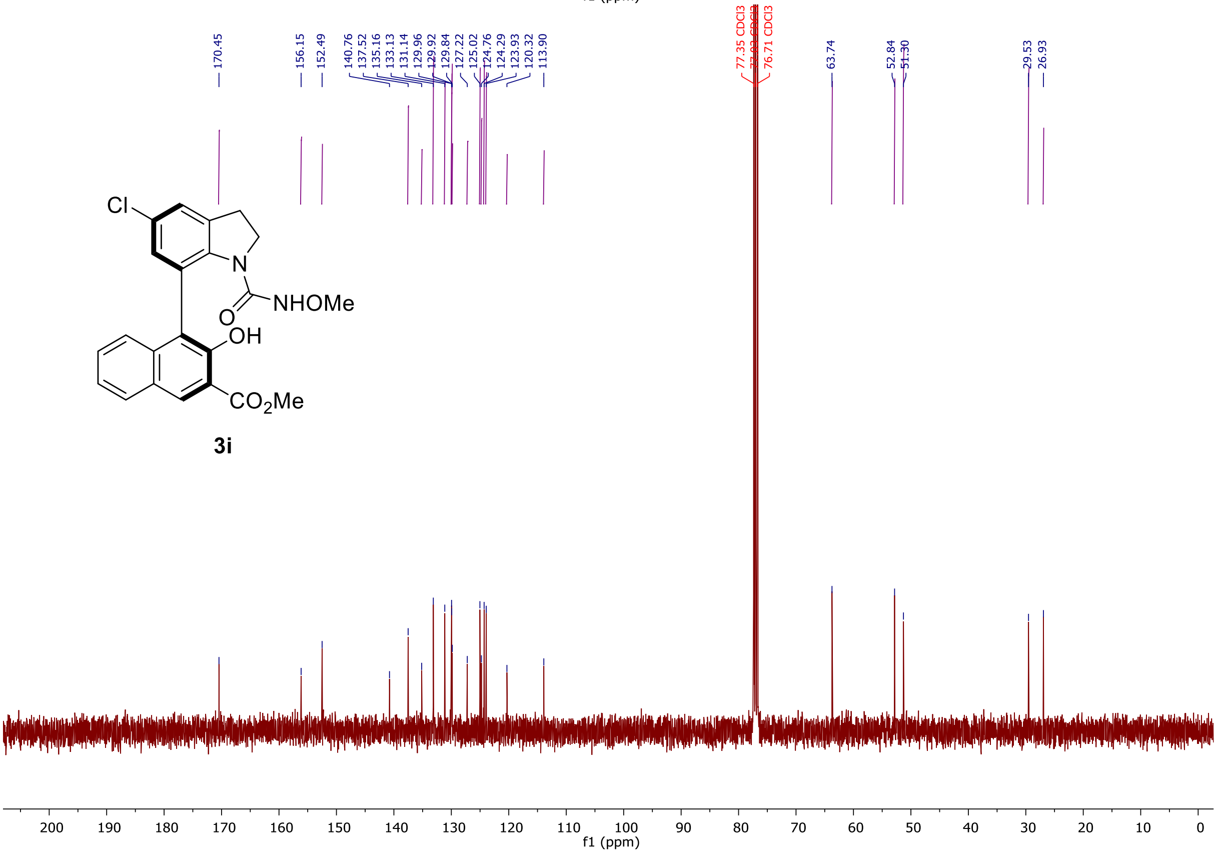

HPLC traces (**3i**): racemate top, enantiomer bottom:

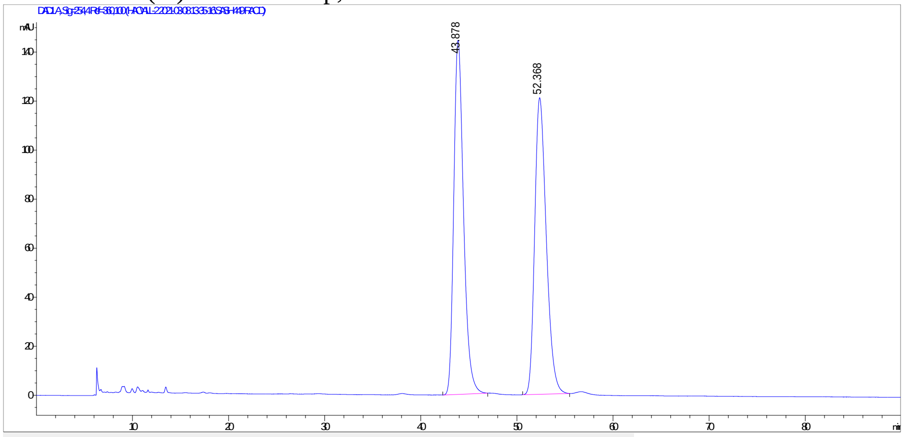

#	Time	Area	Height	Width	Area%	Symmetry
1	43.878	10221.8	144.5	1.0831	50.250	0.732
2	52.368	10120	121	1.2756	49.750	0.703

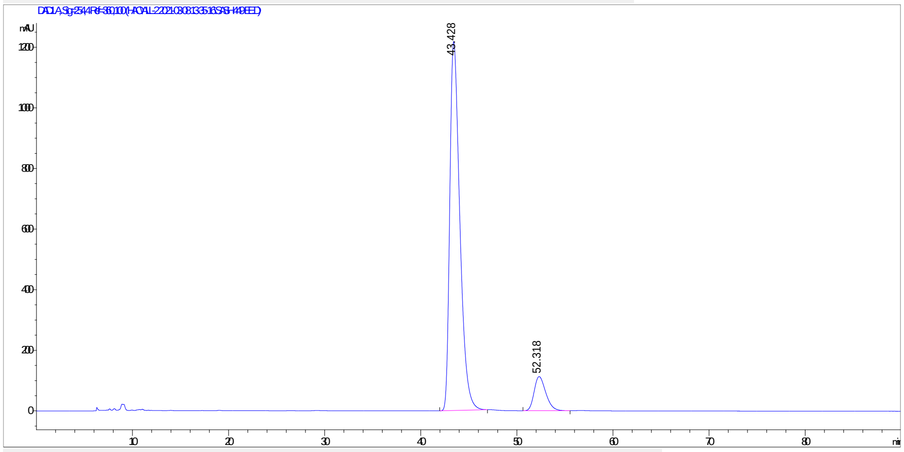

#	Time	Area	Height	Width	Area%	Symmetry
1	43.428	88265.5	1217.8	1.0956	90.282	0.604
2	52.318	9501.2	112.8	1.2488	9.718	0.718

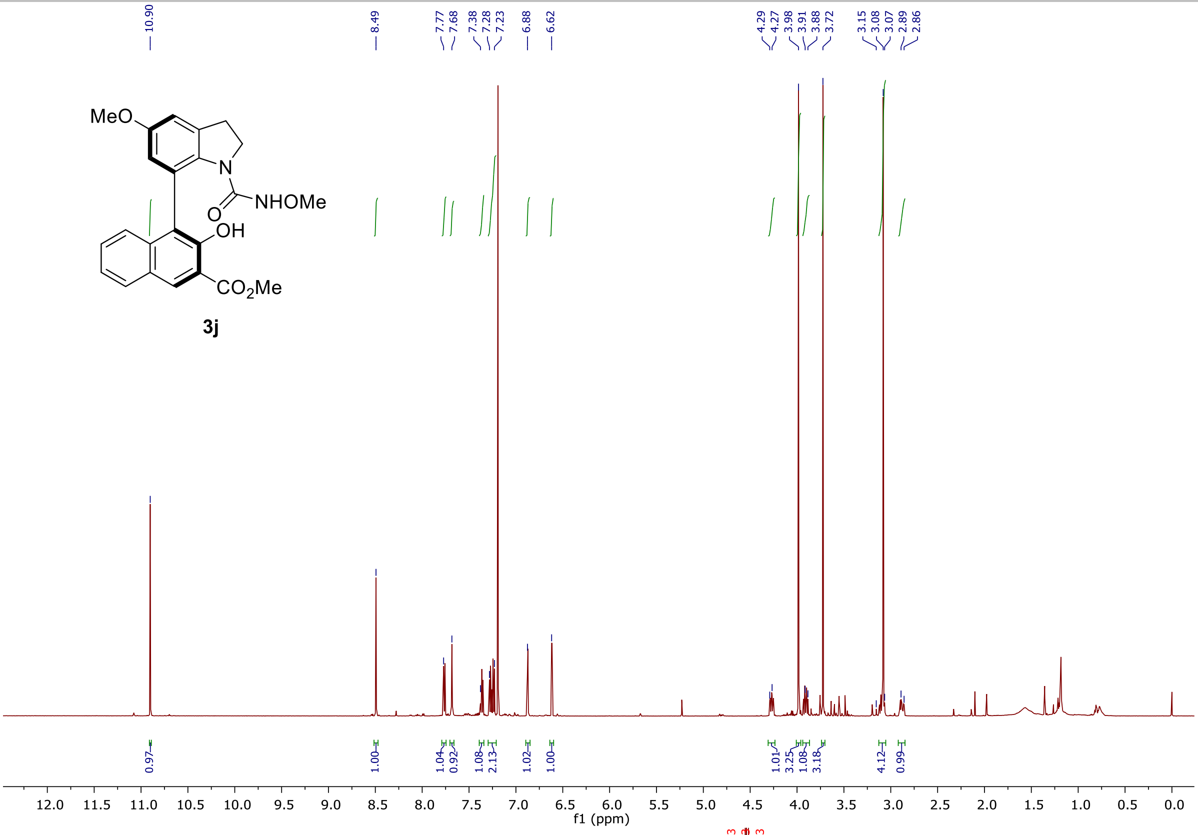

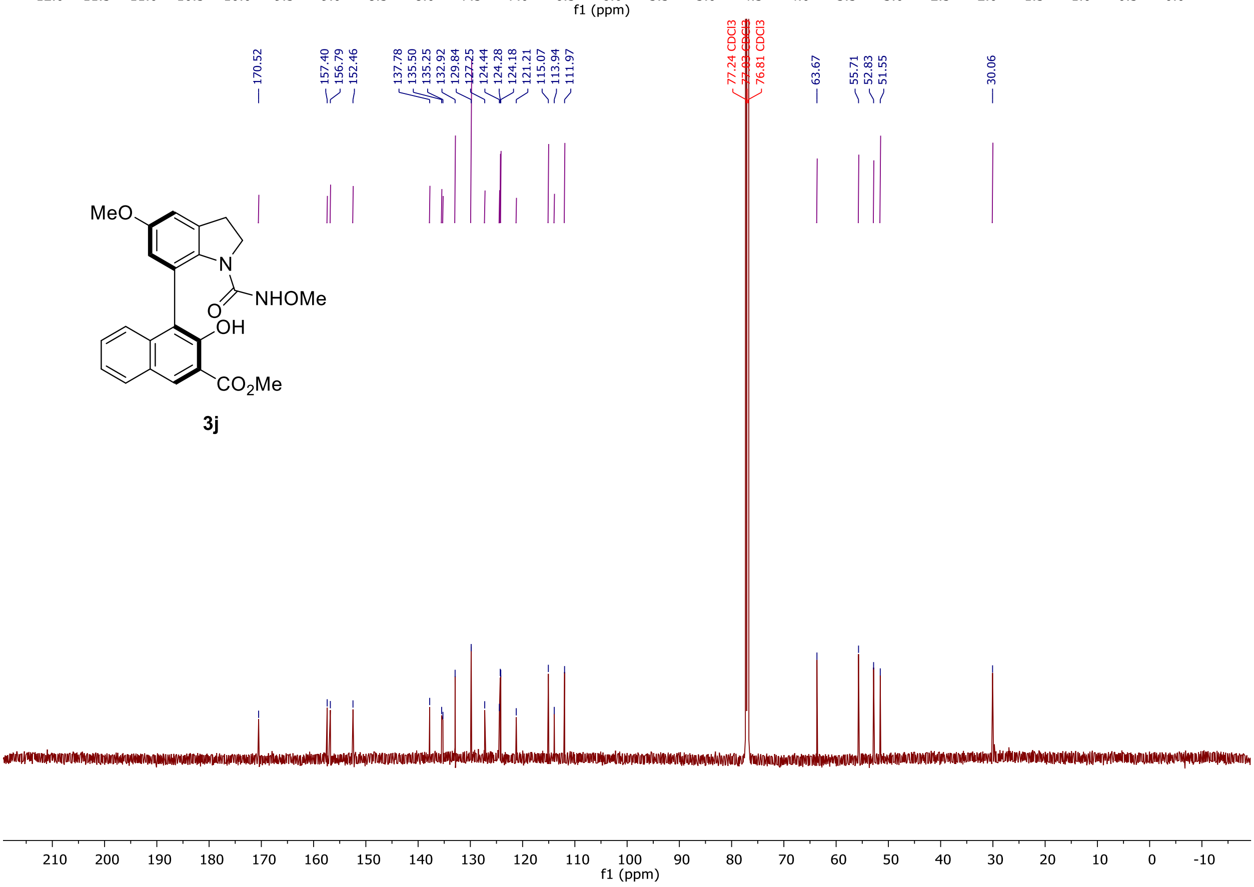

HPLC traces (**3j**): racemate top, enantiomer bottom:

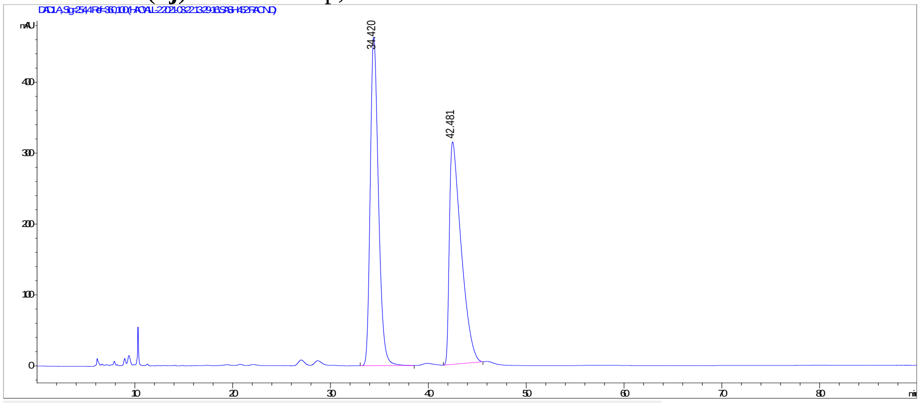

#	Time	Area	Height	Width	Area%	Symmetry
1	34.42	27027.9	463.2	0.9008	50.687	0.695
2	42.481	26295.3	313.9	1.2193	49.313	0.348

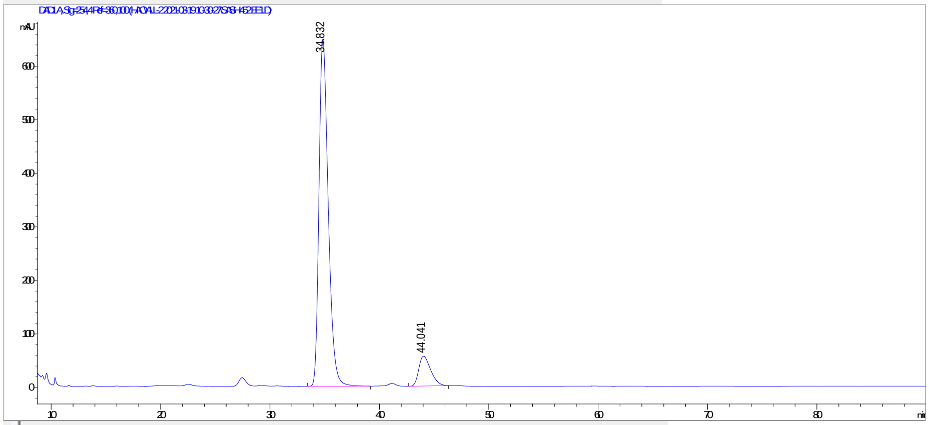

#	Time	Area	Height	Width	Area%	Symmetry
1	34.832	38128.6	648.6	0.9078	90.055	0.666
2	44.041	4210.7	55.8	1.1163	9.945	0.638

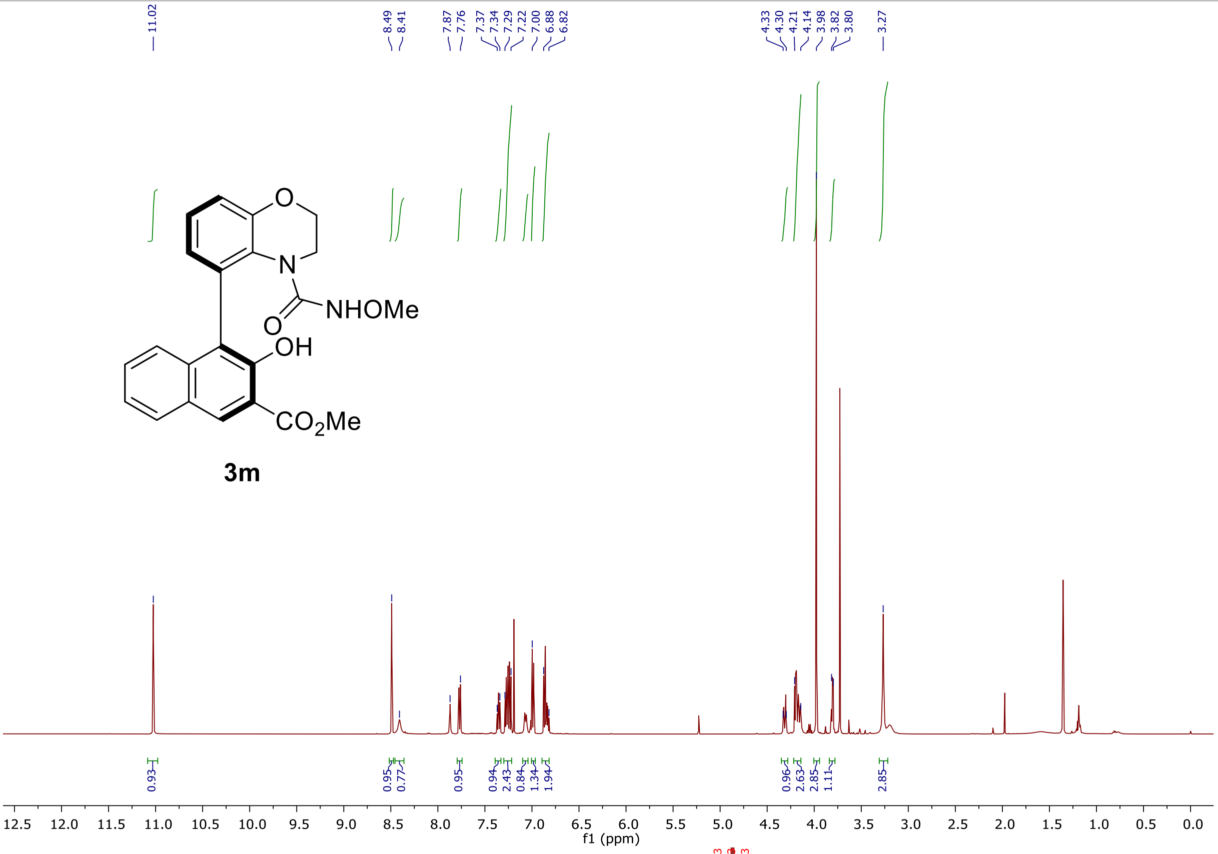
11.02
8.49
8.41
7.87
7.76
7.37
7.34
7.29
7.22
7.00
6.88
6.82
4.33
4.30
4.21
4.14
3.98
3.82
3.80
3.27
O
N
NHOMe
O
OH
CO2Me
3m
0.93
0.95
0.77
0.95
0.94
2.43
0.84
1.34
1.94
0.96
2.63
2.85
1.11
2.85
12.5 12.0 11.5 11.0 10.5 10.0 9.5 9.0 8.5 8.0 7.5 7.0 6.5 6.0 5.5 5.0 4.5 4.0 3.5 3.0 2.5 2.0 1.5 1.0 0.5 0.0
f1 (ppm)

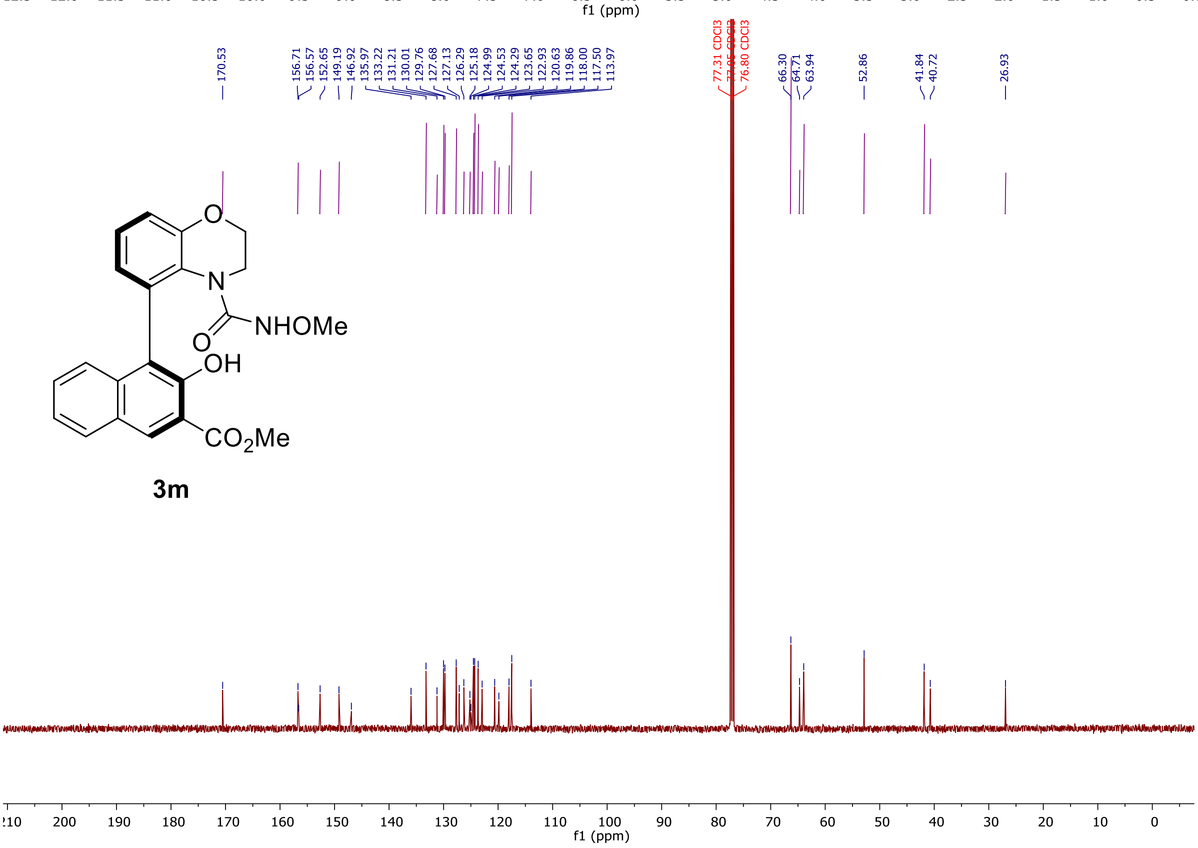
170.53
156.71
156.57
152.65
149.19
146.92
135.97
133.22
131.21
130.01
129.76
127.68
127.13
126.29
125.18
124.99
124.53
124.29
123.65
122.93
120.63
119.86
118.00
117.50
113.97
77.31 CDCl3
76.80 CDCl3
66.30
64.71
63.94
52.86
41.84
40.72
26.93
O
N
NHOMe
O
OH
CO2Me
3m
200 190 180 170 160 150 140 130 120 110 100 90 80 70 60 50 40 30 20 10 0
f1 (ppm)

HPLC traces (**3m**): racemate top, enantiomer bottom:

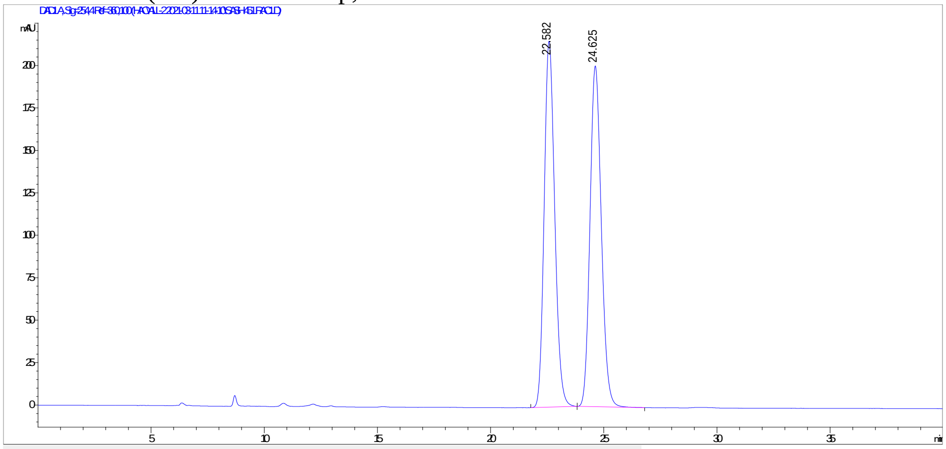

#	Time	Area	Height	Width	Area%	Symmetry
1	22.582	6978.9	215.6	0.5036	50.070	0.816
2	24.625	6959.4	200.7	0.5385	49.930	0.837

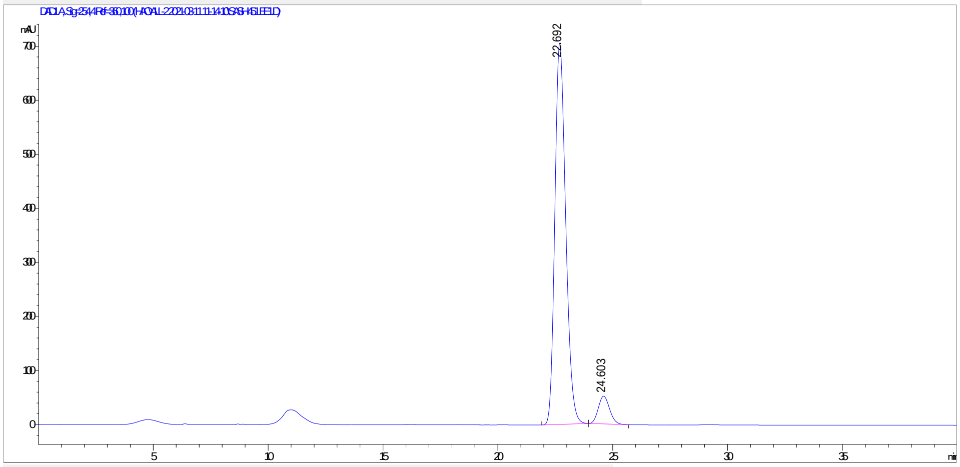

#	Time	Area	Height	Width	Area%	Symmetry
1	22.692	23137.7	705.6	0.5066	92.925	0.762
2	24.603	1761.7	51.5	0.5367	7.075	0.852

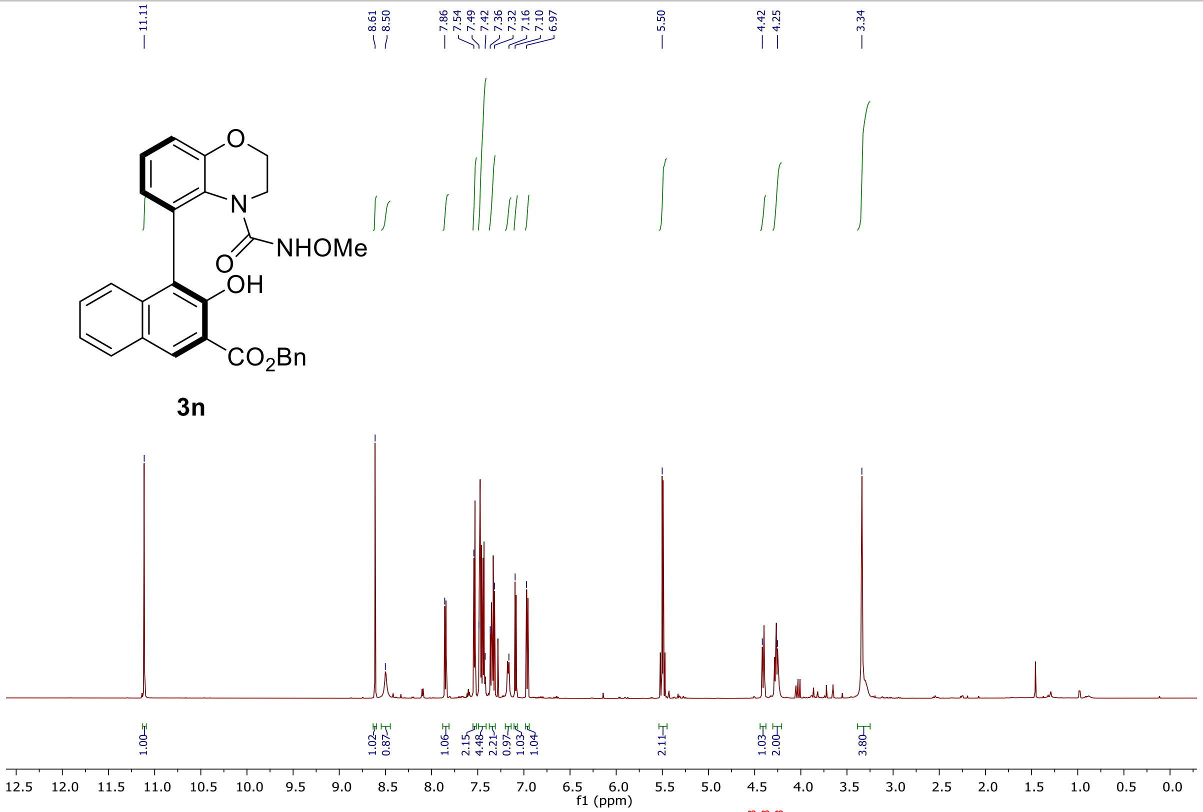
11.11
8.61
8.50
7.86
7.54
7.49
7.42
7.36
7.32
7.16
7.10
6.97
5.50
4.42
4.25
3.34
O
N
NHOMe
O
OH
CO2Bn
3n
1.00
1.02
0.87
1.06
2.15
4.48
2.21
0.97
1.03
1.04
2.11
1.03
2.00
3.80
f1 (ppm)

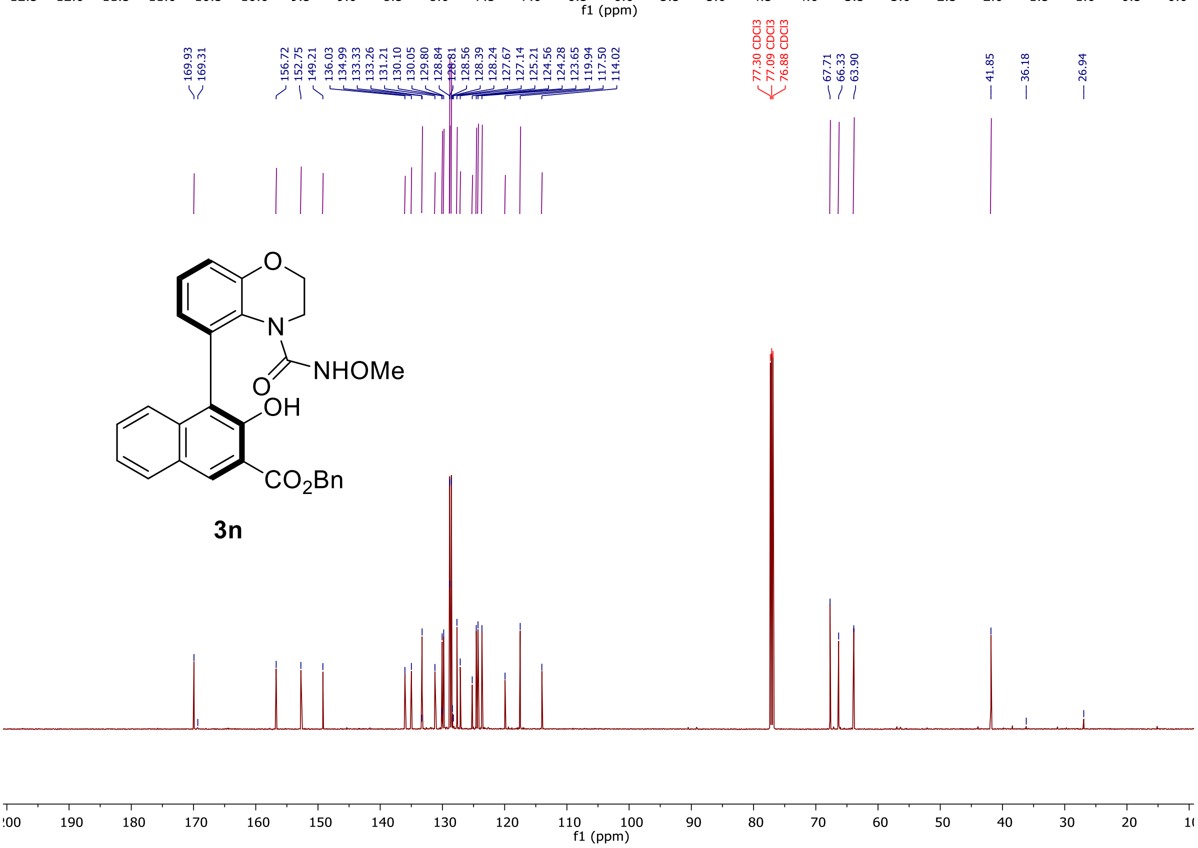
169.93
169.31
156.72
152.75
149.21
136.03
134.99
133.33
133.26
131.21
130.10
130.05
129.80
128.84
128.81
128.56
128.39
128.24
127.67
127.14
125.21
124.56
124.28
123.65
119.94
117.50
114.02
77.30 CDCl3
77.09 CDCl3
76.88 CDCl3
67.71
66.33
63.90
41.85
36.18
26.94
O
N
NHOMe
O
OH
CO2Bn
3n
f1 (ppm)

HPLC traces (**3n**): racemate top, enantiomer bottom:

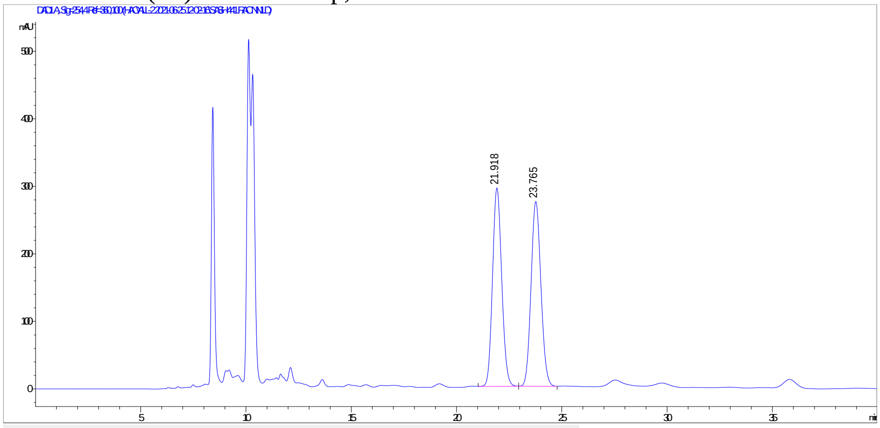

#	Time	Area	Height	Width	Area%	Symmetry
1	21.918	8896.7	293.8	0.4712	49.999	0.874
2	23.765	8897.2	273.6	0.5054	50.001	0.888

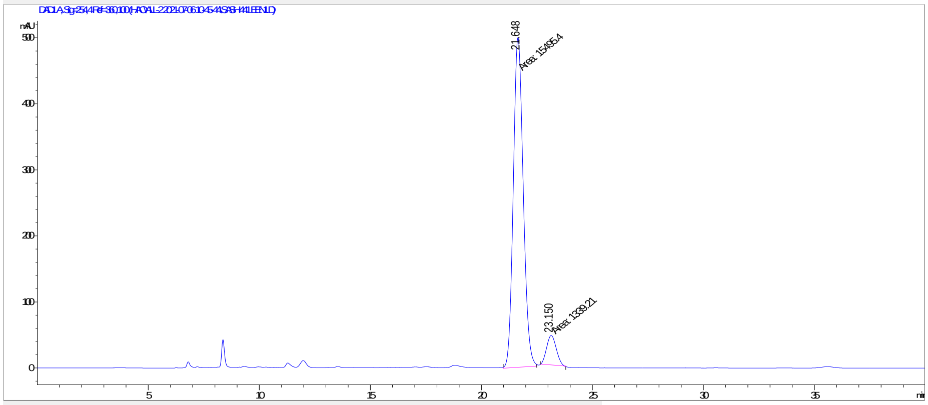

#	Time	Area	Height	Width	Area%	Symmetry
1	21.648	15473.7	499.3	0.5166	91.378	0.863
2	23.15	1459.9	46.2	0.5264	8.622	0.851

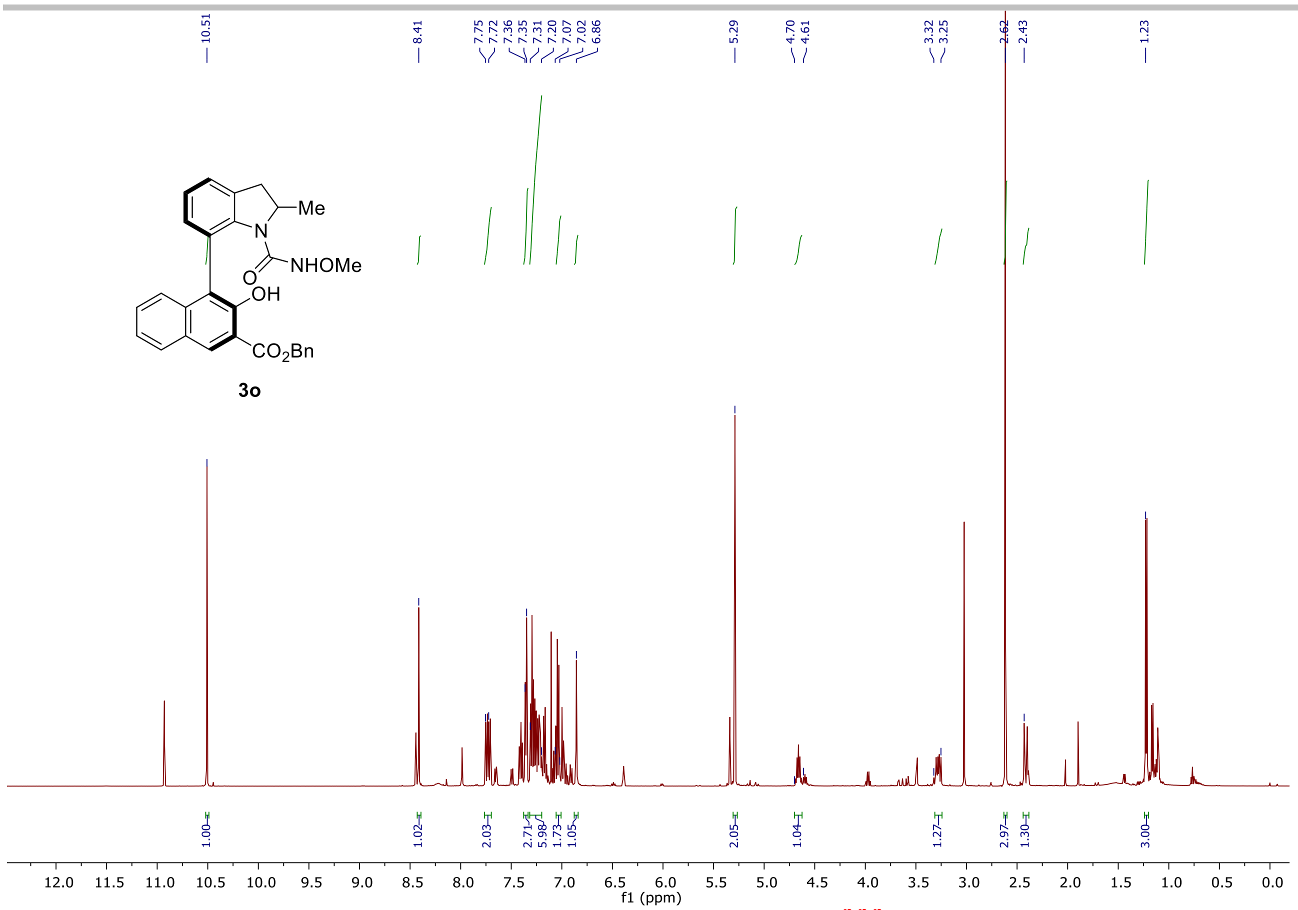
10.51
8.41
7.75
7.72
7.36
7.35
7.31
7.20
7.07
7.02
6.86
5.29
4.70
4.61
3.32
3.25
2.62
2.43
1.23
Me
N
NHOMe
O
OH
CO2Bn
3o
1.00
1.02
2.03
2.71
5.98
1.73
1.05
2.05
1.04
1.27
2.97
1.30
3.00
f1 (ppm)

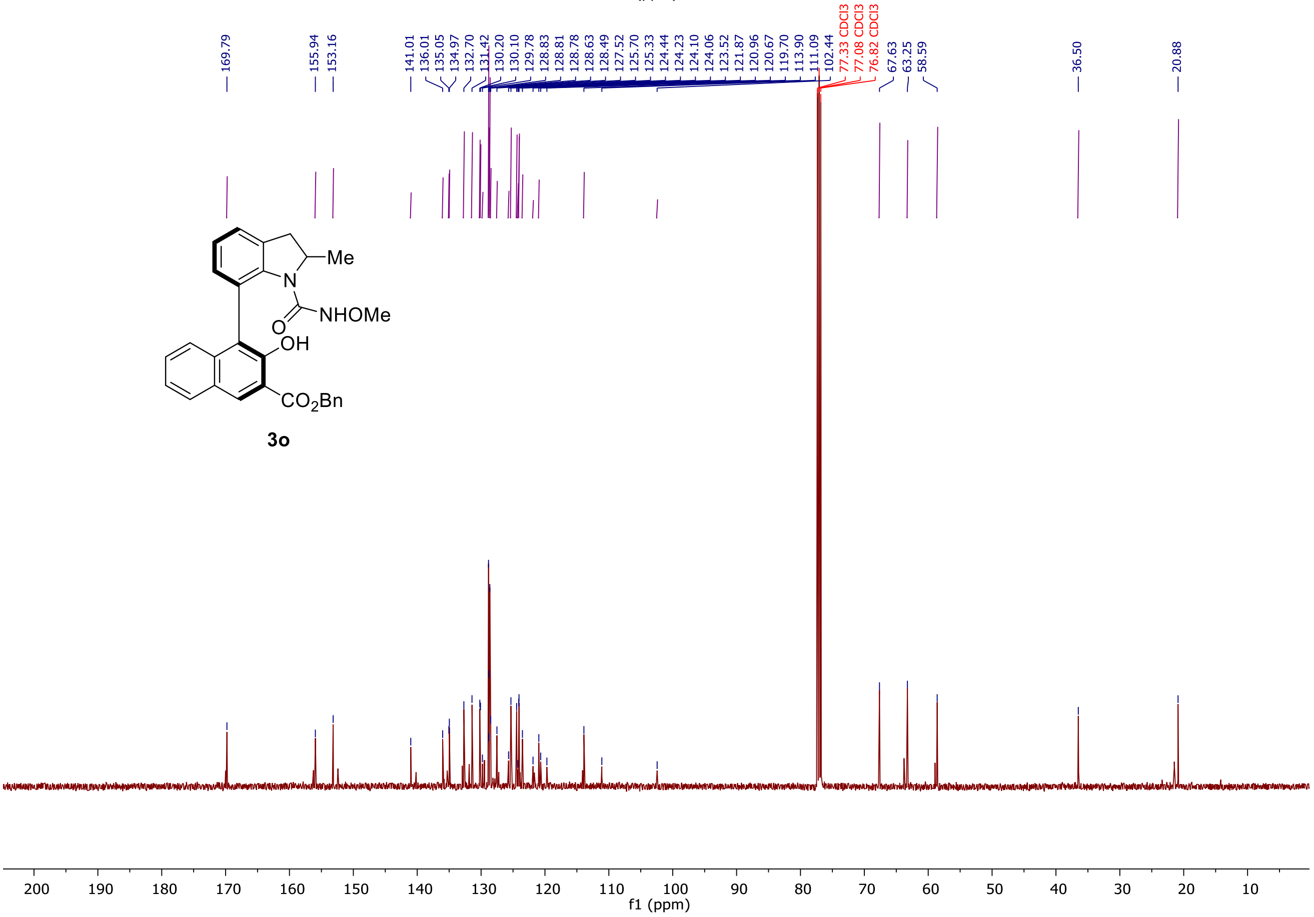
169.79
155.94
153.16
141.01
136.01
135.05
134.97
132.70
131.42
130.20
130.10
129.78
128.83
128.81
128.78
128.63
128.49
127.52
125.70
125.33
124.44
124.23
124.10
124.06
123.52
121.87
120.96
120.67
119.70
113.90
111.09
102.44
77.33 CDCl3
77.08 CDCl3
76.82 CDCl3
67.63
63.25
58.59
36.50
20.88
Me
N
NHOMe
O
OH
CO2Bn
3o
f1 (ppm)

HPLC traces (**3o**): racemate top, enantiomer bottom:

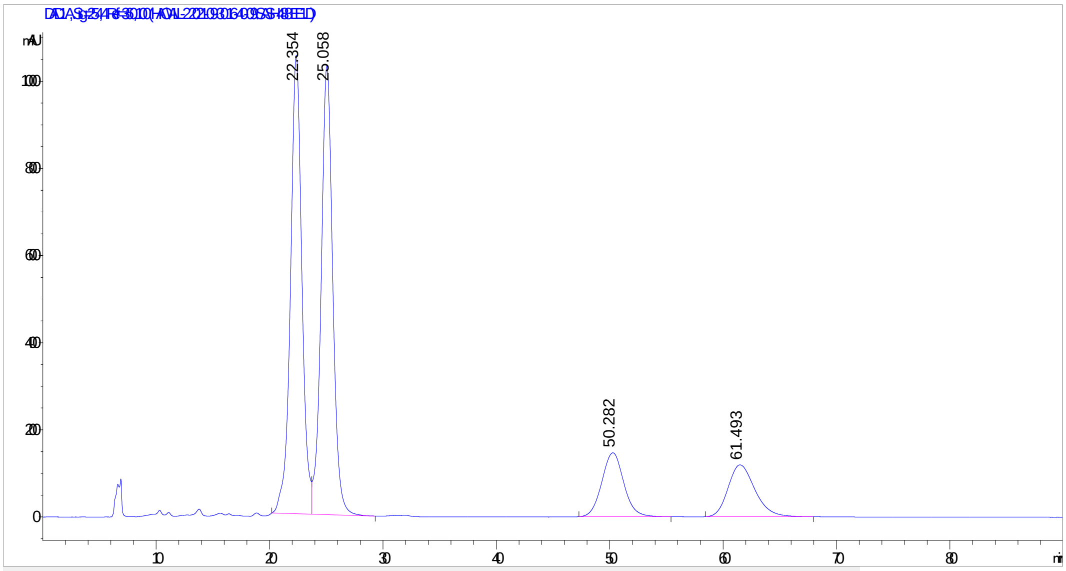

#	Time	Area	Height	Width	Area%	Symmetry
1	22.354	73396.8	1052.3	1.0557	39.430	1.002
2	25.058	73487.4	1032.4	1.0824	39.479	0.936
3	50.282	19713.1	146.6	1.9287	10.590	0.968
4	61.493	19547.5	118.9	2.1996	10.501	0.759

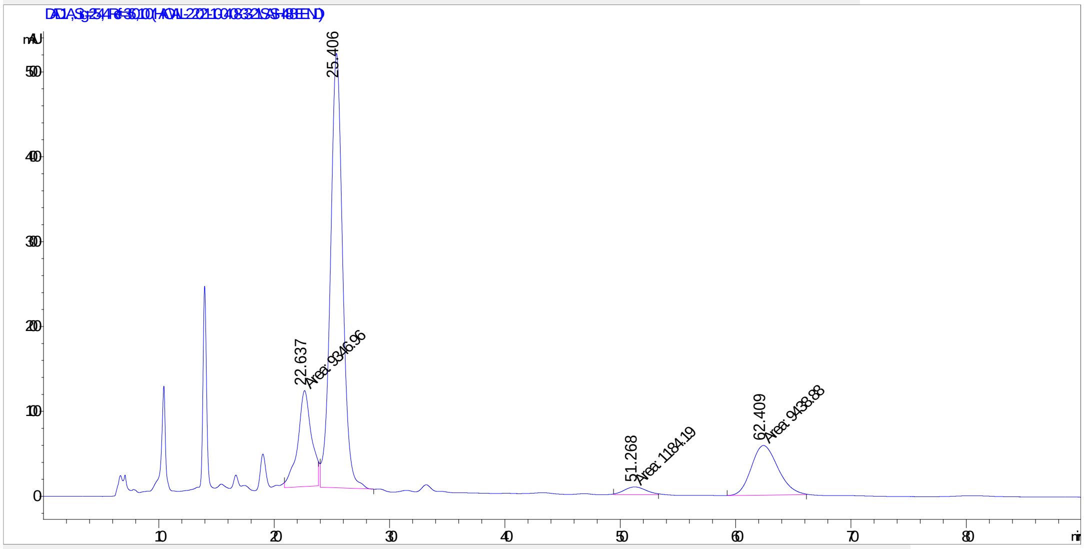

#	Time	Area	Height	Width	Area%	Symmetry
1	22.637	9347	113.1	1.3779	16.246	0.977
2	25.406	37563.7	511.7	1.1164	65.290	0.937
3	51.268	1184.2	9.1	2.158	2.058	0.933
4	62.409	9438.9	58.5	2.6883	16.406	0.806

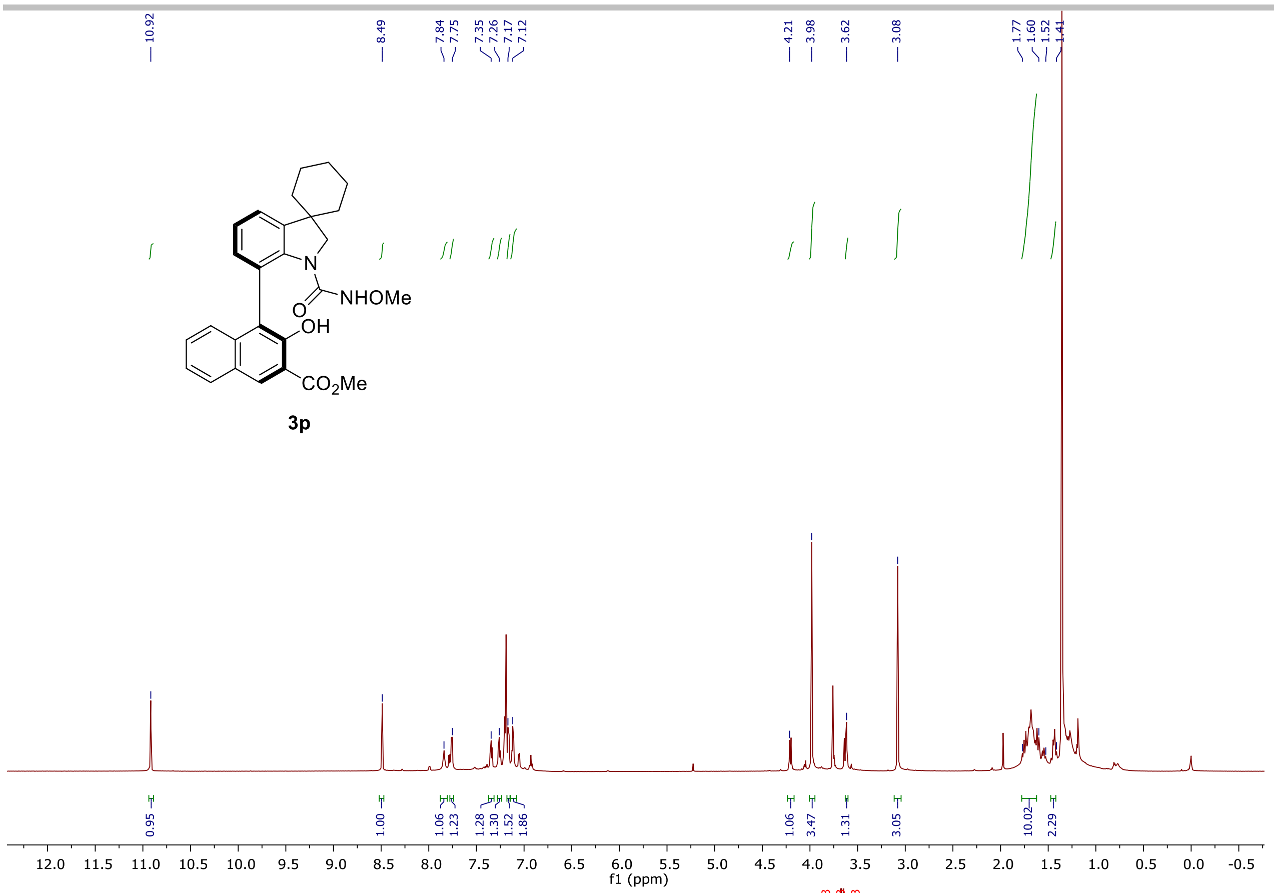
10.92
8.49
7.84
7.75
7.35
7.26
7.17
7.12
4.21
3.98
3.62
3.08
1.77
1.60
1.52
1.41
N
NHOMe
O
OH
CO2Me
3p
0.95
1.00
1.06
1.23
1.28
1.30
1.52
1.86
1.06
3.47
1.31
3.05
10.02
2.29
12.0 11.5 11.0 10.5 10.0 9.5 9.0 8.5 8.0 7.5 7.0 6.5 6.0 5.5 5.0 4.5 4.0 3.5 3.0 2.5 2.0 1.5 1.0 0.5 0.0 -0.5
f1 (ppm)

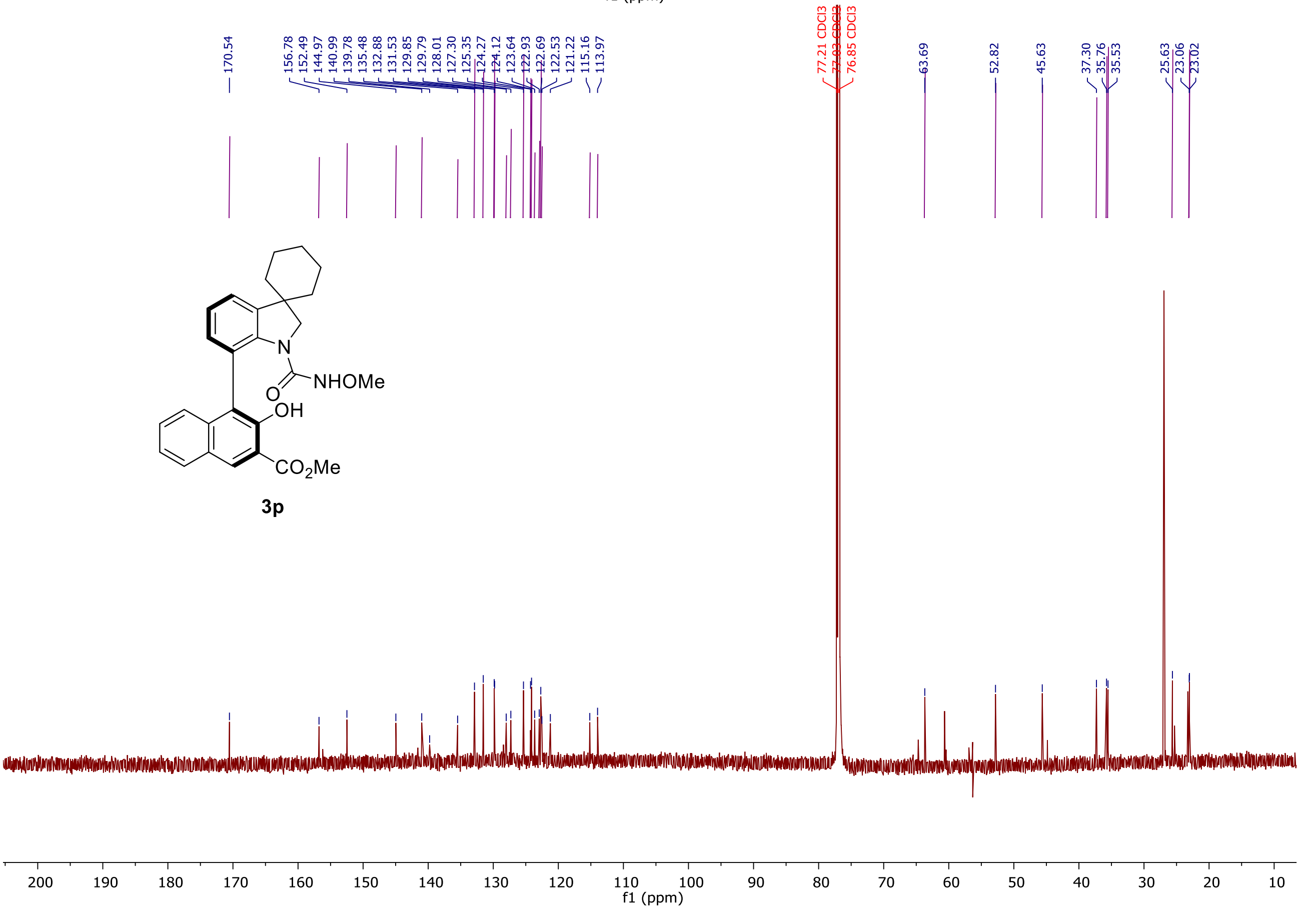
170.54
156.78
152.49
144.97
140.99
139.78
135.48
132.88
131.53
129.85
129.79
128.01
127.30
125.35
124.27
124.12
123.64
122.93
122.69
122.53
121.22
115.16
113.97
77.21 CDCl3
76.85 CDCl3
63.69
52.82
45.63
37.30
35.76
35.53
25.63
23.06
23.02
N
NHOMe
O
OH
CO2Me
3p
200 190 180 170 160 150 140 130 120 110 100 90 80 70 60 50 40 30 20 10
f1 (ppm)

HPLC traces (**3p**): racemate top, enantiomer bottom:

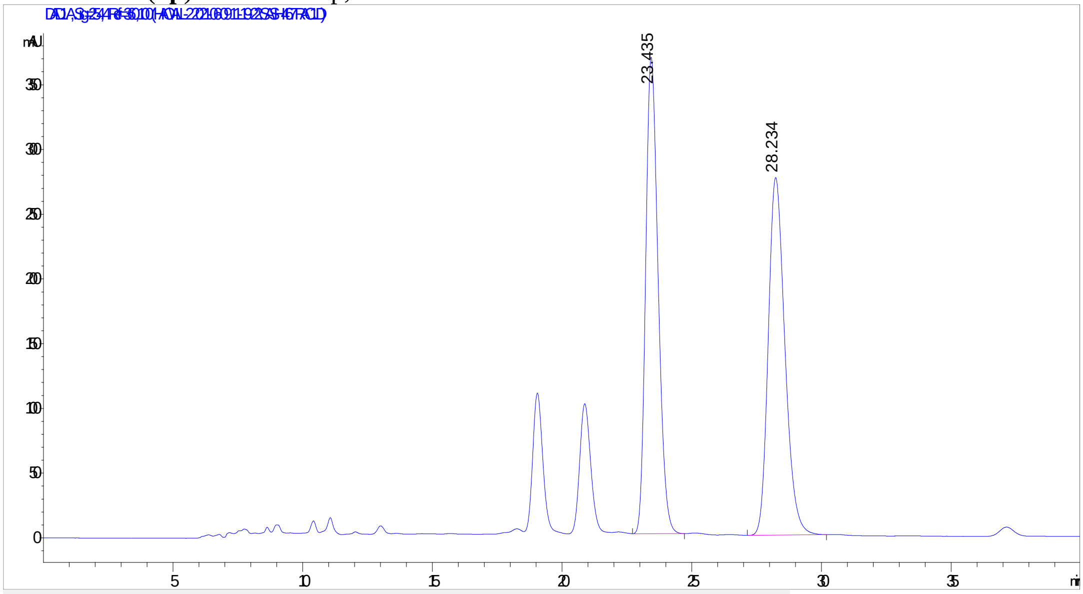

#	Time	Area	Height	Width	Area%	Symmetry
1	23.435	12254.9	368.2	0.5183	49.864	0.748
2	28.234	12321.7	276.3	0.6883	50.136	0.751

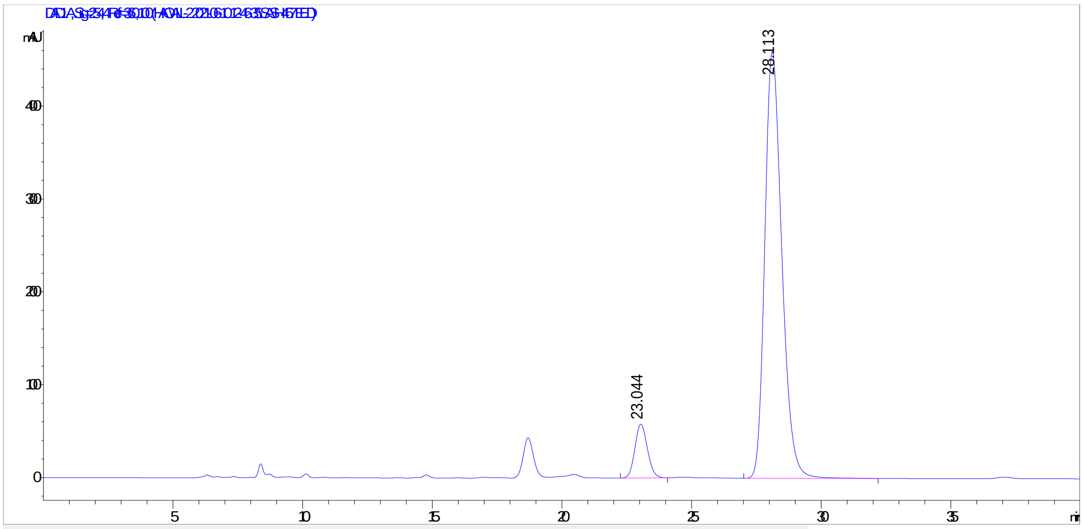

#	Time	Area	Height	Width	Area%	Symmetry
1	23.044	1935.1	57.8	0.5184	8.441	0.867
2	28.113	20989.9	459.9	0.7045	91.559	0.726

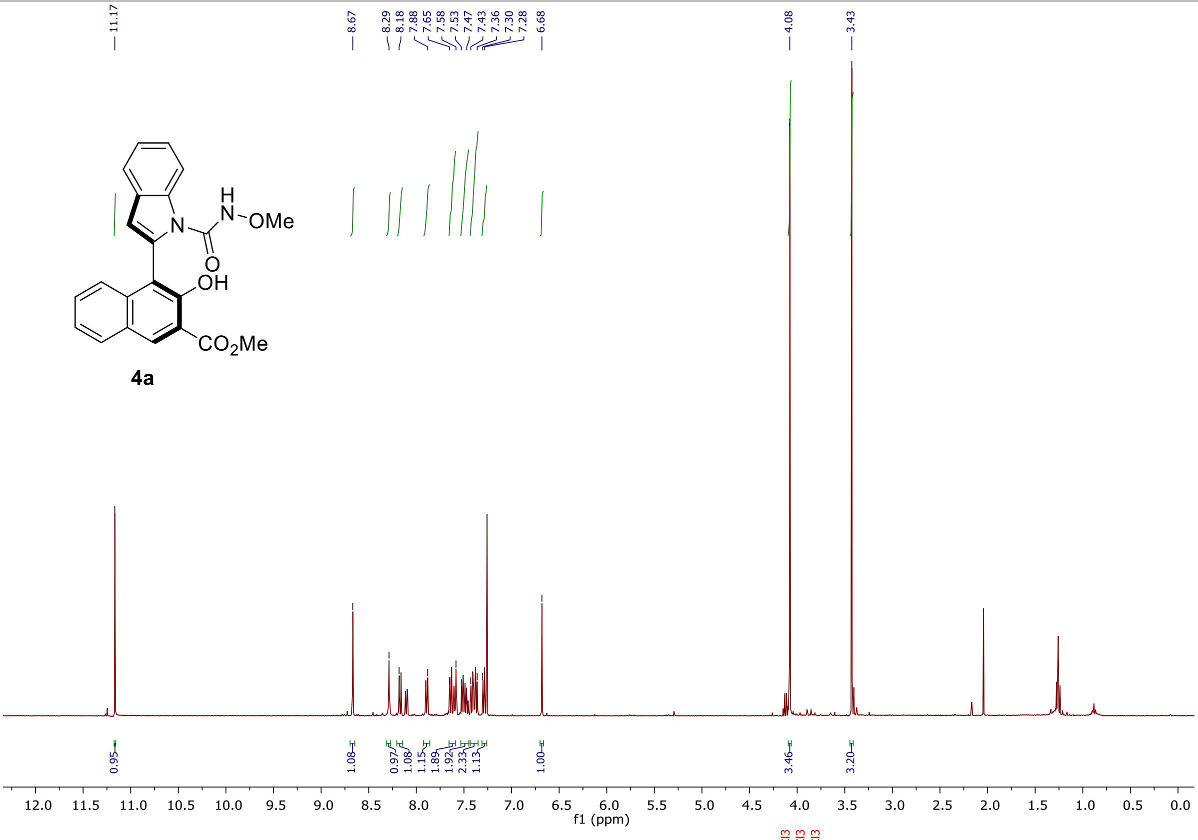
11.17
8.67
8.29
8.18
7.88
7.65
7.58
7.53
7.47
7.43
7.36
7.30
7.28
6.68
4.08
3.43
N
H
N
OMe
O
OH
CO2Me
4a
0.95
1.08
0.97
1.08
1.15
1.89
1.92
2.33
1.13
1.00
3.46
3.20
f1 (ppm)

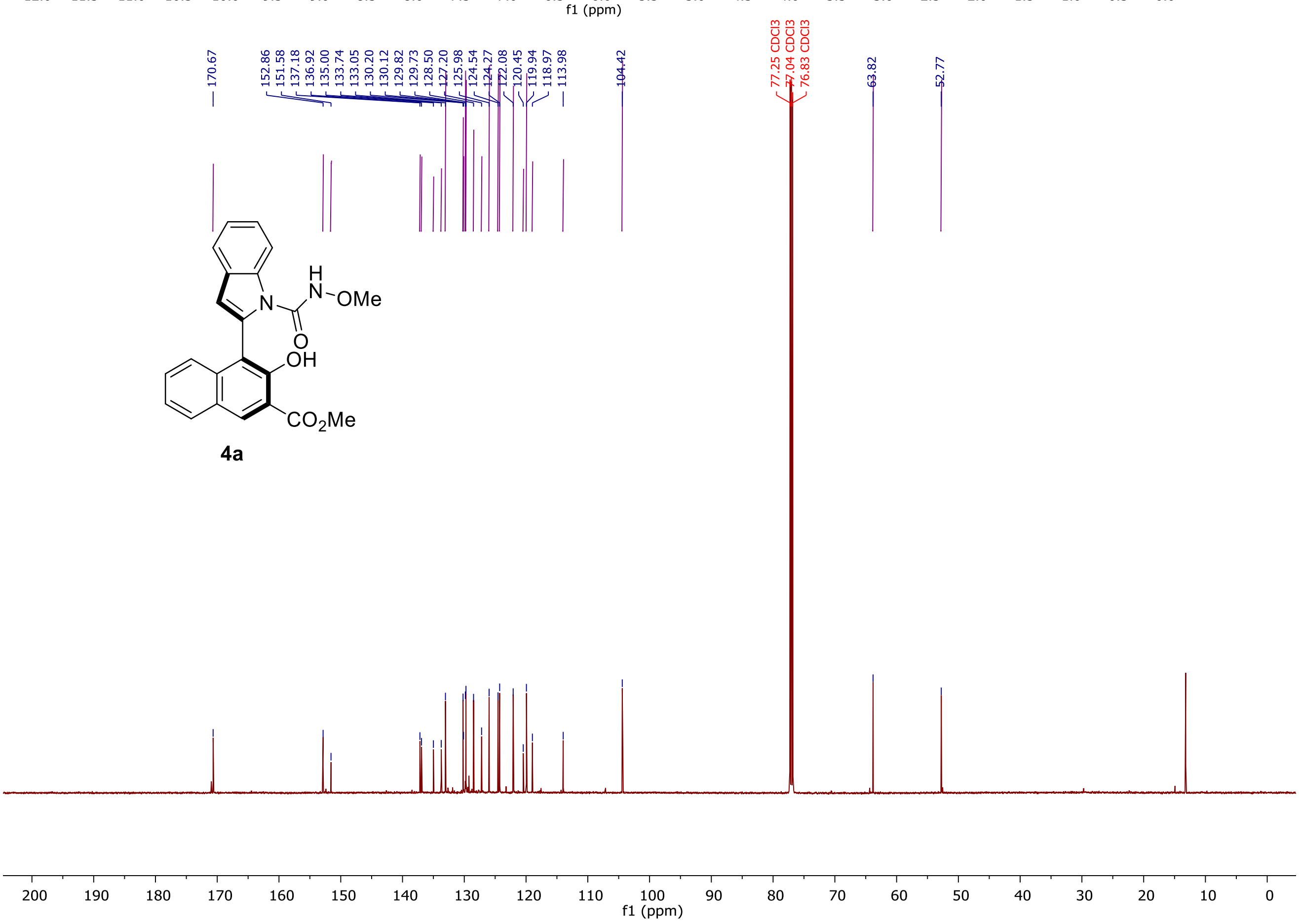
170.67
152.86
151.58
137.18
136.92
135.00
133.74
133.05
130.20
130.12
129.82
129.73
128.50
127.20
125.98
124.54
124.27
122.08
120.45
119.94
118.97
113.98
104.42
77.25 CDCl3
77.04 CDCl3
76.83 CDCl3
63.82
52.77
N
H
N
OMe
O
OH
CO2Me
4a
f1 (ppm)

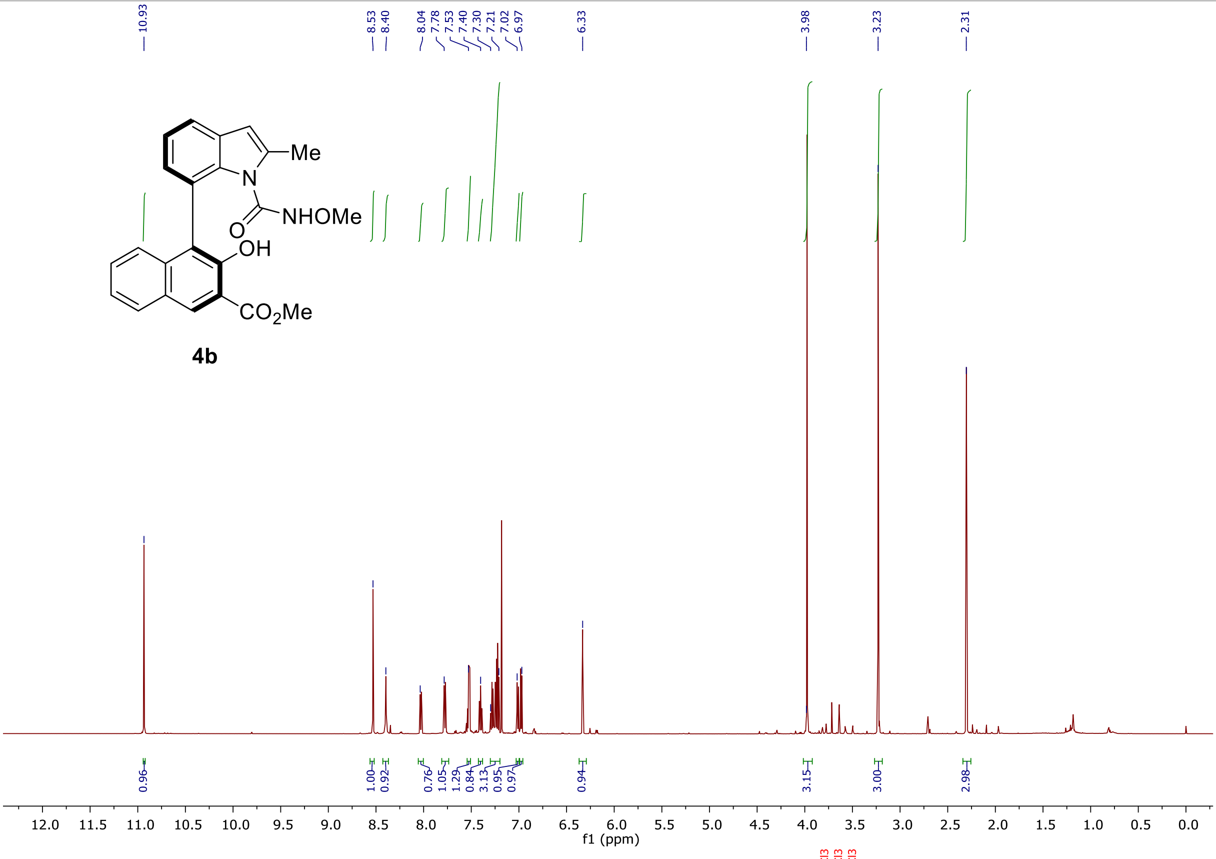

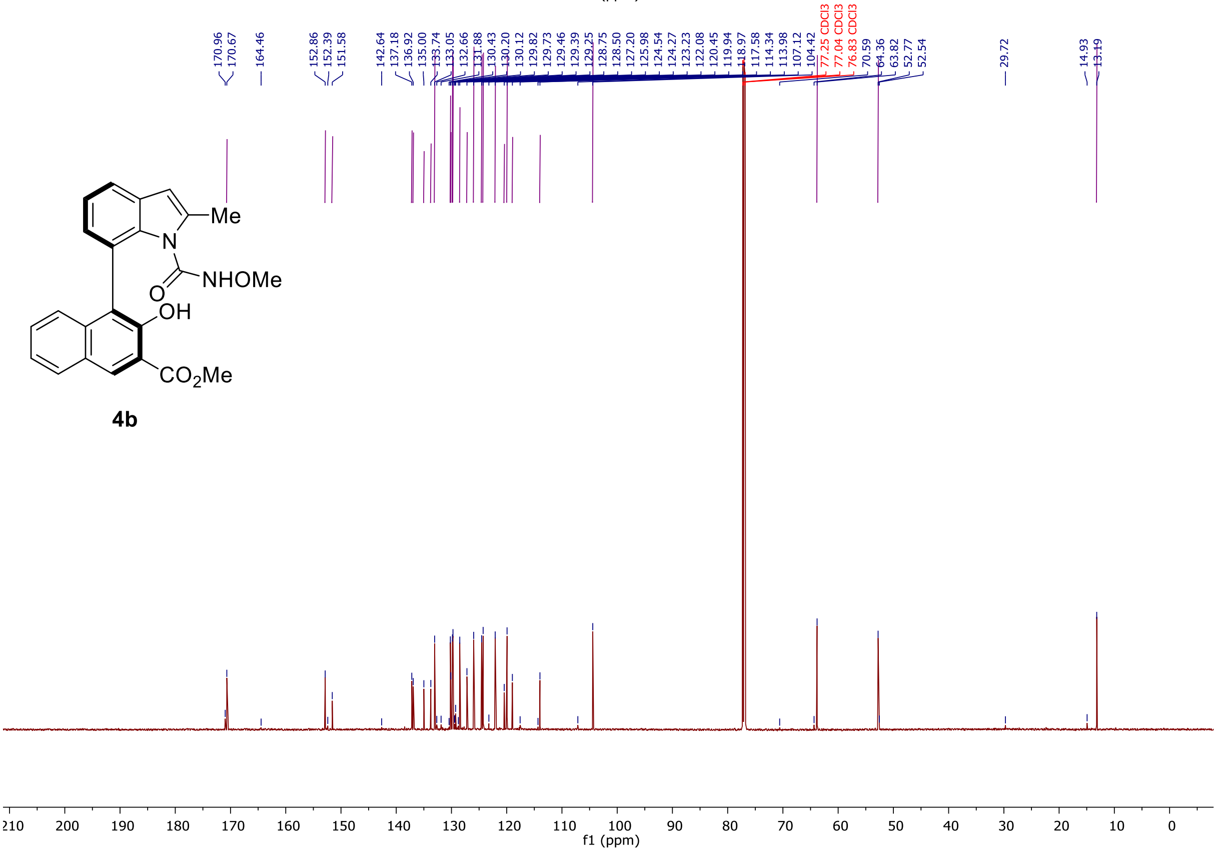

HPLC traces (**4b**): racemate top, enantiomer bottom:

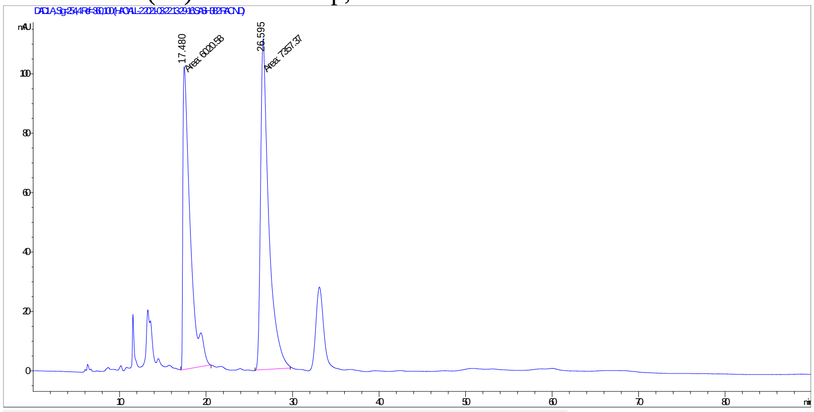

#	Time	Area	Height	Width	Area%	Symmetry
1	17.48	6143.2	102.1	1.0033	45.123	0.218
2	26.595	7471.1	111.6	0.9288	54.877	0.408

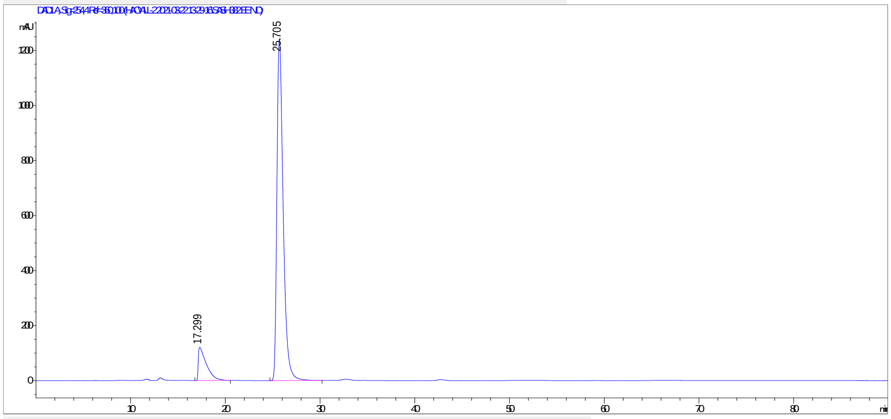

#	Time	Area	Height	Width	Area%	Symmetry
1	17.299	7118.7	120.2	0.833	11.026	0.198
2	25.705	57446.4	1242.3	0.7035	88.974	0.591

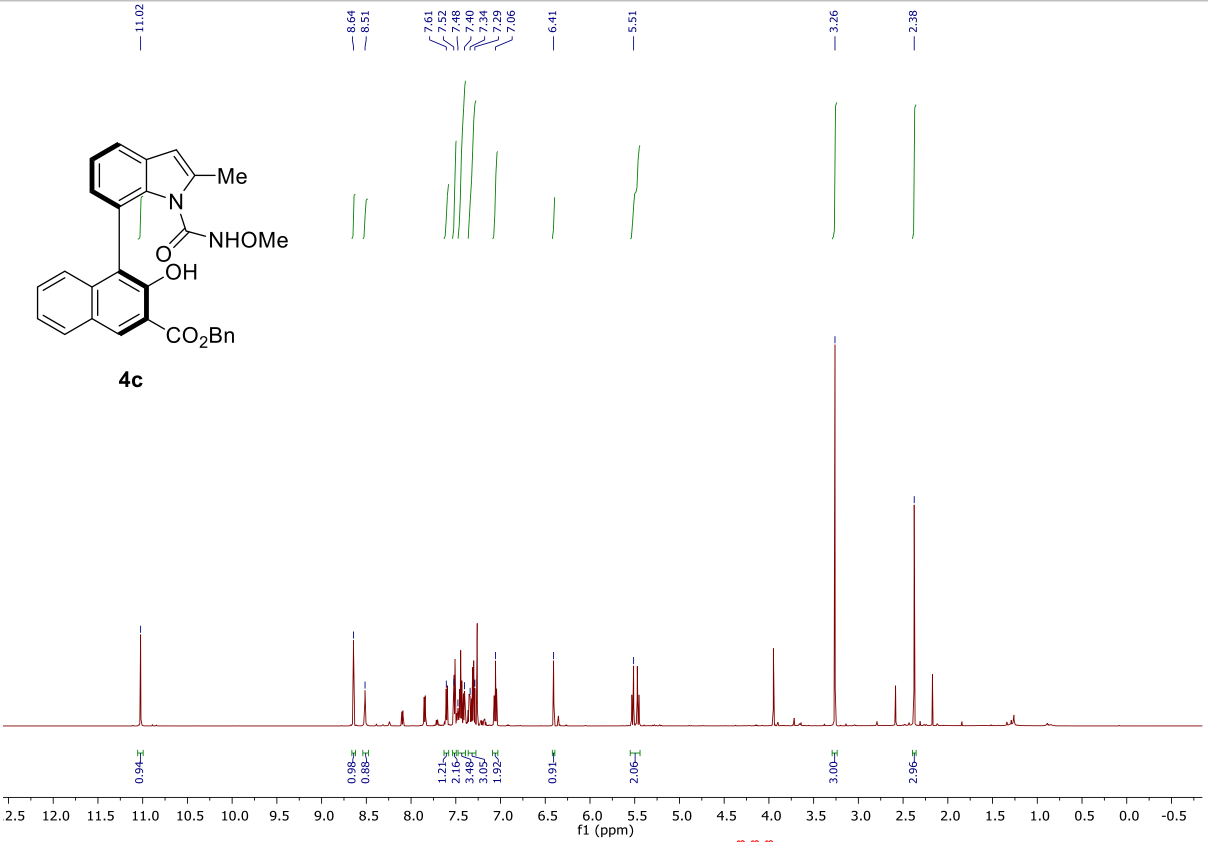
11.02
8.64
8.51
7.61
7.52
7.48
7.40
7.34
7.29
7.06
6.41
5.51
3.26
2.38
Me
N
O
NHOMe
OH
CO2Bn
4c
0.94
0.98
0.88
1.21
2.16
3.48
3.05
1.92
0.91
2.06
3.00
2.96
f1 (ppm)

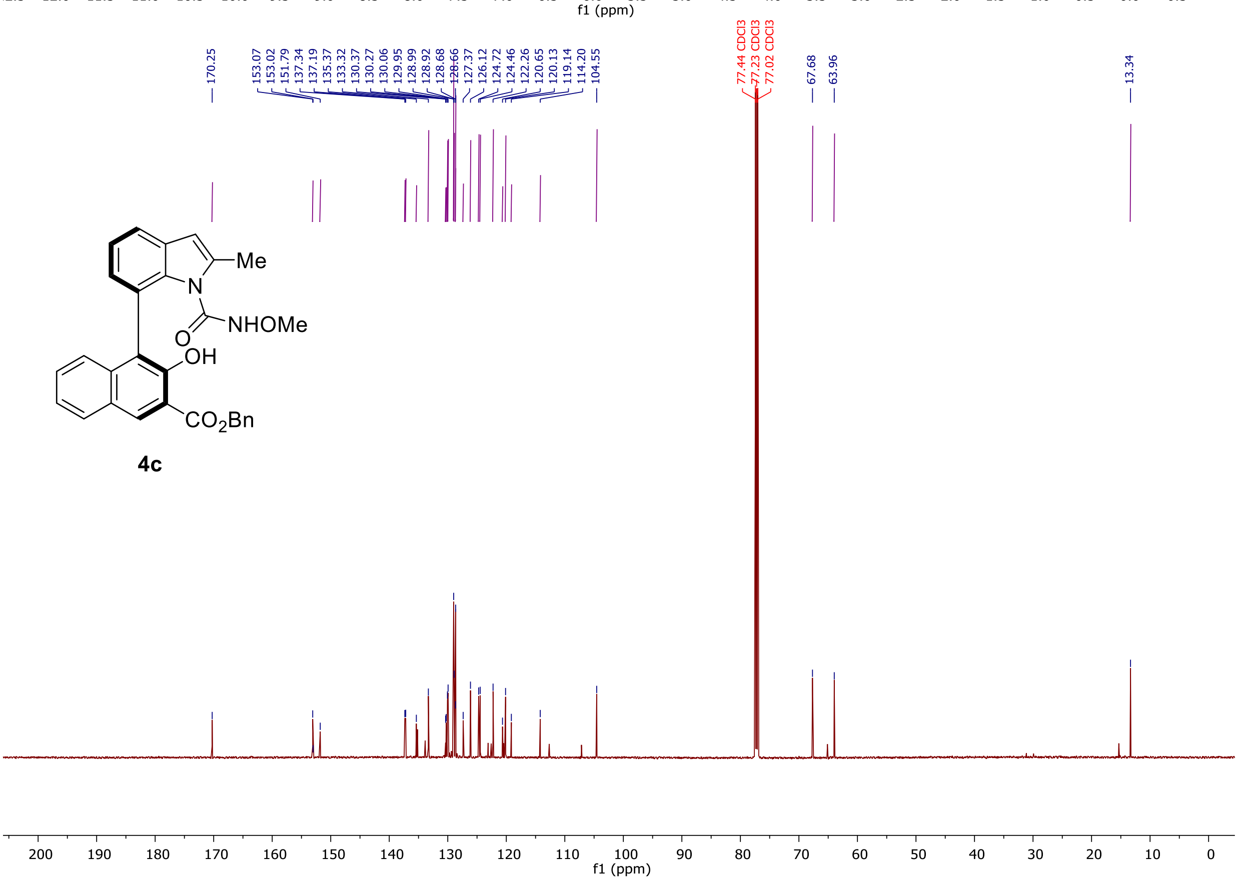
170.25
153.07
153.02
151.79
137.34
137.19
135.37
133.32
130.37
130.27
130.06
129.95
128.99
128.92
128.68
128.66
127.37
126.12
124.72
124.46
122.26
120.65
120.13
119.14
114.20
104.55
77.44 CDCl3
77.23 CDCl3
77.02 CDCl3
67.68
63.96
13.34
Me
N
O
NHOMe
OH
CO2Bn
4c
f1 (ppm)

HPLC traces (**4c**): racemate top, enantiomer bottom:

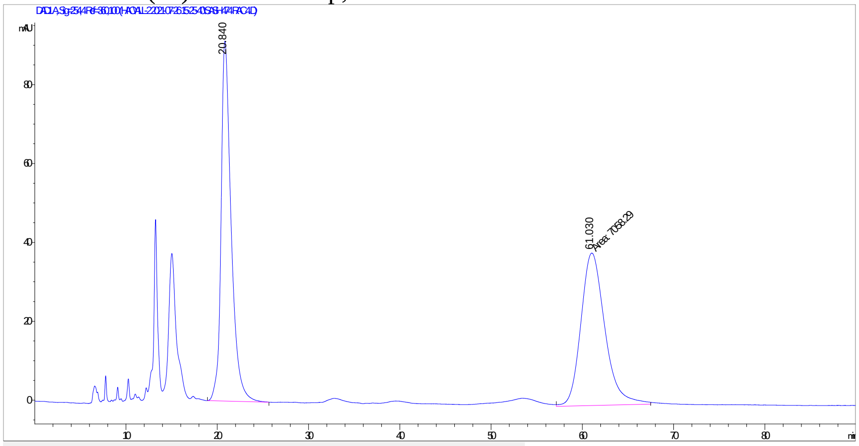

#	Time	Area	Height	Width	Area%	Symmetry
1	20.84	7008.6	91.3	1.1271	49.869	0.596
2	61.03	7045.4	38.7	3.037	50.131	0.799

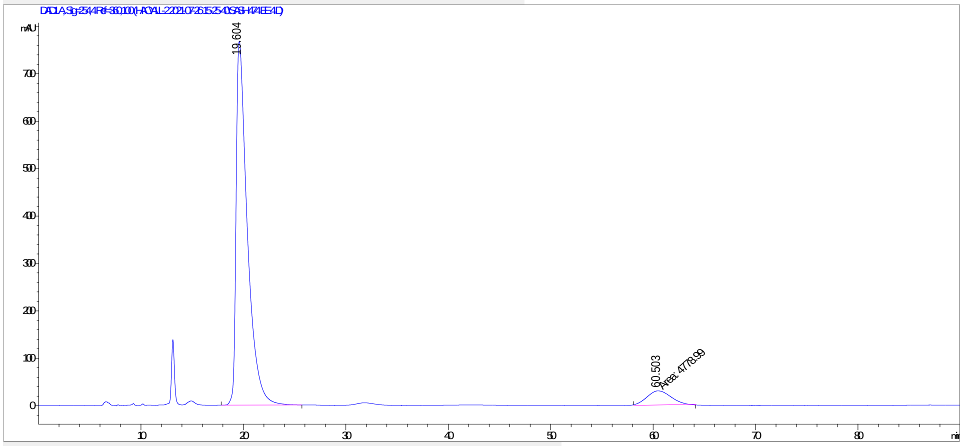

#	Time	Area	Height	Width	Area%	Symmetry
1	19.604	58840.1	770.5	1.2728	91.162	0.389
2	60.503	5704.8	32.1	2.9579	8.838	0.883

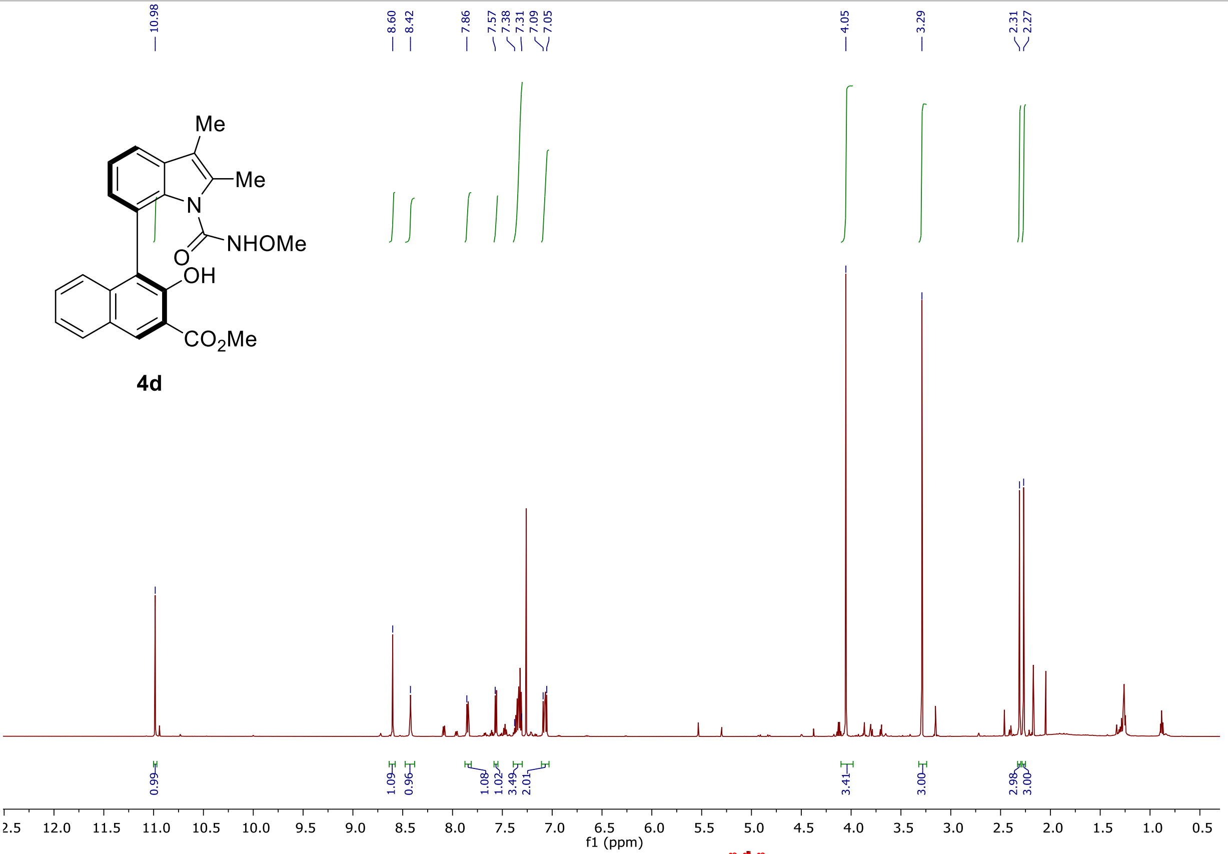
10.98
8.60
8.42
7.86
7.57
7.38
7.31
7.09
7.05
4.05
3.29
2.31
2.27
Me
Me
N
NHOMe
O
OH
CO2Me
0.99
1.09
0.96
1.08
1.02
3.49
2.01
3.41
3.00
2.98
3.00
f1 (ppm)

4d

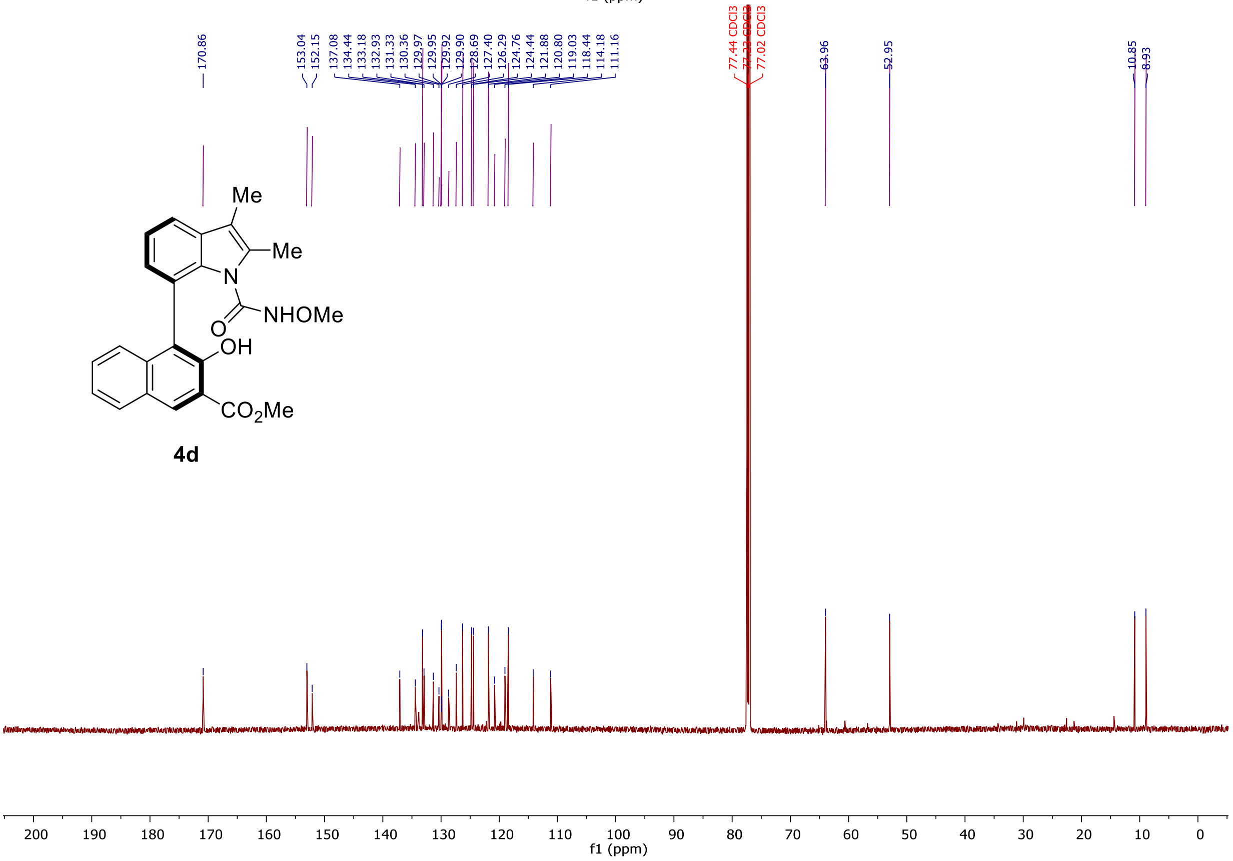
170.86
153.04
152.15
137.08
134.44
133.18
132.93
131.33
130.36
129.97
129.95
129.92
129.90
128.69
127.40
126.29
124.76
124.44
121.88
120.80
119.03
118.44
114.18
111.16
77.44 CDCl3
77.02 CDCl3
63.96
52.95
10.85
8.93
Me
Me
N
NHOMe
O
OH
CO2Me
f1 (ppm)

4d

HPLC traces (**4d**): racemate top, enantiomer bottom:

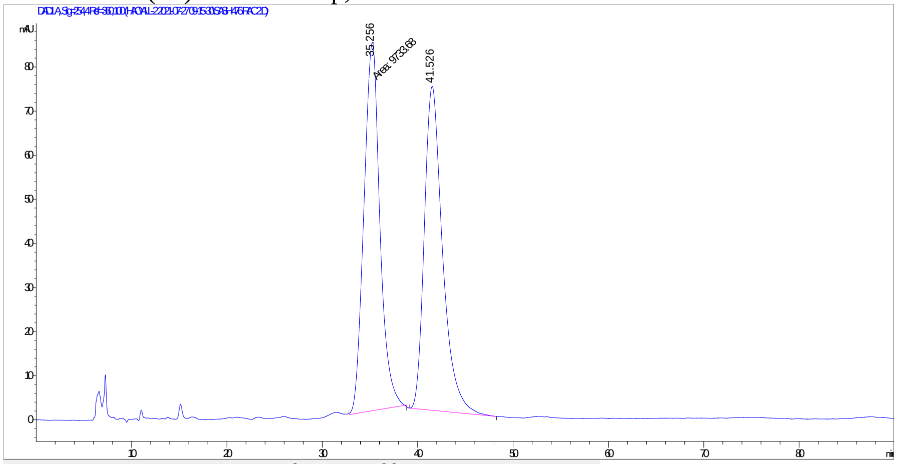

#	Time	Area	Height	Width	Area%	Symmetry
1	35.256	9926.5	84	1.9694	50.460	1.045
2	41.526	9745.5	73.4	1.8764	49.540	0.706

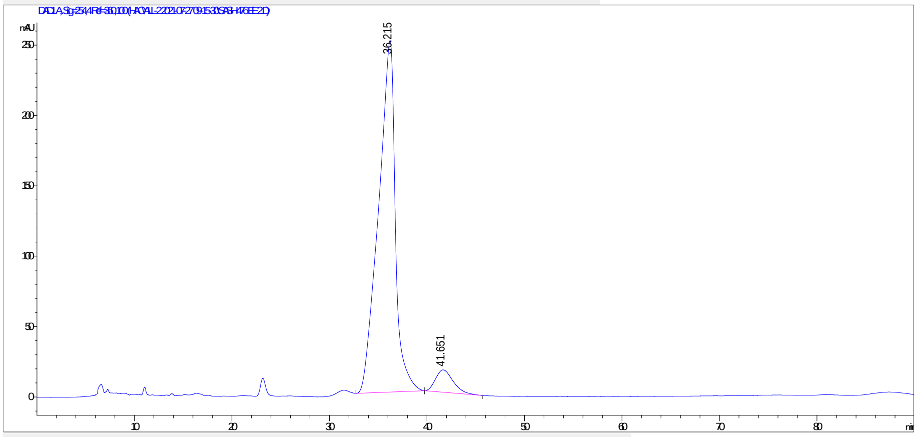

#	Time	Area	Height	Width	Area%	Symmetry
1	36.215	29999.3	249.7	1.6597	93.799	1.961
2	41.651	1983.1	15.9	1.4871	6.201	0.719

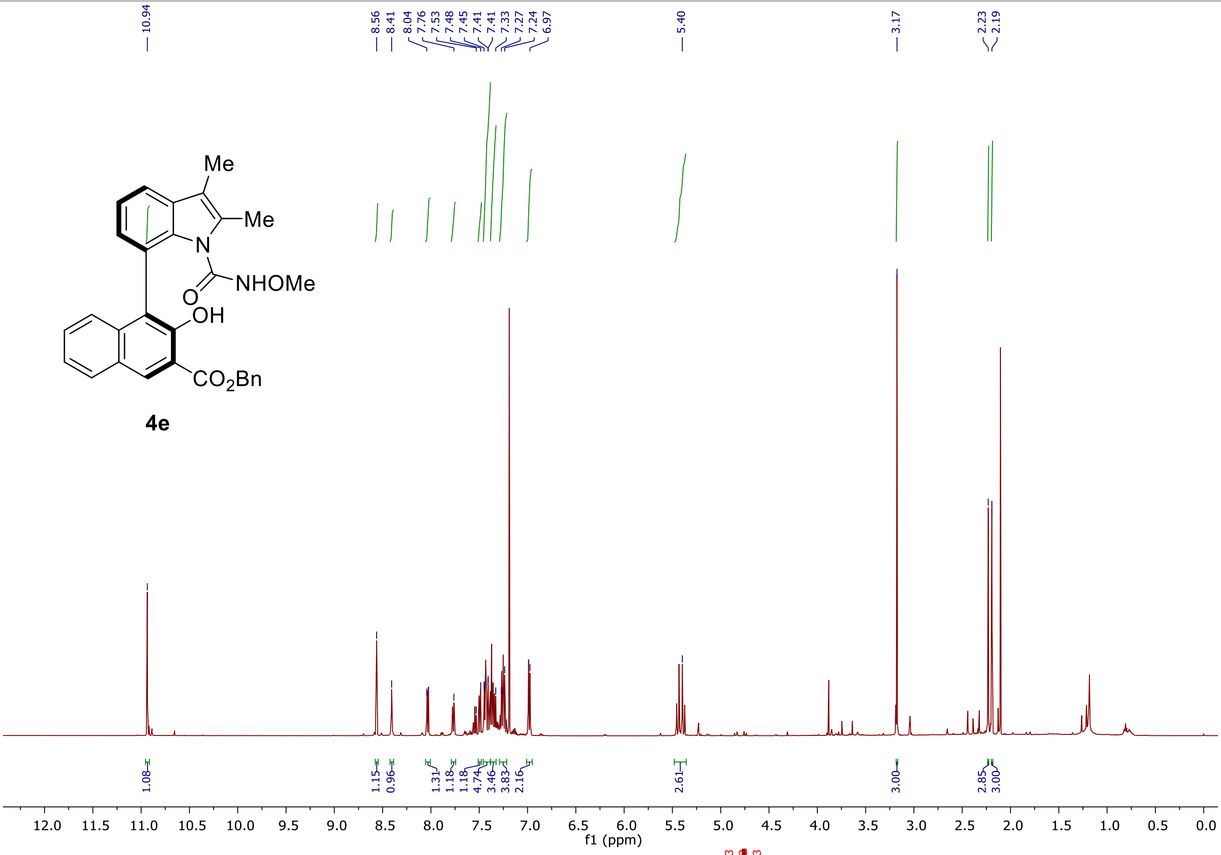
10.94
8.56
8.41
8.04
7.76
7.53
7.48
7.45
7.41
7.41
7.33
7.27
7.24
6.97
5.40
3.17
2.23
2.19
Me
Me
N
O
NHOMe
OH
CO2Bn
4e
1.08
1.15
0.96
1.31
1.18
1.18
4.74
3.46
3.83
2.16
2.61
3.00
2.85
3.00
f1 (ppm)

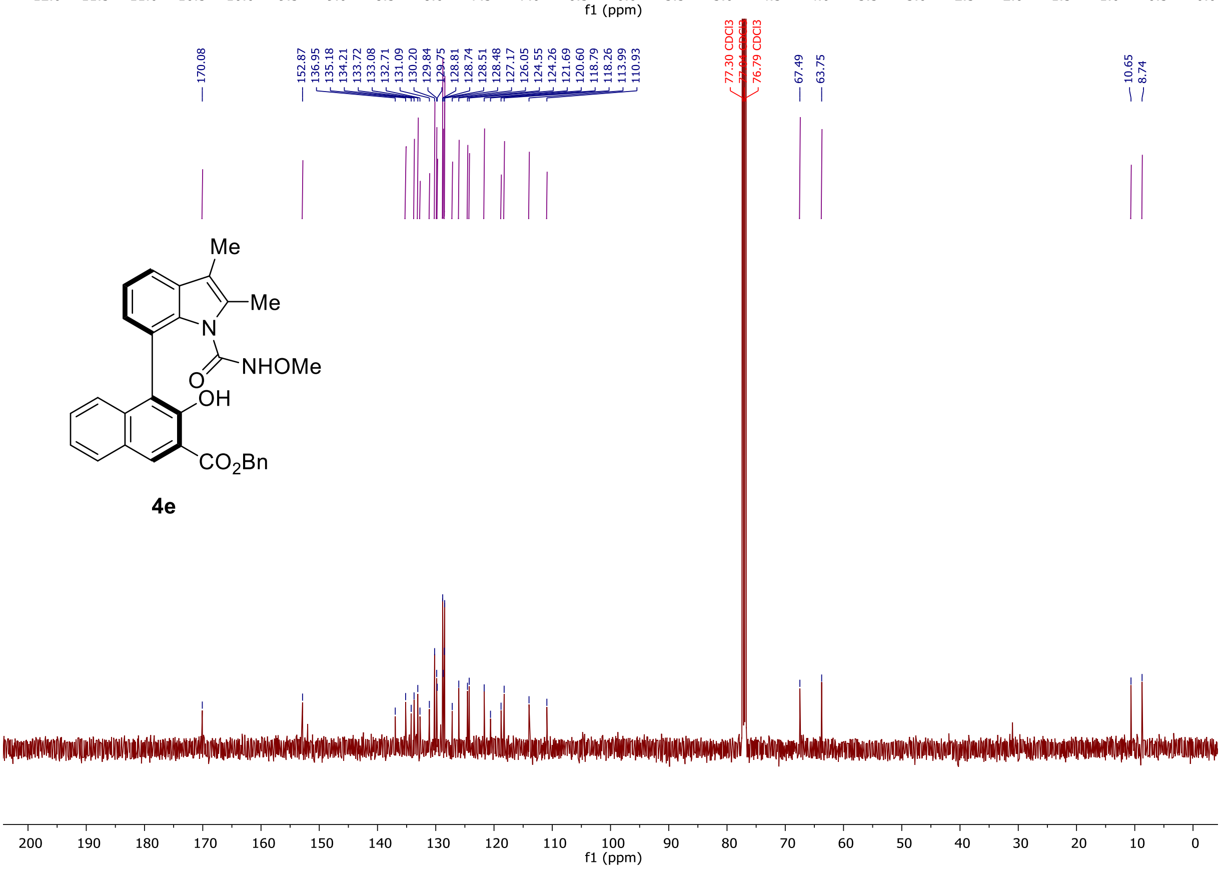
170.08
152.87
136.95
135.18
134.21
133.72
133.08
132.71
131.09
130.20
129.84
129.75
128.81
128.74
128.51
128.48
127.17
126.05
124.55
124.26
121.69
120.60
118.79
118.26
113.99
110.93
77.30 CDCl3
76.79 CDCl3
67.49
63.75
10.65
8.74
Me
Me
N
O
NHOMe
OH
CO2Bn
4e
f1 (ppm)

HPLC traces (**4e**): racemate top, enantiomer bottom:

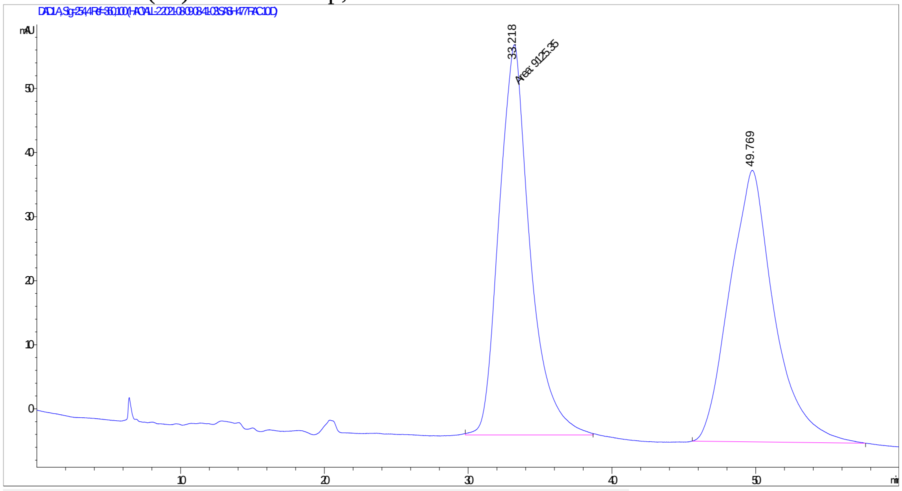

#	Time	Area	Height	Width	Area%	Symmetry
1	33.218	8817.4	60.4	2.4339	49.113	0.94
2	49.769	9135.7	42.4	2.5314	50.887	0.978

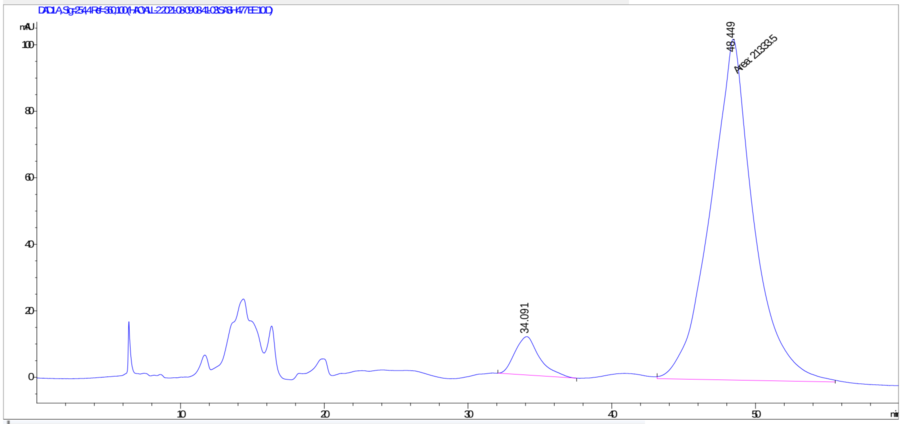

#	Time	Area	Height	Width	Area%	Symmetry
1	34.091	1373.1	11.5	1.414	6.047	0.871
2	48.449	21333.5	102.5	3.468	93.953	1.055

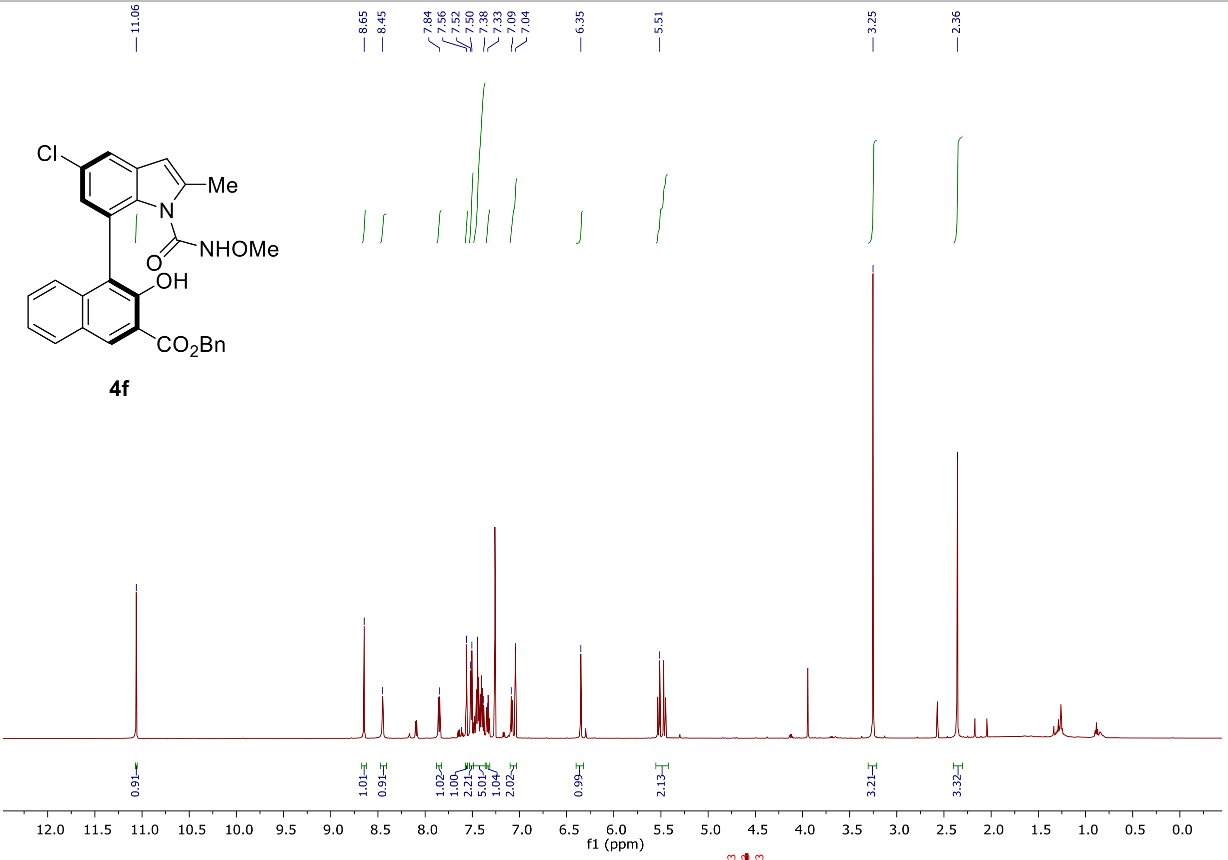
11.06
8.65
8.45
7.84
7.56
7.52
7.50
7.38
7.33
7.09
7.04
6.35
5.51
3.25
2.36
Cl
Me
N
NHOMe
O
OH
CO2Bn
4f
0.91
1.01
0.91
1.02
1.00
2.21
5.01
1.04
2.02
0.99
2.13
3.21
3.32
f1 (ppm)

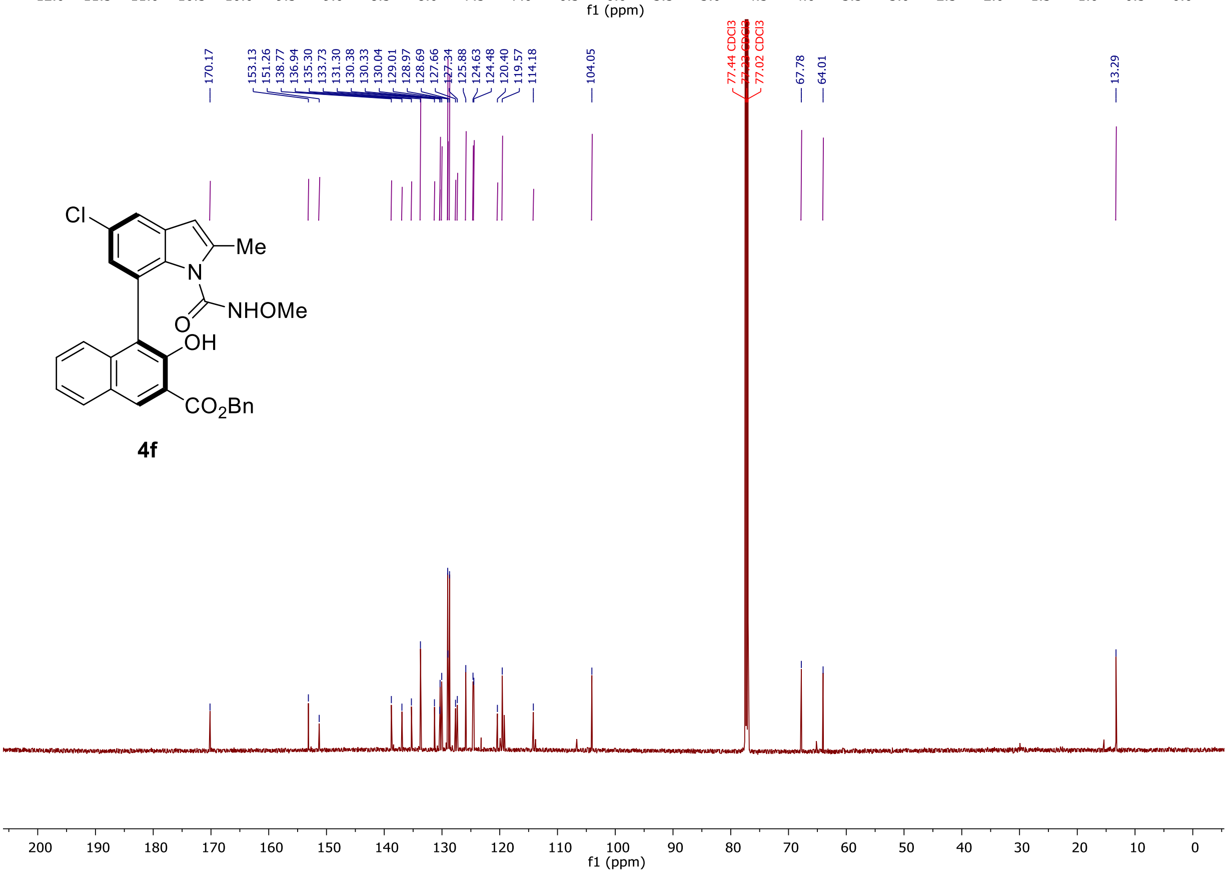
170.17
153.13
151.26
138.77
136.94
135.30
133.73
131.30
130.38
130.33
130.04
129.01
128.97
128.69
127.66
127.34
125.88
124.63
124.48
120.40
119.57
114.18
104.05
77.44 CDCl3
77.02 CDCl3
67.78
64.01
13.29
Cl
Me
N
NHOMe
O
OH
CO2Bn
4f
f1 (ppm)

HPLC traces (**4f**): racemate top, enantiomer bottom:

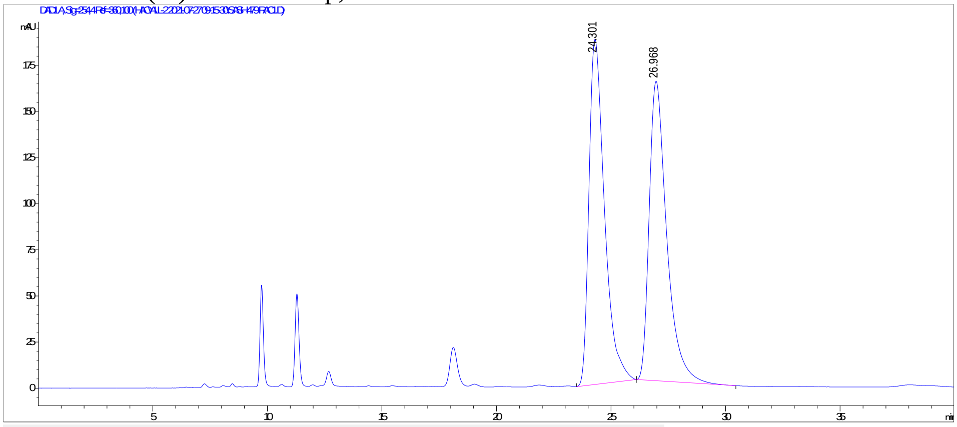

#	Time	Area	Height	Width	Area%	Symmetry
1	24.301	8804.3	187.2	0.7128	50.458	0.584
2	26.968	8644.4	162.4	0.8011	49.542	0.592

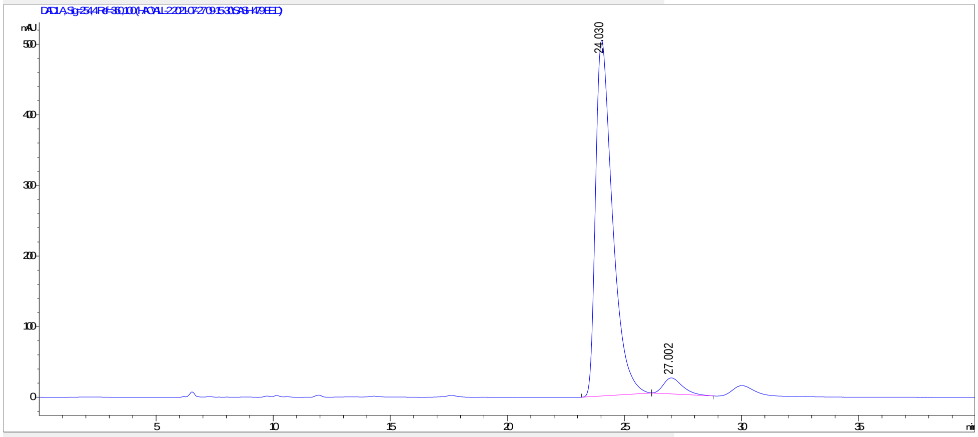

#	Time	Area	Height	Width	Area%	Symmetry
1	24.03	25372.1	504.1	0.7613	95.226	0.514
2	27.002	1271.9	22.6	0.8412	4.774	0.642

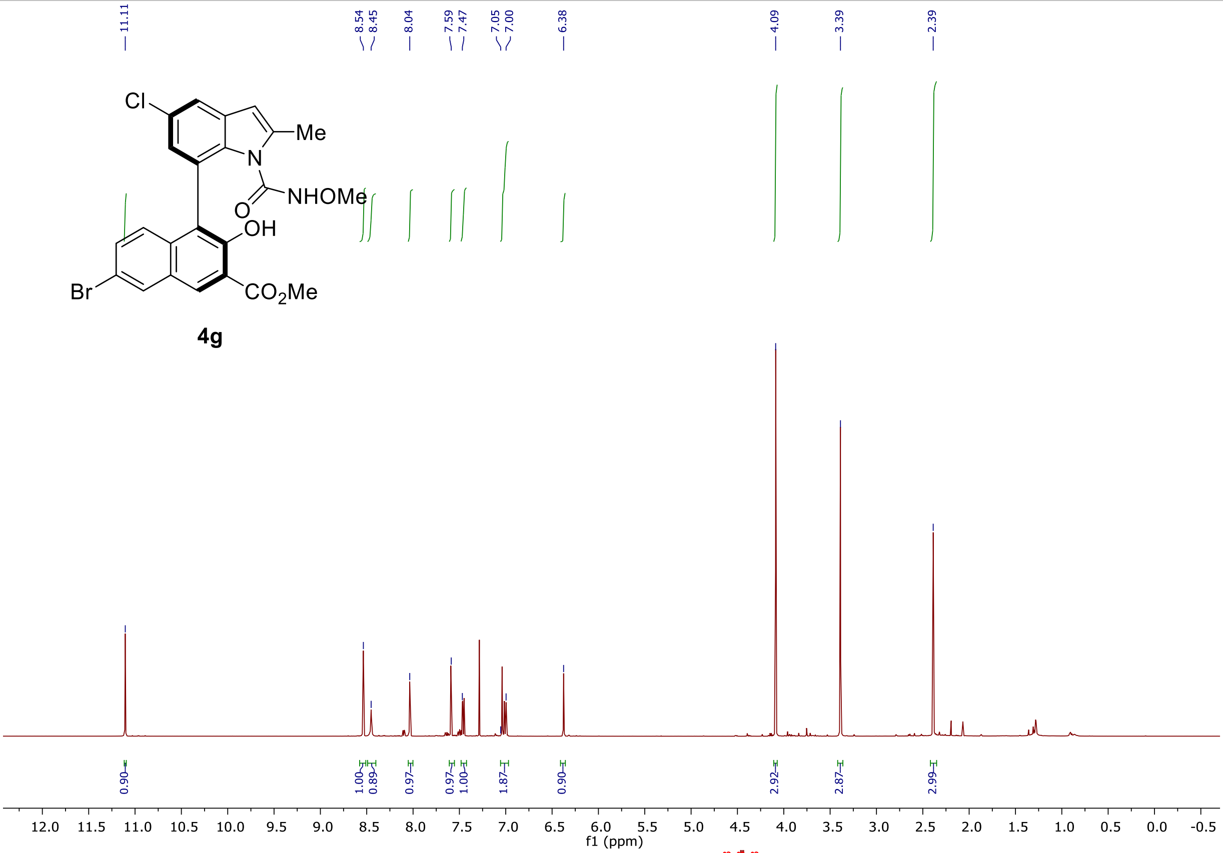
11.11
8.54
8.45
8.04
7.59
7.47
7.05
7.00
6.38
4.09
3.39
2.39
Cl
Me
N
NHOMe
O
OH
Br
CO2Me
4g
0.90
1.00
0.89
0.97
0.97
1.00
1.87
0.90
2.92
2.87
2.99
12.0 11.5 11.0 10.5 10.0 9.5 9.0 8.5 8.0 7.5 7.0 6.5 6.0 5.5 5.0 4.5 4.0 3.5 3.0 2.5 2.0 1.5 1.0 0.5 0.0 -0.5
f1 (ppm)

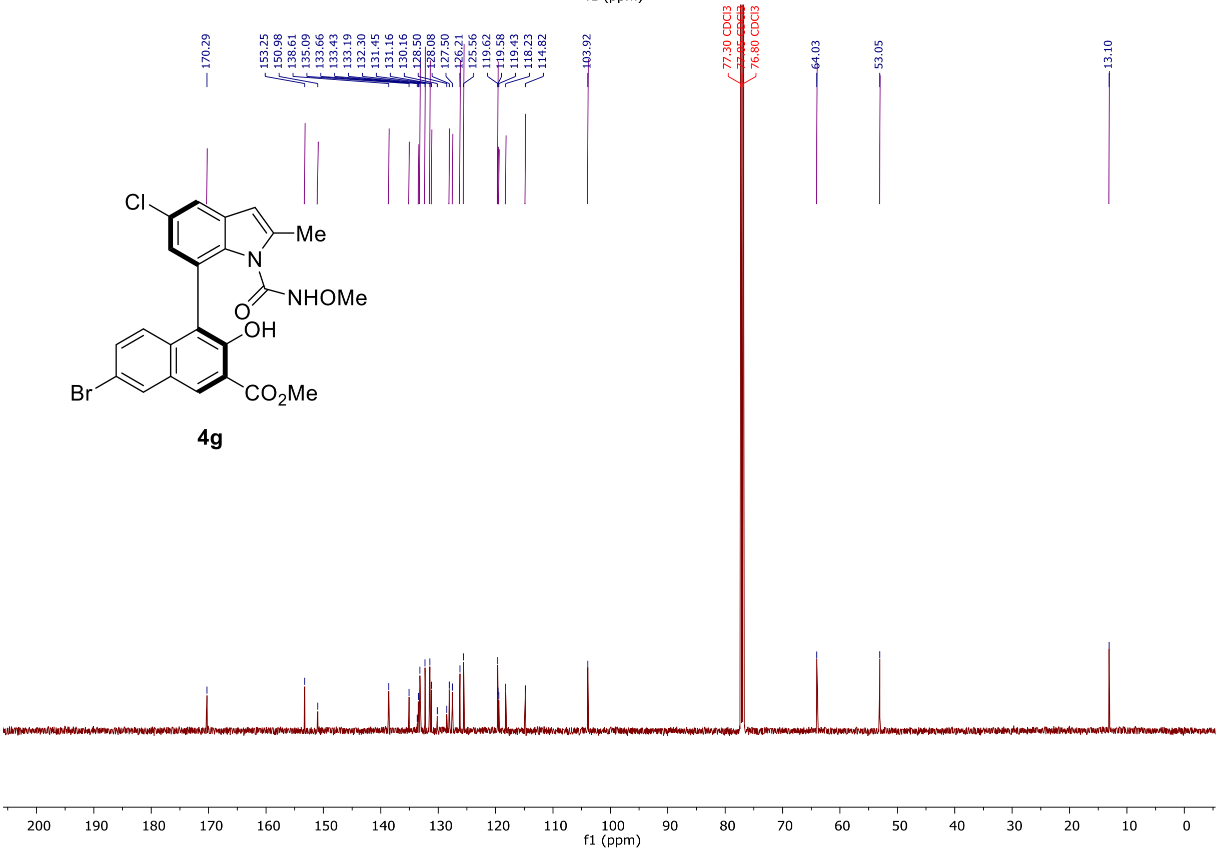
170.29
153.25
150.98
138.61
135.09
133.66
133.43
133.19
132.30
131.45
131.16
130.16
128.50
128.08
127.50
126.21
125.56
119.62
119.58
119.43
118.23
114.82
103.92
77.30 CDCl3
76.80 CDCl3
64.03
53.05
13.10
Cl
Me
N
NHOMe
O
OH
Br
CO2Me
4g
200 190 180 170 160 150 140 130 120 110 100 90 80 70 60 50 40 30 20 10 0
f1 (ppm)

HPLC traces (**4g**): racemate top, enantiomer bottom:

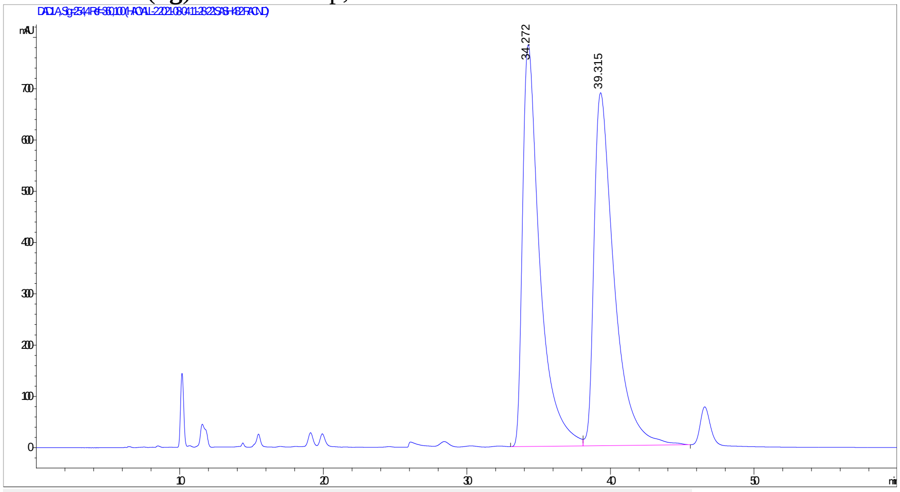

#	Time	Area	Height	Width	Area%	Symmetry
1	34.272	65794.4	783.5	1.2277	49.468	0.441
2	39.315	67210.8	688.3	1.4172	50.532	0.447

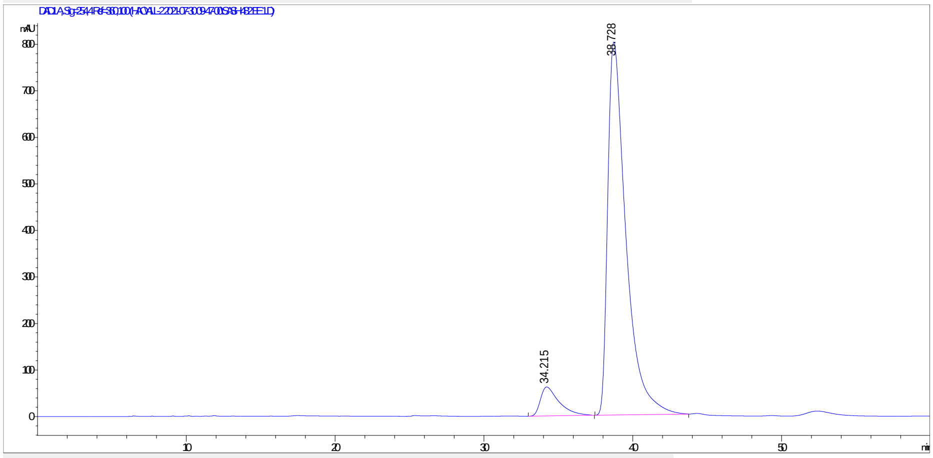

#	Time	Area	Height	Width	Area%	Symmetry
1	34.215	5405.4	62.5	1.2458	7.334	0.471
2	38.728	68299.6	801.9	1.2617	92.666	0.462

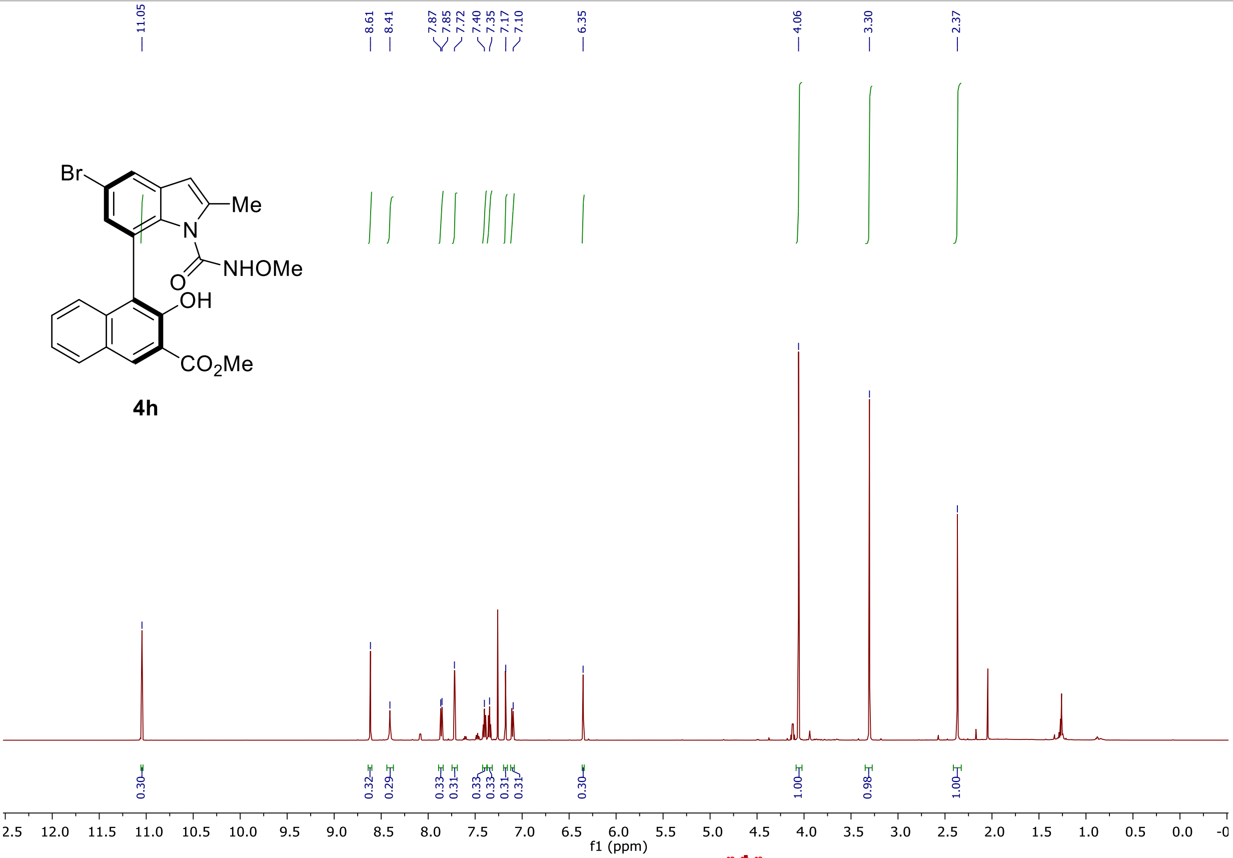
Br
Me
N
NHOMe
O
OH
CO2Me
4h
f1 (ppm)

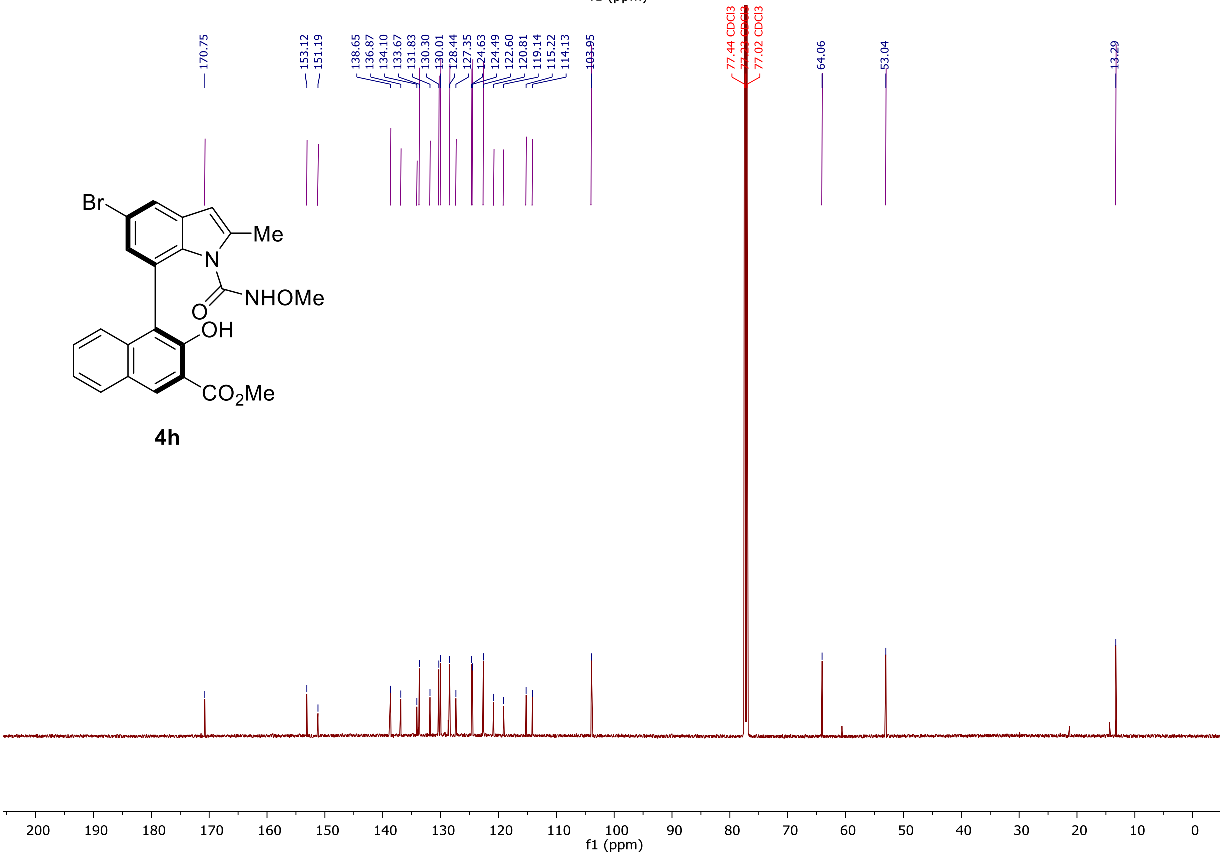
Br
Me
N
NHOMe
O
OH
CO2Me
4h
f1 (ppm)

HPLC traces (**4h**): racemate top, enantiomer bottom:

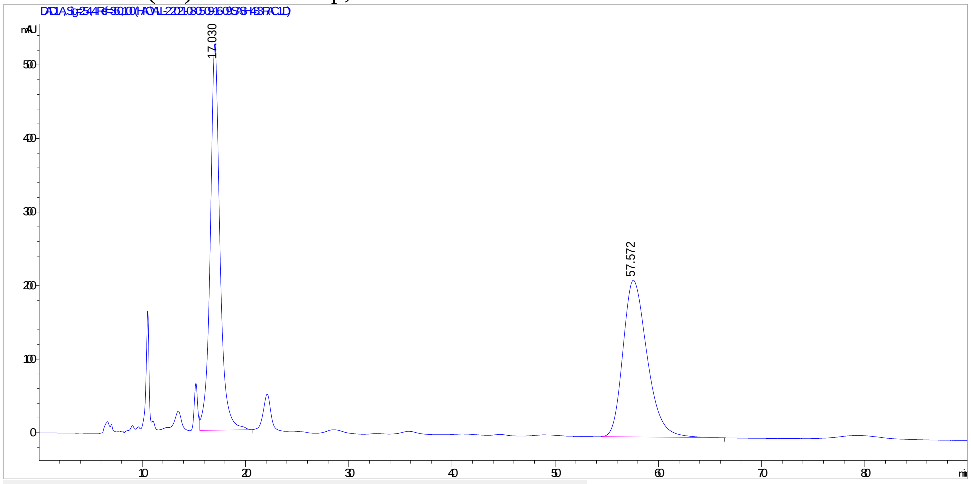

#	Time	Area	Height	Width	Area%	Symmetry
1	17.03	34174.4	525.4	0.9548	49.692	0.867
2	57.572	34598.2	213	2.203	50.308	0.707

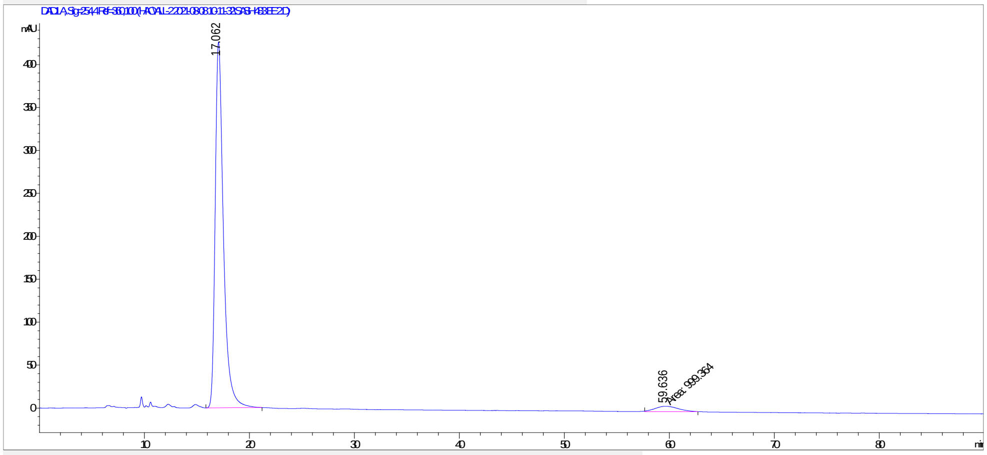

#	Time	Area	Height	Width	Area%	Symmetry
1	17.062	23271.4	426.2	0.821	95.561	0.622
2	59.636	1081.1	6.5	2.7524	4.439	0.819

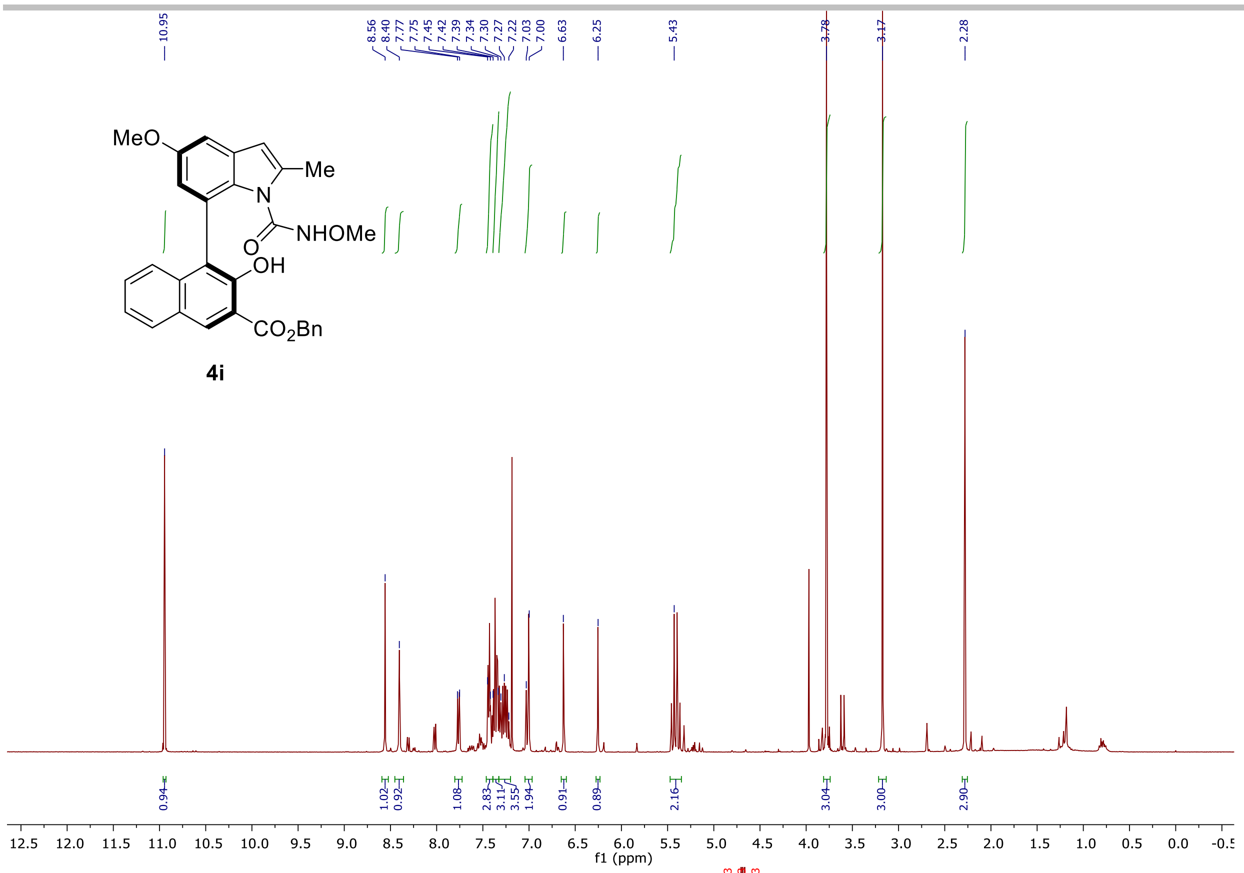
MeO
Me
N
NHOMe
O
OH
CO2Bn
4i
f1 (ppm)

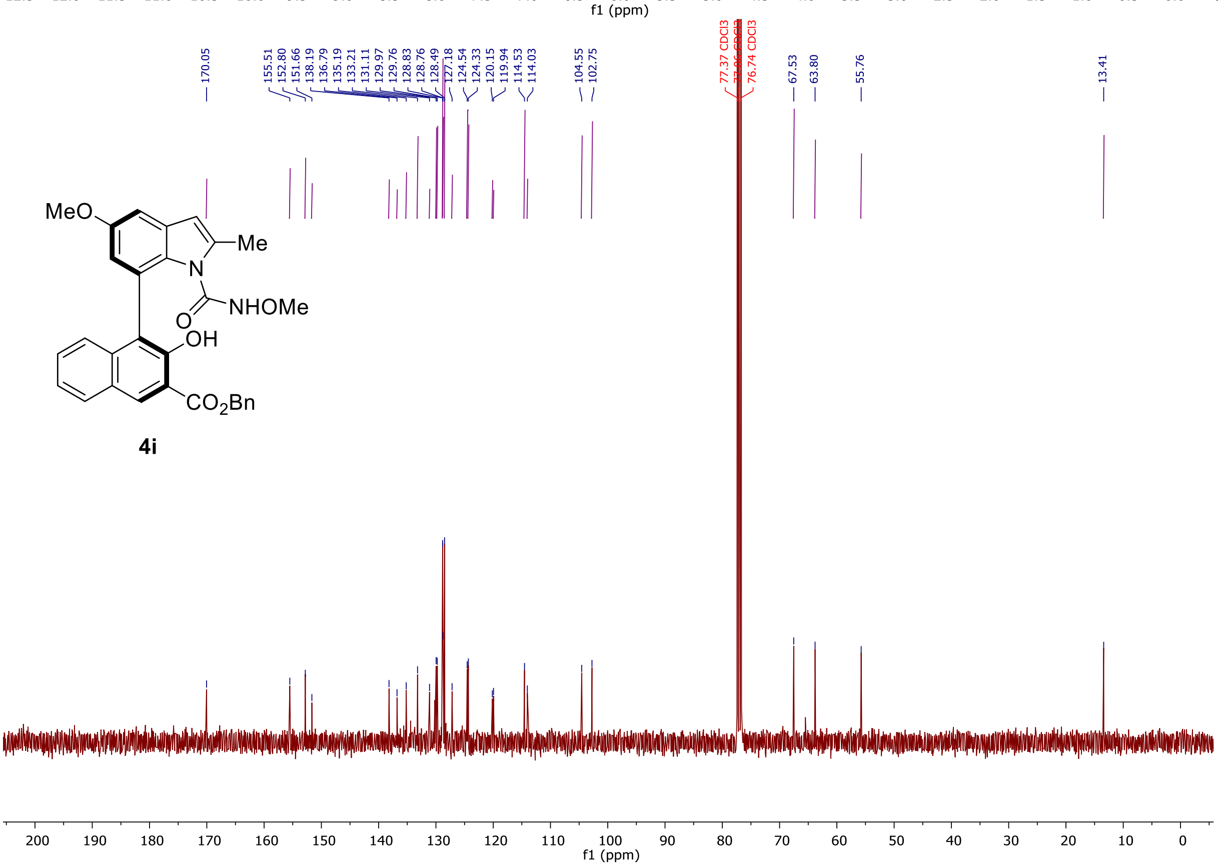
MeO
Me
N
NHOMe
O
OH
CO2Bn
4i
f1 (ppm)

HPLC traces (**4i**): racemate top, enantiomer bottom:

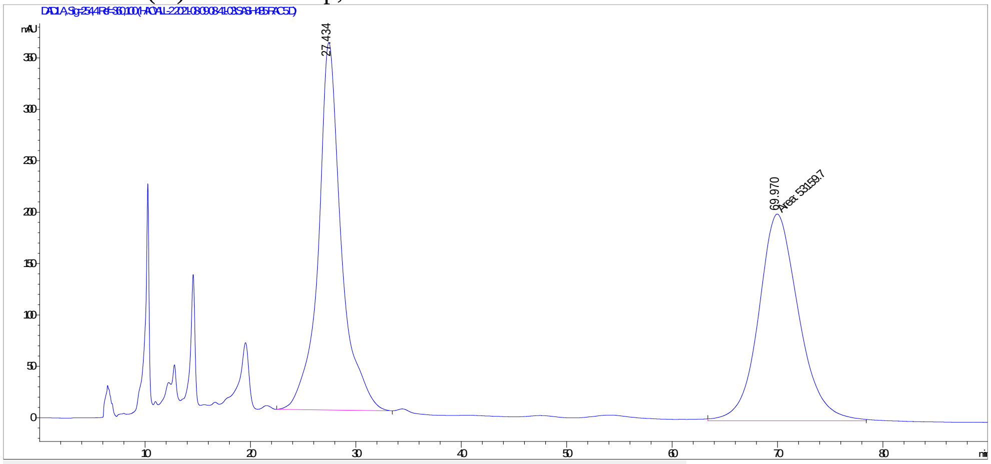

#	Time	Area	Height	Width	Area%	Symmetry
1	27.434	54185.1	357.7	2.1114	51.504	0.83
2	69.968	51019.9	198.6	4.2823	48.496	0.83

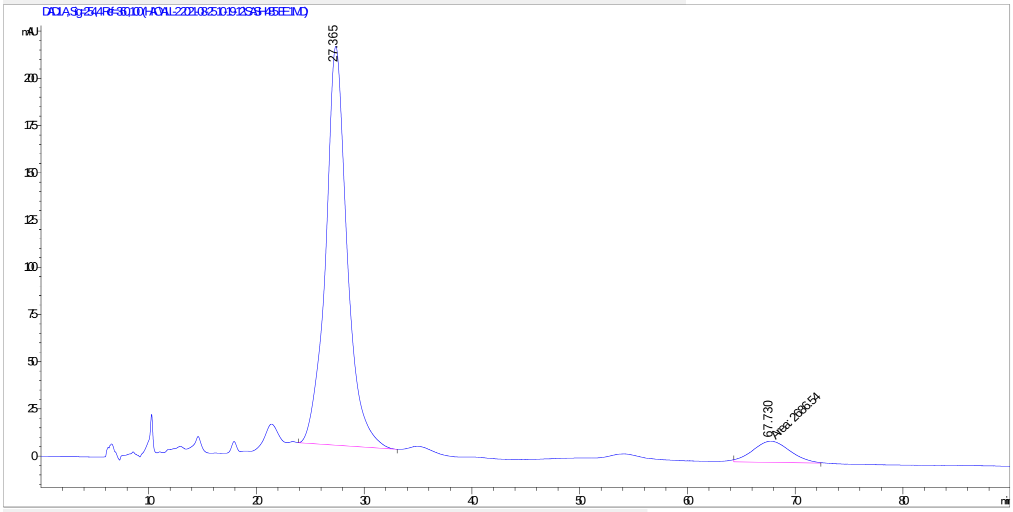

#	Time	Area	Height	Width	Area%	Symmetry
1	27.365	29433.9	211.1	1.9624	91.636	0.871
2	67.73	2686.5	11.2	3.9989	8.364	0.859

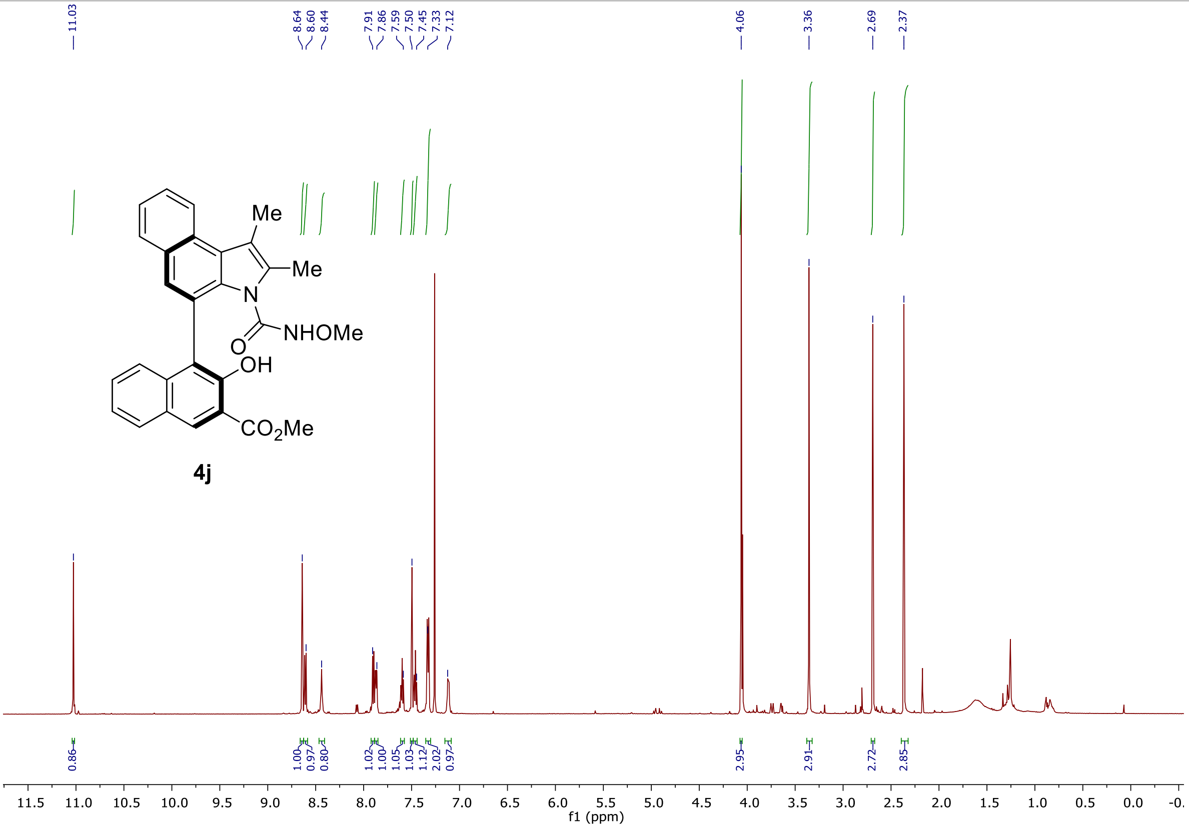

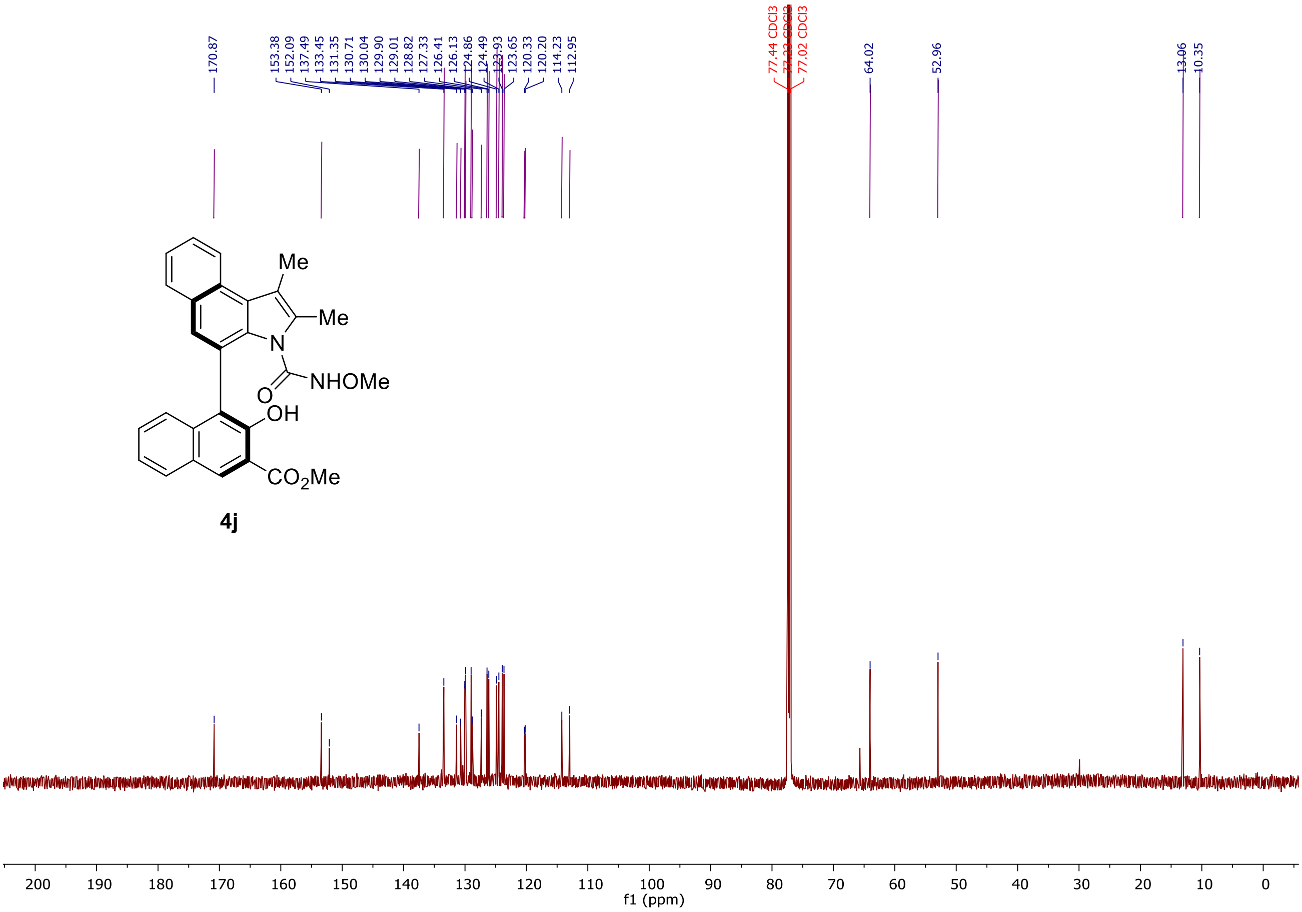

SUPPORTING INFORMATION

HPLC traces (**4j**): racemate top, enantiomer bottom:

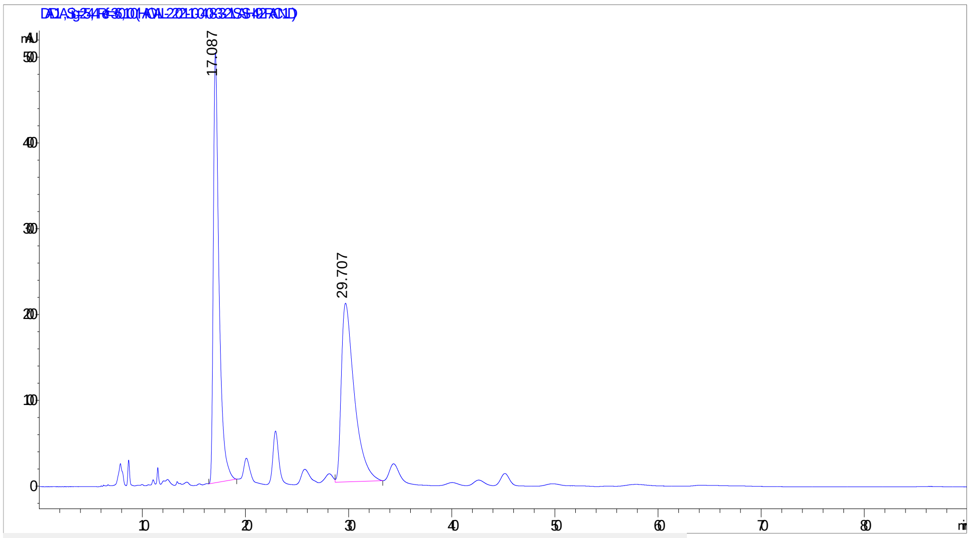

#	Time	Area	Height	Width	Area%	Symmetry
1	17.087	19241.1	501.6	0.5655	51.200	0.514
2	29.707	18339.3	208.2	1.2996	48.800	0.441

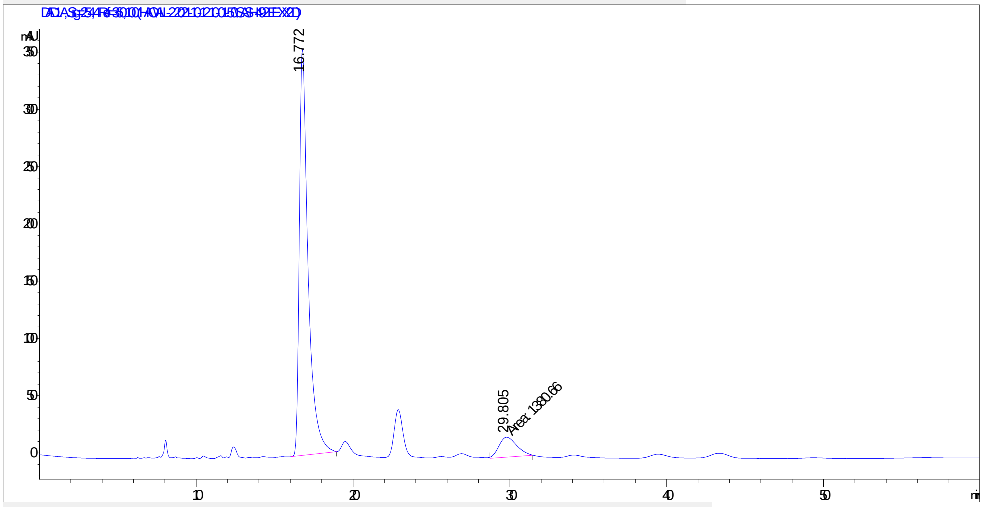

#	Time	Area	Height	Width	Area%	Symmetry
1	16.772	13778.4	354.4	0.5695	90.892	0.491
2	29.805	1380.7	17.4	1.3261	9.108	0.782

References:

[1] a) M. Kitamura, M. Kisanuki, K. Kanemura, T. Okauchi, *Org. Lett.* **2014**, *16*, 1554; b) Z-J. Jia, C. Merten, R. Gontla, C. G. Daniliuc, A. P. Antonchick, H. Waldmann, *Angew. Chem. Int. Ed.* **2017**, *56*, 2429; c) G. Shan, J. Flegel, H. Li, C. Merten, S. Ziegler, A. P. Antonchick, H. Waldmann, *Angew. Chem. Int. Ed.* **2018**, *57*, 14250.

[2] a) L. Zhang, M. Zhu, S. Ni, L. Wen, M. Li, *ACS Catal*. **2019**, *9*, 1680; b) S. Shaaban, H. Li, F. Otte, C. Strohmann, A. P. Antonchick, H. Waldmann, *Org. Lett* **2020**, *22*, 9199.

[3] Frisch, M. J.; Trucks, G. W.; Schlegel, H. B.; Scuseria, G. E.; Robb, M. A.; Cheeseman, J. R.; Scalmani, G.; Barone, V.; Mennucci, B.; Petersson, G. A.; Nakatsuji, H.; Caricato, M.; Li, X.; Hratchian, H. P.; Izmaylov, A. F.; Bloino, J.; Zheng, G.; Sonnenberg, J. L.; Hada, M.; Ehara, M.; Toyota, K.; Fukuda, R.; Hasegawa, J.; Ishida, M.; Nakajima, T.; Honda, Y.; Kitao, O.; Nakai, H.; Vreven, T.; J. A. Montgomery, J.; Peralta, J. E.; Ogliaro, F.; Bearpark, M.; Heyd, J. J.; Brothers, E.; Kudin, K. N.; Staroverov, V. N.; Keith, T.; Kobayashi, R.; Normand, J.; Raghavachari, K.; Rendell, A.; Burant, J. C.; Iyengar, S. S.; Tomasi, J.; Cossi, M.; Rega, N.; Millam, J. M.; Klene, M.; Knox, J. E.; Cross, J. B.; Bakken, V.; Adamo, C.; Jaramillo, J.; Gomperts, R.; Stratmann, R. E.; Yazyev, O.; Austin, A. J.; Cammi, R.; Pomelli, C.; Ochterski, J. W.; Martin, R. L.; Morokuma, K.; Zakrzewski, V. G.; Voth, G. A.; Salvador, P.; Dannenberg, J. J.; Dapprich, S.; Daniels, A. D.; Farkas, O.; Foresman, J. B.; Ortiz, J. V.; Cioslowski, J.; Fox, D. J. Gaussian 09, Rev E.01, Gaussian, Inc.: Wallingford CT, USA, 2013.
